# Matched Ligands for Small, Stable Colloidal Nanoparticles of Copper, Cuprous Oxide and Cuprous Sulfide

**DOI:** 10.1002/chem.202300228

**Published:** 2023-05-08

**Authors:** Bradley E. Cowie, Lisa Häfele, Andreas Phanopoulos, Said A. Said, Ja Kyung Lee, Anna Regoutz, Milo S. P. Shaffer, Charlotte K. Williams

**Affiliations:** ^1^ Department of Chemistry University of Oxford, Chemistry Research Laboratory 12 Mansfield Road Oxford OX1 3TA UK; ^2^ Department of Chemistry, Department of Materials Imperial College London London SW7 2AZ UK; ^3^ Department of Chemistry University College London 20 Gordon Street London WC1H 0AJ UK

**Keywords:** colloids, copper, ligand effects, nanoparticles, organometallics

## Abstract

This work applies organometallic routes to copper(0/I) nanoparticles and describes how to match ligand chemistries with different material compositions. The syntheses involve reacting an organo‐copper precursor, mesitylcopper(I) [CuMes]_z_ (z=4, 5), at low temperatures and in organic solvents, with hydrogen, air or hydrogen sulfide to deliver Cu, Cu_2_O or Cu_2_S nanoparticles. Use of sub‐stoichiometric quantities of protonated ligand (pro‐ligand; 0.1–0.2 equivalents vs. [CuMes]_z_) allows saturation of surface coordination sites but avoids excess pro‐ligand contaminating the nanoparticle solutions. The pro‐ligands are nonanoic acid (HO_2_CR^1^), 2‐[2‐(2‐methoxyethoxy)ethoxy]acetic acid (HO_2_CR^2^) or di(thio)nonanoic acid, (HS_2_CR^1^), and are matched to the metallic, oxide or sulfide nanoparticles. Ligand exchange reactions reveal that copper(0) nanoparticles may be coordinated by carboxylate or di(thio)carboxylate ligands, but Cu_2_O is preferentially coordinated by carboxylate ligands and Cu_2_S by di(thio)carboxylate ligands. This work highlights the opportunities for organometallic routes to well‐defined nanoparticles and the need for appropriate ligand selection.

## Graphical abstract

Ultra‐small and stable colloidal nanoparticles of copper, cuprous oxide and cuprous sulfide were synthesized exploiting the organometallic chemistry of copper mesityl. Sub‐stoichiometric quantities of X‐type carboxylate and di(thio)carboxylate ligands were used to produce colloidal nanoparticles; the nature of the ligand influences both the nanoparticle solubility and stability.

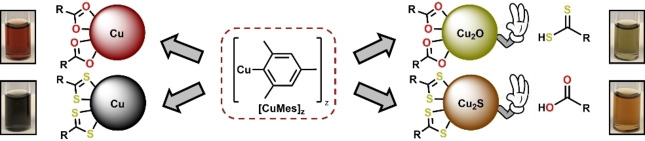

## Introduction

Nanoparticle properties depend on their morphology, size and surface chemistry; a wide range of liquid‐mediated synthetic methods are available, usually employing excess ligand or surfactant to control the structure during growth, as well as to modulate subsequent behaviors.^[1]^ This work describes a ‘platform’ synthesis methodology to prepare copper, cuprous oxide (Cu_2_O) and cuprous sulfide (Cu_2_S) nanoparticles. The syntheses apply an organocopper precursor and reactions occur in organic solvents, at low temperatures or pressures, so as to favor kinetically stabilized products.^[2]^ Chaudret and co‐workers pioneered these types of organometallic routes to colloidal nanoparticles and, in the context of this work, have produced small, soluble copper nanoparticles by exploiting the high reactivity of Cu−C bonds.^[3]^ The reaction between an organo‐copper precursor and hydrogen, in the presence of various pro‐ligands, formed soluble copper nanomaterials;^[3]^ variations of these methods were subsequently developed by other researchers.^[4]^ For example, reaction of mesitylcopper(I), [CuMes]_z_ (z=4, 5), with 4 bar hydrogen, and neutral (L‐type) amine ligands, delivered 3–5.5 nm colloidal copper(0) nanoparticles.[^3^, ^4^] The methodology contrasts with conventional routes to copper nanoparticles, such as chemical reduction of inorganic Cu(I) compounds (e. g. halides) that may be contaminated by salt byproducts, or with hot‐injection methods which require careful control over conditions and temperatures, and make larger particles.^[5]^

Copper, cuprous oxide (Cu_2_O) and cuprous sulfide (Cu_2_S) nanoparticles are useful for (electro/photo)catalysis,^[6]^ gas sensors,^[7]^ plasmonics,^[8]^ optics/electronics and in biomedical applications.^[9]^ Smaller sizes can tune the surface plasmon resonances (Cu(0)), blue‐shift the band‐gaps of semi‐conducting (cuprous oxide/sulfide) phases, and increase the specific active surface area for catalysis or sensing applications.^[10]^ For example, in electrochemical carbon dioxide reduction, ultra‐small Cu(0) NP were more active and selective than larger particles.^[11]^ Colloids of ultra‐small Cu(0) NP can be stabilized by ligands coordinated to the particle surface; most prior studies applied alkyl amine, thiolate, phosphinate or carboxylate ligands.[^3^, ^4b^, ^11b^, ^12^] A greater diversity of ligands may improve understanding of particle‐ligand interactions and provide new opportunities to modulate performance.^[1]^ Ligands covalently coordinated to the metal nanoparticle surface can be described by the well‐known covalent bond classification (CBC) theory as anionic (X‐type), neutral (L‐type) or cationic (Z‐type), depending on the donor atoms.^[13]^ Classifying the bonding modes on a heterogeneous nanoparticle surface is very challenging, and usually requires several complementary analytical methods. For example, Hens and co‐workers applied VT NMR spectroscopy to probe the relative stability of copper nanoparticles coordinated by amine (L‐type), thiolate (X‐type) and carboxylate (X‐type) ligands.^[12a]^ In reactions of the copper nanoparticles with excess ligand, relative to the available surface, amine displaced the thiolate but not oleate. The exchange chemistry was rationalized by copper surface oxidation.^[12a]^ Although NMR spectroscopy can be useful, it is fundamentally insensitive, particularly in heterogeneous systems; IR spectroscopy can be more informative, at least for coordinated ligands featuring active vibrational modes. Another consideration is that most nanoparticle syntheses apply excess neutral donor ligands (L‐type) and, as such, exchange reactions between weakly surface‐coordinated and (excess) ‘free’ ligand tend to dominate. However, there are numerous applications in which excess organic ligand contaminating solutions of particles is a disadvantage, including the in situ formation of polymer‐nanoparticle composites where excess amines/carboxylic acids are cross‐linkers and plasticizers; in electronics applications, where excess ligand may increase resistance or limit consolidation on thermal annealing; in electro/photocatalysis, where excess ligand may block active sites or undergo undesirable side‐reactions, complicating selectivity analyses. Nanoparticle syntheses that apply only sufficient ligand to stabilize the surface, whilst avoiding excess ‘free’ ligand are, therefore, attractive.

In contrast to the growing literature applying hydrogenolysis of organo‐copper precursors to make colloidal copper metal nanoparticles, there is much less work transforming organometallic precursors into ultra‐small, colloidal cuprous oxide (Cu_2_O) or sulfide (Cu_2_S) nanoparticles.^[14]^ Conceptually, there are two possible approaches: either a one step, direct oxidation/sulfidation of the precursor, or a two‐step synthesis first forming copper metal particles followed by a conversion reaction. Several researchers investigated how to suppress copper nanoparticle oxidation, seeking to prevent any reaction with oxygen to form cuprous oxide.[^3b^, ^3c^, ^15^] Accordingly, long‐chain amine ligands were noted as beneficial in ‘protecting’ the copper surfaces from oxidation.[^3b^, ^3c^, ^15^] A smaller number of investigations focused on oxidation of copper nanoparticles, prepared by hot‐injection methods (sizes ∼10 nm), to form Cu_2_O particles.^[16]^ Fau and Chaudret oxidized 2–8 nm copper nanoparticles, prepared either by hydrogenolysis of copper mesityl or amidinate precursors, with 0.1–1 equiv. of carboxylate (stearate or oleate) ligands, at 80–150 °C, to produce cuprous oxide colloids.[^3b^, ^17^] Our group oxidized ultra‐small, 1–3 nm copper nanoparticles, prepared by hydrogenolysis of copper(I)mesityl with 0.1–0.2 equiv. of oleate or di(octyl)phosphinic acid ligands, to form stable 2–5 nm cuprous oxide nanoparticle colloids. Redox cycling between Cu(0) and Cu_2_O nanoparticles by successive hydrogenolysis/air oxidation reactions was feasible.^[12b]^

There are not yet any reports of organo‐copper precursor routes to cuprous(I) sulfides. Rather, these materials are typically synthesized by hot‐injection methods using different copper and sulfur precursors; although other methods are known.^[18]^ In conventional syntheses, the most widely applied ligands are thiols (X‐type), amines (L‐type) and phosphines (L‐type) and, in common with copper nanoparticle syntheses, ligands are often applied in large excess relative to the metal sulfide.^[19]^ There is some precedent for ligand exchange reactions, for example, de Mello Donega and co‐workers exchanged hydrophobic thiols with hydrophilic alternatives to improve nanoparticle water solubility.^[19]^ Cuprous sulfides are more complex than the oxides, presenting a wide range of compositions and crystal structures; in nanoparticle form, the relative stabilities may be altered, and the size, shape and composition will all affect the band‐gap, localized surface plasmon resonance and conductivity.^[20]^ New routes to soluble, well‐defined, single phase cuprous sulfide nanoparticles may offer improvements in selectivity, processability and performance.

## Results and Discussion

**Scheme 1:**
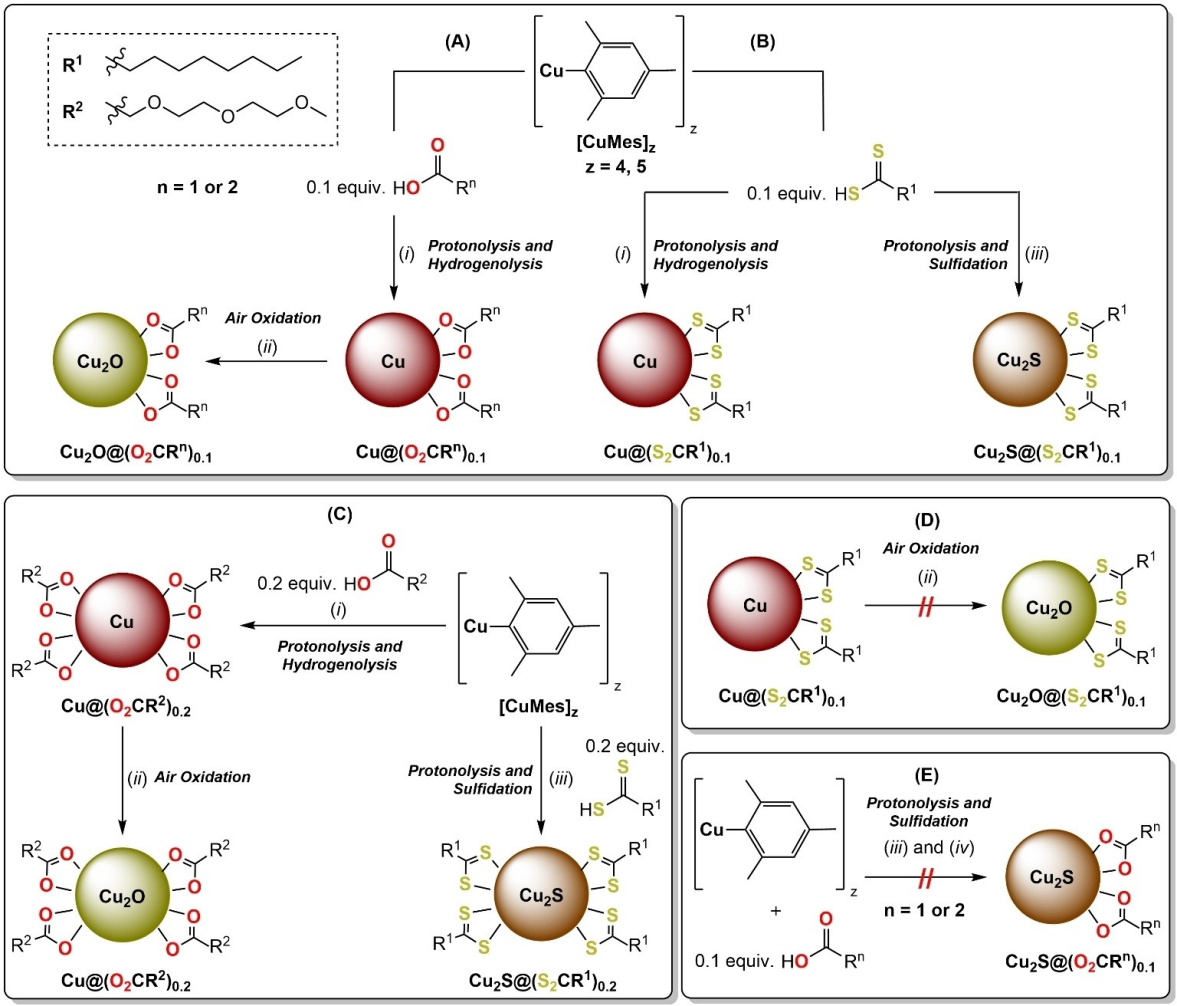
Syntheses of colloidal copper, cuprous oxide and cuprous sulfide nanoparticles exploiting the organometallic reactivity of [CuMes]_z_. **(A)** Syntheses of Cu@(O_2_CR^n^)_0.1_ and Cu_2_O@(O_2_CR^n^)_0.1_ using 0.1 equiv. of HO_2_CR^n^ (n=1 or 2). **(B)** Syntheses of Cu@(S_2_CR^1^)_0.1_ and Cu_2_S@(S_2_CR^1^)_0.1_ using 0.1 equiv. of HS_2_CR^1^. **(C)** Syntheses of Cu_2_O@(O_2_CR^2^)_0.2_ and Cu_2_S@(S_2_CR^1^)_0.2_ where 0.2 equiv. ligands loading are used. Attempted, but unsuccessful, syntheses: **(D)** Cu_2_O@(S_2_CR^1^)_0.1_ and **(E)** Cu_2_S@(O_2_CR^n^)_0.1_. Reagents and Conditions: (*i*) H_2_ (3 bar), 110 °C, 2.5 h; (*ii*) air, 20 °C, 20 h; (*iii*) 0.5 equiv. H_2_S, 100 °C, 16 h; (*iv*) 0.5 equiv. H_2_S, 20 °C, 16 h. Colloidal Cu_2_O@(O_2_CR^2^)_0.1_ were also synthesized directly from [CuMes]_z_ using 0.5 equiv. of H_2_O, in air. All reactions were conducted in toluene with a [Cu]=36 mM. HO_2_CR^1^=H_3_C(CH_2_)_7_CO_2_H (nonanoic acid); HO_2_CR^2^=H_3_C(OCH_2_CH_2_)_2_OCH_2_CO_2_H (2‐[2‐(2‐methoxyethoxy)ethoxy]acetic acid); HS_2_CR^1^=H_3_C(CH_2_)_7_CS_2_H (di(thio)nonanoic acid).

Previously, colloidal ZnO, Cu or Cu_2_O nanoparticles (NP) were synthesized by reacting organo‐zinc and/or organo‐copper complexes with water, hydrogen or air.[^11b^, ^12b^, ^12c^, ^12d^] These syntheses applied sub‐stoichiometric quantities of stearate or di(octyl)phosphinate ligands. Here, copper/cuprous nanoparticle syntheses were investigated using a single organo‐copper precursor, mesitylcopper(I), [CuMes]_z_ (z=4, 5), which was selected due to its highly reactive Cu−C bonds and because mesitylene (by‐product of protonolysis reactions) is both inert and sufficiently volatile to be removed from the nanoparticle products. It was proposed that [CuMes]_z_ would react rapidly and irreversibly with the pro‐ligands, which are acids, to form Cu−carboxylate or Cu−di(thio)carboxylate bonds. Since the ligands are applied in lower quantities, the remaining (excess) [CuMes]_z_ should react with hydrogen/air/hydrogen sulfide to form the target materials. The pro‐ligands were selected to allow comparisons between O‐ and S‐donors whilst ensuring ligands show the similar bidentate coordination modes. The ligand substituents, R, were selected to allow tuning of nanoparticle solubility from apolar (alkyl substituents) and polar (alkyl ether substituents) media. The pro‐ligands were either carboxylic acids, nonanoic acid (HO_2_CR^1^) and 2‐[2‐(2‐methoxyethoxy)ethoxy]acetic acid (HO_2_CR^2^), or a di(thio)carboxylic acid, di(thio)nonanoic acid (HS_2_CR^1^; Scheme 1). The di(thio)carboxylic acid featuring the alkyl ether substituent (i. e. HS_2_CR^2^) proved difficult to synthesize and isolate, hence was not investigated. If the syntheses tended to ligand‐saturated surfaces, the sub‐stoichiometric ratio would determine nanoparticle size. However, previous work showed that this regime is only accessible at low temperatures where nucleation is suppressed.^[21]^ Instead, the reactive organometallic precursors tend to nucleate rapidly and only become decorated with ligands towards the end of the synthesis.^[22]^ The surface coverage of the nanoparticles by the ligand then depends on the sub‐stoichiometric ratio chosen. In most cases, 0.1–0.2 equiv. of carboxylate ligand tends to favor an approximately saturated surface (see Supporting Information for details). In the first experiments, the ligand loading of 0.1 equiv. vs. [CuMes]_z_ was selected, based on previous work yielding well‐defined, soluble and stable 1–3 nm Cu@L and/or 2–5 nm Cu_2_O@L nanoparticles (L=di(octyl)phosphinate or stearate).[^11b^, ^12b^] A note on nanoparticle naming is warranted, henceforth materials are referred to as M@(E_2_CR^n^)_x_, where M=Cu, Cu_2_O or Cu_2_S; E=O or S; R^1^=alkyl, i. e. H_3_C(CH_2_)_7_ and R^2^=alkyl ether, i. e. H_3_C(OCH_2_CH_2_)_2_OCH_2_; x=# mol equiv. pro‐ligand (vs. [CuMes]_z_)=0.1 or 0.2.

### Synthesis of Colloidal Cu and Cu_2_O Nanoparticles

First, the new ligands were tested in the synthesis of copper and cuprous oxide nanoparticles; in the following section, the syntheses are described and the aggregated characterization data are subsequently compared for the different copper phases and ligands. Colloidal copper nanoparticles, Cu@(E_2_CR^n^)_0.1_, were prepared by reacting a toluene solution of [CuMes]_z_ with the appropriate pro‐ligand (0.1 equiv.), under a hydrogen atmosphere (3 bar) at 100 °C (Scheme 1).^[12b]^ The copper nanoparticles, Cu@(E_2_CR^1^)_0.1_ (E=O, S), were isolated by repeated precipitations from toluene using acetone; the precipitation was used to obtain dried powders for characterization experiments and did not result in any changes to the particle or ligand compositions (Figures S1–S3). Cu@(O_2_CR^2^)_0.1_ was stored as a 36 mM solution in toluene in the glove box, as its high solubility in both polar and apolar solvents hampered precipitation methods. The colloidal nanoparticles were characterized by powder X‐ray diffraction (PXRD), UV‐Vis spectroscopy, FTIR spectroscopy and XPS: all measurements were fully consistent with the proposed speciation and structures. All the nanoparticles, Cu@(O_2_CR^n^)_0.1_ (n=1 or 2) and Cu@(S_2_CR^1^)_0.1_, appeared stable for at least 6 months as toluene solutions, when stored under an inert (nitrogen) atmosphere (Figure S4).

Exposure of colloidal solutions of Cu@(O_2_CR^n^)_0.1_ to air, whilst stirring for 20 h at room temperature, resulted in a distinct color change from deep red to dark green, indicative of Cu_2_O NP formation (Scheme 1A and Figure 3A). Cu_2_O@(O_2_CR^1^)_0.1_ nanoparticles were isolated by precipitation from a toluene solution using acetone, whereas Cu_2_O@(O_2_CR^2^)_0.1_ was stored as a 36 mM solution in toluene in the glove box. The selective oxidation of Cu (face‐centered cubic symmetry) NP to Cu_2_O NP (primitive cubic symmetry) as opposed to CuO (monoclinic symmetry) is favored by the small size (<25 nm) of the resulting Cu_2_O nanoparticles. The oxidation does not require a change in crystal or lattice symmetry, i. e. cubic Cu forms cubic Cu_2_O, but rather results in a lattice expansion (volume expansion ∼65 %).^[23]^ The Cu_2_O NP were characterized by PXRD, UV‐Vis, FTIR, TGA and XPS, this data is discussed below.

We also tested whether a one‐step reaction between mesitylcopper(I) and H_2_O could provide colloidal Cu_2_O NP. As such, [CuMes]_z_ was treated with 0.1 equiv. of HO_2_CR^2^ and 0.5 equiv. of H_2_O. When the reaction was performed under nitrogen the oxidation was incomplete, and only reached completion upon exposure to air (Figures S5–S8). These findings indicate that water alone is insufficiently oxidizing to provide cuprous oxide nanoparticles.

Next, the influence of the ligand loading was investigated by repeating the procedure but with 0.2 equiv. of the carboxylate ligand, expected to give an approximately saturated surface (see Supporting Information). Accordingly, [CuMes]_z_ was reacted with 0.2 equiv. of HO_2_CR^n^, under a H_2_ atmosphere (3 bar, 110 °C), followed by exposure to air, at room temperature (Scheme 1C). Powder XRD showed that the resulting products were predominantly cuprous oxide nanoparticles (i. e. Cu_2_O@(O_2_CR^n^)_0.2_); in the case of bulky alkyl chains (n=1), unidentified molecular or cluster species were also formed due to the use of excess ligand (Figure S9),^[22]^ whereas for the oligoether chains (n=2), only Cu_2_O@(O_2_CR^2^)_0.2_ was detected (see below for details).

On the other hand, the attempted oxidation of the Cu@(S_2_CR^1^)_0.1_ to Cu_2_O@(S_2_CR^1^)_0.1_ following the same procedure (exposure to air) was not successful, producing a black precipitate with a yellow supernatant (Scheme 1D). The precipitate was agglomerated Cu_2_O NP (Figure S10), and the FTIR spectrum lacked the stretches associated with surface‐coordinated ligands (Figure S11), indicating that the S‐linked di(thio)carboxylate ligands were displaced during the oxidation, removing the steric colloidal protection from the nanoparticles.

### Synthesis of Colloidal Cu_2_S Nanoparticles

Copper sulfide exists in a variety of crystalline phases, Cu_2−x_S, including chalcocite (Cu_2_S), djurleite (Cu_1.94_S), digenite (Cu_1.8S_S) and anilite (Cu_1.75_S).^[24]^ One challenge is to synthesize single phases of Cu_2_S with high purity and defined stoichiometry. To investigate the potential selectivity of the low temperature organometallic synthetic routes, the reactions of [CuMes]_z_ with different sources of ‘sulfide’, such as either H_2_S and S(SiMe_3_)_2_, were investigated. The reaction of a toluene solution of [CuMes]_z_ with 0.1 equiv. of HO_2_CR^n^ and 0.5 equiv. of H_2_S, at room temperature, did not produce any cuprous sulfide nanoparticles (Scheme 1E and Figures S12–S13). For n=1, no crystalline product was detected, and for n=2, a small quantity of metallic Cu was observed by PXRD. Repeating the reaction with heating overnight at 100 °C yielded only traces of copper on the reaction vessel walls as confirmed by PXRD; in the case of n=2, only a small amount of djurleite, Cu_1.94_S, was observed (Scheme 1E and Figures S14–S15). In contrast, using di(thio)carboxylic acid pro‐ligand, HS_2_CR^1^, under analogous conditions (i. e. overnight at 100 °C), provided stable colloidal Cu_2_S@(S_2_CR^1^)_0.1_ nanoparticles as a dark orange solution (Scheme 1B). The cuprous sulfide nanoparticles were isolated by precipitation from toluene with acetone, followed by centrifugation, and the resulting XRD pattern was consistent with the formation of cuprous sulfide NP (see below). The ligand loading was also explored: applying 0.2 equiv. of HS_2_CR^1^ also yielded colloidal nanoparticles Cu_2_S@(S_2_CR^1^)_0.2_ (Scheme 1C). Similar reactions using S(SiMe_3_)_2_ as the sulfiding agent also yielded cuprous sulfide. In this case, Mes−SiMe_3_ was identified as the major by‐product (Figure S16) and pure Cu_2_S@(S_2_CR^1^)_0.1_ NP were isolated by precipitation from toluene solutions using dried and distilled acetone. All Cu_2_S colloids were characterized by PXRD, UV‐Vis, FTIR, TGA and XPS, and are discussed below.

### Powder X‐ray Diffraction (PXRD), Electron Diffraction and Transmission Electron Microscopy (TEM) Measurements

**Figure 1:**
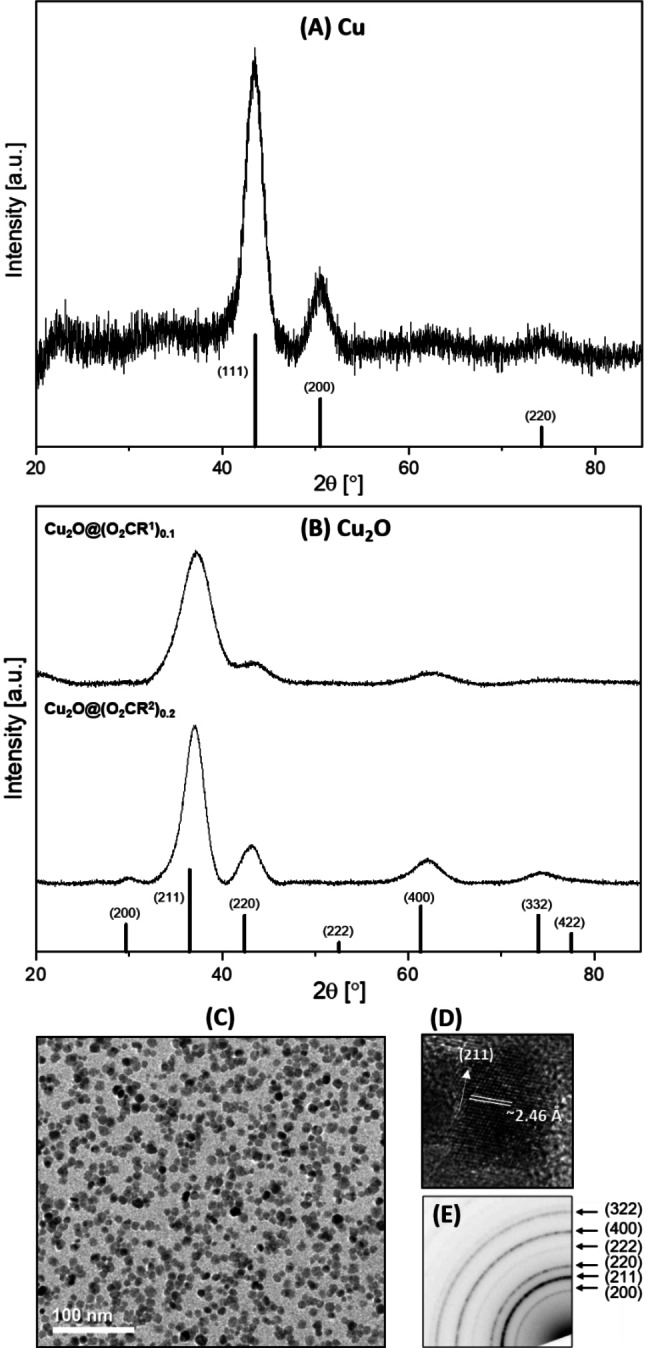
Powder XRD patterns of: **(A)** Cu@(O_2_CR^1^)_0.1_ (3.9 nm; pattern indexed against Cu as vertical bars (JCPDS 01‐085‐1326)), and **(B)** Cu_2_O@(O_2_CR^1^)_0.1_ (top, 2.4 nm) and Cu_2_O@(O_2_CR^2^)_0.2_ (bottom, 3.1 nm); patterns indexed against Cu_2_O as vertical bars (JCPDS 00‐002‐1067; Cu Kα radiation). **(C)** Cu_2_O@(O_2_CR^2^)_0.1_ annular bright field STEM image, **(D)** HRTEM image with lattice fringes and **(E)** SAED pattern.

The Cu, Cu_2_O and Cu_2_S NP were characterized using a range of techniques; each is discussed in turn to aid comparisons between materials. In all cases, powder XRD measurements were important to characterize the materials’ speciation and phase. All the copper nanoparticles, i. e. Cu@(E_2_CR^n^)_0.1_ (E=O, n=1 or 2; E=S, n=1), showed broad diffraction peaks at 44° (111), 51° (200) and 74° (220) which are indexed to cubic copper (Figure 1A and S17–S19). The crystallite sizes are 3.9, 3.1 and 3.0 nm for Cu@(O_2_CR^1^)_0.1_, Cu@(O_2_CR^2^)_0.1_ and Cu@(S_2_CR^1^)_0.1_, respectively, as estimated by Scherrer analysis.

The XRD patterns for the Cu_2_O@(O_2_CR^n^)_0.1_ (n=1 or 2) and Cu_2_O@(O_2_CR^2^)_0.2_ nanoparticles were consistent with the formation of cubic Cu_2_O. All patterns showed broad diffraction peaks at 30 (200), 37 (211), 42 (220), 53 (222), 62 (400) and 74° (332), in line with the expected reference patterns (Figure 1B and S20–S22). The average particle sizes, determined using Scherrer analysis, were quite similar with Cu_2_O@(O_2_CR^1^)_0.1_ being 2.4 nm and Cu_2_O@(O_2_CR^2^)_x_ being 3.1 nm, for both x=0.1 and 0.2. The particles show smaller sizes, by XRD, after oxidation which is initially surprising as oxidation leads to a lattice expansion and hence notionally a larger particle. However, if the oxidation process leads to the formation of additional twin or grain boundaries in the particles, the coherent crystallite size estimated by applying the Scherrer equation to the PXRD data will shrink. In fact, multiple grains are visible in many of the oxide particles in the STEM images (Figures S24B, S26C and S26D). Previously, on cycling between Cu and Cu_2_O,^[12b]^ we also observed multiple grains, and the emergence of a skin‐core texture, showing that even in these small particles, oxidation initiates at the particle surface, and occurs relatively slowly. It is, therefore, reasonable to suppose that it can initiate in more than one location on the copper particle surface, leading to increasing polycrystallinity. The earlier work also showed the same decrease in crystallite (particle) size between Cu@stereate and Cu_2_O@stearate, specifically it reduced from 3.2–3.4 nm to 2.8–3.4 nm after oxidation.^[12b]^

Transmission electron microscopy (TEM) analysis was performed on Cu_2_O@(O_2_CR^n^)_0.1_ (n=1, 2; Figures 1 and S23–S25) and Cu_2_O@(O_2_CR^2^)_0.2_ (Figure S26), and showed an average particle size of 5.6±1.1 nm, 8.7±1.7 nm and 9.2±4.6 nm, respectively. The significantly smaller crystallite size of particles measured by XRD, compared to that observed by TEM can again be rationalized by twin or grain boundaries within the nanocrystals (Figure S26c). Such crystallite effects were previously observed for cuprous oxide nanoparticles, prepared using organometallic chemistry, when using high‐resolution TEM (HR‐TEM).[^12b^, ^25^]

To determine if there is a correlation between ligand length and resulting particle size, the average particle sizes for Cu and Cu_2_O NPs with nonanoate (O_2_CR^1^, 9 carbon atoms) and stearate (18 carbon atoms) ligands are compared. The average size of the Cu@(O_2_CR^1^)_0.1_ NPs was 3.9 nm by PXRD (Scherrer analysis), whereas the average size of the Cu_2_O@(O_2_CR^1^)_0.1_ NPs was 2.4 nm by PXRD (Scherrer analysis) and 5.6±1.1 nm by TEM (see above). The average size of the previously reported Cu@stearate NPs was 3.3 nm by PXRD (Scherrer analysis), and the average particle size of Cu_2_O@stearate by STEM was ∼6 nm.^[12b]^ The particle sizes are very similar using either nonanoate or stearate ligands. Thus, the ligand length does not appear to control the resulting nanoparticle sizes.

**Figure 2:**
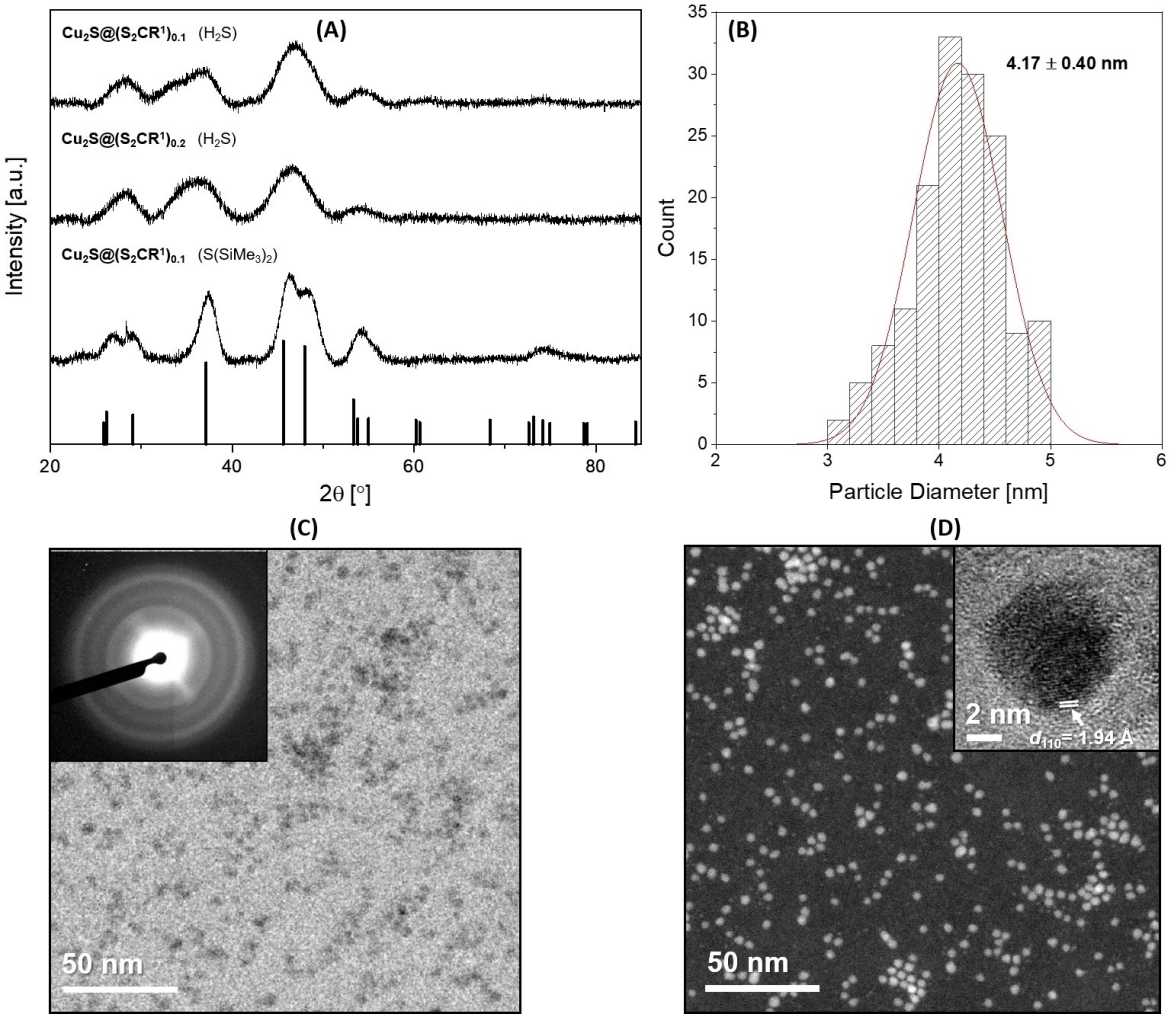
**(A)** Powder XRD pattern of Cu_2_S@(S_2_CR^1^)_0.1_ synthesized using H_2_S (top, 2.3 nm), Cu_2_S@(S_2_CR^1^)_0.2_ synthesized using H_2_S (middle, 2.4 nm), and Cu_2_S@(S_2_CR^1^)_0.1_ synthesized using S(SiMe_3_)_2_ (bottom, 3.9 nm); patterns indexed against hexagonal chalcocite as black vertical bars (JCPDS 01‐084‐0208; Cu Kα radiation). **(B)** Size distribution histogram obtained from the TEM data for Cu_2_S@(S_2_CR^1^)_0.1_ synthesized using H_2_S. **(C)** Annular bright field STEM image of Cu_2_S@(S_2_CR^1^)_0.1_ synthesized using H_2_S; SAED pattern provided in the inset. **(D)** Annular dark field STEM image with an HRTEM image of a single Cu_2_S@(S_2_CR^1^)_0.1_ NP, synthesized using H_2_S, with lattice fringes provided in the inset.

The cuprous sulfide NPs (Cu_2_S@(S_2_CR^1^)_x_; x=0.1 and 0.2) synthesized using H_2_S as the sulfiding agent showed XRD patterns with broad reflections at 28° (100) (002) (101), 37° (102), 47° (110) (103), and 55° (112) (004) (201); Scherrer analysis showed particles with very similar sizes, at 2.3 and 2.4 nm for x=0.1 and 0.2, respectively. The Cu_2_S@(S_2_CR^1^)_0.1_ synthesized from S(SiMe_3_)_2_ is slightly larger in size, forming particles of 3.9 nm (Figure 2A and S27), consistent with a sharper powder XRD pattern with reflections at 27° (100) (002), 29° (101), 37° (102), 46° (110), 48° (103), 54° (112) (004) (201) and 74° (210) (114) (211) (105) (Figure 2A). The powder XRD patterns are most consistent with the formation of hexagonal chalcocite, Cu_2_S (Figures 2A and Supporting Information). Djurleite, Cu_1.94_S, is often observed during the attempted synthesis of Cu_2_S and can be difficult to differentiate from either monoclinic or hexagonal chalcocite by powder XRD, especially for small crystallites,^[19]^ given the overlap and degree of broadness of strong diffraction peaks at ∼38, 46 and 49°. The main difference between the powder XRD pattern of djurleite and those found for the Cu_2_S@(S_2_CR^1^)_x_ (x=0.1 and 0.2) NPs is the relative intensity of the diffraction peak at 28°, which matches the reference pattern of hexagonal chalcocite most closely (Figures 2A and S27–S29).

To further differentiate between the formation of Cu_2_S (hexagonal chalcocite) or Cu_1.94_S (djurleite), the SAED pattern of Cu_2_S@(S_2_CR^1^)_0.1_ was analyzed. The sample showed diffraction rings at 2.97 nm^−1^ (d=3.37 Å (002)), 4.01 nm^−1^ (d=2.50 Å (102)) and 5.07 nm^−1^ (d=1.97 Å (110); Figures 2C and S30). The position of the (110) ring is in agreement with that reported in the literature for hexagonal chalcocite (5.02 nm^−1^),^[26]^ but djurleite is reported to display an electron diffraction ring at d=3.39 Å.^[27]^ Therefore, the electron diffraction data does not conclusively differentiate between chalcocite or djurleite, or a mixture of both phases, for the Cu_2_S@(S_2_CR^1^)_x_ (x=0.1, 0.2). Next, UV‐Vis‐NIR and band gap measurements were pursued for further verification (see below for discussion).

TEM analysis of Cu_2_S@(S_2_CR^1^)_0.1_ (prepared using H_2_S) showed an average particle size of 4.2±0.4 nm, a size range of 3–5 nm (Figure 2B−D), and HRTEM analyses showed a lattice spacing of 1.94 Å, which corresponds to the (110) lattice fringe for hexagonal chalcocite (Figure 2D).^[28]^ TEM analysis of Cu_2_S@(S_2_CR^1^)_0.1_, when prepared using S(SiMe_3_)_2_, showed an average particle size of 5.0±1.0 nm, consistent with the larger crystallite size observed by PXRD, and a size range of 2–8 nm (Figure S31).

### UV‐Vis Spectrophotometry

**Figure 3:**
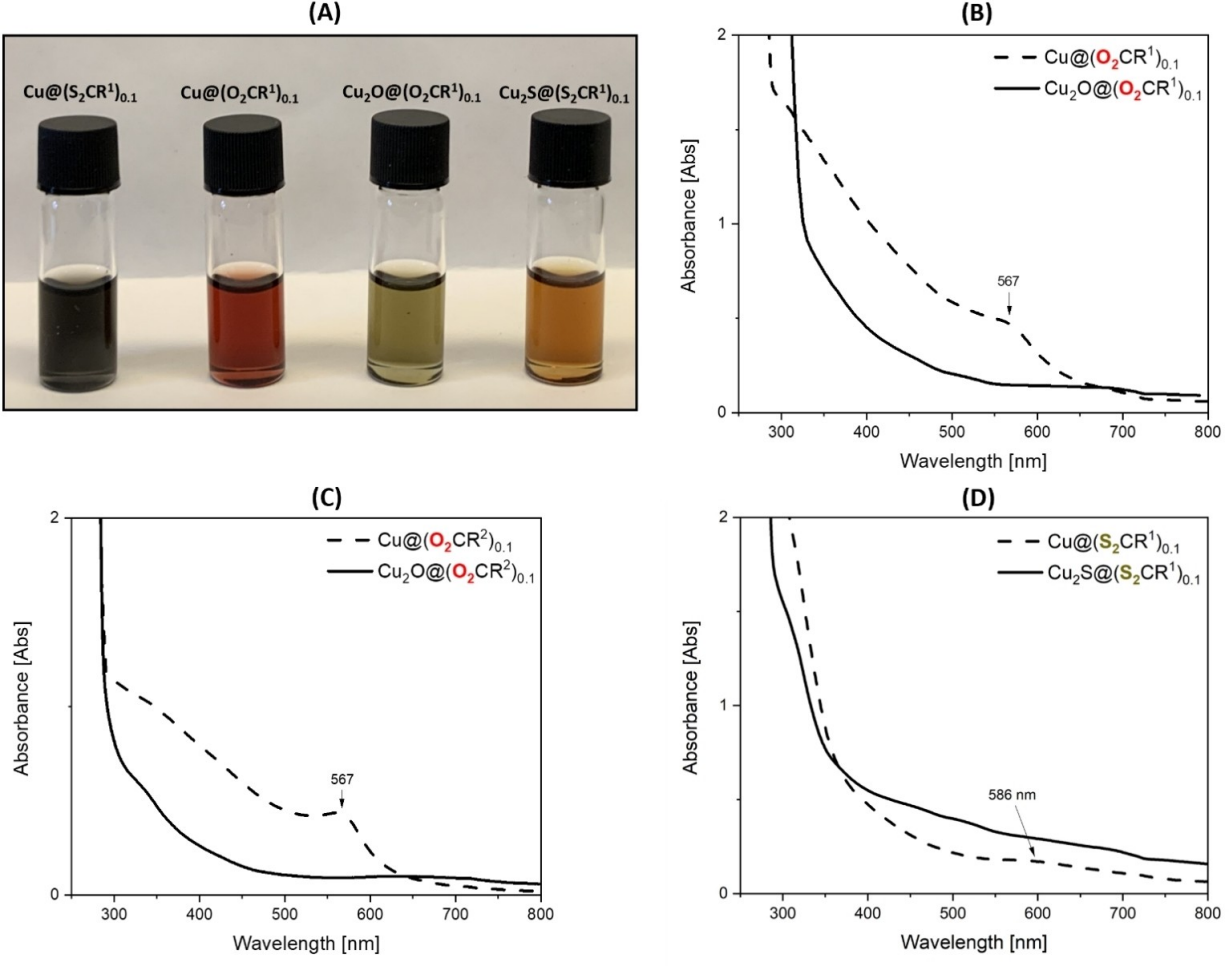
**(A)** Solutions of Cu@(S_2_CR^1^)_0.1_, Cu@(O_2_CR^1^)_0.1_, Cu_2_O@(O_2_CR^1^)_0.1_ and Cu_2_S@(S_2_CR^1^)_0.1_ (left to right), and UV‐Vis spectra of: **(B)** Cu@(O_2_CR^1^)_0.1_ and Cu_2_O@(O_2_CR^1^)_0.1_ (0.5 mM in toluene), **(C)** Cu@(O_2_CR^2^)_0.1_ and Cu_2_O@(O_2_CR^2^)_0.1_ (0.5 mM in toluene), and **(D)** Cu@(S_2_CR^1^)_0.1_ (0.5 mM in toluene) and Cu_2_S@(S_2_CR^1^)_0.1_ (0.3 mM in toluene).

UV‐Vis spectrophotometry of red Cu@(O_2_CR^n^)_0.1_ toluene solutions showed a characteristic surface plasmon resonance (SPR) at 567 nm which is consistent with other copper nanoparticles, coordinated by carboxylate ligands (Figures 3A–C and Supporting Information).^[12b]^ In contrast, toluene solutions of the copper nanoparticles with di(thio)carboxylate ligands, Cu@(S_2_CR^1^)_0.1_ were very dark colored/black and showed the SPR at higher wavelength, 586 nm (Figures 3A, 3D and Supporting Information). A similar red‐shifting of the SPR was previously reported for copper nanoparticles coordinated by thiolate ligands.^[29]^ The changes to the copper nanoparticles’ SPR with different ligands may relate to stronger coordination by the S‐ligands compared with O‐ligands.

The formation of Cu_2_O@(O_2_CR^n^)_0.1_ was confirmed by the disappearance of the copper SPR in the resulting UV‐Vis spectra (Figures 3B, 3C and Supporting Information). Toluene solutions of Cu_2_O@(O_2_CR^n^)_0.1_ (n=1 or 2) and Cu_2_O@(O_2_CR^2^)_0.2_ are emerald green and displayed absorption onsets from ∼725–750 nm (Figures 3B, 3C and S35–S37). While there are a variety of methods to determine optical band‐gaps particularly where absorption onsets are of relatively low intensity, simple Tauc plots provide useful estimates here. Accordingly, the optical band‐gaps were estimated at 2.0–2.2, 2.3–2.4 and 2.4–2.5 eV for Cu_2_O@(O_2_CR^n^)_0.1_ (n=1 or 2) and Cu_2_O@(O_2_CR^2^)_0.2_, respectively (Figures S38–S43). For Cu_2_S@(S_2_CR^1^)_0.1_, UV‐Vis spectrophotometry provides another opportunity to investigate the cuprous sulfide phase, as chalcocite and djurleite possess distinctly different optical band‐gaps. A toluene solution of Cu_2_S@(S_2_CR^1^)_0.1_ showed an absorption onset from 720–730 nm, giving rise to a band‐gap of ∼1.6 eV (Figure 3D and Supporting Information), which is consistent with that calculated for hexagonal chalcocite (1.49 eV)^[30]^ and greater than that calculated for either monoclinic Cu_2_S (1.39 eV) or djurleite Cu_1.94_S (1.12 eV).^[30]^ The optical band‐gap of Cu_2_S@(S_2_CR^1^)_0.2_ was determined to range from 1.5–1.6 eV, again most closely aligned with that for hexagonal chalcocite phases (Figures S47–S49). Djurleite gives rise to an SPR in the near‐IR between 0.4 and 0.8 eV, whereas chalcocite does not,[^20b^, ^27^] thus near IR spectroscopy offers an additional means to differentiate speciation. The near IR spectroscopy cannot be conducted in toluene solutions since it absorbs in the same region as the djurleite SPR (Figure S50), thus the nanoparticles were dissolved in chloroform solutions instead. A 4.5 mM solution of Cu_2_S@(S_2_CR^1^)_0.2_ in chloroform did not show any SPR in the near‐IR spectrum (Figures S51–S52), further supporting the formation of hexagonal chalcocite cuprous sulfide.

### FTIR Spectroscopy

Fourier transform infrared spectroscopy (FTIR) is useful for characterization of the ligand coordination since pro‐ligand (i. e. not coordinated) and coordinated ligands typically show distinct absorption energies.

**Figure 4:**
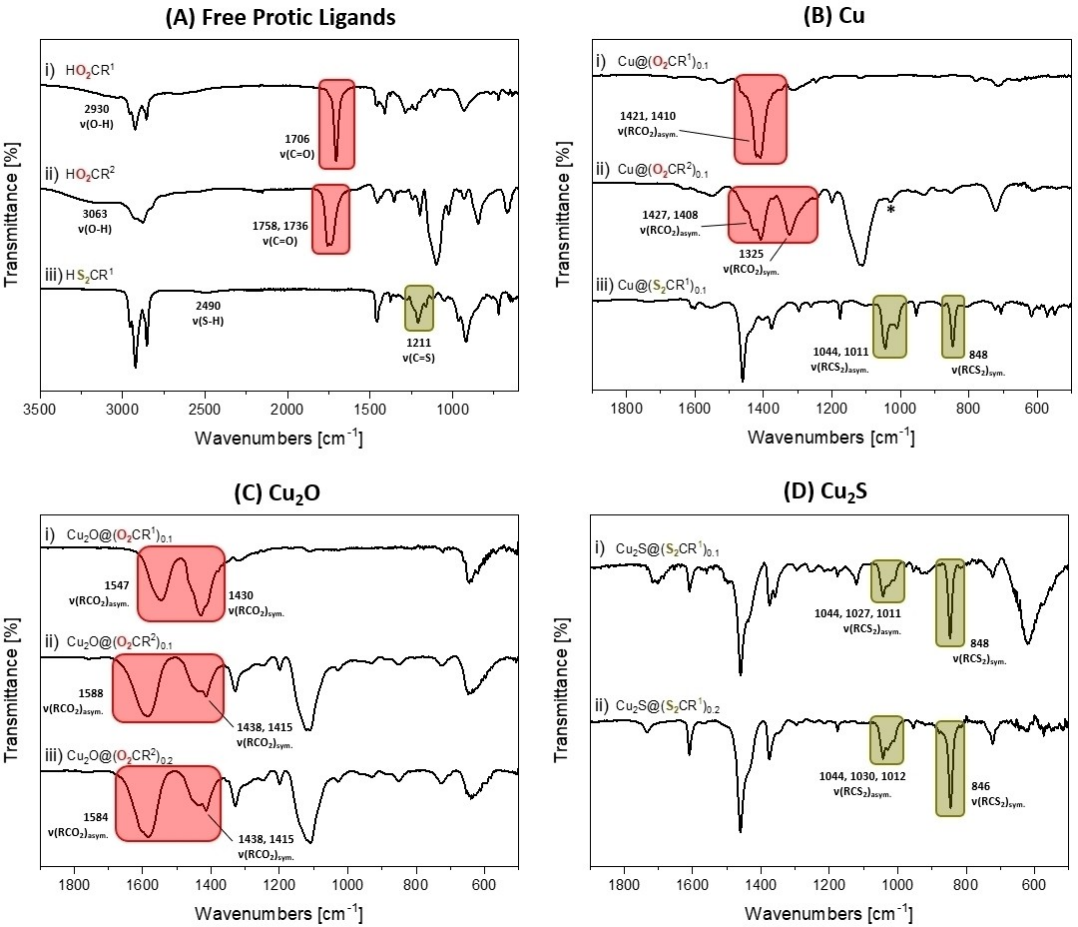
Stacked FTIR spectra of: **(A)** (i) HO_2_CR^1^, (ii) HO_2_CR^2^ and (iii) HS_2_CR^1^, **(B)** (i) Cu@(O_2_CR^1^)_0.1_, (ii) Cu@(O_2_CR^2^)_0.1_ and (iii) Cu@(S_2_CR^1^)_0.1_, **(C)** (i) Cu_2_O@(O_2_CR^1^)_0.1_, (ii) Cu_2_O@(O_2_CR^2^)_0.1_ and (iii) Cu_2_O@(O_2_CR^2^)_0.2_, and **(D)** (i) Cu_2_S@(S_2_CR^1^)_0.1_ and (ii) Cu_2_S@(S_2_CR^1^)_0.2_. The FTIR spectra in **(A)** span from 3500–600 cm^−1^, whereas those in **(B)‐(D)** are expanded from 1900–500 cm^−1^ for clarity in the fingerprint region. Red boxes highlight diagnostic ν(C=O) and ν(RCO_2_) stretches, and green boxes highlight diagnostic ν(C=S) and ν(RCS_2_) stretches. *=mesitylene; asym.=asymmetric stretch; sym.=symmetric stretch. Table S3 summarizes the key stretches observed by FTIR spectroscopy for the free protic acids and isolated nanoparticles.

Its short timescale improves sensitivity and may improve differentiation between ‘free’ and coordinated ligands, or equilibria, in solution. In contrast, despite the benefits of solution NMR, it is challenging to apply to colloidal nanoparticles due to the associated resonance broadening. Indeed, the ^1^H NMR spectra of Cu_2_O@(O_2_CR^n^)_0.1_ (n=1, 2) and Cu_2_S@(S_2_CR^1^)_0.1_ were very poorly resolved (Figures S53–S59). The copper NPs with carboxylate ligands, Cu@(O_2_CR^n^)_0.1_, show both asymmetric and symmetric carboxylate stretches at 1421/1410 cm^−1^ for the ligands featuring the alkyl chain and at 1427/1408 and 1325 cm^−1^ for the ligands with oligoether chains (Figure 4B and Supporting Information). The spectra do not contain any stretches associated with pro‐ligands (i. e. free carboxylic acids) confirming the formation of ligated particles free from any excess ligand (HO_2_CR^1^: ν(O−H)=2930 cm^−1^, ν(C=O)=1706 cm^−1^; HO_2_CR^2^: ν(O−H)=3063 cm^−1^, ν(C=O)=1758, 1736 cm^−1^; Figure 4A and Supporting Information). The copper nanoparticles with di(thio)carboxylate ligands, Cu@(S_2_CR^1^)_0.1_, show diagnostic asymmetric and symmetric di(thio)carboxylate stretches, as expected at lower frequency compared with the O‐ligands, at 1044/1011 and 848 cm^−1^, respectively (Figure 4B and S64). The spectra do not contain any stretches for the free di(thio)carboxylic acid pro‐ligand (HS_2_CR^1^: ν(S−H)=2490 cm^−1^, ν(C=S)=1211 cm^−1^, Figure 4A and S65).^[31]^ It is not feasible to unambiguously assign the copper oxidation state on the basis of the IR spectroscopy data, but one interpretation of the data is that the X‐type ligands are coordinated to Cu(I) species at the nanoparticle surface.

The FTIR spectra of the cuprous oxide samples, i. e. Cu_2_O@(O_2_CR^n^)_0.1_ (n=1 or 2) and Cu_2_O@(O_2_CR^2^)_0.2_, showed asymmetric and symmetric carboxylate stretches at higher wavenumbers than for the analogous Cu NPs. For example, the carboxylate stretches are observed at 1547 and 1430 cm^−1^ for Cu_2_O@(O_2_CR^1^)_0.1_, at 1588 and 1430/1415 cm^−1^ for Cu_2_O@(O_2_CR^2^)_0.1_, and 1584 and 1438/1415 cm^−1^ for Cu_2_O@(O_2_CR^2^)_0.2_ for the asymmetric and symmetric carboxylate stretches, respectively (Figure 4C and Supporting Information). The spectra do not show any absorptions for free carboxylic acid pro‐ligands, indicating the carboxylates remain coordinated to the particle surface after oxidation. The IR stretches for the carboxylate ligands are within the range of values for copper(I) and copper(II) carboxylates, which show absorptions at 1690–1540 (asymmetric) and 1410–1315 cm^−1^ (symmetric), respectively.^[32]^ All the cuprous oxide NP show broad absorption bands, at 3154, 3425 and 3374 cm^−1^, assigned to surface hydroxyl moieties (O−H stretches; Figures S66–S68). Further, the intensity of these hydroxyl stretches is greater for the Cu_2_O NP coordinated by the carboxylates with oligoether substituents. These stretches are tentatively assigned to water/hydroxyl groups coordinated at the particle surfaces, with the increase in intensity reflecting the greater ligand hydrophilicity (as inferred from solubility data).

The FTIR spectrum of Cu_2_S@(S_2_CR^1^)_0.1_ shows stretches at 1044, 1027 and 1011 cm^−1^, assigned as the asymmetric stretches for the di(thio)carboxylate and a stretch at 848 cm^−1^, assigned as the symmetric stretch (Figure 4D and Supporting Information). Literature copper(I) dithiocarboxylates show asymmetric and symmetric di(thio)carboxylate stretches from 1140–1020 and 970–815 cm^−1^, respectively.^[33]^ Similarly, the FTIR spectrum of Cu_2_S@(S_2_CR^1^)_0.2_ shows its asymmetric and symmetric stretches at 1044/1030/1012 and 846 cm^−1^, respectively (Figure 4D and S71). The IR spectra do not feature any free di(thio)carboxylic acid stretches (ν(S−H)=2490 cm^−1^, ν(C=S)=1211 cm^−1^, Figure 4A), indicating their complete transformation into surface coordinated ligands (Figure 4 and Table S3).

### Thermal Gravimetric Analysis (TGA)

Another technique to determine ligand coordination is thermal gravimetric analysis (TGA) since the organic ligands are typically thermolyzed at lower temperatures than the inorganic materials. All Cu NPs were analyzed, in sealed aluminum pans, under nitrogen. The TGA data for Cu@(O_2_CR^n^)_0.1_ (n=1, 2) showed ∼24 and 26 % mass loss, respectively, which is in good agreement with expected values (25 and 28 wt.% for n=1 and 2, respectively). The mass loss for Cu@(S_2_CR^1^)_0.1_ was 21 %, which is significantly lower than the expected value (30 wt.%). The discrepancy is attributed to incomplete volatilization of the di(thio)carboxylate group when experiments were conducted under N_2_, leading to the formation of copper−sulfide species on the NP surface (Figure S72).

**Figure 5:**
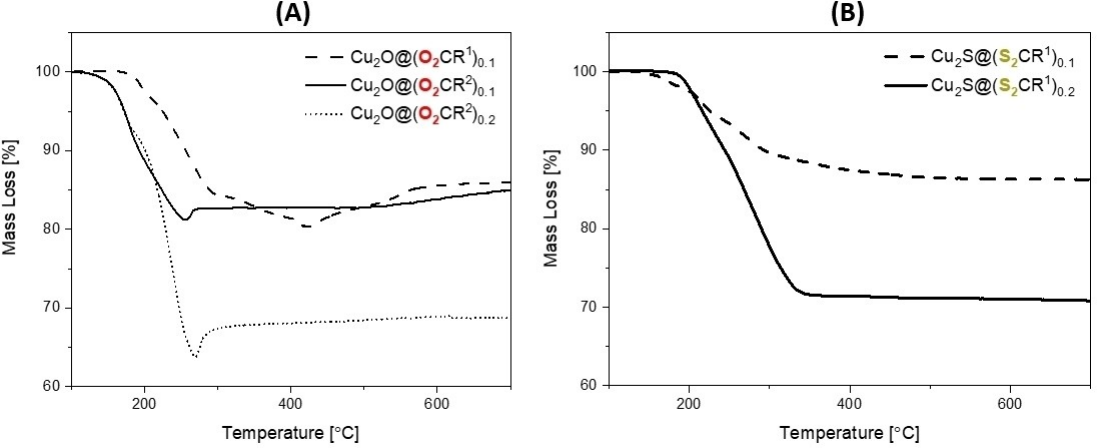
Thermal gravimetric analyses (TGA) for: **(A)** Cu_2_O@(O_2_CR^1^)_0.1_, Cu_2_O@(O_2_CR^2^)_0.1_ and Cu_2_O@(O_2_CR^2^)_0.2_, and **(B)** Cu_2_S@(S_2_CR^1^)_0.1_ (synthesized from S(SiMe_3_)_2_) and Cu_2_S@(S_2_CR^1^)_0.2_ (synthesized from H_2_S).

All Cu_2_O NPs were analyzed in air, and the TGA data for Cu_2_O@(O_2_CR^1^)_0.1_ shows an ∼20 % mass loss from ∼170–400 °C (Figure 5A and S73). Over this temperature range the cuprous oxide can be decomposed to copper and, therefore, the combined (ligand+oxide loss) theoretical mass loss values (22 wt.%) agree well with those determined experimentally (20 wt.%). Cu_2_O@(O_2_CR^2^)_0.1_ shows thermal degradation from 112–256 °C, corresponding to 19 % mass loss, again with values being consistent with calculated values (ligand+oxide reduction=24 wt.%; Figure 5A and S74). The particles with higher ligand loadings, Cu_2_O@(O_2_CR^2^)_0.2_, show corresponding greater mass loss (∼36 wt.%), once again values correspond closely with theoretical values (36 wt.%, Figure 5A and S75). Consistent with proposed cuprous oxide thermal reduction to copper, the samples show a 1–5 wt.% mass increase at temperatures above 250 °C (for R^2^) or >400 C (for R^1^), consistent with partial re‐oxidation to cuprous oxide (complete Cu oxidation=11–12 wt.%; Figure 5A).

The TGA data are consistent with the IR spectroscopic data and indicate ligand surface coordination to the particles. The cuprous oxide thermal reduction to copper has also previously been noted in the literature.^[12a]^ The oligoether ligands undergo lower temperature thermal decompositions than the corresponding ligands with alkyl substituents likely due to the volatile oxygen‐containing degradation byproducts during thermolysis of the oligoether ligands. Reducing the ligand degradation temperature may be useful, for example allowing efficient deposition of colloidal nanoparticle films from solution, followed by ligand removal and particle sintering for applications in catalysis or sensing.

The TGA data for the cuprous sulfide, Cu_2_S, NPs were collected under nitrogen atmosphere. Cu_2_S@(S_2_CR^1^)_0.1_, synthesized using S(SiMe_3_)_2_, undergoes a mass loss of 13 wt.% from 140–430 °C (Figure 5B and S76), in line with the ligand loading (expected=12 wt.%). The Cu_2_S@(S_2_CR^1^)_0.1_, synthesized from H_2_S, shows a 3.2 wt.% mass loss below 100 °C, perhaps due to loss of surface‐coordinated −SH species, followed by the expected ligand mass loss, 14 wt.% from 200–430 °C, which is in agreement with the calculated value (Figure S77). At higher ligand loading, Cu_2_S@(S_2_CR^2^)_0.2_ shows a mass loss of 29 wt% from 175–350 °C (Figure 5B and S78), consistent with predicted values (24 wt.%). The thermal reduction of the cuprous sulfides to copper is not observed, unlike the more easily reduced Cu_2_O phase.^[34]^

### X‐ray Photoelectron Spectroscopy (XPS)

**Figure 6:**
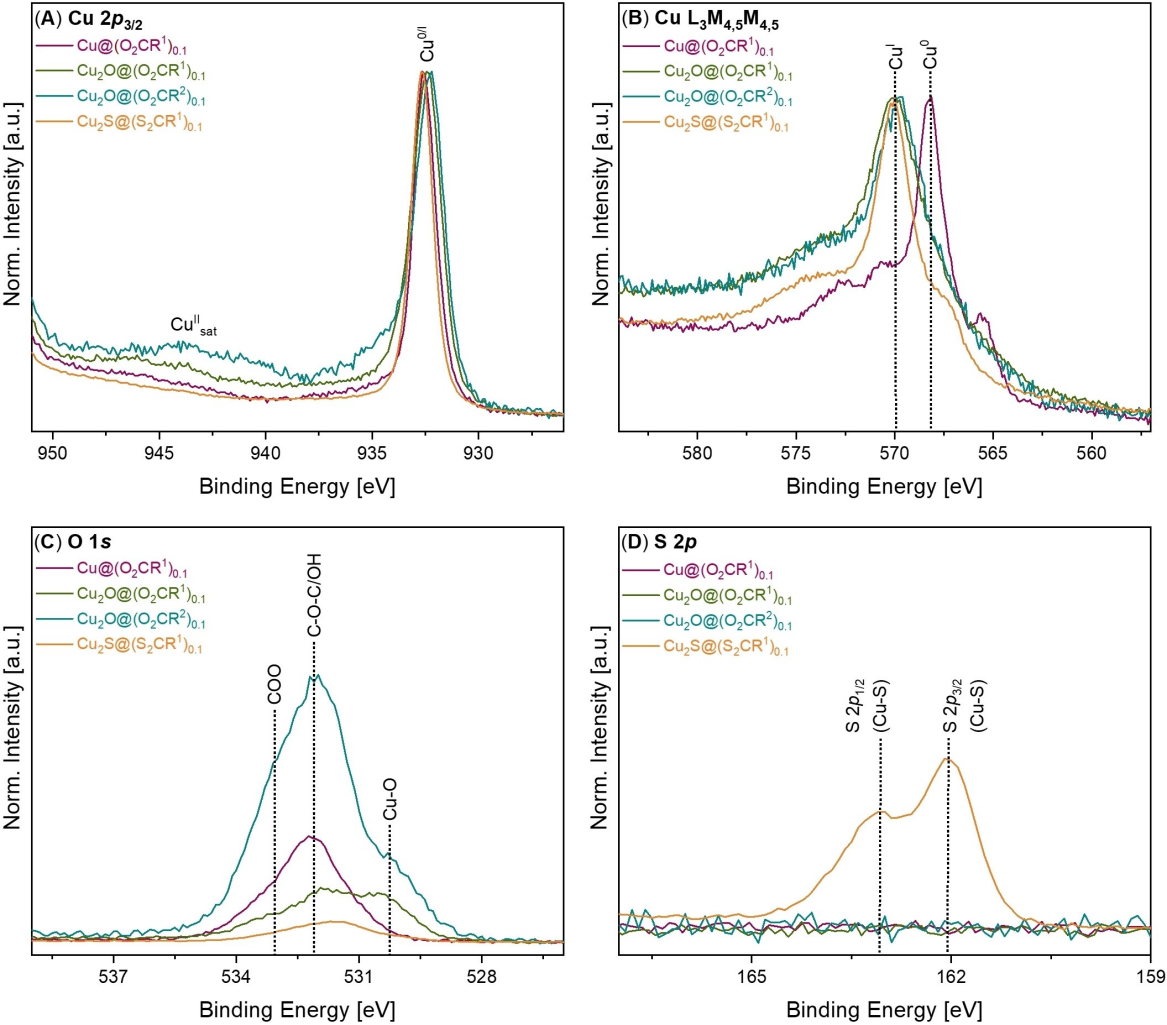
X‐ray photoelectron spectra, including: (**A**) Cu 2*p*_3/2_, (**B**) Cu L_3_M_4,5_M_4,5_, (**C**) O 1s, and (**D**) S 2*p*.

XPS is used to investigate particle surface speciation and the ligand‐particle interface. The copper nanoparticles coordinated by the alkyl carboxylates, Cu@(O_2_CR^1^)_0.1_, show signals consistent with metallic copper, i. e. Cu(0) (Figure 6A and 6B). Whilst the Cu 2*p*_3/2_ core level cannot be used to distinguish between Cu^0^ and Cu^I^ due to a lack of observable chemical shift (Figures 6A and S79–S84), the main Cu L_3_M_4,5_M_4,5_ Auger line shows strong differences. The sample shows an energy, as well as line shape, typical of metallic Cu (Figure 6B and S85). The other copper nanoparticle samples suffered from partial oxidation under the XPS experimental conditions (Figures S86–S87). Nonetheless, in all cases the expected ligand environments were confirmed (i. e. O*‐*containing carboxylate or S‐containing di(thio)carboxylate coordination; Figure 6 and S91–S98). Samples with the oligoether ligand show O 1*s* core spectra with larger signals than those containing the alkyl carboxylate, as expected given the former's higher oxygen content (Figure 6C and Supporting Information). The C 1*s* spectra also show the expected ligand chemistries, including the presence of ether C−O bonds and carboxylate signals (Figure S99).^[35]^

The cuprous oxide samples, Cu_2_O@(O_2_CR^1^)_0.1_ and Cu_2_O@(O_2_CR^2^)_0.1_, both show the Cu 2*p*_3/2_ core level and the Cu L_3_M_4,5_M_4,5_ Auger spectra at typical bonding energies for Cu_2_O (Figures 6A and B).^[36]^ The samples show a small feature in the Cu 2*p*_3/2_ core level to higher BE of the main photoionization peak, as well as a satellite feature at a binding energy (BE) above 940 eV, indicating some oxidation to Cu(II) on the particle surfaces (Figure 6A and Supporting Information).[^36b^, ^37^]

The XPS spectra for Cu_2_S@(S_2_CR^1^)_0.1_ confirm the Cu(I) bonding energies (Figure 6). Well‐defined signals from the sulfur environments are also detected (Figure 6D).[^36b^, ^38^] From peak fits to the Cu 2*p*_3/2_ and S 2*p* spectra of Cu_2_S@(S_2_CR^1^)_0.1_ a Cu : S ratio of ∼2:1 was in good agreement with the theoretical 2 : 1.2 stoichiometry.

### Nanoparticle Solubility Studies

The cuprous oxide NPs with carboxylate ligands (Cu@(O_2_CR^n^)_0.1_) showed good solubilities in a range of solvents with distinct differences between the ligands. The Cu_2_O@(O_2_CR^2^)_0.1_ (i. e. with the oligoether substituents) showed much higher solubility in a range of solvents, specifically showing maximum solubilities of 200, 27, 38 and 34 mg/mL in toluene, THF, acetone and methanol, respectively. In comparison, Cu_*2*_O@(O_2_CR^1^)_0.1_ showed solubility values of 2.3 and 3.2 mg/mL in toluene and THF, respectively, which diminished after successive cycles of solvent removal and re‐dispersion, and was insoluble in acetone or methanol. The Cu_2_S@(S_2_CR^1^)_0.1_ NPs had solubilities of 24 (toluene), 20 (THF) and 1.0 mg/mL (acetone), respectively, and were insoluble in methanol (see Supporting Information for Experimental Details).

### Ligand Exchange Reactions

The colloidal Cu_2_O nanoparticles were only prepared when carboxylate ligands were used and di(thio)carboxylate ligands failed to yield equivalent colloidal NPs. The opposite finding was observed for Cu_2_S nanoparticles, with stable colloidal NP only resulting from use of di(thio)carboxylate ligands. This relationship between ligand donor identity and colloidal nanoparticle stability was explored further through ligand exchange reactivity. For these investigations, cuprous oxide and cuprous sulfide nanoparticles were prepared using 0.2 equiv. carboxylate or di(thio)carboxylate ligands, respectively, to saturate the particles’ surfaces (see Supporting Information for the estimated surface coverage and associated characterization data). These NPs were then exposed to the same loading of the complementary ligand, in solution, and various techniques were used to assess both the speciation and ligand coordination chemistries.

**Figure 7:**
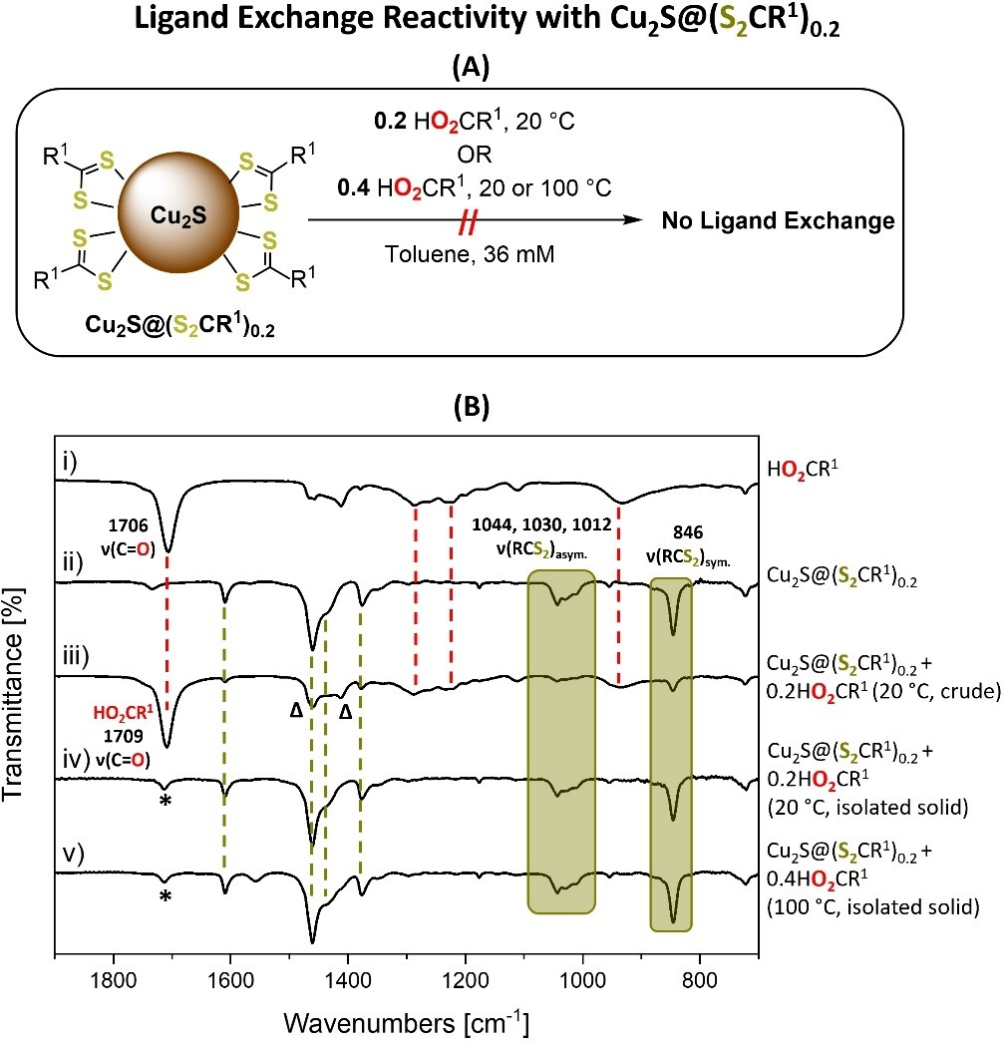
**(A)** Ligand exchange reactions between Cu_2_S@(S_2_CR^1^)_0.2_ and 0.2 or 0.4 equiv. HO_2_CR^1^. **(B)** Stacked FTIR spectra of: (i) HO_2_CR^1^, (ii) Cu_*2*_S@(S_2_CR^1^)_0.2_, (iii) crude reaction product Cu_2_S@(S_2_CR^1^)_0.2_+0.2 equiv. of HO_2_CR^1^ at 20 °C, (iv) isolated product (Cu_2_S@(S_2_CR^1^)_0.2_+0.2 equiv. of HO_2_CR^1^ at 20 °C), and (v) isolated product (Cu_2_S@(S_2_CR^1^)_0.2_+0.4 equiv. of HO_2_CR^1^ at 100 °C). Asymmetric and symmetric ν(RCS_2_) stretches are highlighted in green, and diagnostic stretches of HO_2_CR^2^ and Cu_2_S@(S_2_CR^1^)_0.2_ are indicated by red and green dotted lines, respectively. *=Small quantities of HO_2_CR^1^ that remain after product purification. Δ=Stretches from free HO_2_CR^1^ that overlap with coordinated [S_2_CR^1^].

First, a toluene solution of Cu_2_S@(S_2_CR^1^)_0.2_ was treated with HO_2_CR^1^ (0.2 equiv.) at room temperature (Figure 7A). The reaction solution showed no color change and analysis by FTIR spectroscopy showed absorptions for the starting material, Cu_2_S@(S_2_CR^1^)_0.2_, as well as stretches for the free carboxylic acid. Specifically, the diagnostic asymmetric ν(RCS_2_) stretches at 1044, 1030 and 1012 cm^−1^, and the symmetric ν(RCS_2_) stretch at 846 cm^−1^ were observed. Further, the diagnostic C=O stretch for the free carboxylic acid was observed at 1709 cm^−1^ (Figure 7B and S102). The nanoparticles were then isolated, by precipitation from the toluene solution with acetone; the product was cuprous sulfide with di(thio)carboxylate ligands. It was characterized by XRD, showing retention of the hexagonal chalcocite phase and particle size of 2.6 nm (starting size=2.4 nm by Scherrer analysis; Figures 7B and S103–S104). TGA measurements showed a ligand mass loss (26 %, 200–390 °C) in line with the expected dithiocarboxylate ligand loading (24 wt.%; Figure S105). Next, the Cu_2_S@(S_2_CR^1^)_0.2_ was treated, under more forcing conditions, with an excess of HO_2_CR^1^ (0.4 equiv.) at both room temperature and 100 °C. Hexagonal chalcocite, Cu_2_S, was retained, as verified by PXRD (Figure S106), and no ligand exchange or decomposition occurred, as evidenced by FTIR spectra (Figures S107–S110). The FTIR spectra of the crude products showed absorptions for the di(thio)carboxylate ligand and free carboxylic acid (Figures S107–S108). Moreover, after isolation of the Cu_2_S@(S_2_CR^1^)_0.2_, the mother liquor was reduced to dryness and analyzed using ^1^H NMR spectroscopy which showed signals for the unreacted (free) carboxylic acid, HO_2_CR^1^ (Figures S111–S112). TGA measurements of the isolated nanoparticles showed a mass loss (27 %, 175–400 °C) as expected from the dithiocarboxylate ligand loading (Expected=24 wt.%; Figure S113). Altogether, these reactions demonstrate that the carboxylic acid cannot displace the di(thio)carboxylate ligand from Cu_2_S@(S_2_CR^1^)_0.2_.

**Figure 8:**
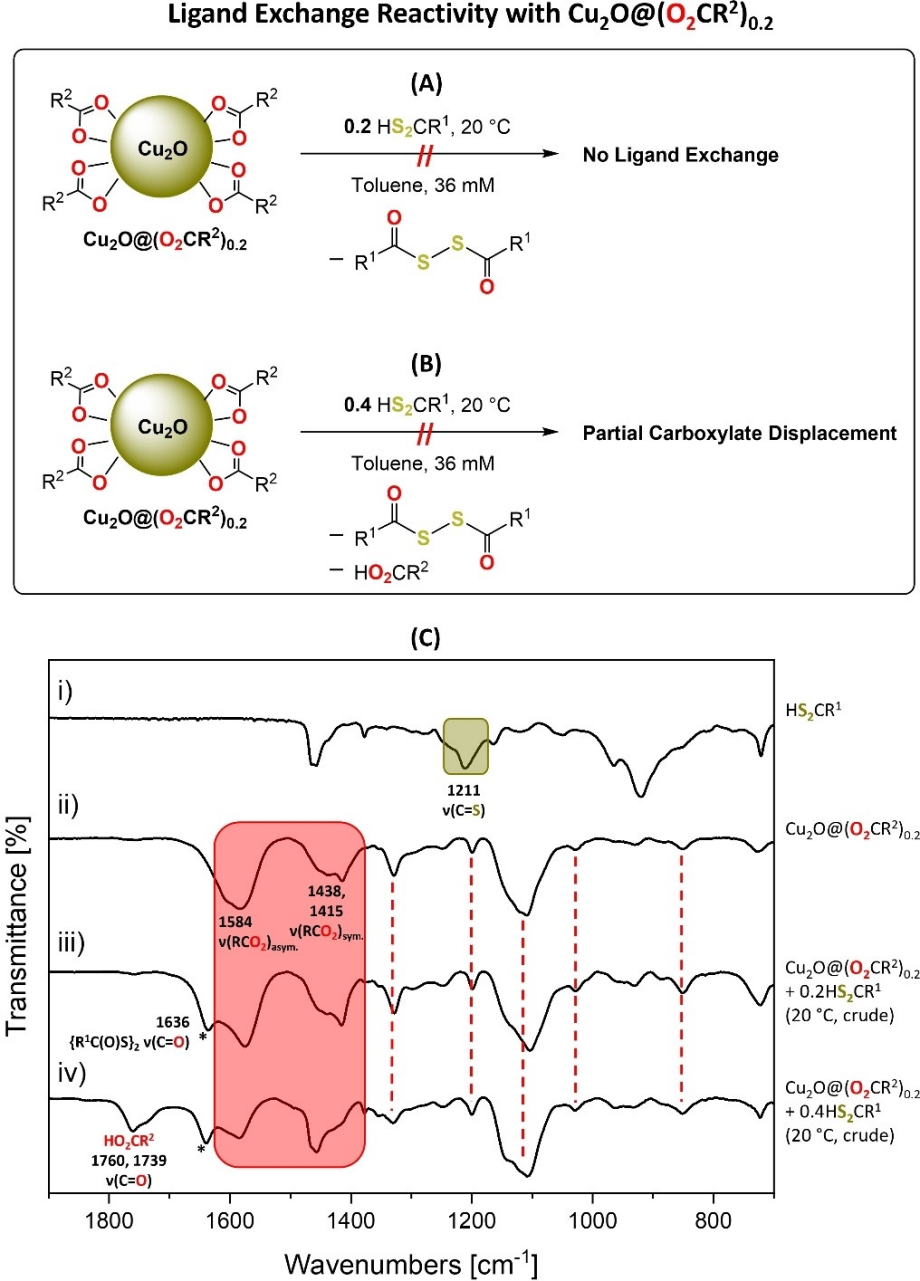
Ligand exchange reactivity using Cu_2_O@(O_2_CR^2^)_0.2_ and (A) 0.2 equiv. of HS_2_CR^1^ at 20 °C and (B) 0.4 equiv. of HS_2_CR^1^ at 20 °C. (C) Stacked FTIR spectra of: (i) HS_2_CR^1^, (ii) Cu_*2*_O@(O_2_CR^2^)_0.2_, (iii) Crude product (Cu_2_O@(O_2_CR^2^)_0.2_+0.2 equiv. of HS_2_CR^1^ at 20 °C) and (iv) Crude product (Cu_2_O@(O_2_CR^2^)_0.2_+0.4 equiv. of HS_2_CR^1^ at 20 °C, expanded from 1900–700 cm^−1^). Asymmetric and symmetric ν(RCO_2_) stretches are highlighted in red, the ν(C=S) stretch is highlighted in green, and other diagnostic Cu_2_O@(O_2_CR^2^)_0.2_ stretches indicated by red dotted lines. * New ν(C=O) stretch assigned as di(nonanoyl)disulfide.

The complementary series of reactions were also undertaken between cuprous oxide NPs, Cu_2_O@(O_2_CR^2^)_0.2_, with 0.2 equiv. of di(thio)carboxylic acid ligands, HS_2_CR^1^, in toluene at room temperature (Figure 8A). Although there was a color change from dark green to brown/orange, all characterization data indicated that cuprous oxide remained coordinated by carboxylate ligands (Figure 8C). After the reaction, the product XRD peak positions were identical to the starting cuprous oxide, showing no change in phase, and a similar particle size of 3.5 nm was observed (Figure S114, starting sample=3.4 nm). Additionally, the FTIR spectrum of the product matched that of pure Cu_2_O@(O_2_CR^2^)_0.2_ NPs (Figure 8C). One curiosity was that the crude spectrum did not show signals for free di(thio)carboxylic acid, rather it showed an unexpected extra stretch at 1636 cm^−1^ (Figure 8C), and loss of the broad stretch at ∼3400 cm^−1^ associated with surface −OH groups (Figure S115).

The crude product was analyzed by ^13^C{^1^H} NMR spectroscopy which showed a singlet at 168 ppm (Figure S116). Taken together, the IR and ^13^C NMR data indicate the formation of a diacyl disulfide (see Table S4 for comparison with literature diacyl disulfides). It is tentatively proposed that the free di(thio)carboxylic acid HS_2_CR^1^ decomposed to the diacyl disulfide, perhaps via a radical process assisted by the Cu(I) hydroxyl species or oxygen, likely leading to the observed color change;^[39]^ di(thio)carboxylic acids are known to be unstable.^[31]^ Treatment of the Cu_2_O@(O_2_CR^2^)_0.2_ with excess of di(thio)carboxylic acid (0.4 equiv.) at room temperature and/or with heating to 100 °C did not yield any cuprous oxide coordinated by di(thio)carboxylate ligands (Figure 8B). Once again, PXRD showed the retention of cubic Cu_2_O phase and FTIR spectroscopy indicated only carboxylate ligand surface coordination (Figures 8Civ and S117–S120). The characteristic disulfide signal was again observed, suggesting the same side‐reactions occurred with the di(thio)carboxylic acid. The IR spectra also showed low intensity signals attributed to trace ‘free’ carboxylic acid indicating that although some ligand may be re‐protonated in the presence of the di(thio)carboxylic acid (which is a stronger acid), there is not any ligand exchange (Figure 8C).

## Discussion

This work demonstrates a series of organometallic routes, conducted at lower temperatures in organic solvents, to make well‐defined, small copper, cuprous oxide and cuprous sulfide nanoparticles. The nanoparticle speciation and size, determined using XRD and TEM, showed the clean formation of 2–4 nm copper, cuprous oxide and cuprous sulfide nanoparticles. Interestingly, the sulfide synthesis selectively forms the stoichiometric chalcocite phase, with two different sulfide sources (H_2_S, and S(SiMe_3_)_2_). The resulting nanoparticles are surface coordinated by X‐type carboxylate or di(thio)carboxylate ligands, as indicated by IR spectroscopy and by XPS measurements. The ligand substituents were either alkyl or oligoether chains, with the latter producing substantially more soluble nanoparticles in both apolar and polar media (up to 200 mg/mL). The IR spectroscopy showed only signals for carboxylate or di(thio)carboxylate moieties, with no evidence for free pro‐ligand being present. The opportunity to avoid excess free ligand could be an advantage for applications in catalysis, sensing, (opto)electronics or multifunctional composites.

The colloidal metallic Cu(0) nanoparticles were successfully stabilized by the coordination of either type of O‐ or S‐donor ligands. However, the cuprous oxide nanoparticles were stabilized only by the carboxylate ligands; di(thio)carboxylate pro‐ligands failed to yield any colloidal nanoparticle product, and showed no ligand exchange with carboxylate ligated cuprous oxide nanoparticles. Conversely, the cuprous sulfide nanoparticles were stabilized only by the di(thio)carboxylate ligand; carboxylate pro‐ligands failed to yield any colloidal sulfide nanoparticle product, and showed no ligand exchange with di(thio)carboxylate ligated cuprous sulfide nanoparticles, even under forcing conditions. These data confirm that the ligand must be appropriately‐matched to the nanoparticle composition. The preference of a particular surface to select for a similar chalcogen seems somewhat unexpected given that both ligands coordinate effectively to copper(0). Clearly, the Cu(I/II) surface coordination is influenced by the other neighboring atom(s) in the crystal structure. One possibility is that the Cu_2_O nanoparticle surface is more ionic and hence more oxophilic, whereas the equivalent surface for Cu_2_S is more covalent and hence has increased thiophilicity.^[40]^ It is possible that the ionic structure of Cu_2_O is better suited to ‘hard’ donors like carboxylic acids. Conversely, the covalency of Cu_2_S favors coordination by softer, sulfur‐containing ligands, such as a di(thio)carboxylate. Alternatively, Cu_2_O stabilization by carboxylate ligands and Cu_2_S stabilization by di(thio)carboxylate ligands may be associated with the relative O⋅⋅⋅O or S⋅⋅⋅S separations in the RCO_2_ and RCS_2_ ligands, respectively, and their corresponding relationship to the copper cation separations on the Cu_2_O and Cu_2_S surfaces. It is feasible to estimate the separations from molecular structures, determined by X‐ray crystallography, of model Cu(I) complexes of dithiocarboxylate and carboxylate ligands, respectively. Accordingly, the S⋅⋅⋅S distance was determined as ∼2.9 Å from the structure of [Cu(S_2_CCy)(PPh_3_)_2_] (Cy=cyclohexyl) (distances determined using Mercury v.2022.3.0).^[41]^ The S⋅⋅⋅S separation matches the estimated Cu⋅⋅⋅Cu separations in Cu_2_S (∼2.7 Å),^[42]^ suggesting the ligand could effectively bridge two Cu(I) cations on the NP surface. In contrast, the estimated O⋅⋅⋅O separation in the carboxylate ligand is ∼2.2 Å, as determined from [Cu_3_(μ_3_‐OH)(μ‐pz)_3_(O_2_C(CH_2_)_5_Me)_2_]_2_(μ‐C_10_H_8_N_2_) (pz=pyrazolate).^[43]^ The O⋅⋅⋅O separation is significantly shorter than the estimated Cu⋅⋅⋅Cu surface separation in Cu_2_S, perhaps preventing the ligand adopting a bridging coordination mode. One limitation of this argument is, however, the finding that the O⋅⋅⋅O separation is also significantly shorter than the estimated Cu⋅⋅⋅Cu separation on the surface of Cu_2_O (∼3 Å).^[44]^ Therefore, if a bridging coordination mode between two Cu(I) cations was the dominant coordination mode, the carboxylate ligand would appear to be unlikely to effectively stabilize the Cu_2_O NP. It is, however, likely that the carboxylate and di(thio)carboxylate ligands can adopt other coordination modes, for example mono‐ or bidentate coordination to single Cu(I) on the NP surface in which case ligand donor atom separations are less relevant. As a result, it appears most appropriate to rationalize the ligand coordination preferences between the nanoparticles by the relative ligand donor atom oxo/thiophilicity and match to the hardness/softness of the Cu_2_O vs. Cu_2_S surface Cu(I) sites.

There are relatively few other studies exploring ligand exchange reactivity at copper nanoparticle surfaces, and those reported often applied a very large ligand excess. For example, Hens and co‐workers showed that excess undecanoic acid ligands displaced oleyl amine from 4 nm colloidal copper nanoparticles.^[12a]^ They proposed carboxylate binding occurred to an ultrathin cuprous oxide surface on the copper nanoparticles. In another piece of work, Hens and co‐workers investigated the exchange processes of CdSe or PbSe nanoparticles coordinated by oleate ligands, with short‐chain alcohols (i. e. MeOH, EtOH and ^*i*^PrOH): they observed dynamic ligand exchange at the NP surface.^[45]^ Owen and co‐workers exchanged oleate ligands coordinated to CdSe, CdS, PbSe or PbS nanoparticles with L‐type primary alkylamines.^[46]^ The alkylamines displaced the carboxylate groups, although the extent of ligand exchange was dependent on ligand concentration, steric profile, denticity, and chemistry. Hens and co‐workers showed the successful displacement of neutral L‐type octadecylamine from the surface of CuInS_2_ nanoparticles by 2‐phenylethanethiol at elevated temperatures. Equivalent reactions with oleic acid failed to result in any ligand exchange.^[47]^ In comparison to these prior studies, using different materials, this work systematically compares the stabilization afforded to various Cu/Cu(I) surfaces by two similar X‐type ligands, both featuring the same alkyl chain substituents and both capable of surface stabilization by bidentate coordination. The ligands differ only in the O‐ or S‐donor atoms and hence allow for insights into the ligand binding affinities using different donor atoms. In addition, the use of sub‐stoichiometric quantities of ligands in the exchange reactions allows for ligand surface chemistry to be investigated free from excess pro‐ligand. This chemistry depends upon the direct coordination of the ligand to the surface metal atoms; the binding modes are quite different to non‐covalent interactions between carboxylic acids and surface hydroxides through hydrogen bonding.

## Conclusions

Small, well‐defined colloidal nanoparticles of Cu, Cu_2_O and Cu_2_S were synthesized by organometallic reactions from mesitylcopper(I) precursor ([CuMes]_z_; z=4, 5). The syntheses applied sub‐stiochiometric quantities of carboxylic acid or di(thio)carboxylic acids as directly coordinated ligands and allow for comparisons between O‐ or S‐donors. The Cu(0) nanoparticles were effectively coordinated by either oxygen‐containing carboxylate or sulfur‐containing di(thio)carboxylate ligands, as evidenced by a range of X‐ray diffraction, spectroscopic and thermal methods. In contrast, Cu_2_O nanoparticles must be coordinated by carboxylate ligands and cuprous sulfide by di(thio)carboxylate ligands, respectively. Ligand exchange investigations further underscore the importance of ligand ‘selection’ and matched surface chemistry. The use of excess carboxylic acid as a ligand or surfactant is very common in metal oxide nanochemistry yet may present disadvantages for application. Thus, to better inform ligand‐surface interactions, the concepts of sub‐stoichiometric ligand loadings and chemically informed surface‐carboxylate matching explored in this work should be investigated for other metal oxides, including zinc oxide and other doped metal oxides. There is a wealth of nanoparticle chemistry exploiting metal sulfides/selenides that may benefit from use of di(thio)carboxylate ligands and their future investigation is recommended, particularly where bidentate coordination modes may be more effective than the widely applied thiolates. There is significant scope to broaden and generalize the ligand chemistries, for example applying carboxylate or di(thio)carboxylates with controllable steric features or by using other donors, for example phosphinic acids/thiophosphinic acids. The investigation of thiocarboxylates would allow for either anionic O‐ or S‐coordination chemistry and should also be prioritized. More generally, exploiting organometallic reactivity allows for the efficient, low temperature synthesis of well‐defined nanoparticle ‘inks’ which should be applied to facilitate deposition of metals, oxides and sulfides for a range of applications.

## Experimental Section

**General Materials and Methods**: All manipulations, aside from the oxidation of Cu@(O_2_CR^n^)_x_ (n=1, x=0.1; n=2, x=0.1 or 0.2) in air, were conducted inside a nitrogen‐filled glove box or using a double‐manifold Schlenk line, equipped with a nitrogen atmosphere.^[48]^ In order to use hydrogen gas for the synthesis of Cu@(O_2_CR^n^)_x_ and Cu@(S_2_CR^1^)_0.1_, a double manifold Schlenk line, equipped with a hydrogen atmosphere, was constructed. Prior to its use, both manifolds were evacuated under dynamic vacuum while heated. Once cooled to room temperature, both manifolds remained under dynamic vacuum overnight. Immediately prior to use, hydrogen gas was bubbled through the Schlenk line for 10 min to remove any residual moisture in the H_2_ manifold. Toluene was obtained from a solvent purification system and stored, under an N_2_ atmosphere over molecular sieves (3 Å), in an ampoule equipped with a Young's tap. Acetone was dried over molecular sieves (3 Å), distilled and stored in an ampoule equipped with a Young's tap prior to use. CS_2_ was degassed and stored, under N_2_ atmosphere, in an ampoule equipped with a Young's tap. 1‐Bromooctane was purchased from Sigma Aldrich and used without further purification. Magnesium turnings were purchased from Alfa Aesar and stirred under dynamic vacuum prior to use. S(SiMe_3_)_2_ was purchased from Sigma Aldrich, distilled under dynamic vacuum and stored in a glove box prior to use. Nonanoic acid (HO_2_CR^1^) was purchased from Sigma Aldrich, degassed and stored in the glove box prior to use. 2‐[2‐(2‐methoxyethoxy)ethoxy]acetic acid (HO_2_CR^2^) was purchased from Sigma Aldrich, distilled under dynamic vacuum and stored in the glove box prior to use. Mesitylcopper(I), [CuMes]_z_ (z=4, 5), was purchased from Strem Chemicals, recrystallized from toluene (80 mL for 5 g of [CuMes]_z_) at −30 °C and stored in the glovebox freezer; [CuMes]_z_ is thermally and photochemically sensitive. The synthesis of di(thio)nonanoic acid (HS_2_CR^1^) was adapted from a literature procedure.^[31]^ H_2_S was obtained as a lecture bottle (300 grams, ≥99.5 %) from Sigma Aldrich. An H_2_S solution in d_8_‐toluene was prepared by adding H_2_S (1 bar) to degassed d_8_‐toluene (degassed by 3 freeze‐pump‐thaw cycles; ∼25 mL) in an ampoule equipped with a Young's tap, which was then sealed and stirred overnight at room temperature. Afterwards, the H_2_S atmosphere was removed by replacing the atmosphere inside the ampoule with N_2_ over the course of 30 min, and the concentration of H_2_S in d_8_‐toluene was quantified periodically by ^1^H NMR spectroscopy using P(C_6_H_4_OMe‐*p*)_3_ as an internal standard. A trap equipped with a three‐way adapter containing a solution to destroy excess thiol (1 : 1 ratio of 82 % water, 10 % 1‐butanol, 5 % sodium dodecyl sulphate, 3 % cyclohexane:5 % sodium hypochlorite solution) was used to neutralize any excess H_2_S used during the procedure. All labware exposed to S(SiMe_3_)_2_ or H_2_S was rinsed thoroughly with 5 % sodium hypochlorite solution following their use. A Sigma 2–6E centrifuge (SciQuip; 3900 rpm) was used for the isolation of Cu, Cu_2_O and Cu_2_S nanoparticles following their precipitation from toluene solutions using dry, degassed acetone.

^1^H and ^13^C NMR spectra were obtained on Bruker AV‐400 and 600 MHz spectrometers, respectively, and referenced relative to SiMe_4_ (0 ppm) through a resonance of the deuterated solvent used, or proteo impurity of the solvent (C_6_D_6_ = 7.16 ppm (^1^H NMR); 128.06 ppm (^13^C NMR). Powder X‐ray diffraction experiments were performed using a PANalytical Xpert Pro diffractometer, using a Cu K*α* radiation source (*λ=*0.154 nm) at 40 mA and 40 kV with a step size of 0.017° 2θ, scan step time of 85 s and scan range of 5–90° 2θ for 1 hour experiments, and a step size of 0.017° 2θ, scan step time of 1051 s and scan range of 5–90° 2θ for 12 hour experiments. Baseline corrections were processed using Fityk Software (version 1.3.1; Marcin Wojdyr, 2010),^[49]^ and line fittings were processed using either Fityk Software^[49]^ or Origin2020. The average crystallite size (*D*) was estimated according to the Scherrer equation, *D=*k*λ*/βcosθ, where β is the full width at half maximum (FWHM) of the diffraction peak after instrumental broadening correction and k is the shape factor for the average crystallite. β is calculated from β2=βo2−b2, where βo is the measured FWHM of the sample following fitting to a Gaussian function, and b is the measured FWHM of a well‐crystallized material (LaB6, 99.5 %, Alfa Aesar) to account for instrument broadening and *k=*0.9 for powders, assuming spherical shape. Air‐sensitive samples were prepared in a glovebox by drop casting a toluene solution of the sample onto a glass slide; samples were then placed into a sealed sample holder. FTIR spectra were obtained on a Shimadzu IRSpirit spectrometer, fitted with a KBr window and DLATGS detector with temperature control. FTIR spectra were recorded inside a glove box using a single reflection ATR accessory and measured in transmission scanning mode. Samples were drop‐cast onto the sample holder from toluene and scanned from 4700–340 cm^−1^ (100 scans, 4 cm^−1^ resolution). UV‐visible spectra in toluene ([Cu]=0.5 mM for Cu@(O_2_CR^n^)_0.1_, Cu@(S_2_CR^1^)_0.1_ and Cu_2_O@(O_2_CR^n^)_0.1_; [Cu]=4.2 mM for Cu_2_O@(O_2_CR^2^)_0.1_ when synthesized using H_2_O; [Cu]=4 mM for Cu_2_O@(O_2_CR^2^)_0.2_; [Cu]=0.3 mM for Cu_2_S@(S_2_CR^1^)_0.1_) were collected using a Cary 60 UV‐Vis spectrophotometer (Agilent Technologies). UV‐Vis‐NIR spectra in chloroform ([Cu]=4.5 mM) for Cu_2_S@(S_2_CR^1^)_0.2_ were collected using a PerkinElmer Lambda 1050+ UV/VIS/NIR Spectrometer equipped with a 150 mm InGaAs Integrating Sphere detector. Thermal gravimetric analysis thermograms for Cu@(O_2_CR^n^)_0.1_ (n=1, 2) and Cu@(S_2_CR^1^)_0.1_ were collected on a TGA5500 System (TA Instruments), equipped with the TRIOS software package. Samples were analyzed under a N_2_ atmosphere, in sealed 80 μL aluminum pans (TA Instruments); they were heated to 100 °C, at a rate of 5 °C per minute and held for 15 min, then heated to 600 °C, at a rate of 5 °C per minute. TGA thermograms for Cu_2_O@(O_2_CR^n^)_x_ (n=1, x=0.1; n=2, x=0.1 or 0.2) and Cu_2_S@(S_2_CR^1^)_x_ (x=0.1 or 0.2) were collected on a Mettler Toledo TGA/DSC 1 STAR^e^ System; samples of Cu_2_O were analyzed under air, and samples of Cu_2_S were analyzed under a N_2_ atmosphere. Samples were heated to 100 °C at a rate of 10 °C per minute and held for 10 min, then heated to 800 °C at a rate of 10 °C per minute. All TGA curves were normalized to 100 % following any residual solvent loss. X‐ray photoelectron spectroscopy (XPS) was used to characterize the surface of the nanoparticles. The spectra were recorded on a Thermo Scientific K‐Alpha X‐ray photoelectron spectrometer system operating at 1×10^−8^ mbar base pressure. This system incorporates a monochromated, microfocused Al Kα X‐ray source (hν=1486.6 eV) and a 180° double focusing hemispherical analyser with a 2D detector. An X‐ray spot size of 400 μm was used and the X‐ray source was operated at 6 mA emission current and 12 kV anode bias. A flood gun was used to minimize sample charging. Samples were mounted using conductive carbon tape and transferred to the spectrometer using a special glove box module which ensured that samples were never exposed to air. Data were collected at 200 eV pass energy for survey, 20 eV pass energy for core level and 15 eV pass energy for valence spectra. All data were analyzed using the Avantage software package and performed by Dr Anna Regoutz (University College London). Transmission electron microscope (TEM) samples were prepared by drop‐casting diluted colloidal solutions (toluene, 36 mM) onto ultrathin (∼3 nm) carbon films on lacy carbon support film, 300 mesh, gold TEM grids (Agar scientific) while in a glove box. TEM images were acquired on a Cs aberration corrected Titan 80/300 TEM/STEM microscope operated at 300 kV and equipped with a Bruker XFlash EDS detector and Gatan Tridiem Giff. A JEOL‐3000F field emission TEM was operated at 200 kV to perform selected area electron diffraction from specimens of Cu_2_O@(O_2_CR^2^)_0.1_ and Cu_2_S@(S_2_CR^1^)_0.1_ (synthesized from H_2_S). Specimens were drop cast onto holey carbon coated copper TEM grids (Agar Scientific). Image and diffraction data was collected via a Gatan US‐4000 bottom mount imaging camera.

### Syntheses

**Cu@(E_2_CR^n^)_0.1_ (E=O, n=1 or 2; E=S, n=1)**: A solution of the appropriate acid, HE_2_CR^n^ (HO_2_CR^1^: 28.5 mg, 0.18 mmol; HO_2_CR^2^: 32.1 mg, 0.18 mmol; HS_2_CR^1^: 34.3 mg, 0.18 mmol), in toluene (10 mL) was added to a solution of [CuMes]_z_ (329 mg, 1.8 mmol) in toluene (40 mL) in a 200 mL ampoule equipped with a Young's tap. The overall volume of toluene used was 50 mL to ensure a [Cu] concentration of 36 mM. The solution was degassed by three freeze‐pump‐thaw cycles and H_2_ (1 bar) was introduced while the solution was fully submerged in liquid N_2_. The sealed reaction mixture was then allowed to warm to room temperature, before being heated for 2.5 h, in a pre‐heated oil bath, at 110 °C. The solution was then removed from the oil bath, allowed to cool to room temperature and stirred overnight (∼16 h). Excess H_2_ was removed by briefly exposing the reaction mixture to dynamic vacuum followed by N_2_ (x 3). For Cu@(E_2_CR^1^)_0.1_, the resulting deep red and black colloidal solutions for E=O and S, respectively, were evaporated to dryness under reduced pressure. The resulting oily residue was re‐dissolved in toluene (∼5 mL) and the Cu@(E_2_CR^1^)_0.1_ nanoparticles were isolated by precipitation with acetone (∼15 mL) and centrifugation (3900 rpm). The dark red and black solids, for E=O and S, respectively, were re‐dissolved in toluene (∼3 mL), transferred into pre‐weighed vials, and evaporated to dryness under reduced pressure, resulting in isolated yields=96 mg (67 %, Cu@(O_2_CR^1^)_0.1_); 108 mg (73 %, Cu@(S_2_CR^1^)_0.1_). For Cu@(O_2_CR^2^)_0.1_, product isolation using equivalent precipitation was not feasible due to its high solubility in common organic solvents, rather the solution was stored in the glove box freezer at −30 °C. PXRD: 2θ [°]=44, 51 and 74 (Cubic Cu^0^; JCPDS 01‐085‐1326), estimated particle size of 3.9 nm for Cu@(O_2_CR^1^)_0.1_, 3.1 nm for Cu@(O_2_CR^2^)_0.1_ and 3.0 nm for Cu@(S_2_CR^1^)_0.1_ nm (Scherrer equation). FTIR Cu@(O_2_CR^1^)_0.1_: ν=2958 (m, C−H), 2924 (s, C−H), 2872 (w, C−H), 2852 (s, C−H), 1663 (w), 1576 (w), 1525 (w), 1421 (w), 1410 (s, asym. (RCO_2_)), 1311 (w), 1244 (w), 1206 (w), 1118 (w), 780 (w), 713 cm^−1^ (w). Cu@(O_2_CR^2^)_0.1_: ν=2976 (w, C−H), 2922 (s, C−H), 2875 (s, C−H), 2818 (w, C−H), 1628 (w), 1552 (w), 1427, 1408 (s, asym. (RCO_2_)), 1370 (w), 1325 (s, sym. (RCO_2_)), 1246 (w), 1120 (w), 1111 (s), 1029 (w), 932 (w), 888 (w), 851 (w), 721 cm^−1^ (m). Cu@(S_2_CR^1^)_0.1_: ν=3015 (w, C−H), 2957 (s, C−H), 2922 (s, C−H), 2854 (s, C−H), 1610 (w), 1602 (w), 1550 (w), 1461 (s), 1402 (w), 1377 (m), 1296 (w), 1260 (w), 1176 (m), 1102 (w), 1044, 1011 (s, asym. (RCS_2_)), 954 (m), 877 (w), 848 (s, sym. (RCS2)), 805 (w), 723 (m), 706 (m), 617 (m), 594 (w), 571 (m), 549 cm^−1^ (m).

**Oxidation to Cu_2_O@(O_2_CR^n^)_0.1_ (n=1 or 2)**: A 36 mM solution of Cu@(O_2_CR^n^)_0.1_ (n=1 or 2) in toluene (50 mL) in a 200 mL ampoule, equipped with a Young's tap, was exposed to air, at room temperature for ∼4 h, before sealing the tap and stirring overnight (∼16 h). The original deep red colloidal solution turned dark green but remained colloidal. Cu_2_O@(O_2_CR^1^)_0.1_ was concentrated (∼5 mL) and isolated by precipitation from toluene with acetone (∼15 mL) followed by centrifugation (3900 rpm). The remaining dark green solid was re‐dissolved in toluene (∼3 mL), transferred into a pre‐weighed vial and evaporated to dryness under reduced pressure, affording a dark green solid. Isolated yield=75 mg (58 %). For Cu_2_O@(O_2_CR^2^)_0.1_, the precipitation was not feasible due to its high solubility in common organic solvents, instead the solution was stored in the glove box freezer at −30 °C. PXRD: 2θ [°]=30, 37, 42, 53, 62 and 74 (Cubic Cu_2_O; JCPDS 00‐002‐1067); estimated particle size of 2.4 nm for Cu_2_O@(O_2_CR^1^)_0.1_ and 3.1 nm for Cu_2_O@(O_2_CR^2^)_0.1_ (Scherrer equation). FTIR Cu_2_O@(O_2_CR^1^)_0.1_: ν=3154 (br, surface O−H), 2956 (m, C−H), 2924 (s, C−H), 2872 (w, C−H), 2854 (m, C−H), 1547 (s, asym. (RCO_2_)), 1430 (s, sym. (RCO_2_)), 645 cm^−1^ (m). Cu_2_O@(O_2_CR^2^)_0.1_: ν=3425 (br, surface O−H), 2953 (w, C−H), 2918 (s, C−H), 2871 (w, C−H), 2852 (s, C−H), 1588 (s, asym. (RCO_2_)), 1415 (s, sym. (RCO_2_)), 1308 (m), 1260 (w), 1199 (m), 1105 (s), 930 (w), 888 (w), 851 (w), 801 (w), 720 cm^−1^ (m).

**Cu_2_O@(O_2_CR^2^)_0.1_**: **Method B –** A solution of HO_2_CR^2^ (9.8 mg, 5.5×10^−2^ mmol) in toluene (3.0 mL) was added to [CuMes]_z_ (100 mg, 0.55 mmol) in toluene (12.2 mL), at room temperature, in a 50 mL ampoule equipped with a Young's tap. The overall volume of toluene used (15.2 mL) was selected to ensure an overall [Cu]=36 mM. Next, a solution of H_2_O in THF (2.8 M, 0.10 mL, 0.27 mmol) was added to the copper solution, at room temperature and under a nitrogen atmosphere. Following the addition, the reaction mixture was exposed to air and stirred overnight (∼16 h), at room temperature. The resulting green solution, after evaporation to dryness, was analyzed by PXRD, FTIR and UV‐Vis spectroscopy, which were identical to those data for Cu_2_O@(O_2_CR^2^)_0.1_ synthesized by oxidation of Cu@(O_2_CR^2^)_0.1_ in air.

**Cu_2_O@(O_2_CR^2^)_0.2_**: A solution of HO_2_CR^2^ (39.0 mg, 0.219 mmol) in toluene (5 mL) was added to a solution of [CuMes]_z_ (200 mg, 1.10 mmol), in toluene (25.4 mL) in an ampoule equipped with a Young's tap. The overall volume of toluene used was 30.4 mL to ensure a [Cu] concentration of 36 mM. The solution was degassed, by three freeze‐pump‐thaw cycles, and H_2_ (1 bar) was introduced, while the solution was fully submerged in liquid N_2_. The sealed reaction mixture was then allowed to warm to room temperature, before being heated for 2.5 h, in a pre‐heated oil bath, at 110 °C. The solution was then removed from the oil bath, allowed to cool to room temperature and stirred overnight (∼16 h). Excess H_2_ was removed by briefly exposing the reaction mixture to dynamic vacuum, followed by N_2_ (x 3), ensuring not to remove any solvent. The Young's tap was then opened to air and the deep red colloidal solution turned dark green but remained colloidal. The Young's tap was left open for ∼4 h before being closed; the reaction solution was stirred overnight at room temperature. Further product purification by precipitation was not feasible due to its high solubility in common organic solvents. Therefore, the resulting dark green colloidal solution was stored in the glove box freezer at −30 °C. PXRD: 2θ [°]=30, 37, 52, 62, 74 (cubic Cu_2_O; JCPDS 00‐002‐1067); estimated particle size of 3.1 nm. FTIR: ν=3366 (br, surface O−H), 2922 (s, C−H), 2874 (s, C−H), 2822 (s, C−H), 1584 (s, asym. (RCO_2_)), 1438, 1415 (m, sym. (RCO_2_)), 1328 (m), 1247 (w), 1199 (w), 1110 (s), 1027 (w), 930 (w), 851 (w), 726 (w), 636 cm^−1^ (m).

**Cu_2_S@(S_2_CR^1^)_0.1_**: **Method A –** Di(thio)nonanoic acid (HS_2_CR^1^; 41.6 mg, 0.219 mmol) in toluene (10 mL) was added to [CuMes]_z_ (400 mg, 2.19 mmol) in toluene (50.8 mL), at room temperature, in a 200 mL ampoule equipped with a Young's tap. The overall volume of toluene used was 60.8 mL to ensure a [Cu] concentration of 36 mM. H_2_S in d_8_‐toluene (0.60 M, 1.83 mL, 1.10 mmol) was added to the resulting scarlet solution at room temperature, and the reaction mixture was stirred overnight (∼16 h), at 100 °C. The resulting mahogany solution was cooled to room temperature and evaporated to dryness under reduced pressure. The resulting dark brown/black oily residue was re‐dissolved in toluene (∼5 mL) and the desired Cu_2_S@(S_2_CR^1^)_0.1_ nanoparticles were precipitated via the addition of acetone (∼20 mL) and isolated by centrifugation. The orange mother liquors were then decanted from the centrifuge tubes and the remaining pale‐yellow solid was re‐dissolved in toluene (∼3 mL), transferred into a pre‐weighed vial and evaporated to dryness under reduced pressure, affording a black, crystalline solid. Isolated yield=140 mg (72 %). PXRD: 2θ [°]=28, 34, 37, 47 and 55 (Hexagonal chalcocite; JCPDS 01‐084‐0208); estimated particle size of 2.3 nm. FTIR: ν=2955 (s, C−H), 2921 (s, C−H), 2872 (w, C−H), 2852 (s, C−H), 1703 (m), 1609 (m), 1460 (s), 1375 (m), 1359 (m), 1295 (w), 1253 (w), 1213 (w), 1195 (w), 1176 (w), 1122 (m), 1044, 1027, 1011 (m, asym. (RCS_2_)), 954 (w), 921 (w), 848 (s, sym. (RCS_2_)), 721 (w), 618 cm^−1^ (s).

**Cu_2_S@(S_2_CR^1^)_0.1_**: **Method B –** Di(thio)nonanoic acid (HS_2_CR^1^; 10.4 mg, 5.47×10^−2^ mmol) in toluene (3 mL) was added to [CuMes]_z_ (100 mg, 0.547 mmol) in toluene (12.2 mL), at room temperature, in a 100 mL ampoule equipped with a Young's tap. The overall volume of toluene used was 15.2 mL to ensure a [Cu] concentration of 36 mM. S(SiMe_3_)_2_ (57.7 μL, 0.274 mmol) was added to the resulting scarlet solution at room temperature, and the reaction mixture was stirred overnight (∼16 h) at 100 °C. The resulting mahogany solution was cooled to room temperature and evaporated to dryness under reduced pressure. The resulting dark brown/black oily residue was re‐dissolved in toluene (∼2 mL) and the desired Cu_2_S@(S_2_CR^1^)_0.1_ nanoparticles were precipitated via the addition of acetone (∼10 mL) and isolated by centrifugation. The orange mother liquors were then decanted from the centrifuge tube and the remaining pale‐yellow solid was re‐dissolved in toluene (1 mL), transferred into a pre‐weighed vial and evaporated to dryness under reduced pressure to afford a black, crystalline solid. Isolated yield=29 mg (60 %). The characterization data were identical to the product made using Method A.

**Cu_2_S@(S_2_CR^1^)_0.2_**: Di(thio)nonanoic acid (HS_2_CR^1^; 83.2 mg, 0.438 mmol) in toluene (10 mL) was added to [CuMes]_z_ (400 mg, 2.19 mmol) in toluene (50.8 mL), at room temperature, in a 200 mL ampoule equipped with a Young's tap. The overall volume of toluene used was 60.8 mL to ensure a [Cu] concentration of 36 mM. H_2_S in d_8_‐toluene (1.5 M, 0.7 mL, 1.10 mmol) was added to the resulting scarlet solution at room temperature, and the reaction mixture was stirred overnight (∼16 h) at 100 °C. The resulting mahogany solution was cooled to room temperature and evaporated to dryness under reduced pressure. The resulting dark brown/black oily residue was re‐dissolved in toluene (∼5 mL) and the desired Cu_2_S@(S_2_CR^1^)_0.2_ nanoparticles were precipitated via the addition of acetone (∼20 mL) and isolated by centrifugation. The orange mother liquors were then decanted from the centrifuge tubes and the remaining pale‐yellow solid was re‐dissolved in toluene (∼3 mL), transferred into a pre‐weighed vial and evaporated to dryness under reduced pressure to afford a black, crystalline solid. Isolated yield=154 mg (71 %). PXRD: 2θ [°]=28, 36, 47 and 54 (Hexagonal chalcocite; JCPDS 01‐084‐0208); estimated particle size of 2.4 nm. FTIR: ν=2954 (s, C−H), 2921 (s, C−H), 2871 (w, C−H), 2853 (s, C−H), 1734 (w), 1609 (m), 1460 (s), 1376 (m), 1347 (w), 1176 (w), 1044, 1030, 1012 (m, asym. (RCS_2_)), 953 (w), 846 (s, sym. (RCS_2_)), 722 cm^−1^ (m).

## Supporting Information

Supporting Information is available: experimental methods associated with ligand exchange reactivity, attempted syntheses, a determination of maximum nanoparticle surface coverage and solubility studies; a table for comparing key stretching bands observed by FTIR spectroscopy; a table of important ^13^C NMR and FTIR data for literature diacyl disulfides; and experimental spectra. Additional referenced cited within the Supporting Information.[^50^, ^51^, ^52^, ^53^, ^54^, ^55^, ^56^, ^57^, ^58^, ^59^]

## Conflict of interest

The authors declare no conflict of interest.

1

## Supporting information

As a service to our authors and readers, this journal provides supporting information supplied by the authors. Such materials are peer reviewed and may be re‐organized for online delivery, but are not copy‐edited or typeset. Technical support issues arising from supporting information (other than missing files) should be addressed to the authors.

Supporting Information

## Data Availability

The data that support the findings of this study are available from the corresponding author upon reasonable request.
