# Supplementary material for: Matched Ligands for Small, Stable Colloidal Nanoparticles of Copper, Cuprous Oxide and Cuprous Sulfide
Source: Chemistry. 2023 May 8;29(35):e202300228. doi: 10.1002/chem.202300228 (PMC10947121; doi:10.1002/chem.202300228)
Supplement: Supplementary file 1 — Supporting Information [file CHEM-29-0-s001.pdf]

Chemistry–A European Journal

Supporting Information

# Matched Ligands for Small, Stable Colloidal Nanoparticles of Copper, Cuprous Oxide and Cuprous Sulfide

Bradley E. Cowie, Lisa Häfele, Andreas Phanopoulos, Said A. Said, Ja Kyung Lee, Anna Regoutz, Milo S. P. Shaffer,* and Charlotte K. Williams*

## Table of Contents


**Attempted Syntheses**........ 2

- $Cu_2O@(O_2CR^1)_{0.2}$........ 2
- $Cu_2O@(S_2CR^1)_{0.1}$........ 2
- $Cu_2S@(O_2CR^1)_{0.1}$ at 100 °C........ 2
- $Cu_2S@(O_2CR^2)_{0.1}$ at 100 °C........ 2

**Ligand Exchange Reactivity**........ 2

- $Cu_2O@(O_2CR^2)_{0.2}$ + 0.2 equiv. $HS_2CR^1$ at 20 °C........ 2
- $Cu_2O@(O_2CR^2)_{0.2}$ + 0.4 equiv. $HS_2CR^1$ at 20 °C........ 3
- $Cu_2O@(O_2CR^2)_{0.2}$ + 0.4 equiv. $HS_2CR^1$ at 100 °C........ 3
- $Cu_2S@(S_2CR^1)_{0.2}$ + 0.2 equiv. $HO_2CR^1$ at 20 °C........ 3
- $Cu_2S@(S_2CR^1)_{0.2}$ + 0.4 equiv. $HO_2CR^1$ at 20 °C........ 3
- $Cu_2S@(S_2CR^1)_{0.2}$ + 0.4 equiv. $HO_2CR^1$ at 100 °C........ 3

**Determination of Nanoparticle Surface Coverage**........ 4

**Experimental Spectra and Data for Characterizing Cu, $Cu_2O$ and $Cu_2S$ Nanoparticles**........ 6

**Table S3.** Comparison of key stretching bands observed by FT-IR spectroscopy........ 42

**Solubility Studies:** Example Procedure........ 63

**Experimental Spectra and Data Associated with Ligand Exchange Reactivity for $Cu_2S$**........ 64

**Experimental Spectra and Data Associated with Ligand Exchange Reactivity for $Cu_2O$**........ 70

**Table S4.** Important $^{13}C$ NMR and FT-IR data for literature diacyl disulfides........ 71

## Attempted Syntheses:

**$Cu_2O@(O_2CR^1)_{0.2}$:** A solution of $HO_2CR^1$ (57 mg, 0.36 mmol) in toluene (5 mL) was added to a solution of $[CuMes]_z$ (329 mg, 1.8 mmol) in toluene (45 mL) in an ampoule equipped with a Young's tap. The overall volume of toluene used was 50 mL to ensure a [Cu] concentration of 36 mM. The solution was degassed by three freeze-pump-thaw cycles and $H_2$ (1 bar) was introduced while the solution was fully submerged in liquid $N_2$. The sealed reaction mixture was then allowed to warm to room temperature, before being heated for 2.5 h, in a pre-heated oil bath at 110 °C. The solution was then removed from the oil bath, allowed to cool to room temperature and stirred overnight (~ 16 hours). Excess $H_2$ was removed by quickly exposing the reaction mixture to dynamic vacuum followed by $N_2$ (x 3), ensuring not to remove any solvent. The Young's tap was then opened to air and the deep red colloidal solution turned dark green but remained colloidal. The Young's tap was left open for ~4 hours before being closed; the reaction solution was stirred overnight at room temperature. The resulting dark green solution was concentrated to a volume of ~5 mL, precipitated via the addition of acetone (~20 mL) and isolated by centrifugation. The remaining dark green solid was re-dissolved in toluene (~3 mL), transferred into a pre-weighed vial and evaporated to dryness under reduced pressure, affording a dark green solid. PXRD: 2θ [°] = 8, 11, 15, 22 (unidentified impurity), 30, 37, 52, 62, 74 (cubic $Cu_2O$; JCPDS 00-002-1067).

**$Cu_2O@(S_2CR^1)_{0.1}$:** $Cu@(S_2CR^1)_{0.1}$ was prepared as described above, and a 36 mM solution in toluene (22.8 mL) in an ampoule equipped with a Young's tap was exposed to air at room temperature. Soon after exposure, a black solid precipitated from the solution with the formation of a yellow supernatant. The tap was sealed after ~4 hours of exposure and the suspension stirred overnight (~16 hours). The supernatant was decanted, and the precipitate was washed with toluene (~15 mL) and isolated by centrifugation. The black solid was dried under reduced pressure and analyzed by PXRD and FT-IR spectroscopy. PXRD: 2θ [°] = 29, 36, 42, 49, 61, 73 and 75 (Cubic $Cu_2O$; JCPDS 00-002-1067). No stretches representing surface-coordinated ligand were observed by FT-IR spectroscopy.

**$Cu_2S@(O_2CR^1)_{0.1}$ at 100 °C:** Nonanoic acid ($HO_2CR^1$; 8.7 mg, $5.47\times10^{-2}$ mmol) in toluene (5 mL) was added to $[CuMes]_z$ (100 mg, 0.547 mmol) in toluene (10.2 mL) at room temperature in a 50 mL ampoule equipped with a Young's tap; the overall volume of toluene used was 15.2 mL to ensure a [Cu] concentration of 36 mM. $H_2S$ in $d_8$-toluene (0.64 M, 0.43 mL, 0.275 mmol) was added to the clear, slightly yellow solution at room temperature, turning it red/brown; the reaction mixture was stirred overnight (~16 hours) at 100 °C. The resulting black/brown solution was cooled to room temperature and evaporated to dryness under reduced pressure. Toluene (~3 mL) was added to the crude residue, which was drop cast onto a glass slide for powder X-ray diffraction. PXRD: 2θ [°] = 7, 8, 9, 9.8, 10.4, 11, 12, 36, 43 (cubic $Cu^0$), 51 (cubic $Cu^0$) and 74 (cubic $Cu^0$; JCPDS 01-085-1326).

**$Cu_2S@(O_2CR^2)_{0.1}$ at 100 °C:** 2-[2-(2-methoxyethoxy)ethoxy]acetic acid ($HO_2CR^2$; 9.8 mg, $5.47\times10^{-2}$ mmol) in toluene (5 mL) was added to $[CuMes]_z$ (100 mg, 0.547 mmol) in toluene (10.2 mL) at room temperature in a 50 mL ampoule equipped with a Young's tap; the overall volume of toluene used was 15.2 mL to ensure a [Cu] concentration of 36 mM. $H_2S$ in $d_8$-toluene (1.5 M, 0.18 mL, 0.275 mmol) was added to the clear, slightly yellow solution at room temperature, turning it chestnut brown; the reaction mixture was stirred overnight (~16 hours) at 100 °C. The resulting translucent brown solution was cooled to room temperature and evaporated to dryness under reduced pressure. The resulting material was analyzed by PXRD and FT-IR spectroscopy. PXRD: 2θ [°] = 33, 44 (cubic $Cu^0$), 46, 49, 51 (cubic $Cu^0$), 54 and 74 (cubic $Cu^0$; JCPDS 01-085-1326).

## Ligand Exchange Reactivity:

**$Cu_2O@(O_2CR^2)_{0.2}$ + 0.2 equiv. $HS_2CR^1$ at 20 °C:** A solution of $HS_2CR^1$ (20.8 mg, 0.109 mmol) in toluene (0.5 mL) was added, dropwise, to a solution of $Cu_2O@(O_2CR^2)_{0.2}$ (48.9 mg, 0.274 mmol), in toluene (14.7 mL; 36 mM solution in [Cu]), at room temperature. The reaction was stirred for 2 hours during which time it changed colour from dark green to brown/orange. An FT-IR spectrum of the crude mixture was then obtained, and the solution was evaporated to dryness under reduced pressure. The crude mixture of $HS_2CR^1$ and $Cu_2O@(O_2CR^2)_{0.2}$ was analyzed by PXRD, TGA and IR spectroscopy. PXRD: 2θ [°] = 29, 37, 43, 52, 62, 74 (cubic $Cu_2O$; JCPDS 00-002-1067); estimated particle size of 3.5 nm. FT-IR: ν = 2920 (s, C–H), 2871 (s, C–H), 2855 (s, C–H), 1758 (w), 1636 (s, $R^1C(O)S_2C(O)R^1$ ν(C=O)), 1575 (s, asym. ($RCO_2$)), 1440, 1415 (s, sym. ($RCO_2$)), 1328 (s), 1296 (w), 1246 (w), 1198 (m), 1104 (s), 1029 (w), 953 (w), 931 (w), 885 (w), 851 (m), 723 (m), 606 $cm^{-1}$ (s).

**$Cu_2O@(O_2CR^2)_{0.2}$ + 0.4 equiv. $HS_2CR^1$ at 20 °C:** A solution of $HS_2CR^1$ (41.6 mg, 0.219 mmol) in toluene (0.5 mL) was added, dropwise, to a 36 mM solution of $Cu_2O@(O_2CR^2)_{0.2}$ in toluene (15.2 mL), at room temperature and stirred for 2 hours. The solution turned from dark green to brown/orange in colour. The solution was then evaporated to dryness under reduced pressure. The crude mixture of $HS_2CR^1$ and $Cu_2O@(O_2CR^2)_{0.2}$ was analyzed by PXRD and FT-IR spectroscopy. PXRD: 2θ [°] = 37, 43, 62 (cubic $Cu_2O$; JCPDS 00-002-1067). FT-IR: ν = 3190 (br, $HO_2CR^2$ ν(O–H)), 2955 (m, C–H), 2922 (s, C–H), 2872 (m, C–H), 2853 (s, C–H), 1760 (s, $HO_2CR^2$ ν(C=O)), 1641 (s, $R^1C(O)S_2C(O)R^1$ ν(C=O)), 1586 (s, asym. ($RCO_2$)), 1457 (s, sym. ($RCO_2$)), 1378 (w), 1354 (w), 1330 (m), 1248 (w), 1199 (m), 1107 (s), 1027 (w), 962 (w), 932 (w), 851 (m), 722 $cm^{-1}$ (m).

**$Cu_2O@(O_2CR^2)_{0.2}$ + 0.4 equiv. $HS_2CR^1$ at 100 °C:** A solution of $HS_2CR^1$ (41.6 mg, 0.219 mmol), in toluene (0.5 mL), was added, dropwise, to a 36 mM solution of $Cu_2O@(O_2CR^2)_{0.2}$ in toluene (15.2 mL), at room temperature, and the solution turned from dark green to brown/orange in colour. The reaction was stirred for 2 hours at 100 °C. The solution was then evaporated to dryness under reduced pressure. The crude mixture of $HS_2CR^1$ and $Cu_2O@(O_2CR^2)_{0.2}$ was analyzed by PXRD and FT-IR spectroscopy. PXRD: 2θ [°] = 28, 36, 42, 47, 61, 74 (cubic $Cu_2O$; JCPDS 00-002-1067). FT-IR: ν = 3155 (br, $HO_2CR^2$ ν(O–H)), 2953 (m, C–H), 2920 (s, C–H), 2871 (m, C–H), 2853 (s, C–H), 1762, 1742 (s, $HO_2CR^2$ ν(C=O)), 1494 (w), 1457 (s), 1376 (w), 1353 (m), 1338 (w), 1278 (w), 1248 (m), 1200 (m), 1145 (s), 1102 (s), 962 (w), 932 (w), 851 (m), 722 $cm^{-1}$ (w).

**$Cu_2S@(S_2CR^1)_{0.2}$ + 0.2 equiv. $HO_2CR^1$ at 20 °C:** A solution of $HO_2CR^1$ (36.8 mg, 0.233 mmol) in toluene (1 mL) was added, dropwise, to a 36 mM solution of $Cu_2S@(S_2CR^1)_{0.2}$ (115 mg, 0.582 mmol), in toluene (31.3 mL) at room temperature, and stirred for 2 hours. The solution did not undergo a colour change. An FT-IR spectrum of the crude mixture was then obtained, and the solution was concentrated to ~2 mL under reduced pressure. The crude mixture of unreacted $HO_2CR^1$ and $Cu_2S@(S_2CR^1)_{0.2}$ was precipitated from toluene, with acetone (~8 mL), and isolated by centrifugation. The mother liquors were discarded, and the resulting pale-yellow solid was re-dissolved in toluene (~3 mL), transferred into a pre-weighed vial and evaporated to dryness under reduced pressure to afford a black, crystalline solid. This material was further analyzed by PXRD and TGA. PXRD: 2θ [°] = 28, 37, 47 and 54 (Hexagonal chalcocite; JCPDS 01-084-0208); estimated particle size of 2.6 nm. FT-IR: ν = 2956 (s, C–H), 2923 (s, C–H), 2872 (w, C–H), 2853 (s, C–H), 1713 (w, $HO_2CR^1$ ν(C=O)), 1610 (m), 1461 (s), 1376 (m), 1346 (w), 1177 (w), 1044, 1030, 1012 (m, asym. ($RCS_2$)), 955 (w), 846 (s, sym. ($RCS_2$)), 721 $cm^{-1}$ (m).

**$Cu_2S@(S_2CR^1)_{0.2}$ + 0.4 equiv. $HO_2CR^1$ at 20 °C:** A solution of $HO_2CR^1$ (22.8 mg, 0.144 mmol), in toluene (0.5 mL), was added, dropwise, to a 36 mM solution of $Cu_2S@(S_2CR^1)_{0.2}$ in toluene (10.0 mL), at room temperature, and stirred for 2 hours. An FT-IR spectrum of the crude mixture was then obtained, and the solution was concentrated to ~2 mL under reduced pressure. The crude mixture of unreacted $HO_2CR^1$ and $Cu_2S@(S_2CR^1)_{0.2}$ was precipitated from toluene with acetone (~8 mL) and isolated by centrifugation. The mother liquors were analyzed by $^1H$ NMR spectroscopy which revealed unreacted $HO_2CR^1$. The resulting pale- yellow solid from centrifugation was re-dissolved in toluene (~3 mL), transferred into a pre-weighed vial and evaporated to dryness under reduced pressure to afford a black, crystalline solid. This material was further analyzed by PXRD and TGA. PXRD: 2θ [°] = 35, 46, 54, 62 (Hexagonal chalcocite; JCPDS 01-084-0208). FT-IR: ν = 2956 (s, C–H), 2922 (s, C–H), 2872 (m, C–H), 2852 (s, C–H), 1713 (w, $HO_2CR^1$ ν(C=O)), 1608 (m), 1557 (w), 1460 (s), 1377 (m), 1344 (w), 1296 (w), 1259 (w), 1174 (w), 1104 (m), 1044, 1028 (m, asym. ($RCS_2$)), 954 (w), 846 (s, sym. ($RCS_2$)), 721 $cm^{-1}$ (m).

**$Cu_2S@(S_2CR^1)_{0.2}$ + 0.4 equiv. $HO_2CR^1$ at 100 °C:** A solution of $HO_2CR^1$ (29.3 mg, 0.185 mmol) in toluene (0.5 mL) was added, dropwise, to a solution of $Cu_2S@(S_2CR^1)_{0.2}$ (45.6 mg, 0.231 mmol), in toluene (12.9 mL; 36 mM solution in [Cu]), at room temperature. The reaction mixture was then stirred for 2 hours at 100 °C; it did not undergo a colour change. An FT-IR spectrum of the crude mixture was obtained, and the solution was concentrated to ~2 mL under reduced pressure. The crude mixture of unreacted $HO_2CR^1$ and $Cu_2S@(S_2CR^1)_{0.2}$ was precipitated from toluene with acetone (~8 mL) and isolated by centrifugation. The mother liquors were analyzed by $^1H$ NMR spectroscopy which showed unreacted $HO_2CR^1$. The resulting pale-yellow solid from centrifugation was re-dissolved in toluene (~3 mL), transferred into a pre-weighed vial and evaporated to dryness under reduced pressure to afford a black, crystalline solid. FT-IR: ν = 2955 (s, C–H), 2923 (s, C–H), 2871 (m, C–H), 2853 (s, C–H), 1713 (w, $HO_2CR^1$ ν(C=O)), 1610 (m), 1557 (w), 1461 (s), 1376 (m), 1347 (w), 1294 (w), 1178 (w), 1043, 1028, 1012 (m, asym. ($CS_2$)), 954 (w), 845 (s, sym. ($CS_2$)), 724 $cm^{-1}$ (m).

## Determination of Nanoparticle Surface Coverage

The method for determining the theoretical nanoparticle surface coverage by ligand coordination has been adapted from the literature.[50]

The theoretical total surface area of a nanoparticle sample, $S_T$, is:

$$S_T = \frac{3nV_m}{r}$$

where $n$ is the number of moles of $Cu_2O$ or $Cu_2S$, $V_m$ is the molar volume of $Cu_2O$ and $Cu_2S$, and r is the particle radius, in Å, as an average from powder XRD and TEM measurements; see Tables S1 and S2 for the appropriate values.

Next, the number of moles of ligand per mole of $Cu_2O$ or $Cu_2S$, $\eta_C$, for each sample was calculated from the organic content measured by TGA and determined using the following equation:

$$\eta_C = \frac{(\frac{W_O}{M_O})}{(\frac{W_i}{M_i})}$$

where $W_O$ and $W_i$ are the weight percent (wt%) of organic and inorganic components, respectively, and $M_O$ and $M_i$ are the molecular weight of the ligand and that of either $Cu_2O$ or $Cu_2S$, respectively. Note that because the TGA data for $Cu_2O$ show both ligand and oxide loss, the values used in these calculations were based only on ligand loading. See Tables S1 and S2 for the appropriate values.

Assuming that all ligands are bound to the surface of the nanoparticle and form a close packed monolayer, the theoretical surface area occupied by ligand coordination, $S_S$, was then calculated using the following equation:

$$S_S = Z \times \eta_C \times Surface\ Area\ per\ Ligand$$

where $Z$ is Avogadro's number. The carboxylate ligands, $[O_2CR^n]^-$ (n = 1, 2), were estimated to cover an area of 20.5 Å$^2$.[51] The surface area per di(thio)carboxylate ligand was estimated as 37.9 Å$^2$ using a similar method to Tasker and co-workers[52] based on the calculated head group surface area of a di(thio)carboxylate using literature C–S bond lengths (average = 1.685 Å for $[Cu\{S_2C(Cy)\}(PPh_3)_2]$; Cy = cyclohexyl),[41] the anticipated bond angle for an $sp^2$-hybridized C-atom in a di(thio)carboxylate (i.e. 120 °), and assuming a hexagonal close-packing arrangement on the surface of the nanoparticle (Table S2).

Finally, the ratio of $S_S$ to $S_T$ provides the percentage surface coverage (Table S2).

**Table S1.** Summarized values from theoretical nanoparticle surface coverage calculations.

| Material | *n* | $V_m$ [Å$^3$ mol$^{-1}$] | Particle Size, r, by PXRD [Å] | Particle Size, r, by TEM [Å] | $W_O$ [%] | $M_O$ [g mol$^{-1}$] |
|---|---|---|---|---|---|---|
| $Cu_2O@(O_2CR^1)_{0.1}$ | 3 | $2.385 \times 10^{25}$ | 24.0 | 56.4 | 11.0 | 157.2 |
| $Cu_2O@(O_2CR^2)_{0.1}$ | 3 | $2.385 \times 10^{25}$ | 31.0 | 86.6 | 12.4 | 177.2 |
| $Cu_2O@(O_2CR^2)_{0.2}$ | 3 | $2.385 \times 10^{25}$ | 31.0 | 91.9 | 24.8 | 177.2 |
| $Cu_2S@(S_2CR^1)_{0.1}$ ($H_2S$) | 3 | $2.842 \times 10^{25}$ | 23.0 | 41.7 | 13.0 | 189.4 |
| $Cu_2S@(S_2CR^1)_{0.1}$ ($S(SiMe_3)_2$) | 3 | $2.842 \times 10^{25}$ | 39.0 | 49.9 | 14.0 | 189.4 |

**Table S2.** Summarized values from theoretical nanoparticle surface coverage calculations.

| Material | $W_i$ [%] | $M_i$ [g mol$^{-1}$] | Surface Area per Ligand [Å$^2$] | Theoretical Particle Surface Area [$S_T$, Å$^2$ mol$^{-1}$] | Moles of Ligand per Mole of Particle [$\eta_C$, mol] | Theoretical Surface Area Occupied by Ligand [$S_S$, Å$^2$] | Surface Coverage [%] |
|---|---|---|---|---|---|---|---|
| $Cu_2O@(O_2CR^1)_{0.1}$ | 89.0 | 143.1 | 20.5 | $5.339 \times 10^{24}$ | 0.1125 | $1.389 \times 10^{24}$ | 26 |
| $Cu_2O@(O_2CR^2)_{0.1}$ | 87.6 | 143.1 | 20.5 | $3.650 \times 10^{24}$ | 0.1143 | $1.411 \times 10^{24}$ | 39 |
| $Cu_2O@(O_2CR^2)_{0.2}$ | 75.2 | 143.1 | 20.5 | $3.493 \times 10^{24}$ | 0.2658 | $3.281 \times 10^{24}$ | 94 |
| $Cu_2S@(S_2CR^1)_{0.1}$ ($H_2S$) | 87.0 | 159.2 | 37.9 | $7.895 \times 10^{24}$ | 0.1256 | $2.866 \times 10^{24}$ | 36 |
| $Cu_2S@(S_2CR^1)_{0.1}$ ($S(SiMe_3)_2$) | 86.0 | 159.2 | 37.9 | $6.923 \times 10^{24}$ | 0.1368 | $3.123 \times 10^{24}$ | 45 |

**Note:** Theoretical calculations were not completed for $Cu_2S@(S_2CR^1)_{0.2}$ because a TEM analysis, providing an accurate size determination, was not performed.

## Experimental Spectra and Data for Characterizing Cu, $Cu_2O$ and $Cu_2S$ Nanoparticles:

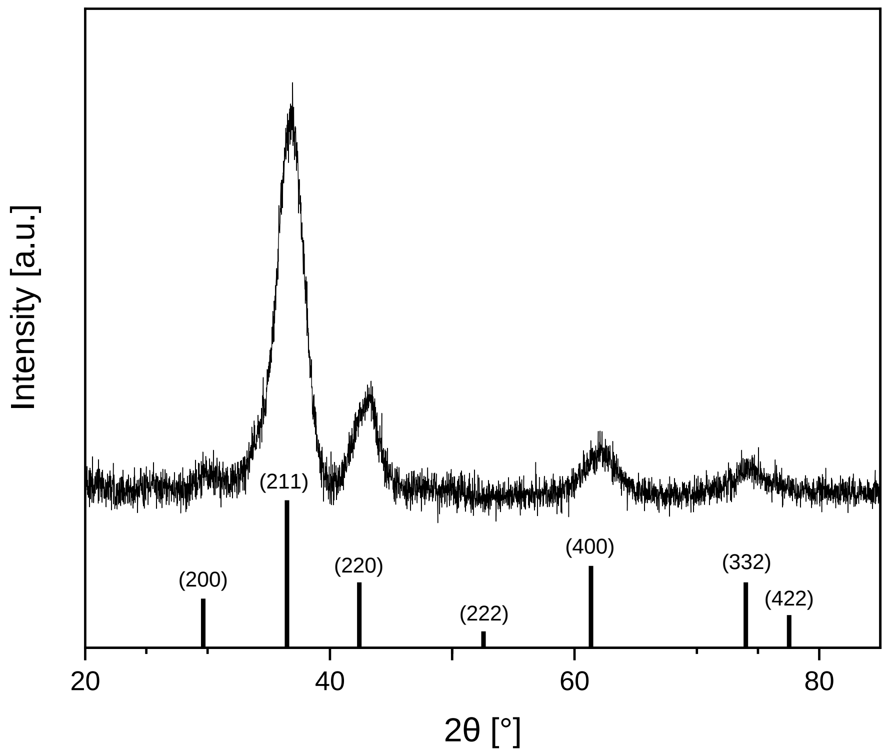


**Figure S1.** Powder X-ray diffraction pattern of crude $Cu_2O@(O_2CR^1)_{0.1}$, which appears the same as the powder X-ray diffraction pattern of isolated $Cu_2O@(O_2CR^1)_{0.1}$ (see Figure S20). Pattern indexed against cubic $Cu_2O$ as vertical bars (JCPDS 00-002-1067). Average crystallite size = 3.4 nm (Scherrer analysis).

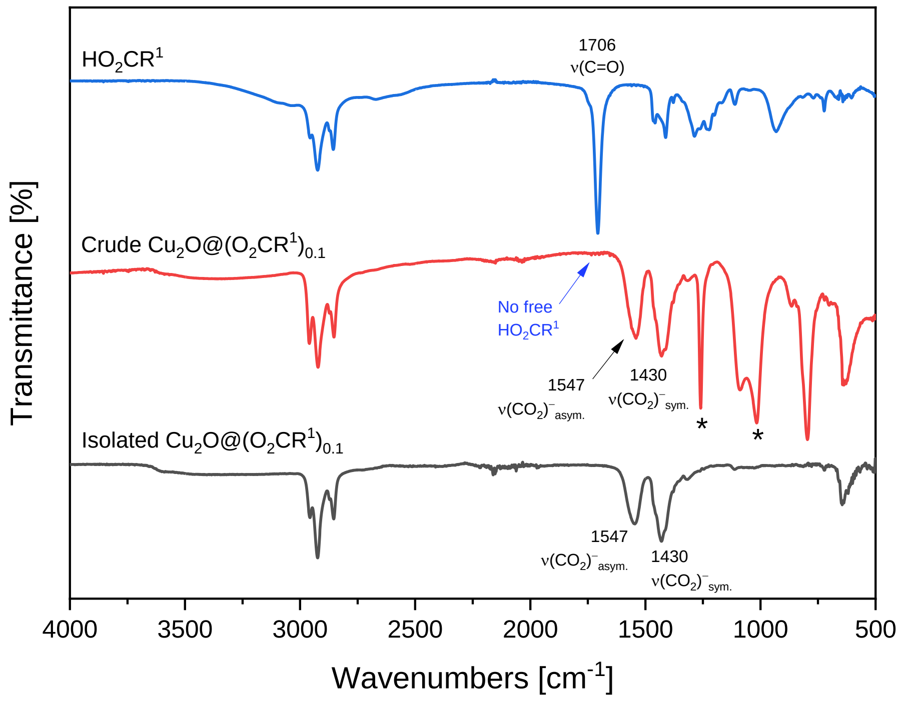


**Figure S2.** Stacked FT-IR spectra of $HO_2CR^1$ (top, blue), crude $Cu_2O@(O_2CR^2)_{0.1}$ (middle, red) and isolated $Cu_2O@(O_2CR^2)_{0.1}$ by precipitation from toluene using acetone (bottom, dark grey). Unreacted carboxylic acid is not observed in the IR spectrum of crude $Cu_2O@(O_2CR^2)_{0.1}$, indicating excess ligand is not washed away. * = mesitylene.

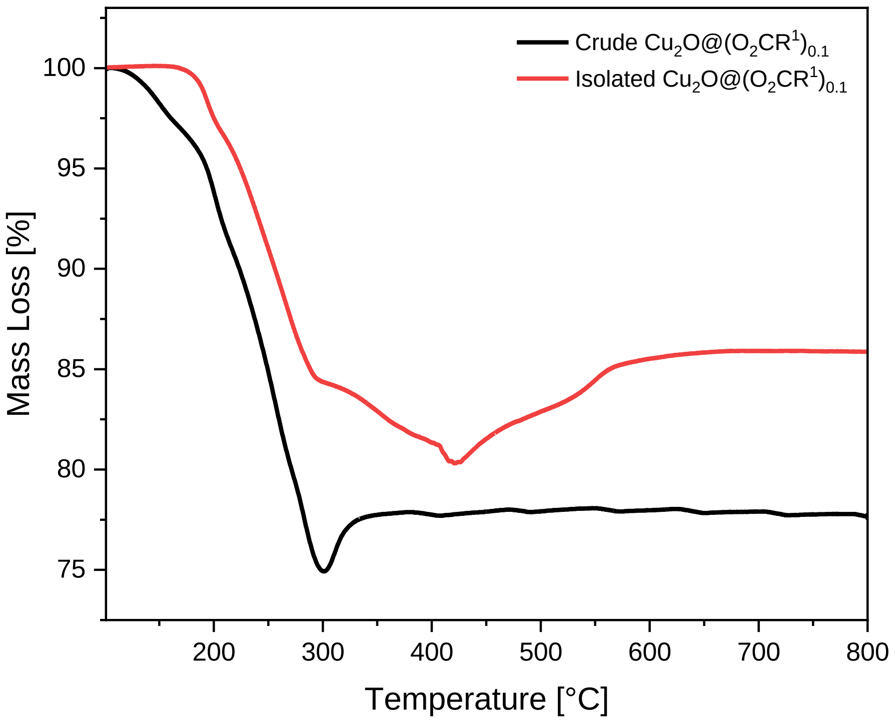


**Figure S3.** Stacked TGA thermograms of crude $Cu_2O(O_2CR^1)_{0.1}$ (black) and isolated $Cu_2O(O_2CR^1)_{0.1}$ (red) by precipitation from toluene using acetone. The anticipated mass loss for complete loss of oxide and ligand is 22 wt%.

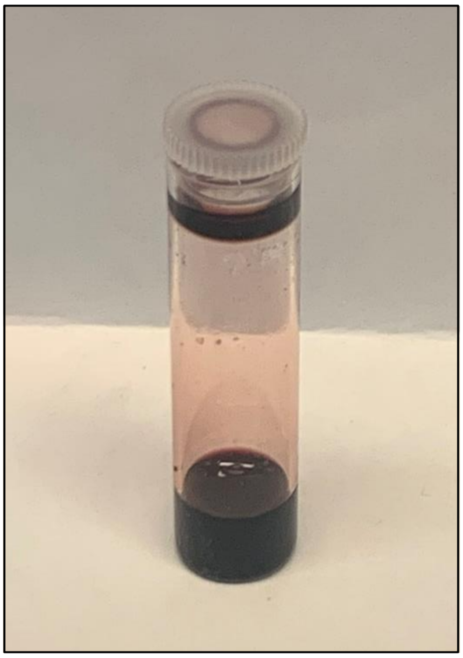

**Figure S4.** Photo of $Cu@(O_2CR^2)_{0.1}$ stored in the glove box freezer for 12 months; the solution is colloidal and well-dispersed with no signs of oxidation (i.e. change in colour from red to green) or Cu deposition.

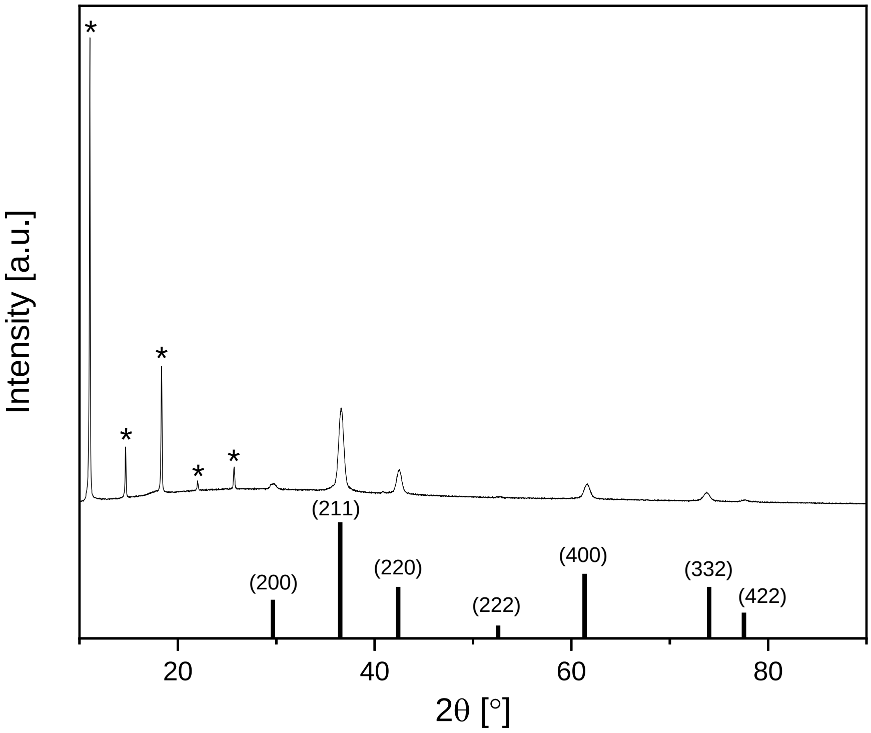


**Figure S5.** Powder X-ray diffraction pattern from the attempted synthesis of $Cu_2O@(O_2CR^2)_{0.1}$ from $[CuMes]_z$, 0.1 equiv. $HO_2CR^2$ and 0.5 equiv. of $H_2O$ under nitrogen; pattern indexed against cubic $Cu_2O$ as vertical bars (JCPDS 00-002-1067). * Represent unidentifiable diffraction peaks.

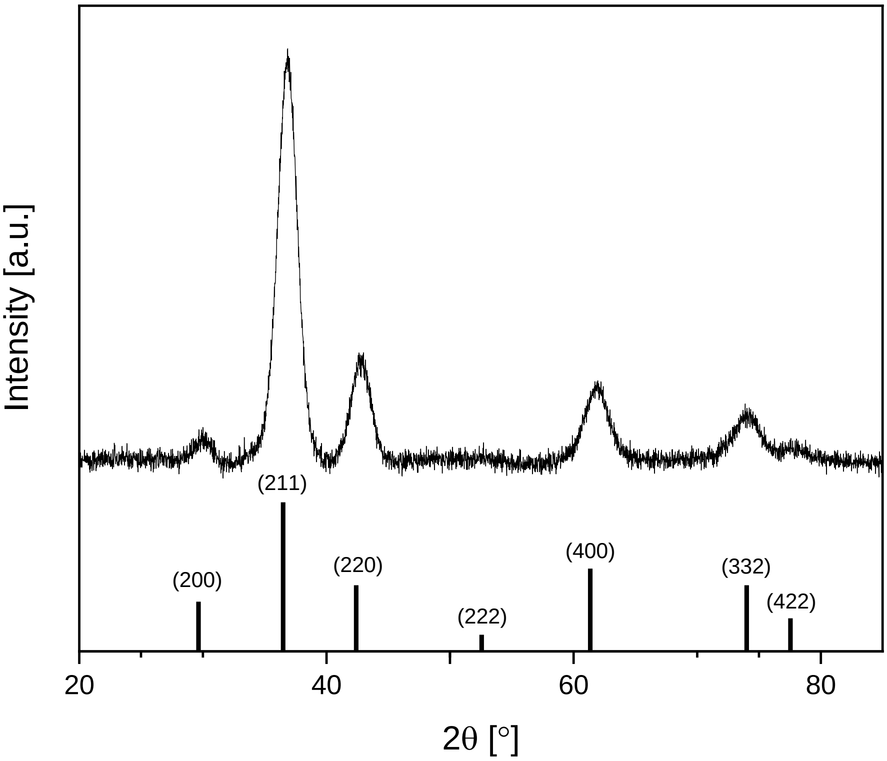


**Figure S6.** Powder X-ray diffraction pattern from the synthesis of $Cu_2O@(O_2CR^2)_{0.1}$ from $[CuMes]_z$, 0.1 equiv. $HO_2CR^2$ and 0.5 equiv. of $H_2O$ in air; pattern indexed against cubic $Cu_2O$ as vertical bars (JCPDS 00-002-1067). Average crystallite size = 4.8 nm (Scherrer analysis).

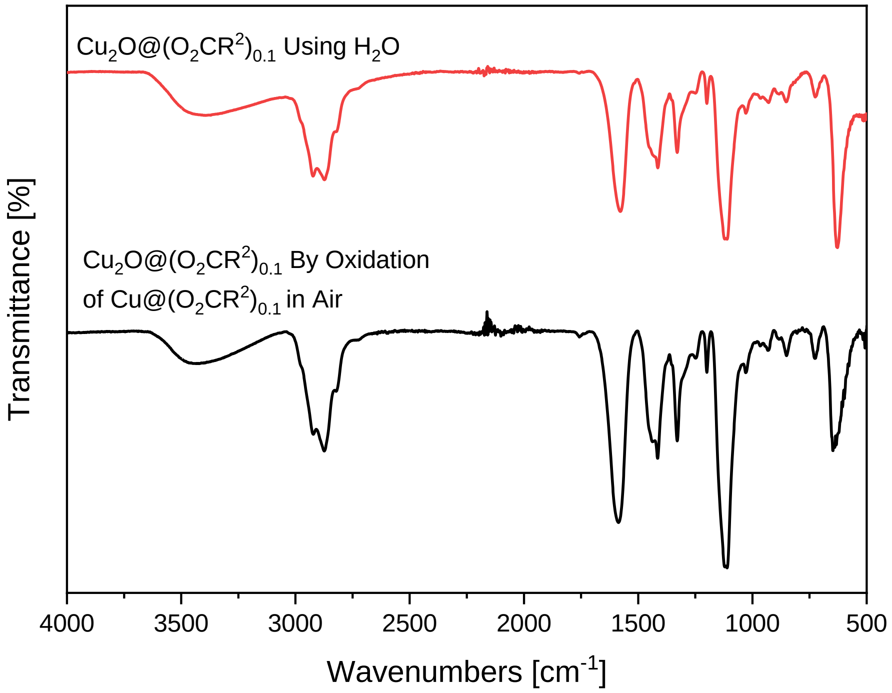


**Figure S7.** Stacked FT-IR spectra of $Cu_2O@(O_2CR^2)_{0.1}$ synthesized directly from [CuMes]z using $H_2O$ (top, red), and by oxidation of $Cu@(O_2CR^2)_{0.1}$ in air (bottom, black).

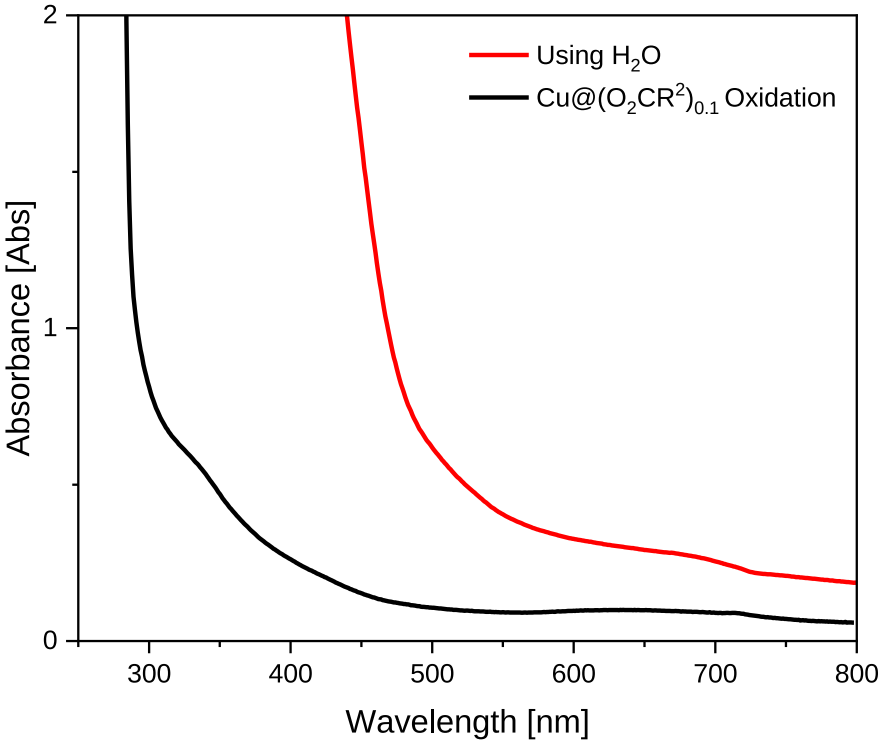


**Figure S8.** Stacked UV-Vis spectra of $Cu_2O@(O_2CR^2)_{0.1}$ synthesized using $H_2O$ (red, 4.2 mM solution in toluene) and by oxidation of $Cu@(O_2CR^2)_{0.1}$ in air (black, 0.5 mM solution in toluene). Both display absorption onsets between 725-735 nm and optical band-gaps of ~2.4 eV.

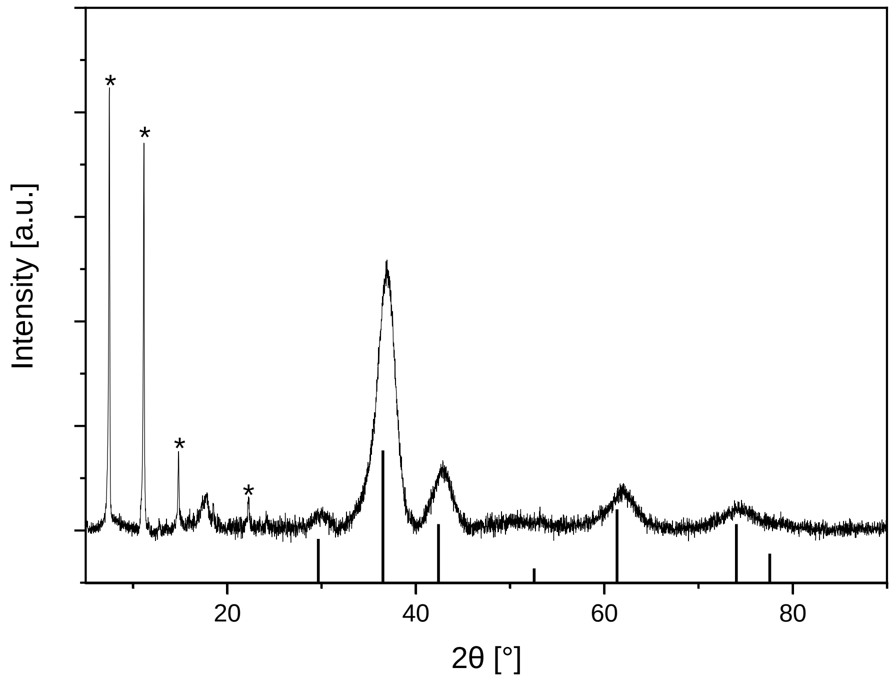


**Figure S9.** Powder X-ray diffraction pattern of the isolated material from the attempted synthesis of $Cu_2O@(O_2CR^1)_{0.2}$; pattern indexed against cubic $Cu_2O$ as vertical bars (JCPDS 00-002-1067). * Represent unidentifiable diffraction peaks.

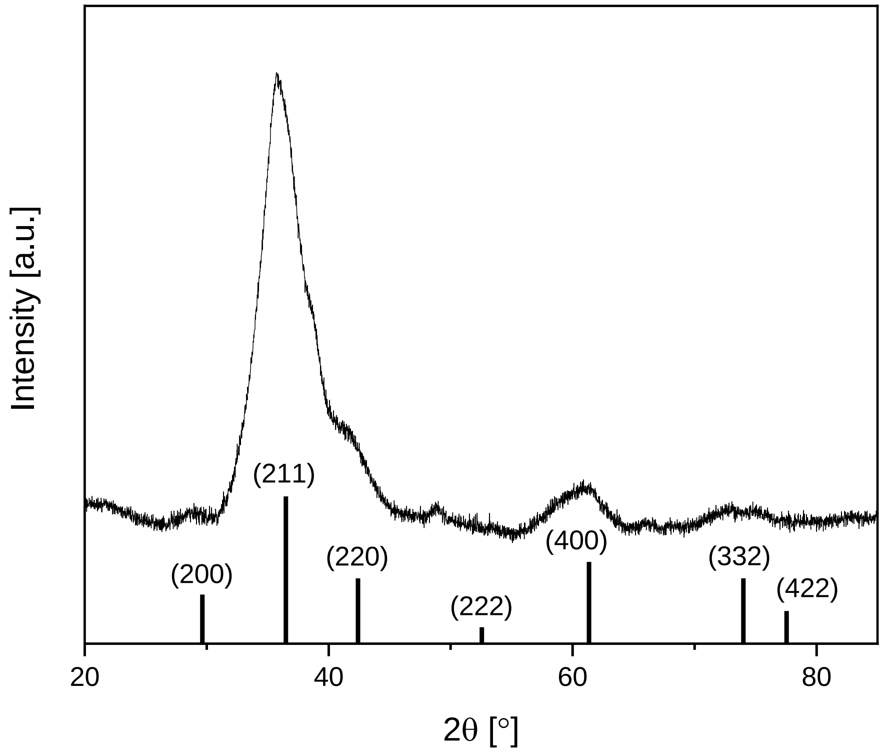


**Figure S10.** Powder X-ray diffraction pattern of the isolated precipitate from an attempted synthesis of $Cu_2O@(S_2CR^1)_{0.1}$; pattern indexed against cubic $Cu_2O$ as vertical bars (JCPDS 00-002-1067).

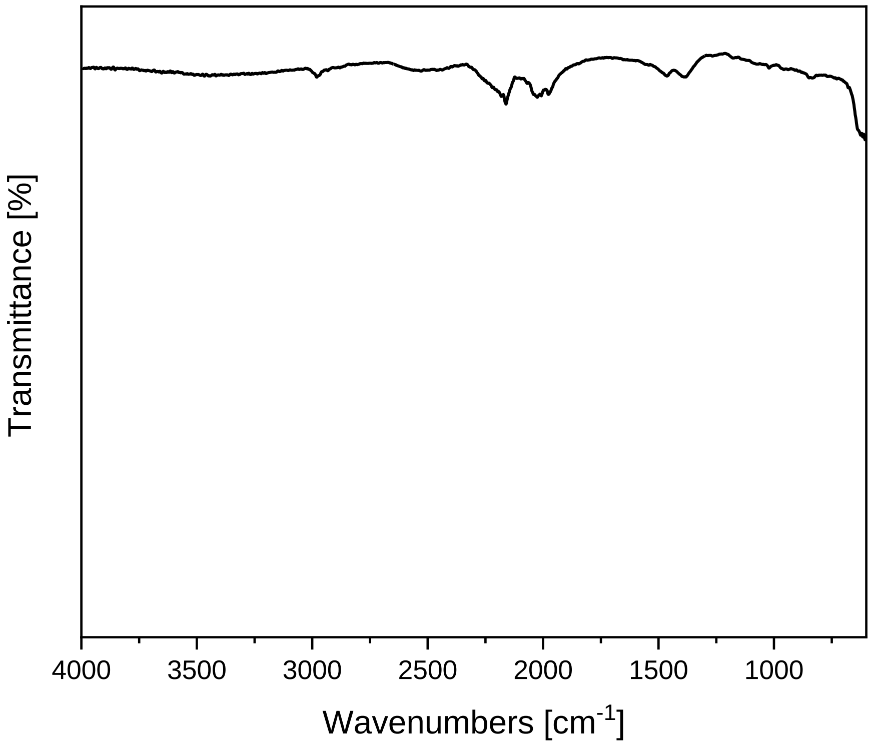


**Figure S11.** FT-IR spectrum of the isolated precipitate from an attempted synthesis of $Cu_2O@(S_2CR^1)_{0.1}$.

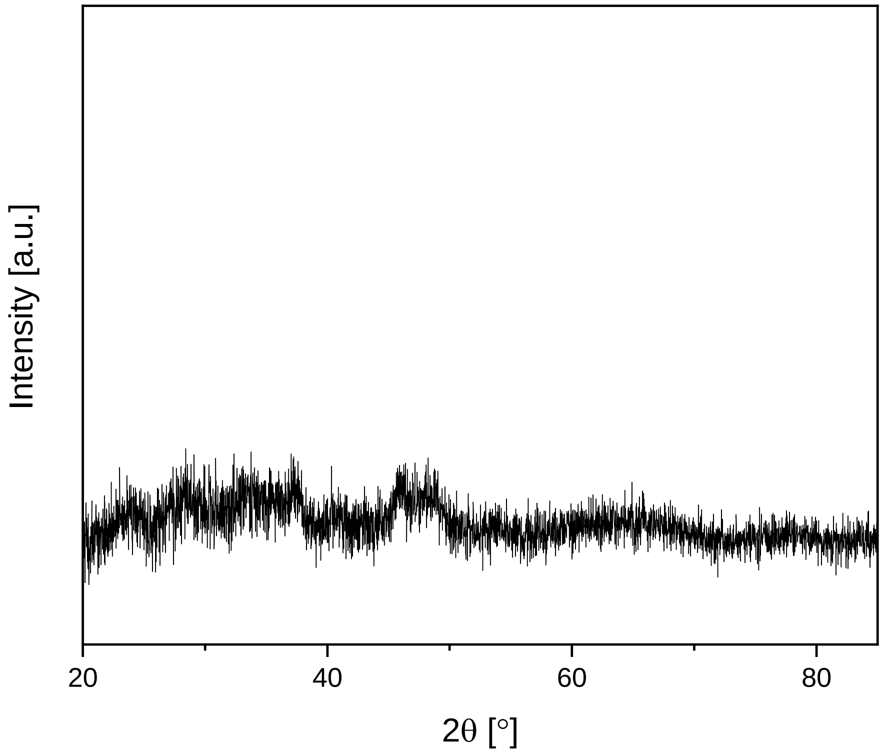


**Figure S12.** Powder X-ray diffraction pattern of the isolated product from the attempted synthesis of $Cu_2S@(O_2CR^1)_{0.1}$ when the reaction was conducted for 16 hours at room temperature.

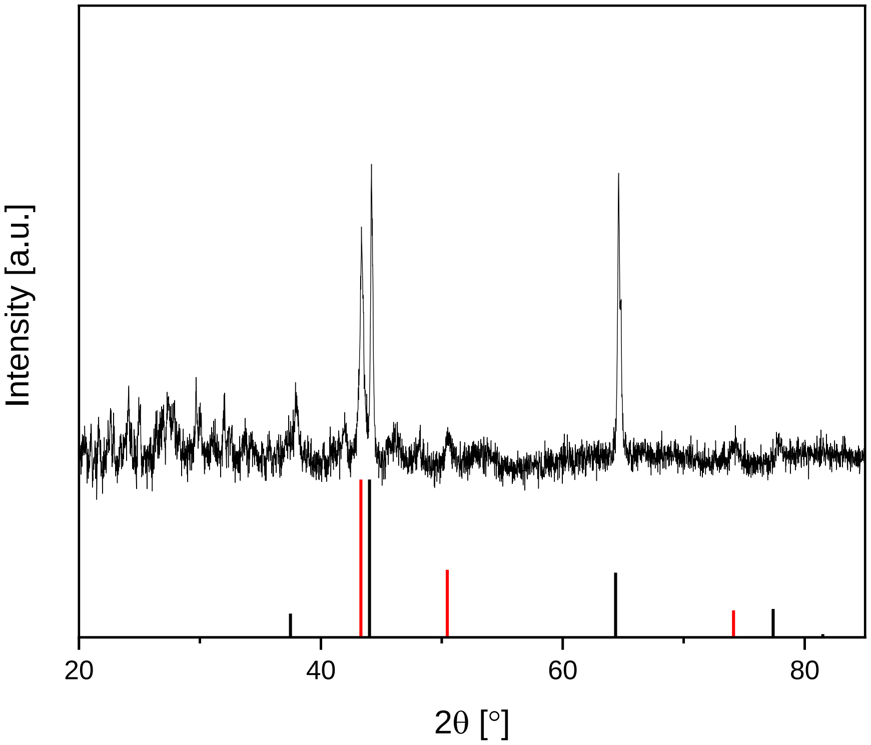


**Figure S13.** Powder X-ray diffraction pattern of the isolated product from the attempted synthesis of $Cu_2S@(O_2CR^2)_{0.1}$ when the reaction was conducted for 16 hours at room temperature; pattern indexed against cubic Cu (red; JCPDS 01-085-1326) and cubic silicon carbide (black; JCPDS 00-049-1623) as vertical bars; the presence of silicon carbide is attributed to small amounts of residue left on the diffractometer by previous users.

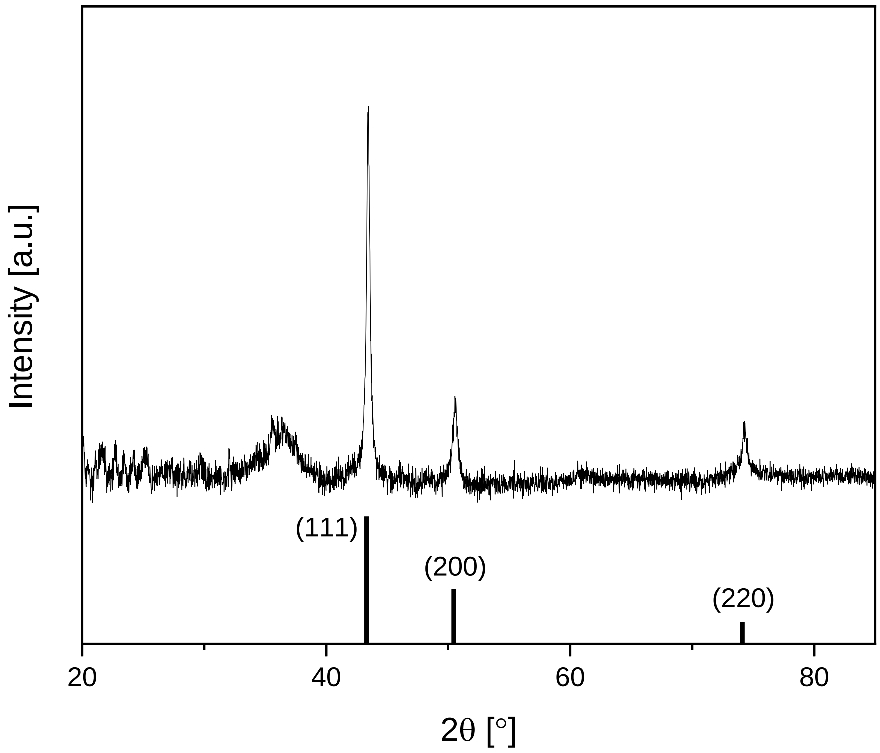


**Figure S14.** Powder X-ray diffraction pattern of the isolated product from the attempted synthesis of $Cu_2S@(O_2CR^1)_{0.1}$ when the reaction was conducted for 16 hours at 100 °C; pattern indexed against cubic Cu as vertical bars (JCPDS 01-085-1326).

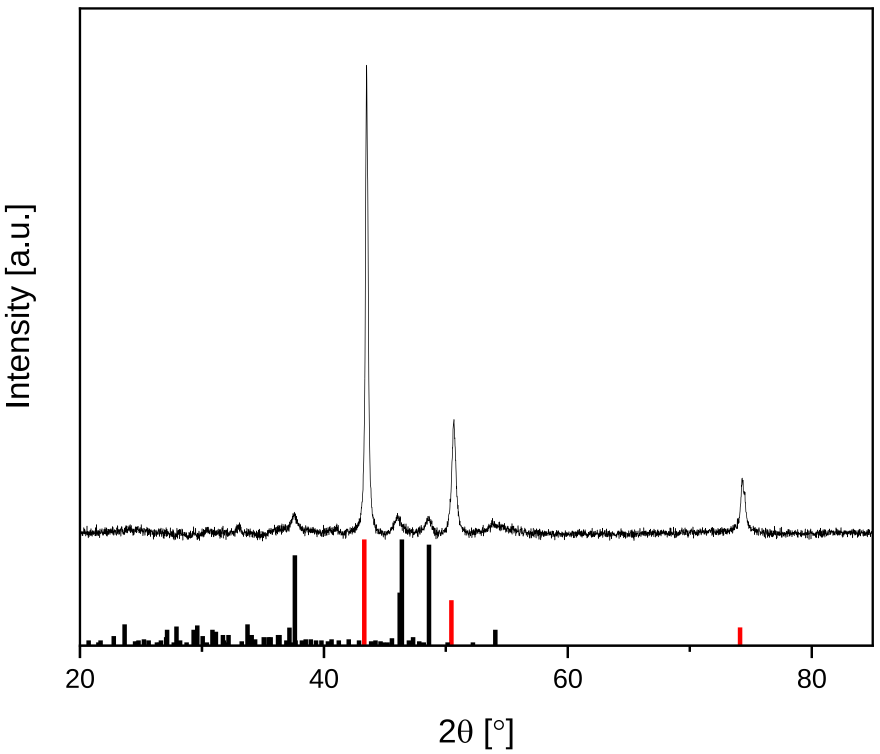


**Figure S15.** Powder X-ray diffraction pattern of the isolated product from the attempted synthesis of $Cu_2S@(O_2CR^2)_{0.1}$ when the reaction was conducted for 16 hours at 100 °C; pattern indexed against cubic Cu (red; JCPDS 01-085-1326) and monoclinic djurleite (black; JCPDS 00-034-0660) as vertical bars. JCPDS 00-034-0660 was collected using Mo Kα as a radiation source and transformed to Cu Kα using X’Pert HighScore Plus software.

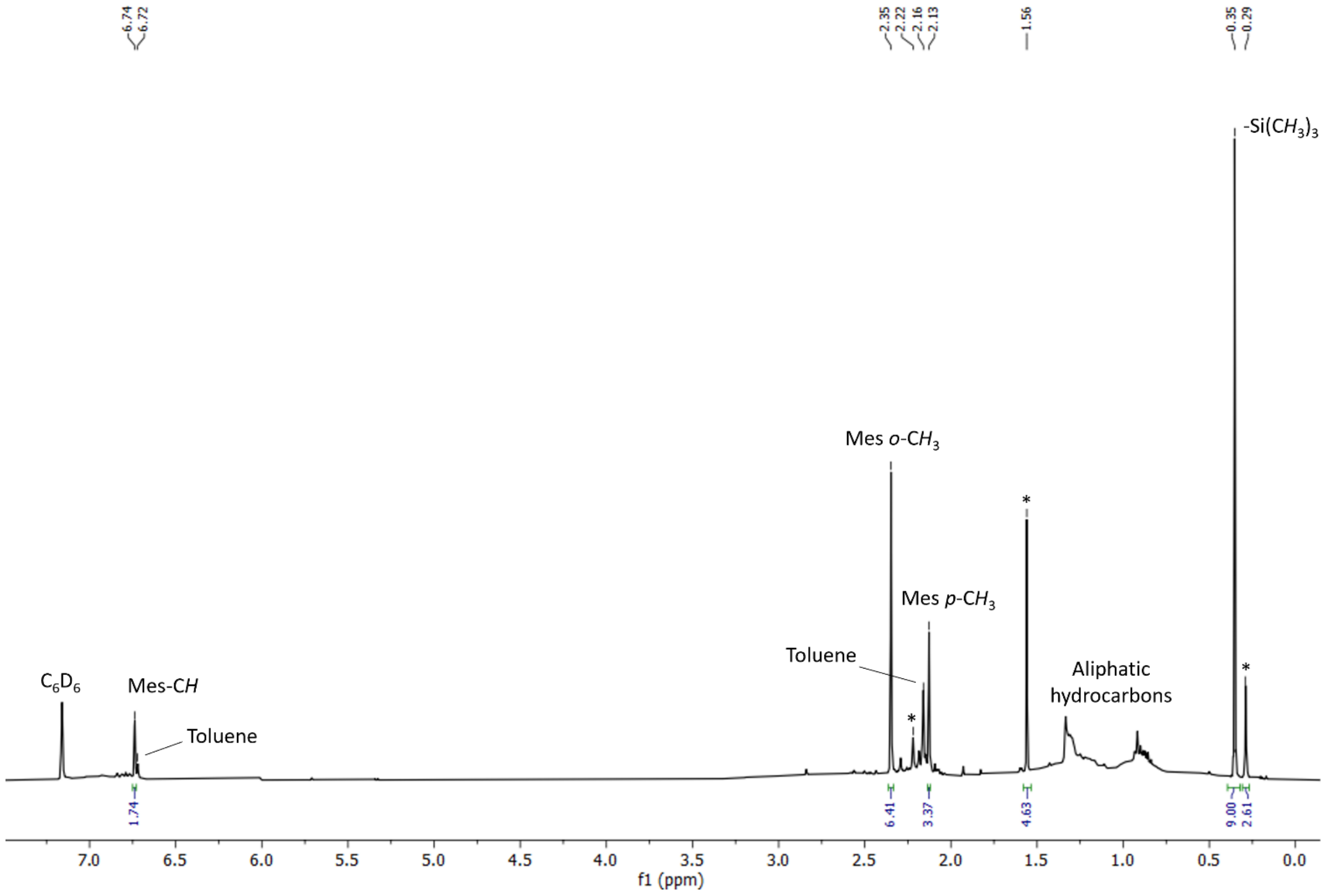


**Figure S16.** $^{1}H$ NMR spectrum of acetone mother liquors evaporated to dryness under reduced pressure, following isolating $Cu_2S@(S_2CR^1)_{0.1}$ by centrifugation ($C_6D_6$, 400 MHz, 20 °C). $Cu_2S@(S_2CR^1)_{0.1}$ was synthesized using $S(SiMe_3)_2$, and the major reaction byproduct observed is Mes-$SiMe_3$;[53] no unreacted $HS_2CR^1$ is observed (see Figure S58 for reference). * = unidentified species.

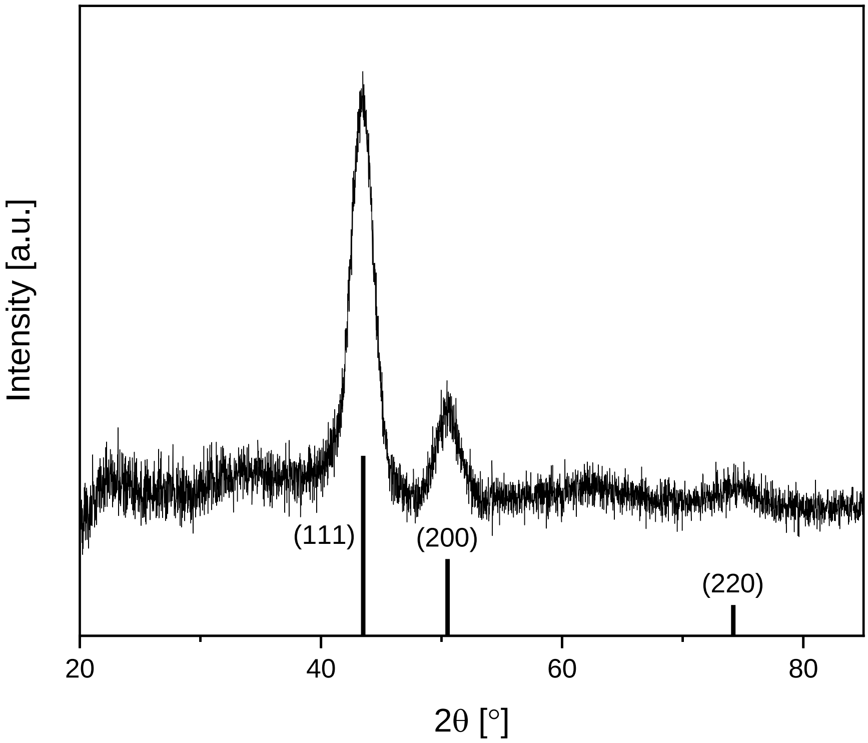


**Figure S17.** Powder X-ray diffraction pattern of Cu@$(O_2CR^1)_{0.1}$ (3.9 nm); pattern indexed against cubic Cu as vertical bars (JCPDS 01-085-1326).

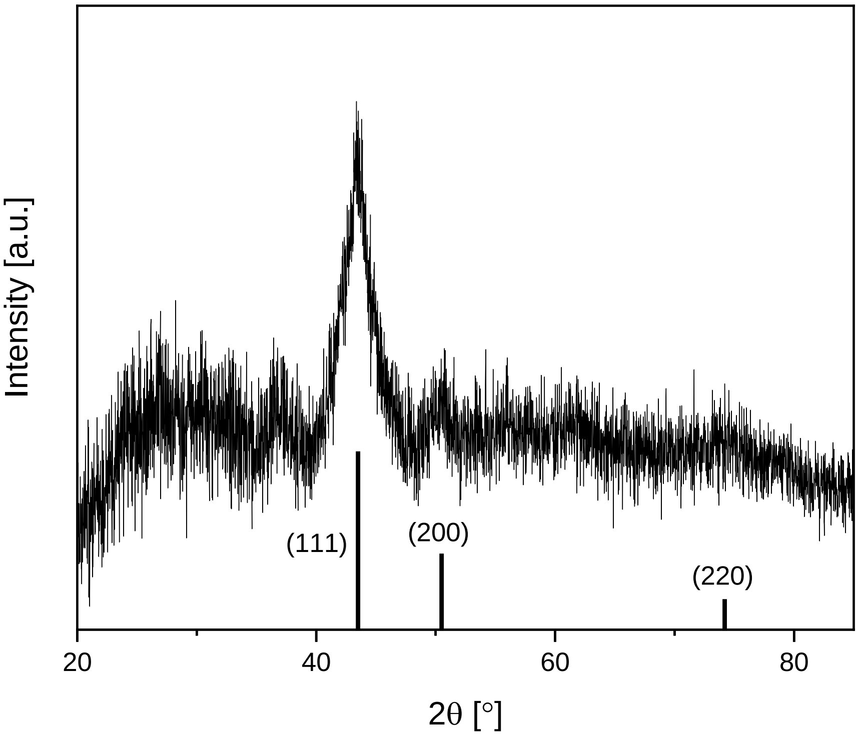


**Figure S18.** Powder X-ray diffraction pattern of Cu@$(O_2CR^2)_{0.1}$ (3.1 nm); pattern indexed against cubic Cu as vertical bars (JCPDS 01-085-1326).

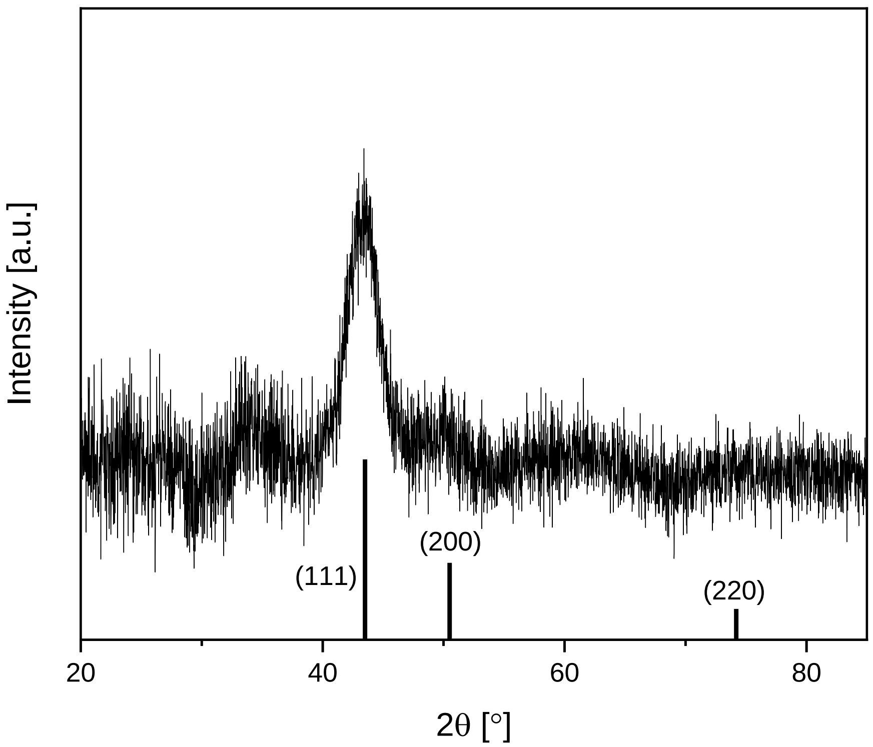


**Figure S19.** Powder X-ray diffraction pattern of Cu@$(S_2CR^1)_{0.1}$ (3.0 nm); pattern indexed against cubic Cu as vertical bars (JCPDS 01-085-1326).

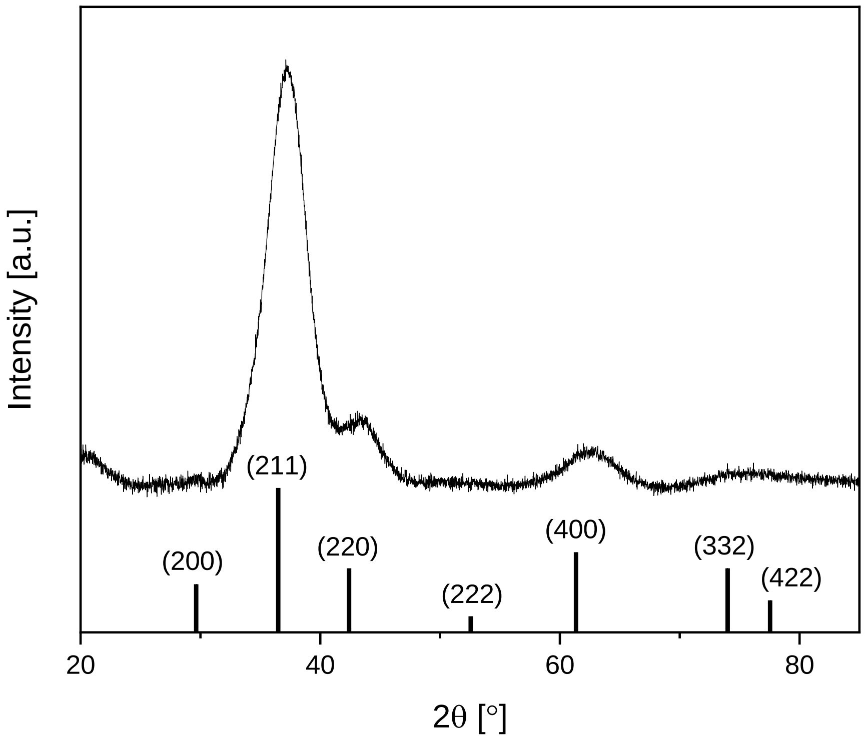


**Figure S20.** Powder X-ray diffraction pattern of $Cu_2O$@$(O_2CR^1)_{0.1}$ (2.4 nm) when oxidized in air; pattern indexed against cubic $Cu_2O$ as vertical bars (JCPDS 00-002-1067).

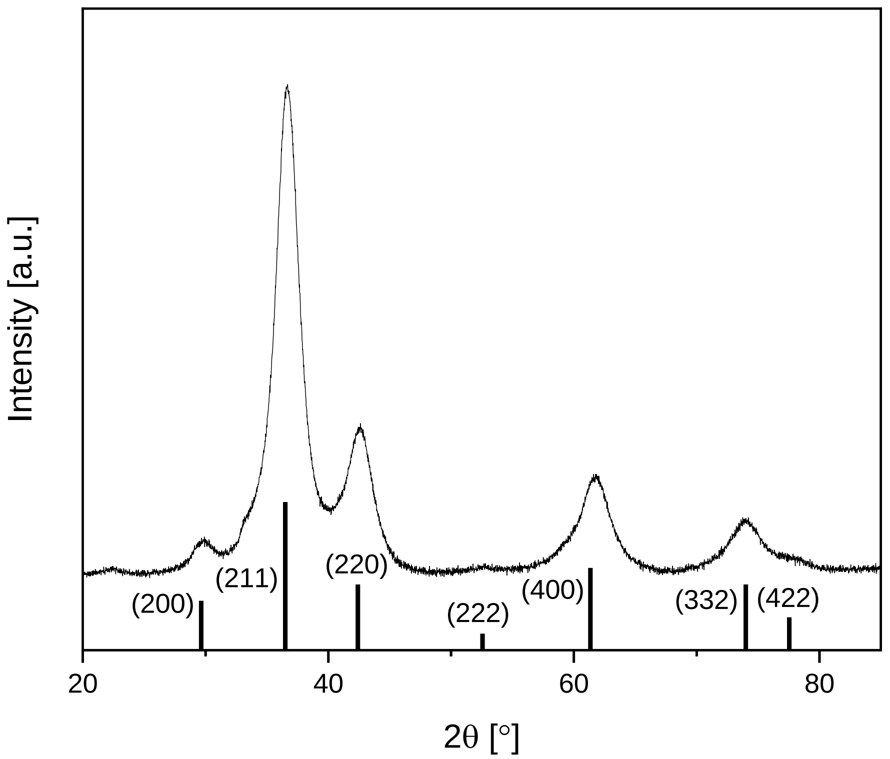


**Figure S21.** Powder X-ray diffraction pattern of $Cu_2O@(O_2CR^2)_{0.1}$ (3.1 nm) when oxidized in air; pattern indexed against cubic $Cu_2O$ as vertical bars (JCPDS 00-002-1067).

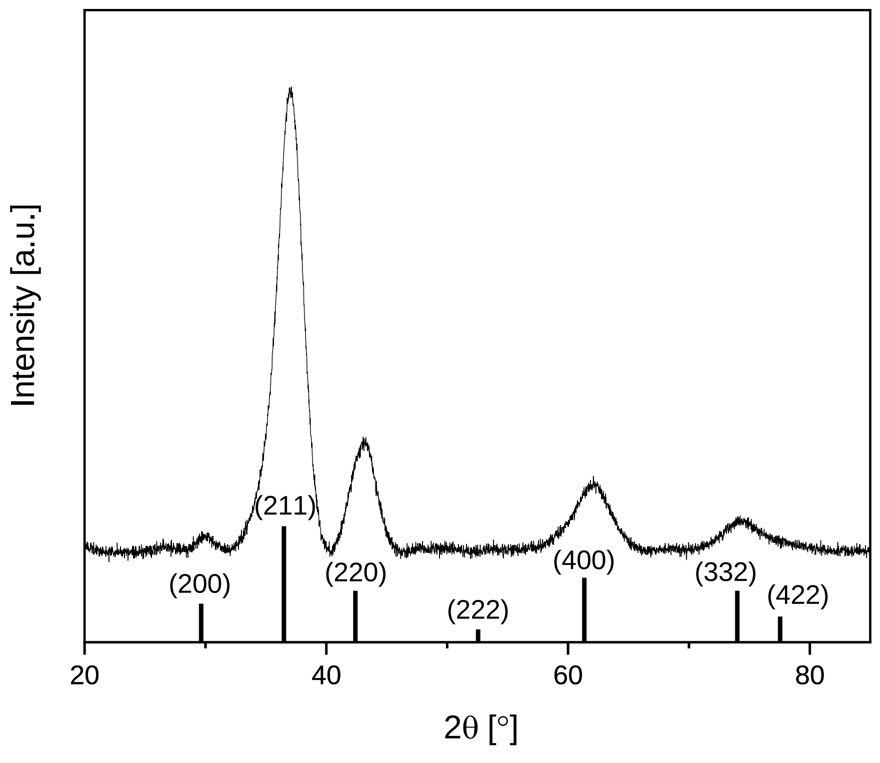


**Figure S22.** Powder X-ray diffraction pattern of $Cu_2O@(O_2CR^2)_{0.2}$ (3.1 nm); pattern indexed against cubic $Cu_2O$ as vertical bars (JCPDS 00-002-1067).

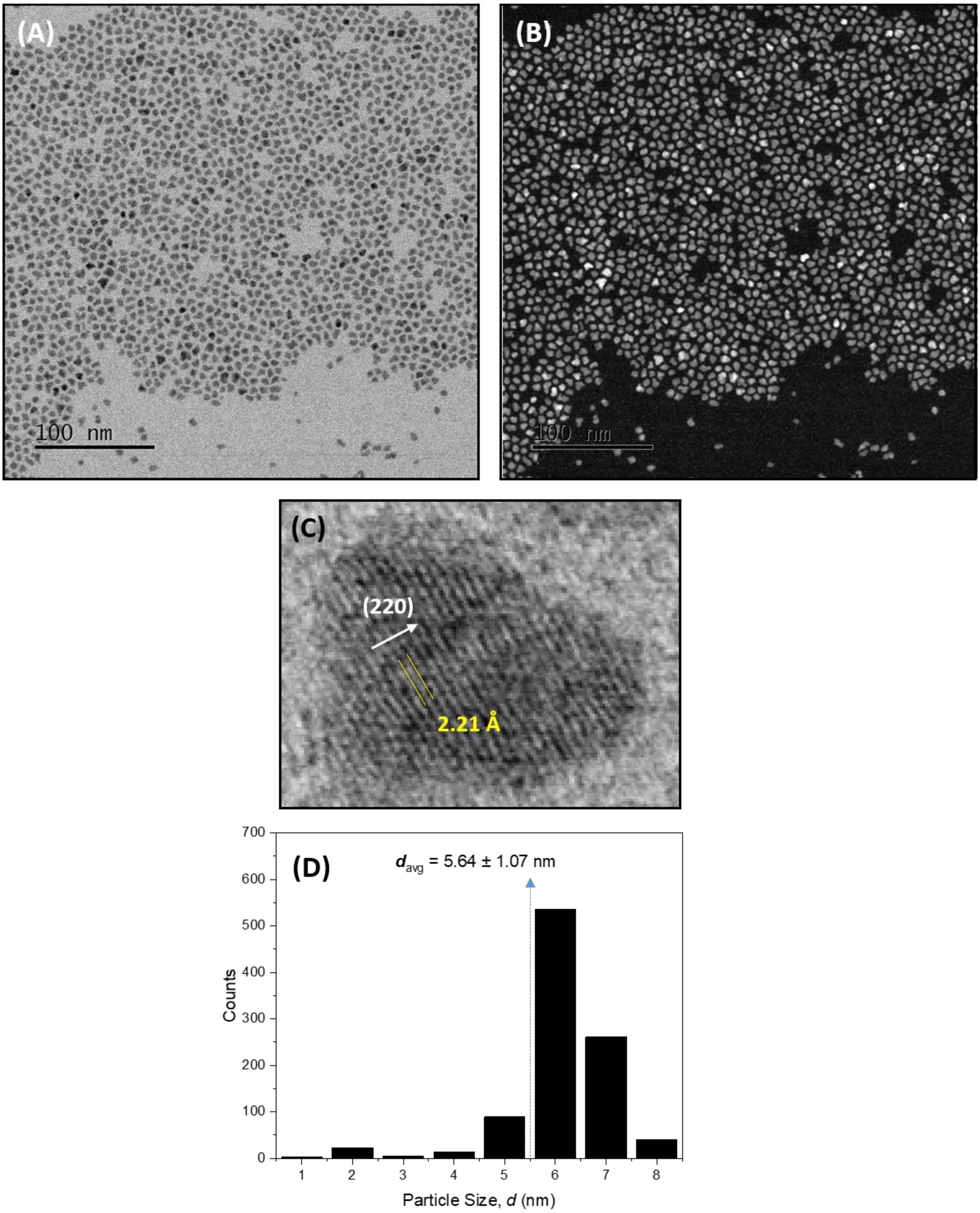


**Figure S23. (A)** Annular bright field STEM image, **(B)** annular dark field STEM image and **(C)** HR-TEM image with lattice fringes for $Cu_2O@(O_2CR^1)_{0.1}$. **(D)** Size distribution histogram obtained from the TEM data for $Cu_2O@(O_2CR^1)_{0.1}$.

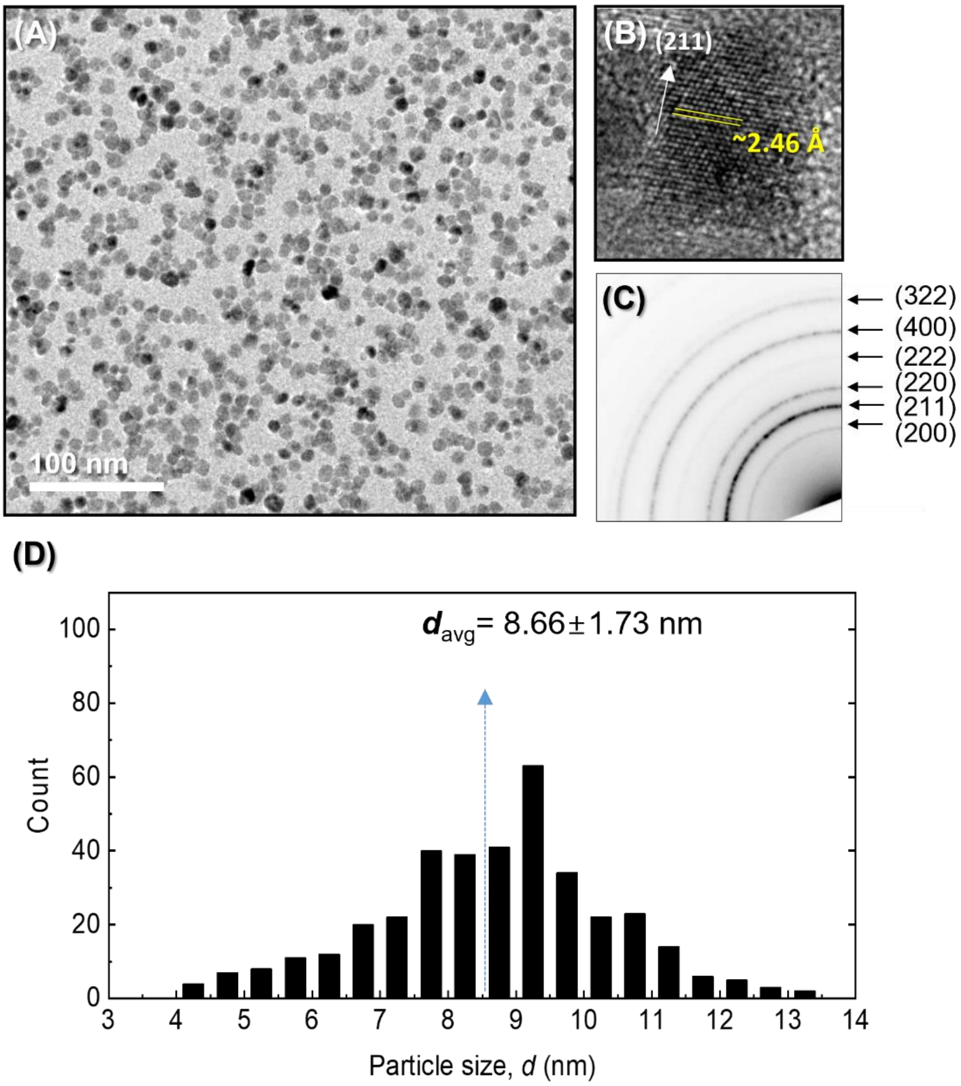


**Figure S24. (A)** Annular bright field STEM image, **(B)** HR-TEM image with lattice fringes, and **(C)** SAED pattern for $Cu_2O@(O_2CR^2)_{0.1}$. **(D)** Size distribution histogram obtained from the TEM data for $Cu_2O@(O_2CR^2)_{0.1}$.

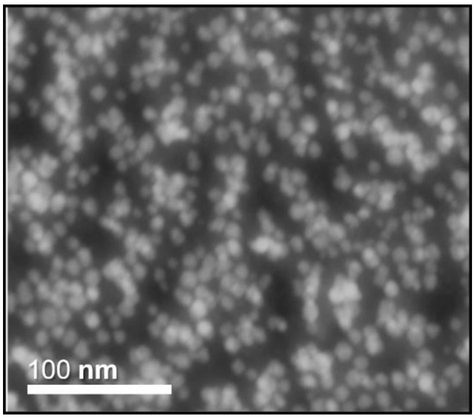


**Figure S25.** Annular dark field STEM image of $Cu_2O@(O_2CR^2)_{0.1}$.

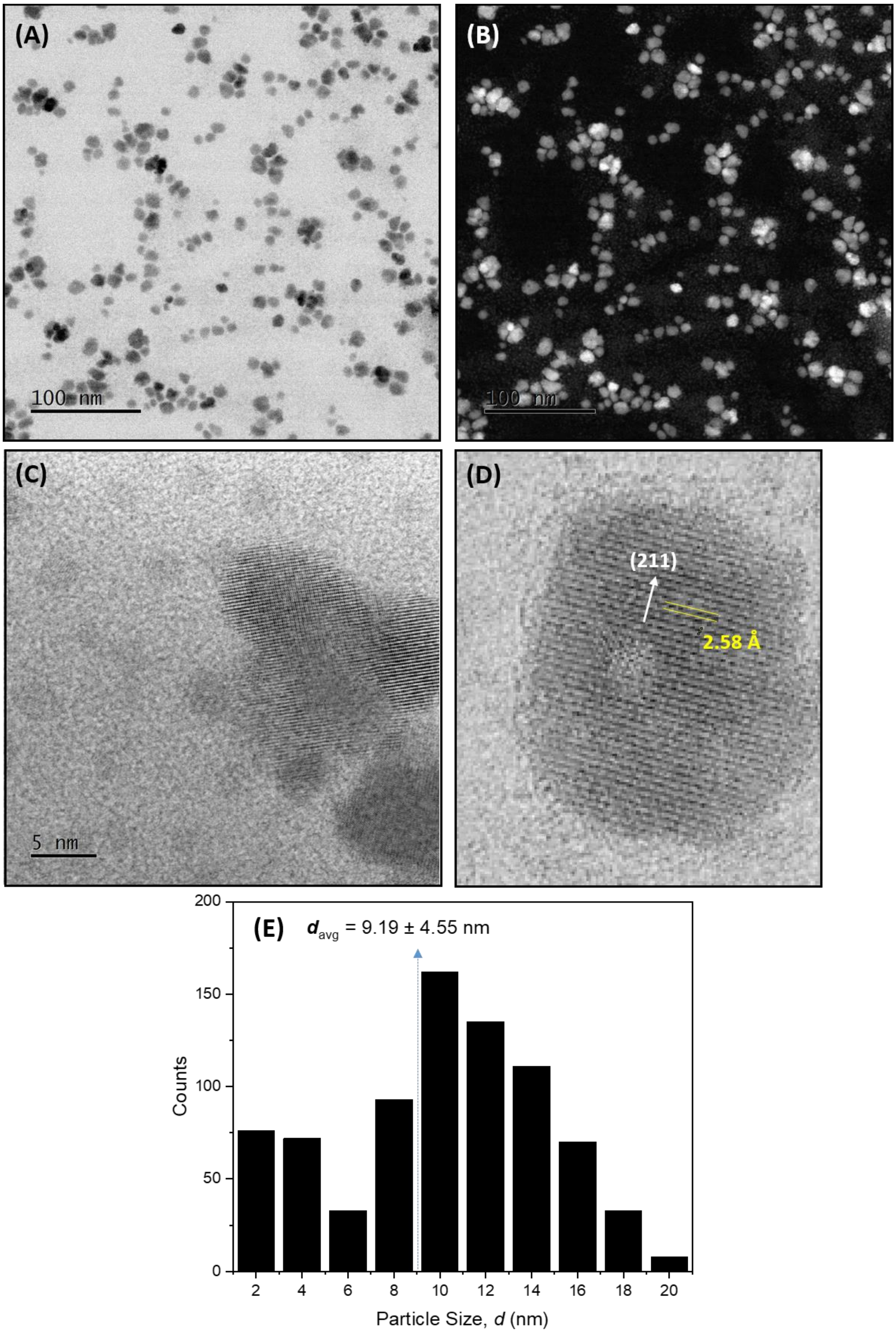


**Figure S26. (A)** Annular bright field STEM image, **(B)** annular dark field STEM image, **(C)** TEM image depicting the polycrystallinity of the sample and **(D)** HR-TEM image with lattice fringes for $Cu_2O@(O_2CR^2)_{0.2}$. **(E)** Size distribution histogram obtained from the TEM data for $Cu_2O@(O_2CR^2)_{0.2}$.

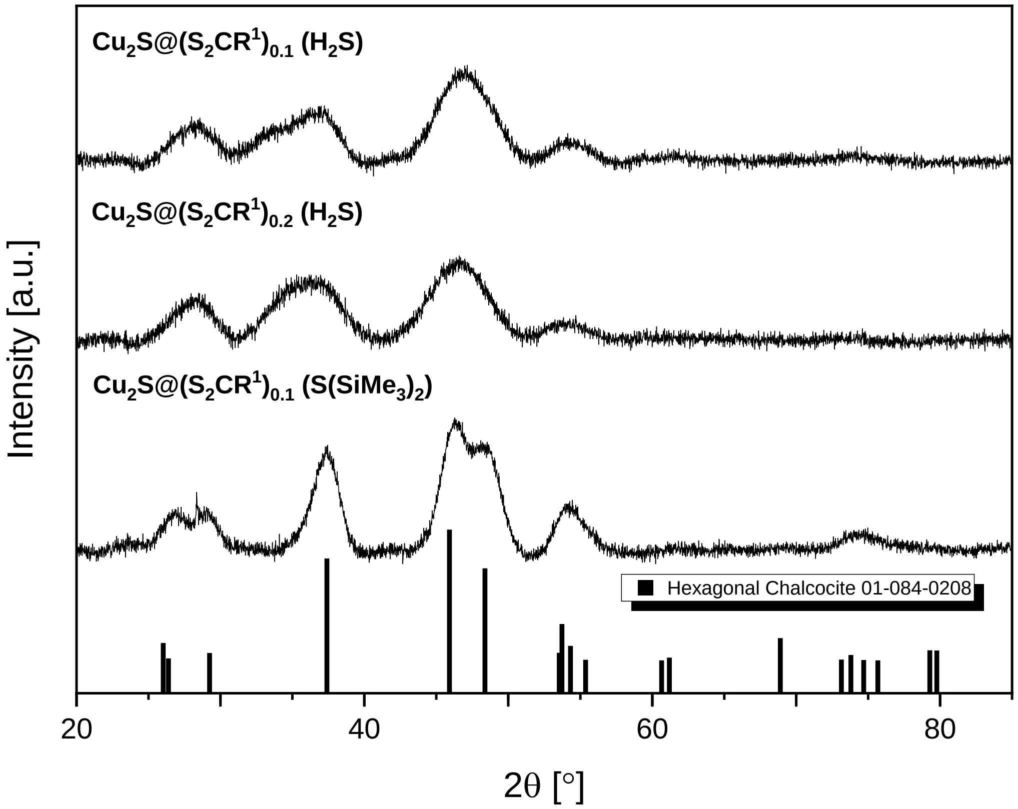


**Figure S27.** Powder X-ray diffraction patterns of $Cu_2S@(S_2CR^1)_{0.1}$ and $Cu_2S@(S_2CR^1)_{0.2}$ synthesized using $H_2S$, and $Cu_2S@(S_2CR^1)_{0.1}$ synthesized using $S(SiMe_3)_2$, indexed against hexagonal chalcocite, $Cu_2S$, as black vertical bars (JCPDS 01-084-0208).

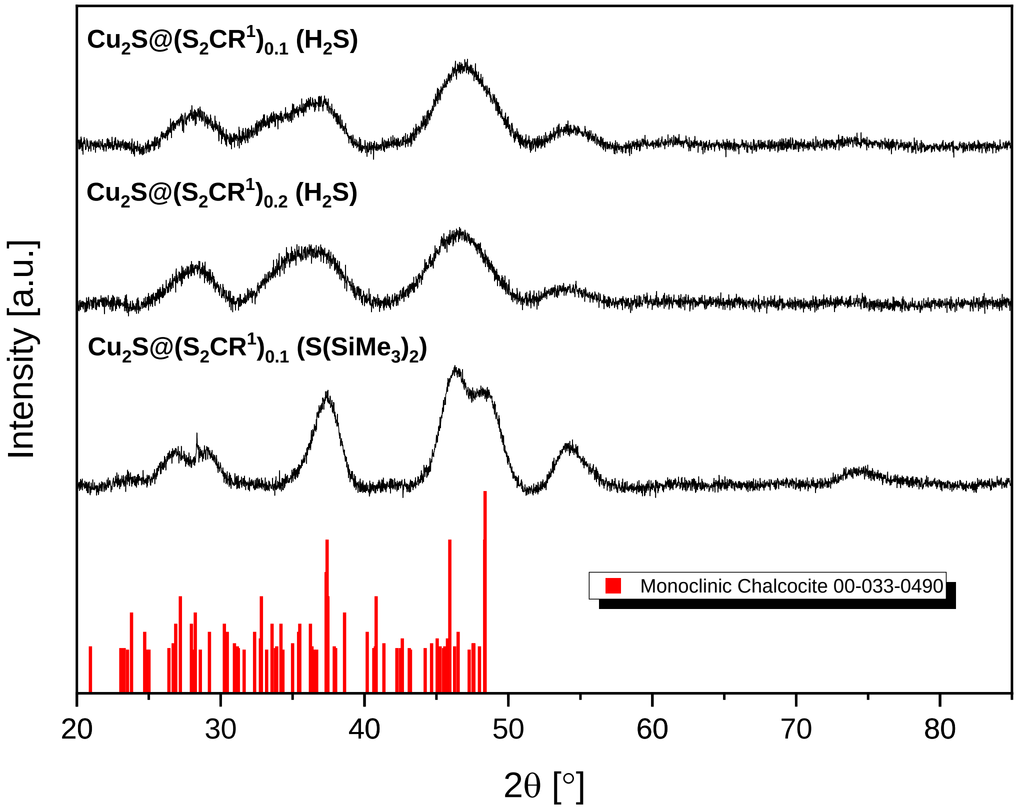


**Figure S28.** Powder X-ray diffraction patterns of $Cu_2S@(S_2CR^1)_{0.1}$ and $Cu_2S@(S_2CR^1)_{0.2}$ synthesized using $H_2S$, and $Cu_2S@(S_2CR^1)_{0.1}$ synthesized using $S(SiMe_3)_2$, indexed against monoclinic chalcocite, $Cu_2S$, as red vertical bars (JCPDS 00-033-0490). JCPDS 00-033-0490 was collected using Mo Kα as a radiation source and transformed to Cu Kα using X'Pert HighScore Plus software.

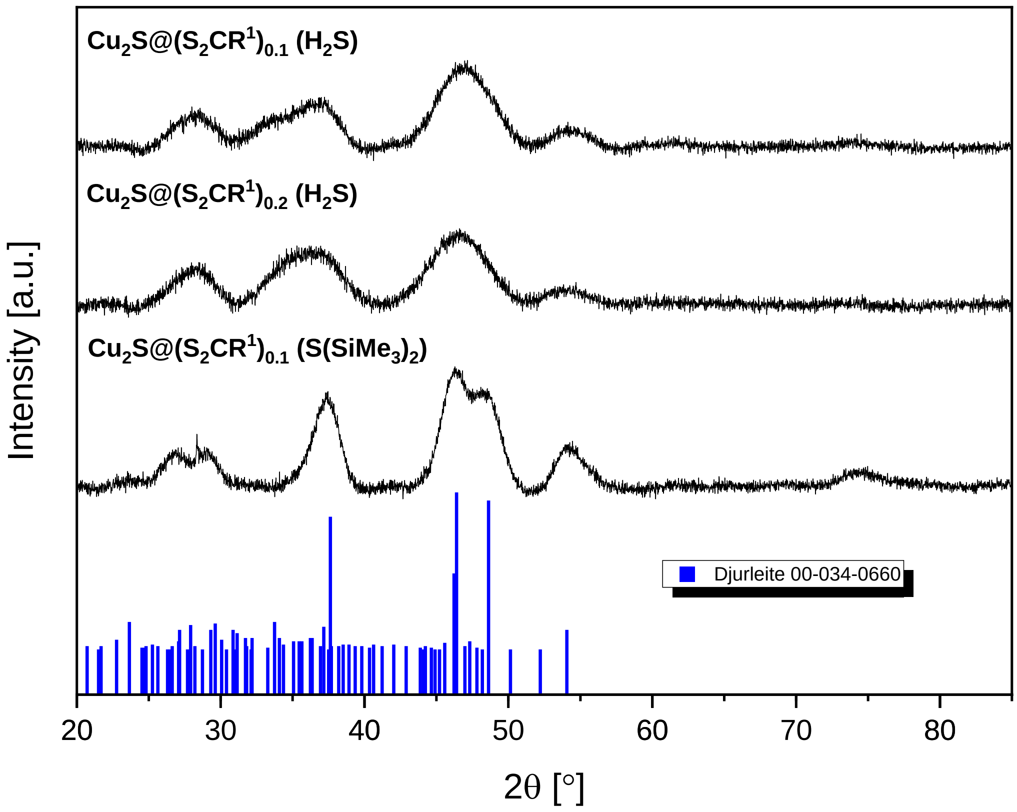


**Figure S29.** Powder X-ray diffraction patterns of $Cu_2S@(S_2CR^1)_{0.1}$ and $Cu_2S@(S_2CR^1)_{0.2}$ synthesized using $H_2S$, and $Cu_2S@(S_2CR^1)_{0.1}$ synthesized using $S(SiMe_3)_2$, indexed against djurleite, $Cu_{1.94}S$, as blue vertical bars (JCPDS 00-034-0660). JCPDS 00-034-0660 was collected using Mo Kα as a radiation source and transformed to Cu Kα using X'Pert HighScore Plus software.

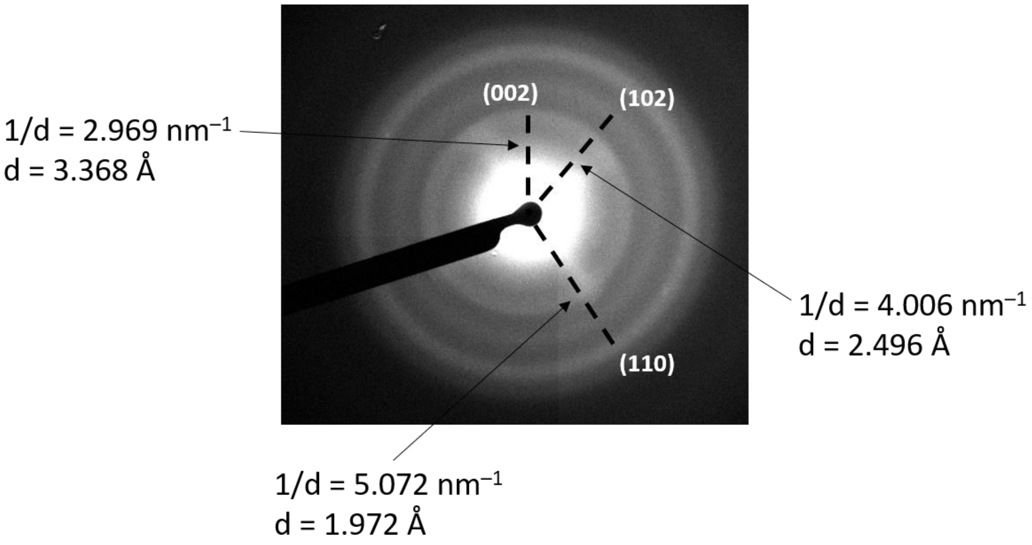


**Figure S30.** SAED pattern of $Cu_2S@(S_2CR^1)_{0.1}$ when synthesized using $H_2S$.

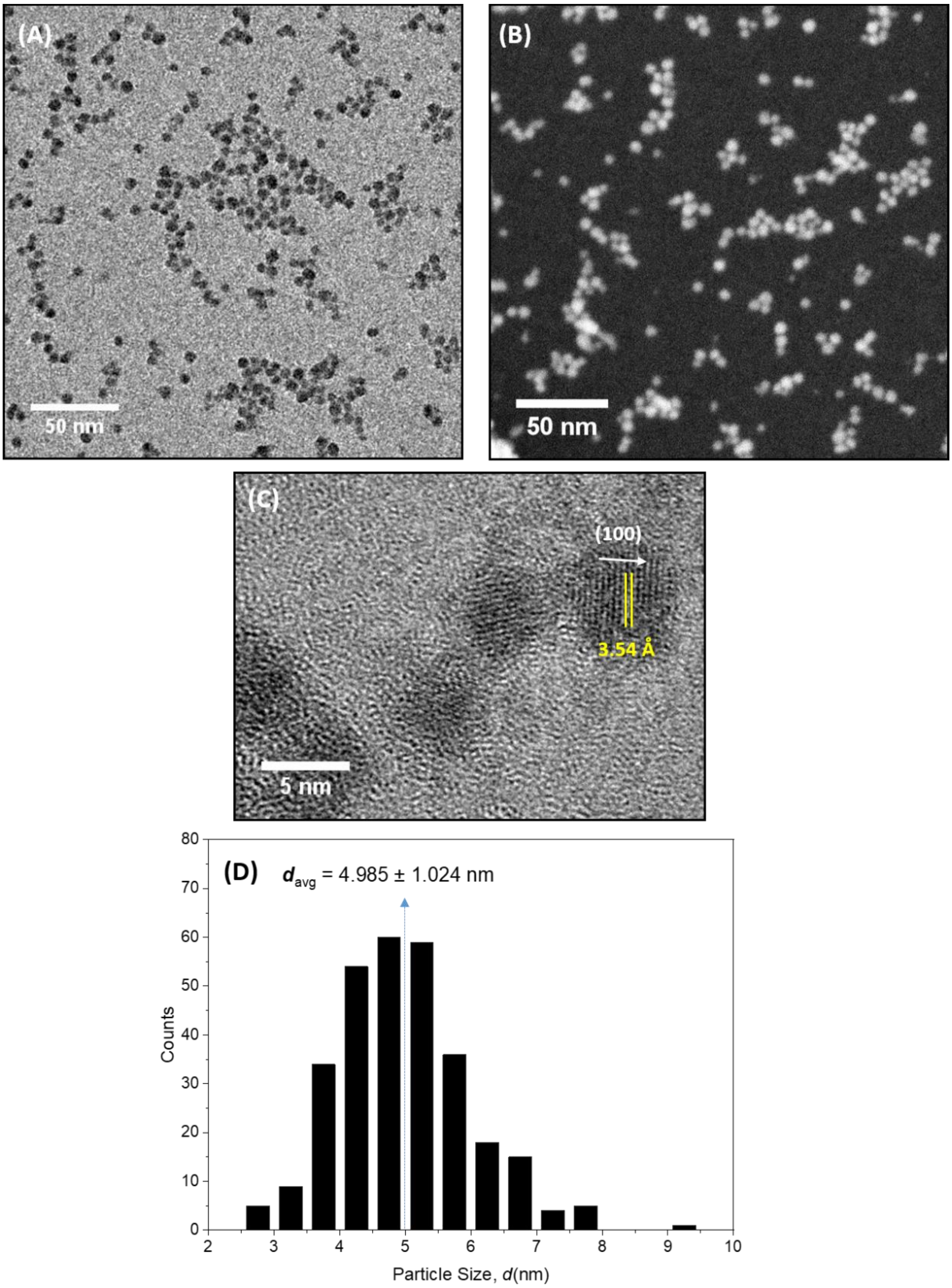


**Figure S31. (A)** Annular bright field STEM image, **(B)** annular dark field STEM image and **(C)** HR-TEM image and lattice fringes for $Cu_2S@(S_2CR^1)_{0.1}$ when synthesized using $S(SiMe_3)_2$ as the sulfiding agent. **(D)** Size distribution histogram obtained from the TEM data for $Cu_2S@(S_2CR^1)_{0.1}$ when synthesized using $S(SiMe_3)_2$.

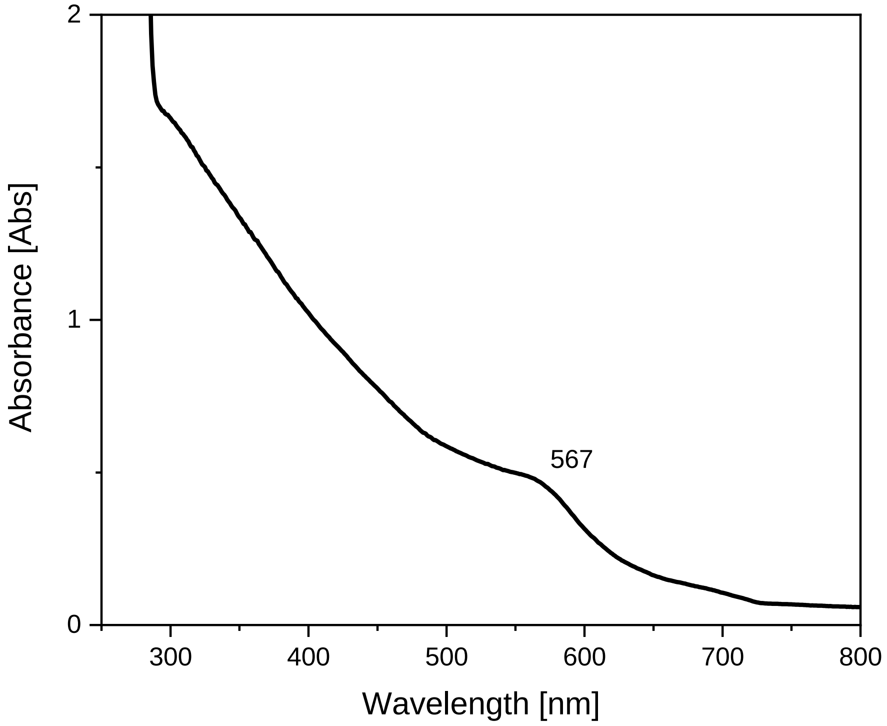


**Figure S32.** UV-Vis spectrum of Cu@($O_2CR^1$)$_{0.1}$ (0.5 mM solution in toluene).

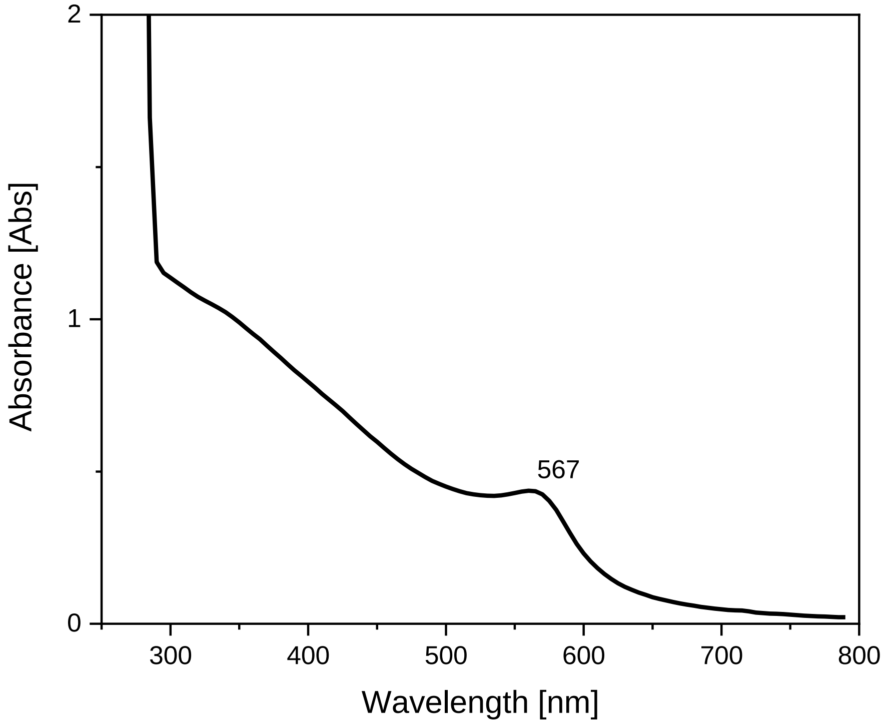


**Figure S33.** UV-Vis spectrum of Cu@($O_2CR^2$)$_{0.1}$ (0.5 mM solution in toluene).

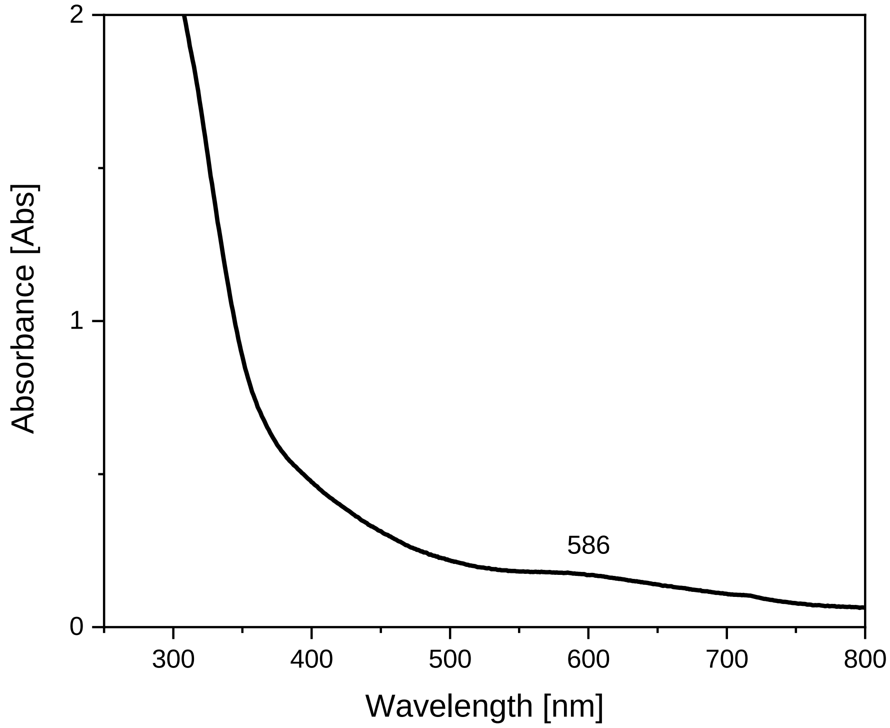


**Figure S34.** UV-Vis spectrum of Cu@($S_2CR^1$)$_{0.1}$ (0.5 mM solution in toluene).

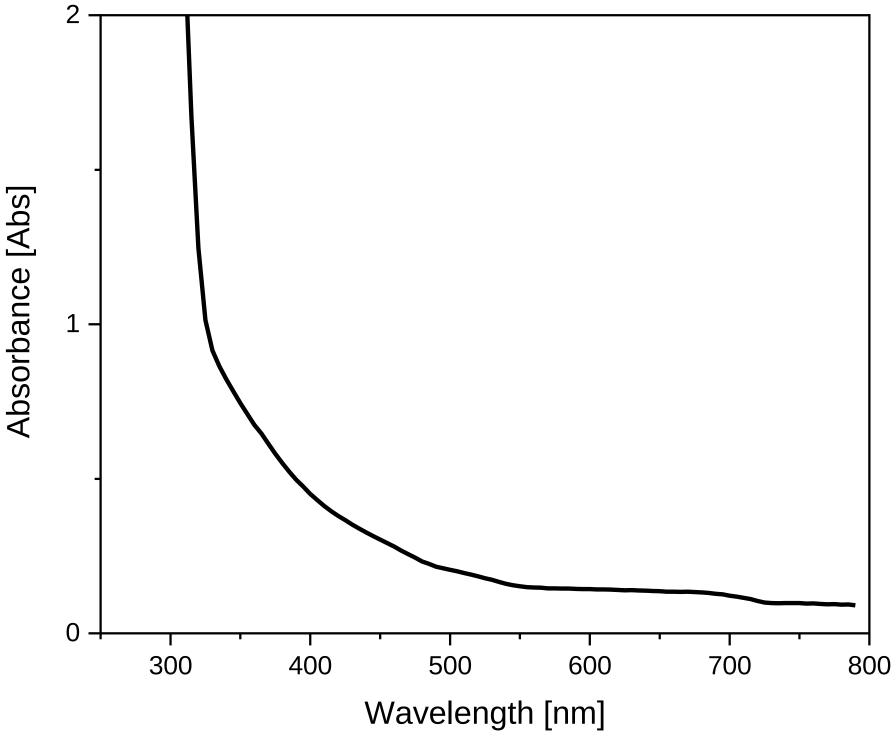


**Figure S35.** UV-Vis spectrum of $Cu_2O$@($O_2CR^1$)$_{0.1}$ (0.5 mM solution in toluene) when synthesized in air.

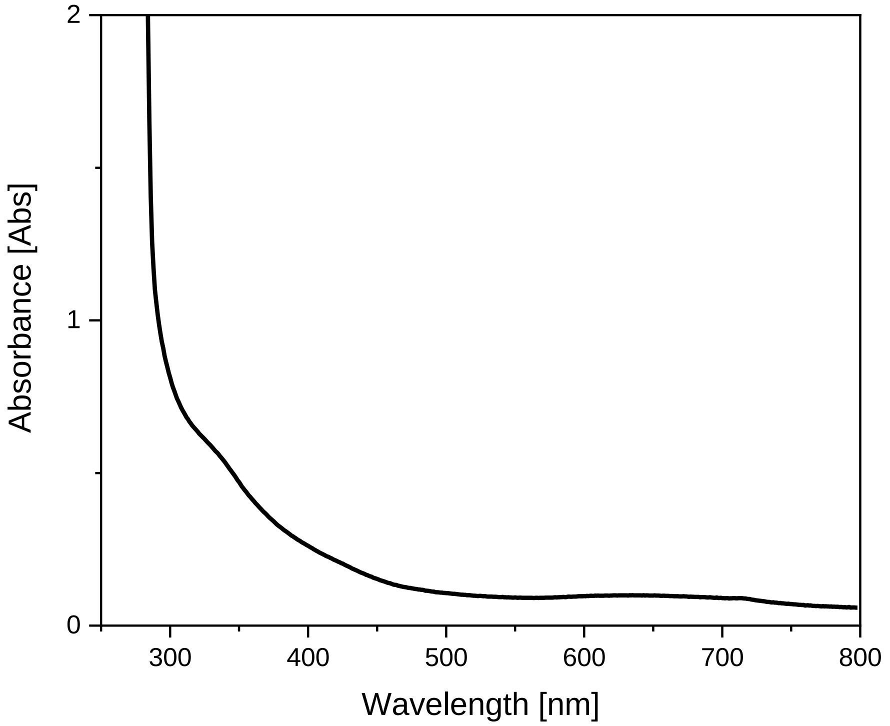


**Figure S36.** UV-Vis spectrum of $Cu_2O@(O_2CR^2)_{0.1}$ (0.5 mM solution in toluene) when synthesized in air.

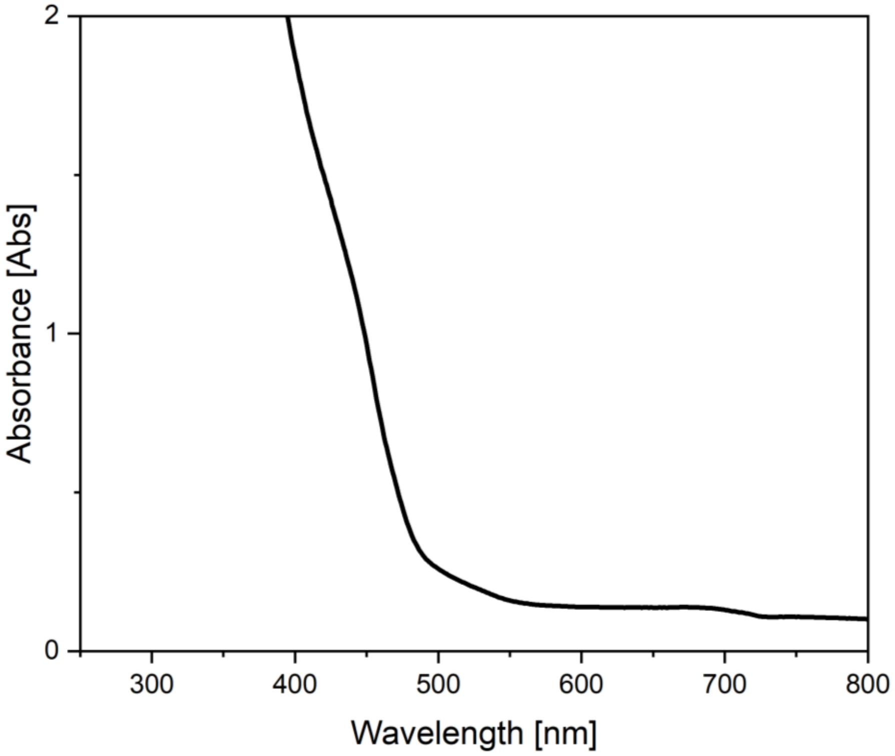


**Figure S37.** UV-Vis spectrum of $Cu_2O@(O_2CR^2)_{0.2}$ (4 mM solution in toluene).

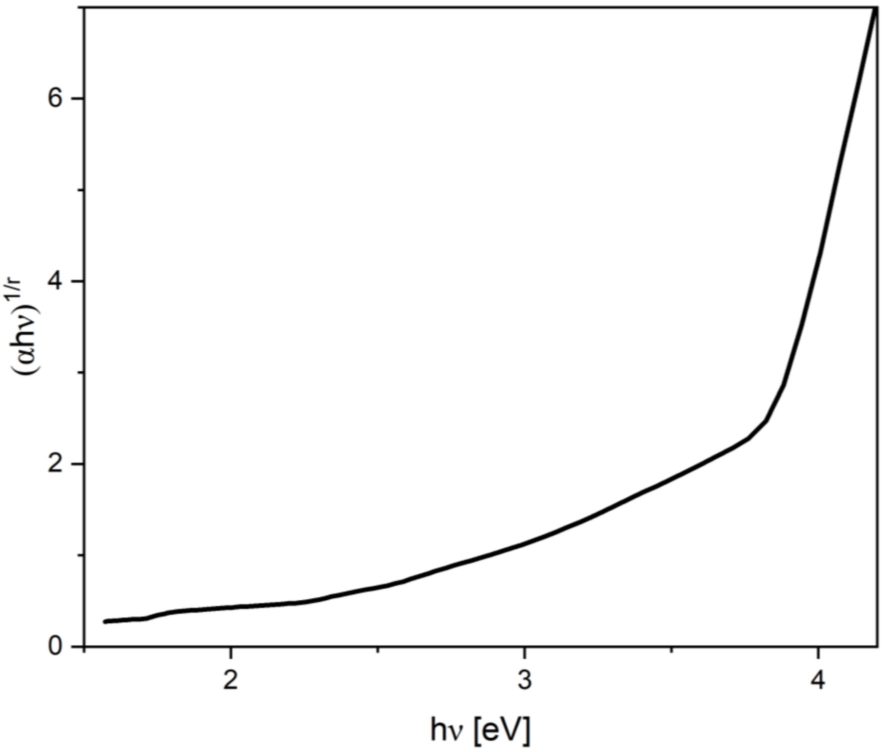


**Figure S38.** Tauc plot for $Cu_2O@(O_2CR^1)_{0.1}$ (r = 3/2).[54, 55]

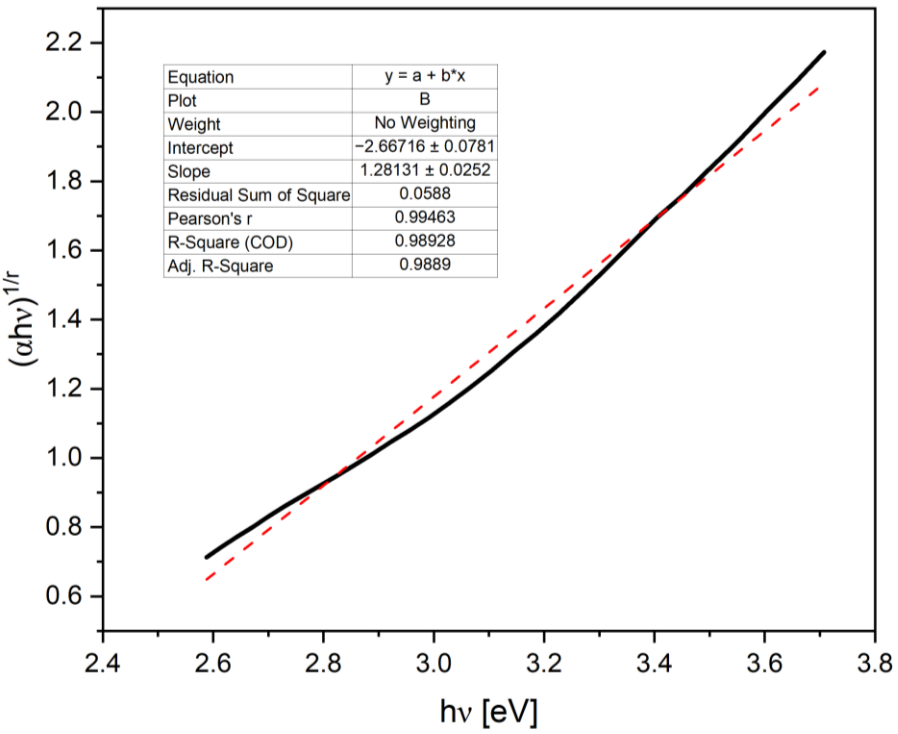


**Figure S39.** Band gap determination from the Tauc plot for $Cu_2O@(O_2CR^1)_{0.1}$ (r = 3/2); optical band-gaps have been reported as a range, taking into account errors associated with the slope and y-intercept of the line of best fit.[54, 55]

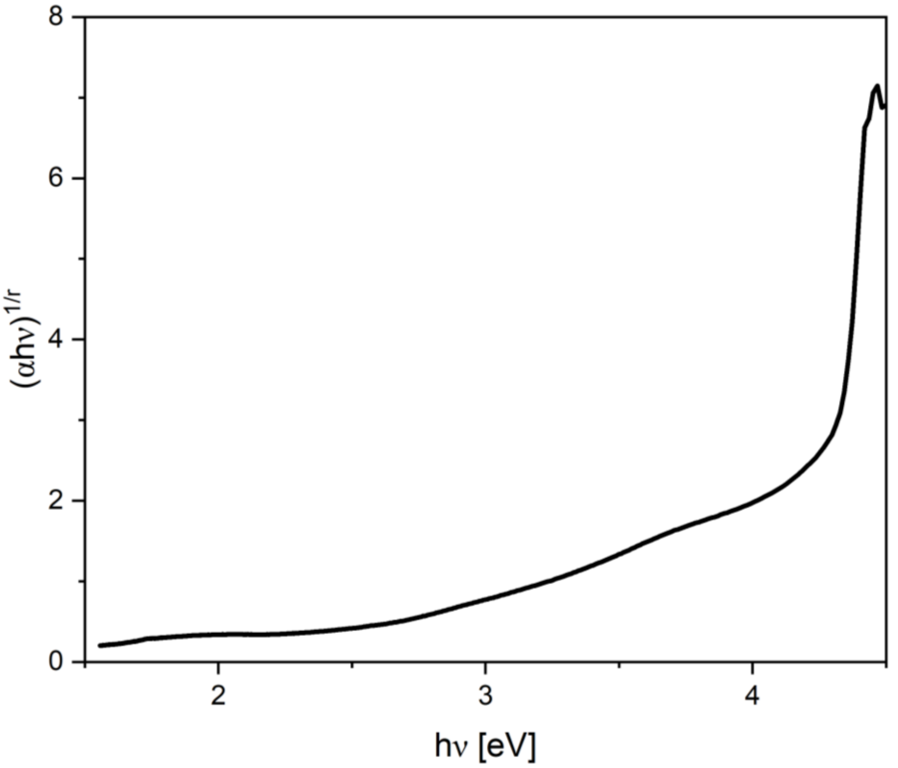


**Figure S40.** Tauc plot for $Cu_2O@(O_2CR^2)_{0.1}$ (r = 3/2).[54, 55]

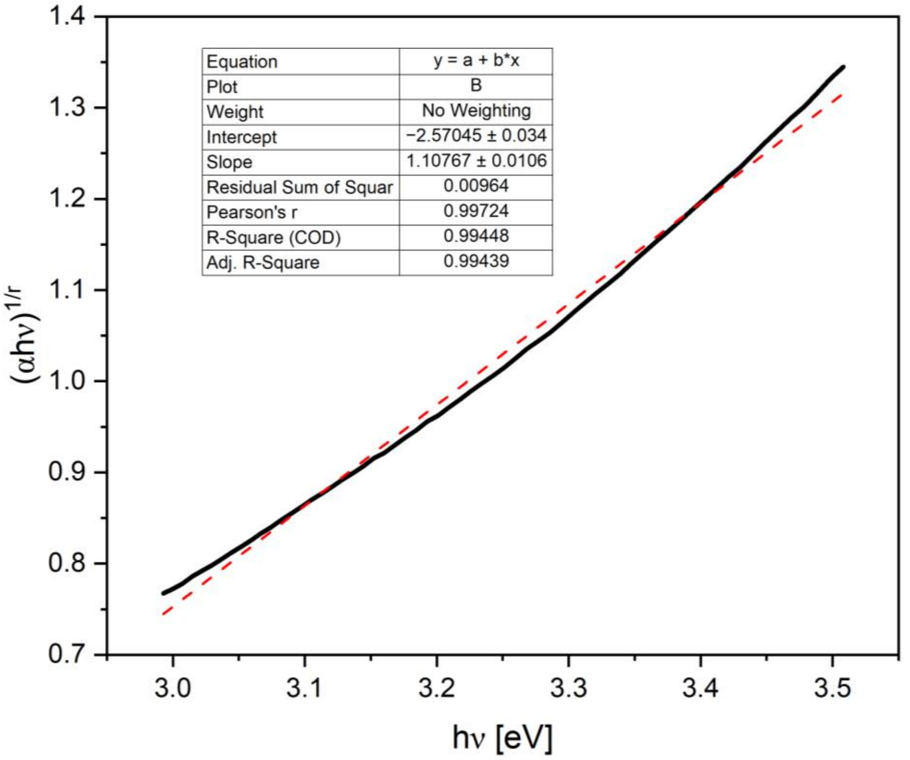


**Figure S41.** Band gap determination from the Tauc plot for $Cu_2O@(O_2CR^2)_{0.1}$ (r = 3/2); optical band-gaps have been reported as a range, taking into account errors associated with the slope and y-intercept of the line of best fit.[54, 55]

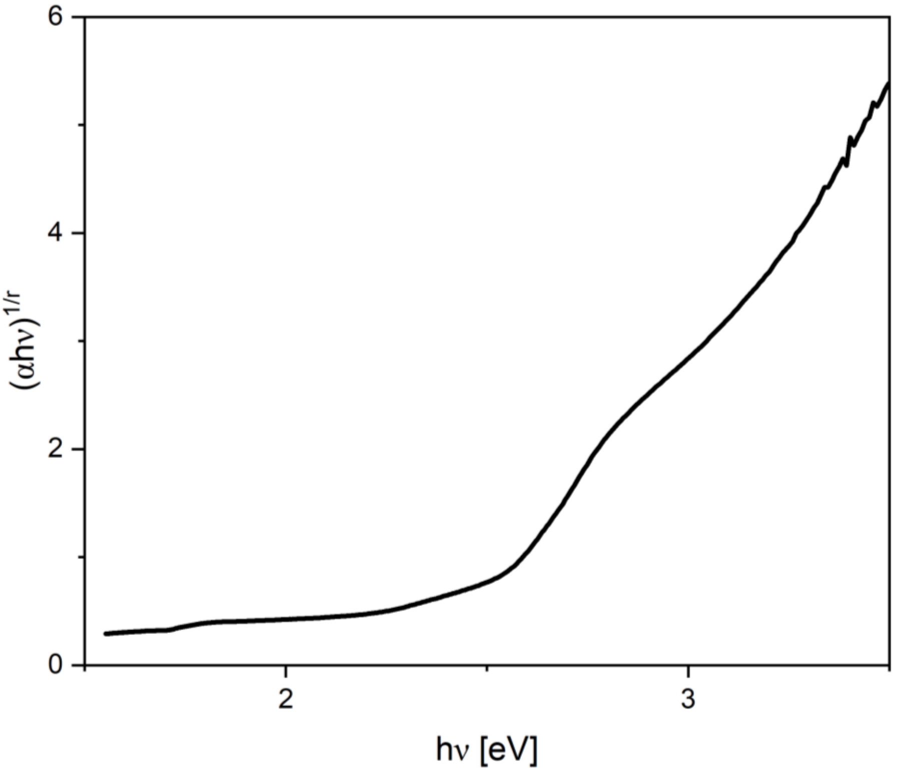


**Figure S42.** Tauc plot for $Cu_2O@(O_2CR^2)_{0.2}$ (r = 3/2).[54, 55]

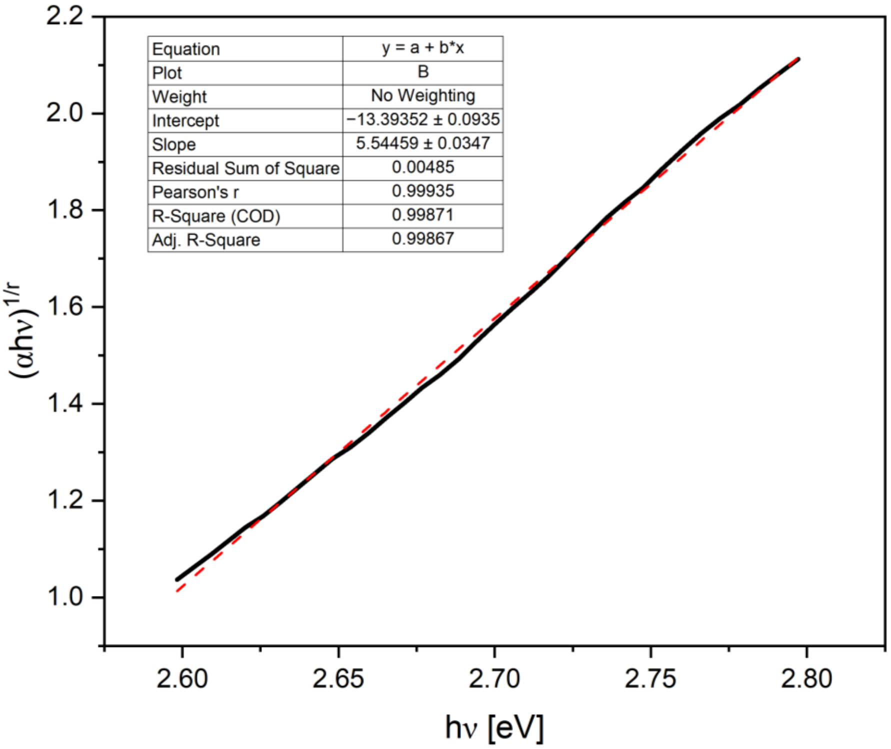


**Figure S43.** Band gap determination from the Tauc plot for $Cu_2O@(O_2CR^2)_{0.2}$ (r = 3/2); optical band-gaps have been reported as a range, taking into account errors associated with the slope and y-intercept of the line of best fit.[54, 55]

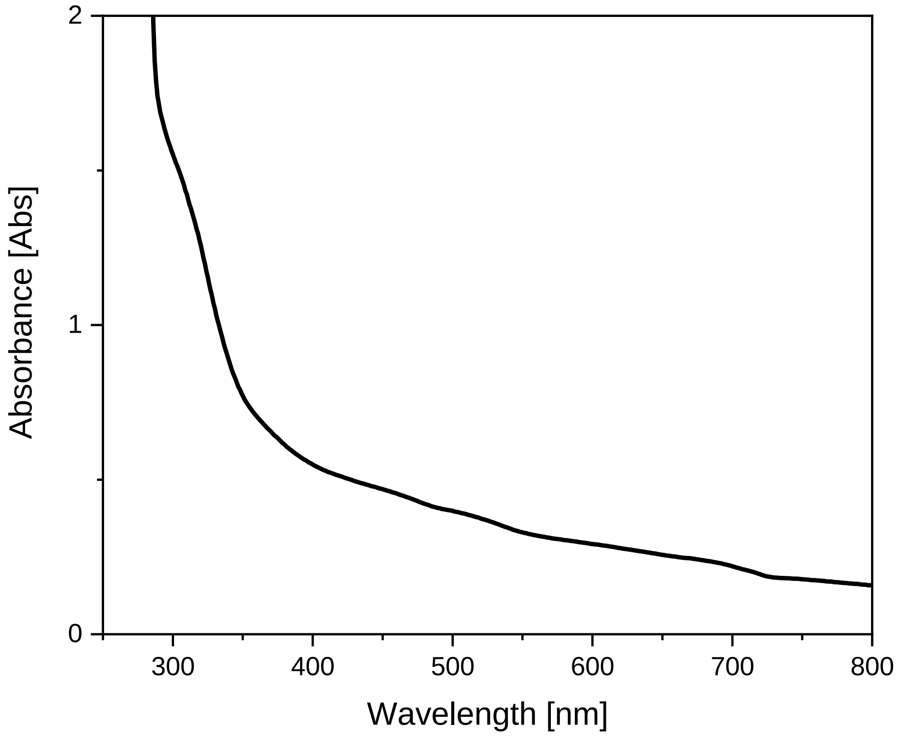


**Figure S44.** UV-Vis spectrum of $Cu_2S@(S_2CR^1)_{0.1}$ (0.3 mM solution in toluene).

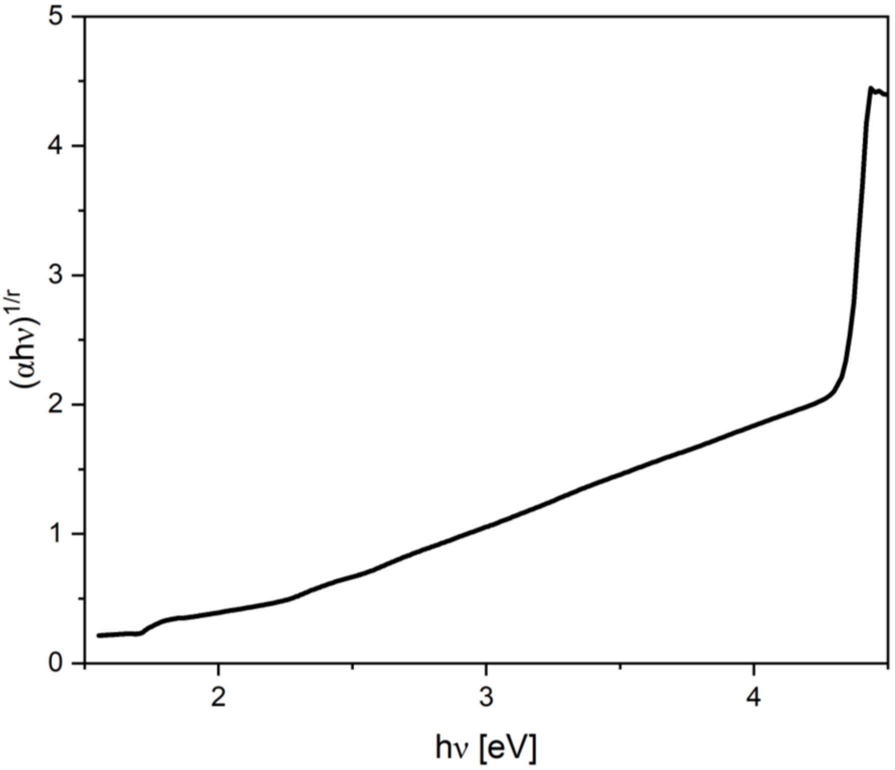


**Figure S45.** Tauc plot for $Cu_2S@(S_2CR^1)_{0.1}$ (r = 2).[56]

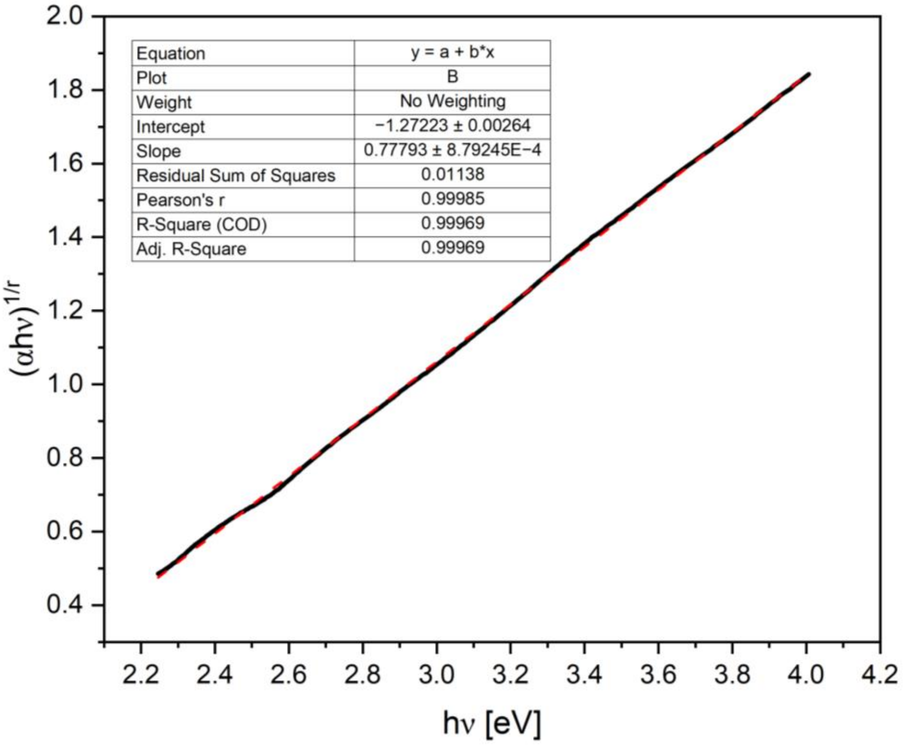


**Figure S46.** Band gap determination from the Tauc plot for $Cu_2S@(S_2CR^1)_{0.1}$ (r = 2); optical band-gaps have been reported as a range, taking into account errors associated with the slope and y-intercept of the line of best fit.[56]

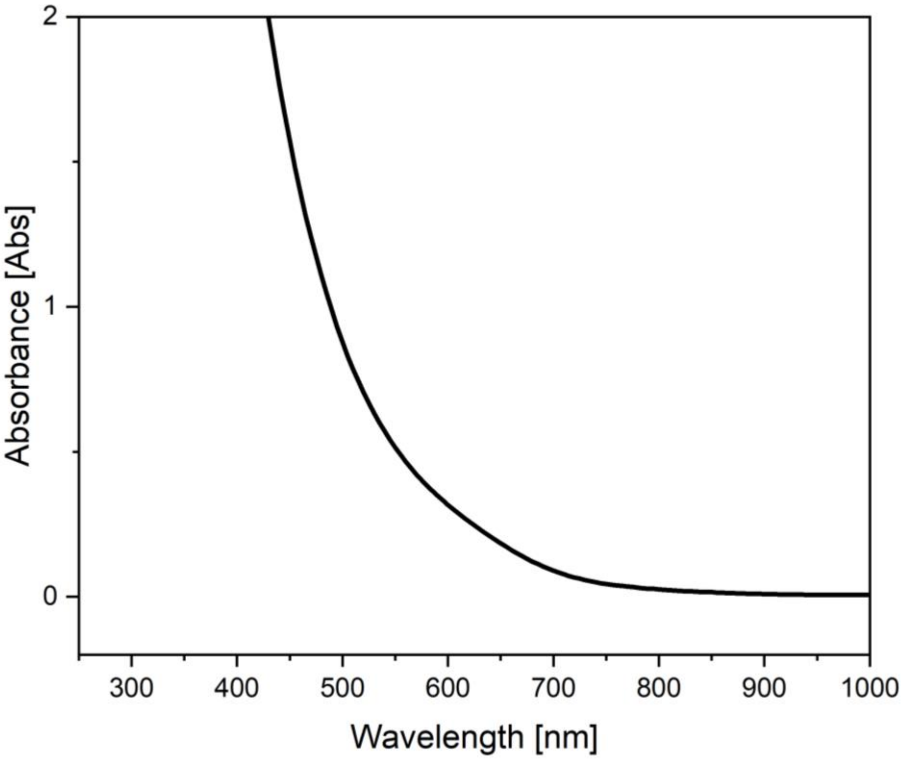


**Figure S47.** UV-Vis spectrum of $Cu_2S@(S_2CR^1)_{0.2}$ (4.5 mM solution in chloroform).

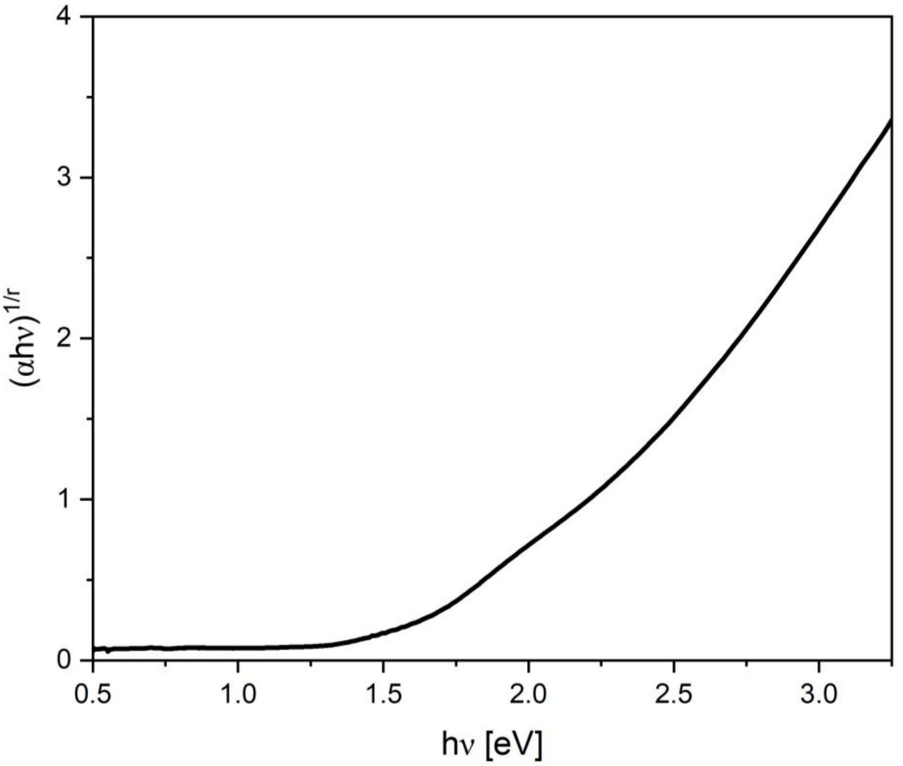


**Figure S48.** Tauc plot for $Cu_2S@(S_2CR^1)_{0.2}$ (r = 2).[56]

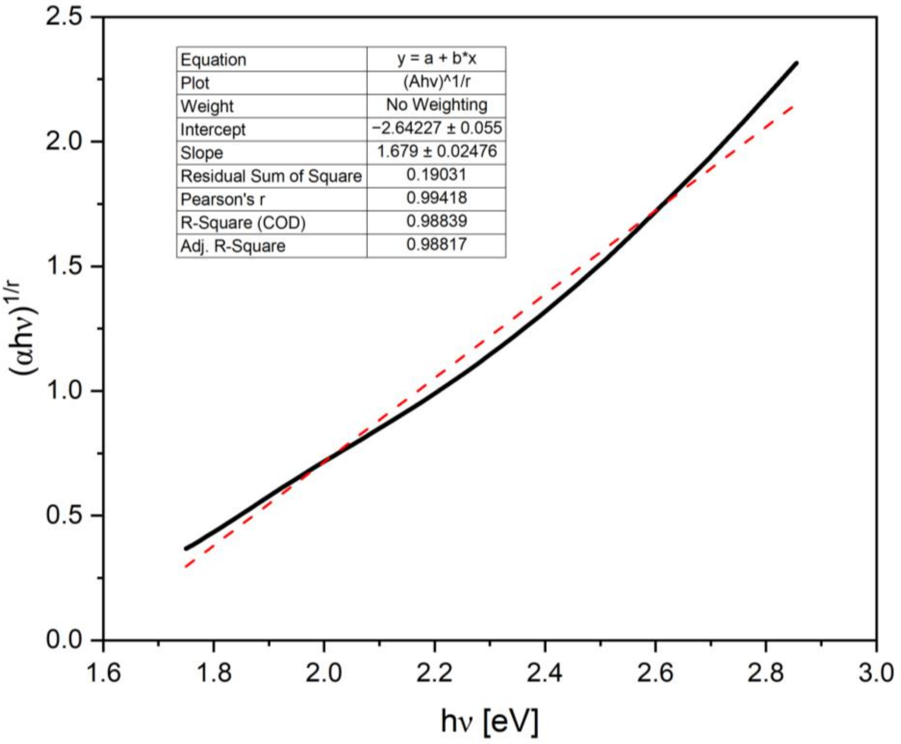


**Figure S49.** Band gap determination from the Tauc plot for $Cu_2S@(S_2CR^1)_{0.2}$ (r = 2); optical band-gaps have been reported as a range, taking into account errors associated with the slope and y-intercept of the line of best fit.[56]

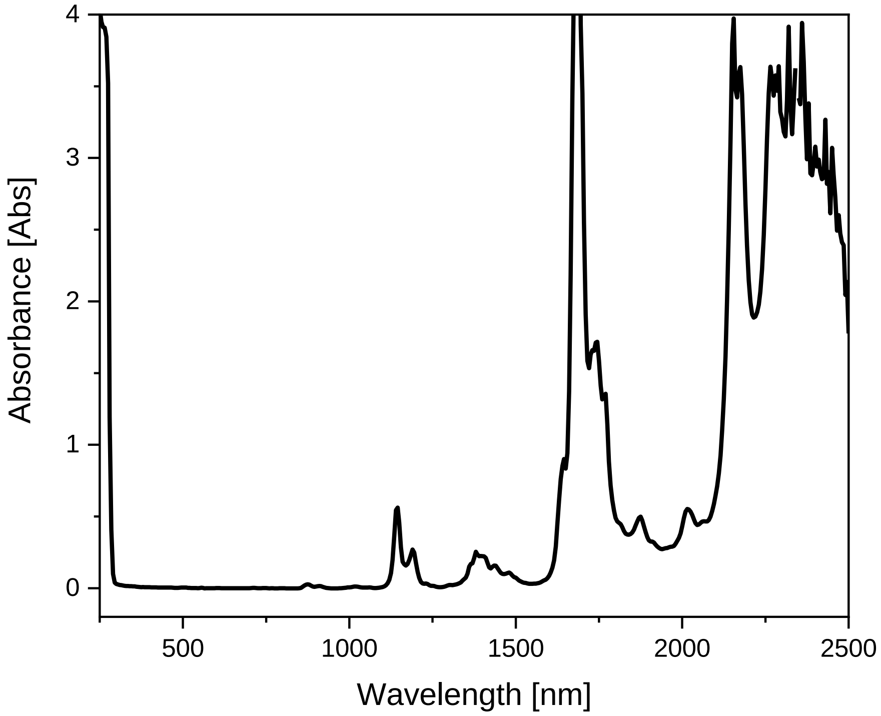


**Figure S50.** UV-Vis-NIR spectrum of toluene.

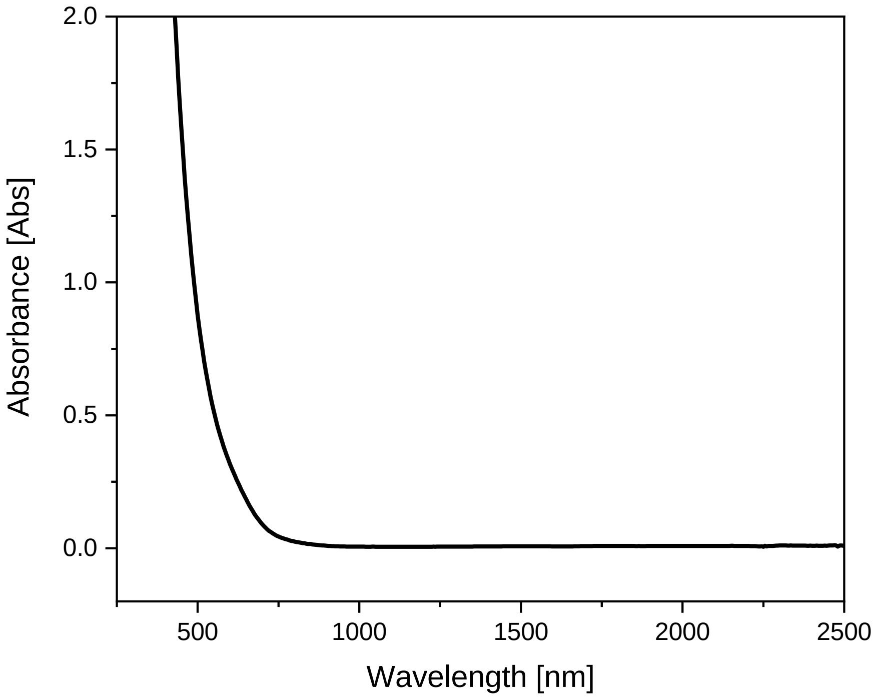


**Figure S51.** UV-Vis-NIR spectrum of $Cu_2S@(S_2CR^1)_{0.2}$ (4.5 mM solution in chloroform).

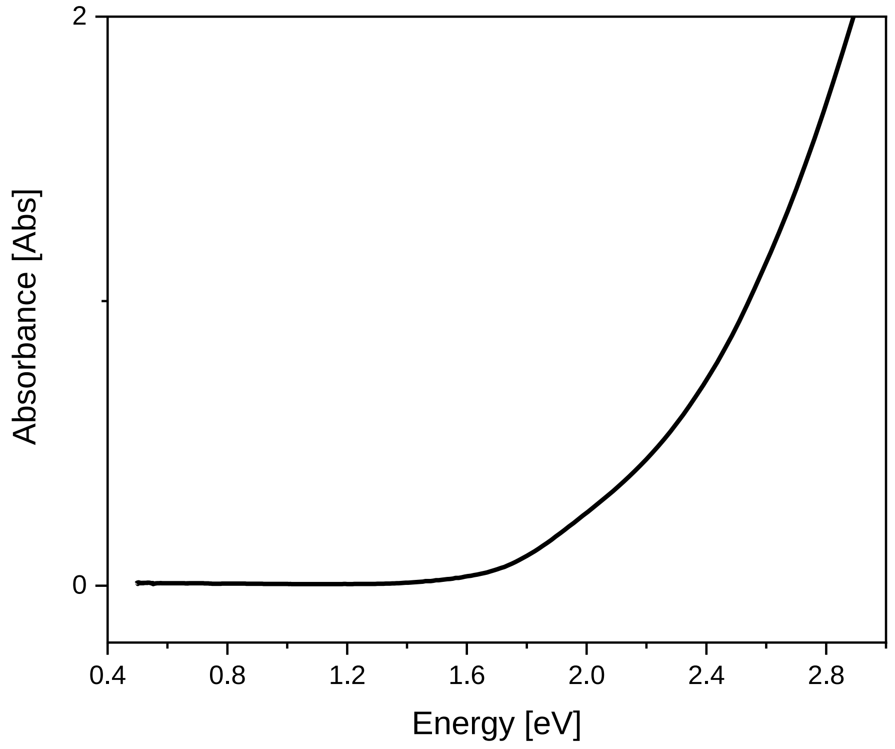


**Figure S52.** UV-Vis-NIR spectrum of $Cu_2S@(S_2CR^1)_{0.2}$ (4.5 mM solution in chloroform).

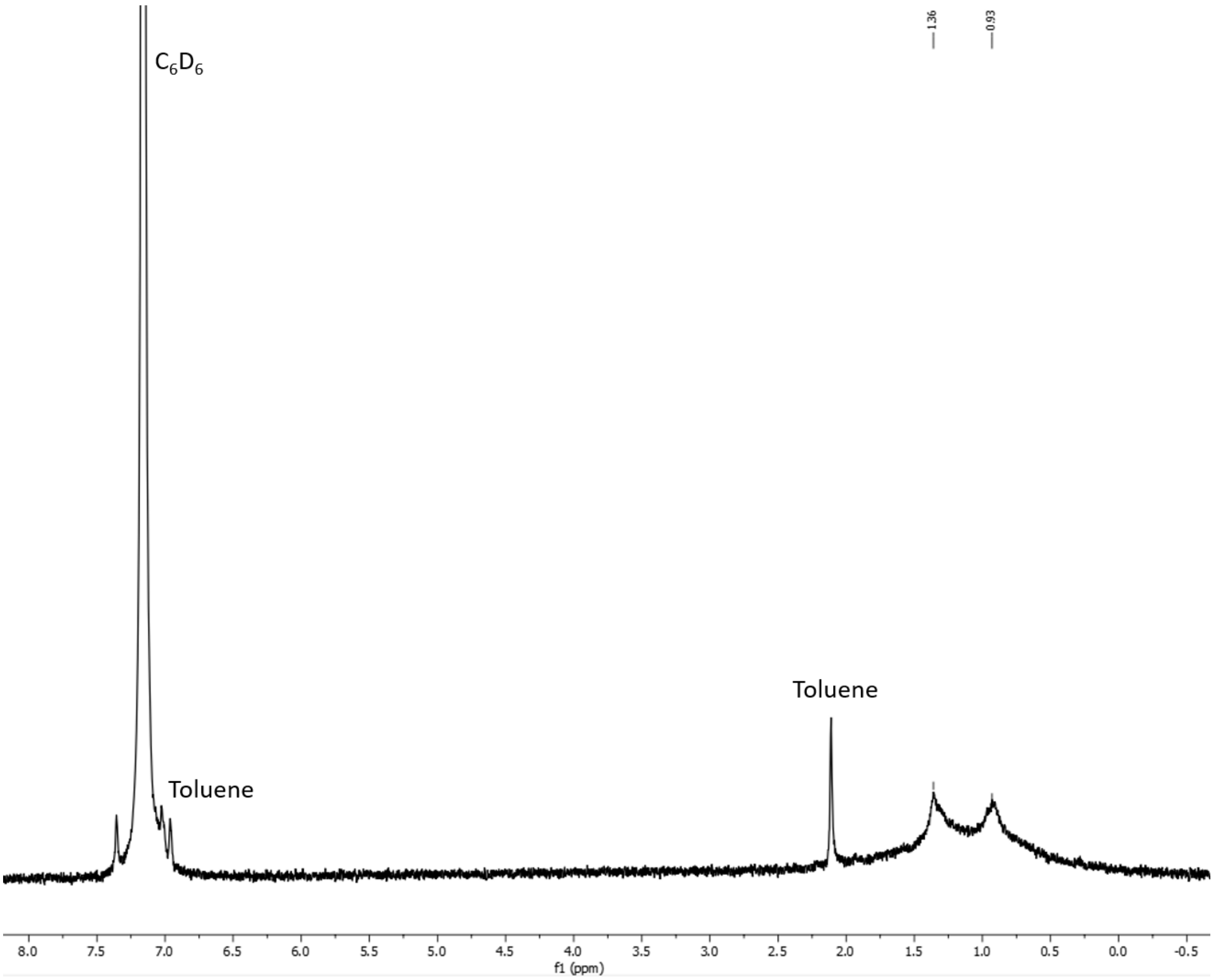


**Figure S53.** $^1$H NMR spectrum of $Cu_2O@(O_2CR^1)_{0.1}$ ($C_6D_6$, 400 MHz, 20 °C).

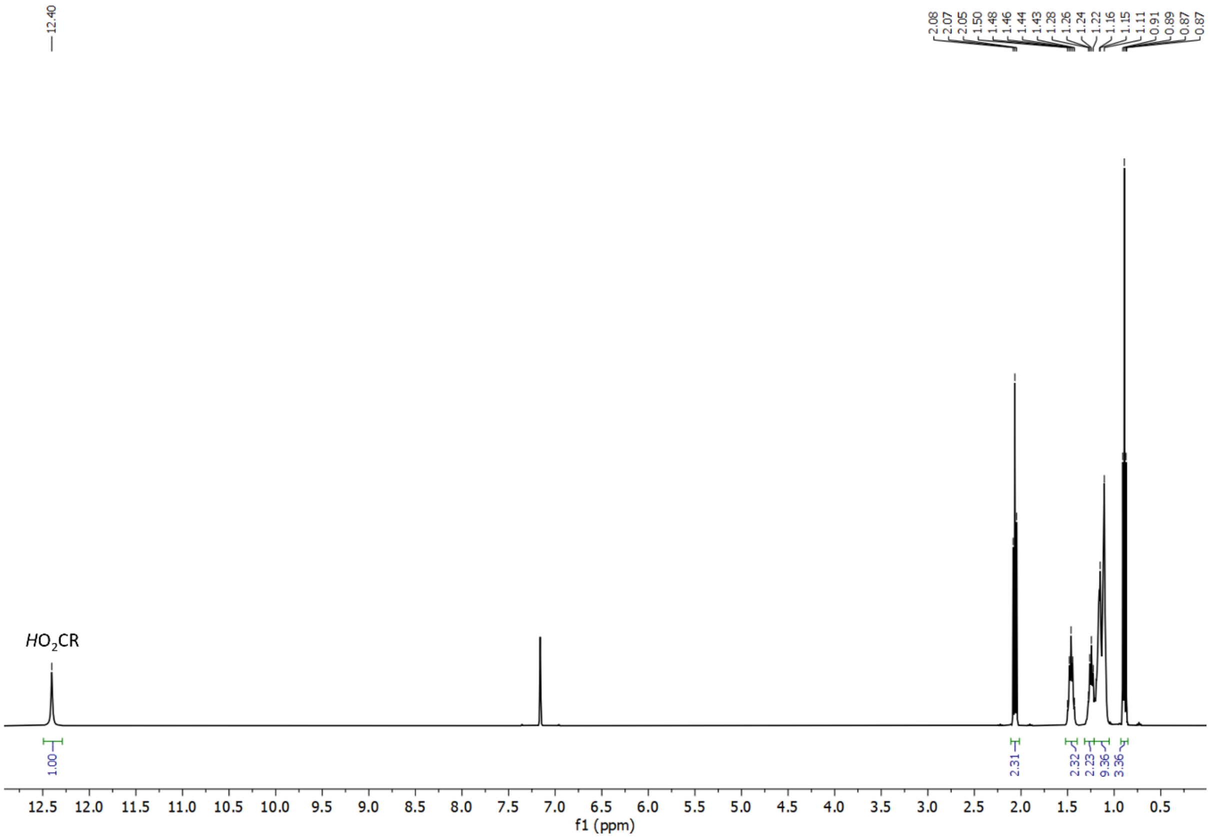


**Figure S54.** $^1H$ NMR spectrum of $HO_2CR^1$ ($C_6D_6$, 400 MHz, 20 °C).

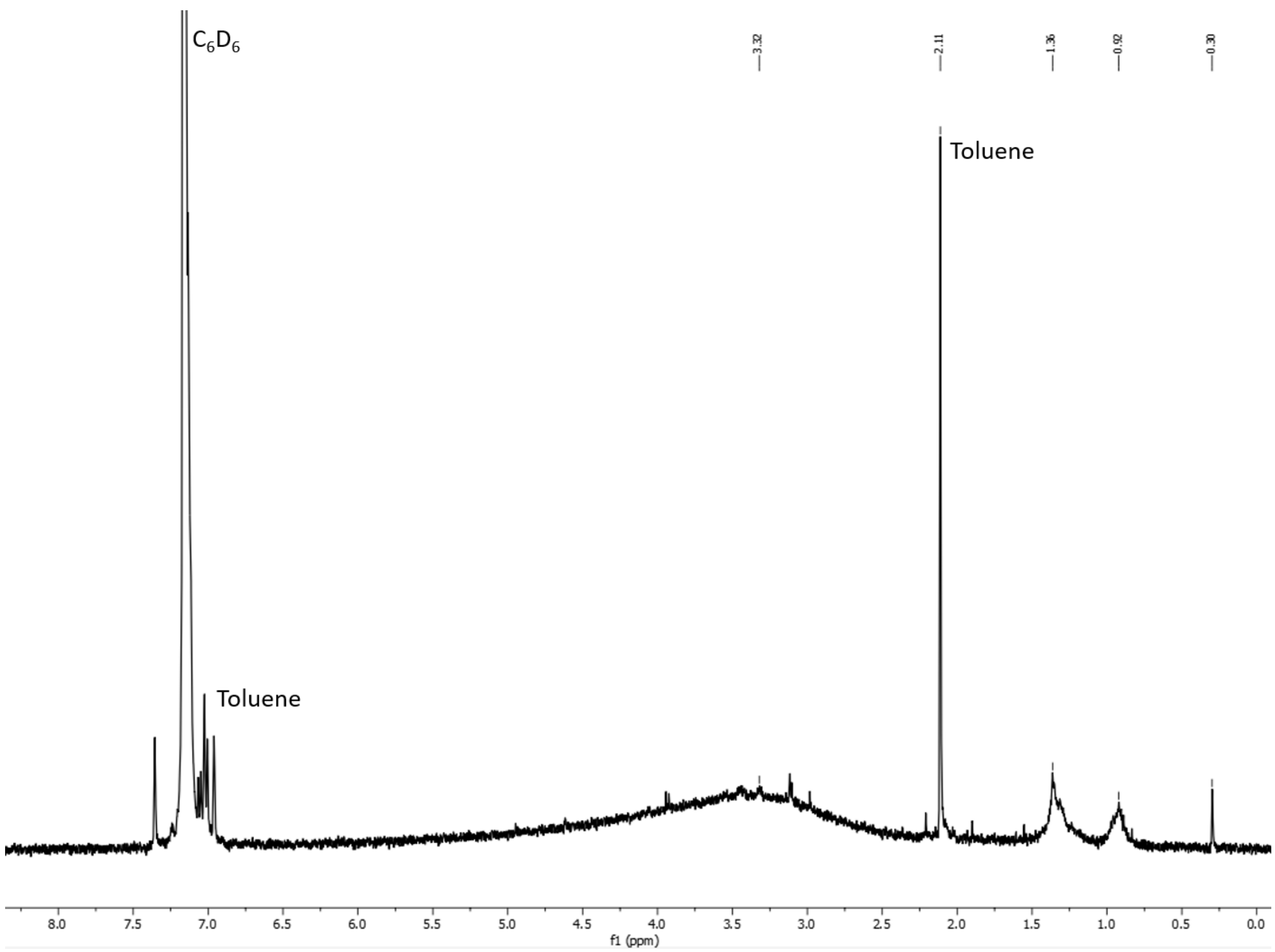


**Figure S55.** $^1$H NMR spectrum of $Cu_2O@(O_2CR^2)_{0.1}$ ($C_6D_6$, 400 MHz, 20 °C).

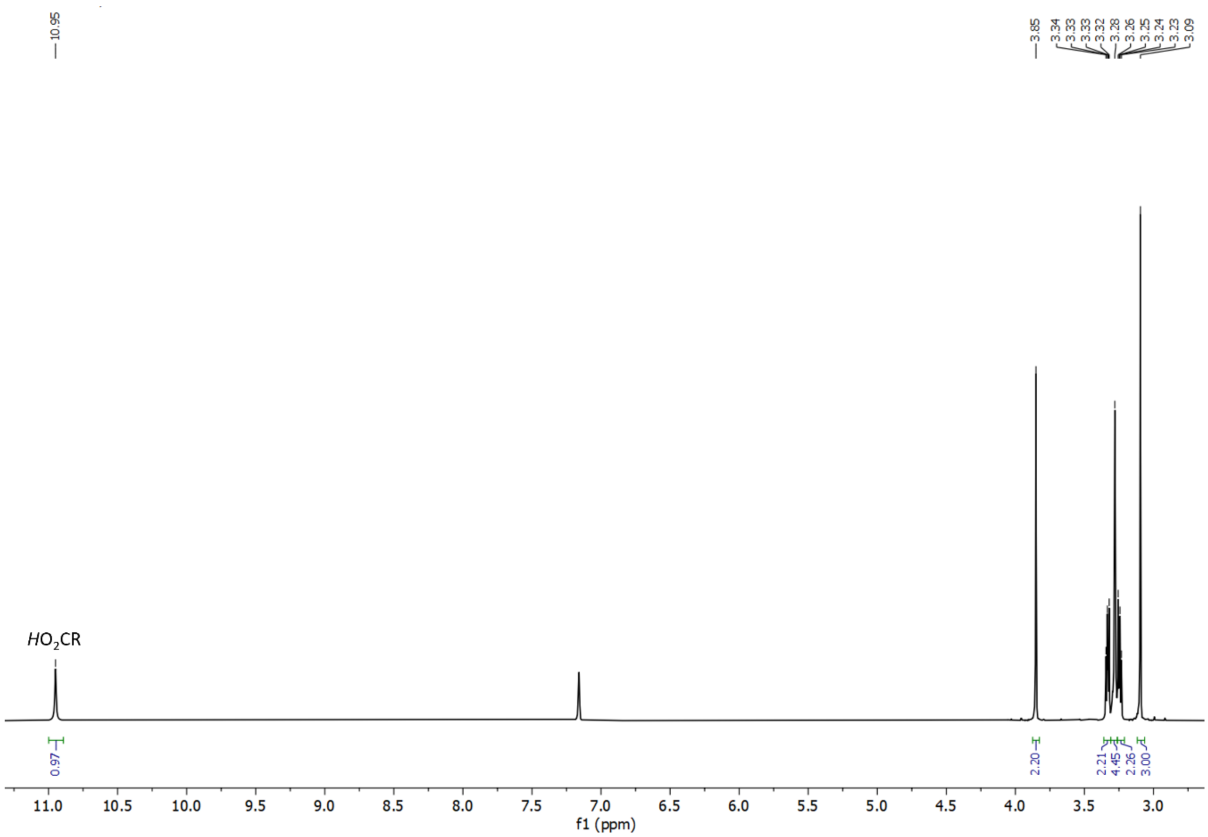


**Figure S56.** $^{1}$H NMR spectrum of $HO_2CR^2$ ($C_6D_6$, 400 MHz, 20 °C).

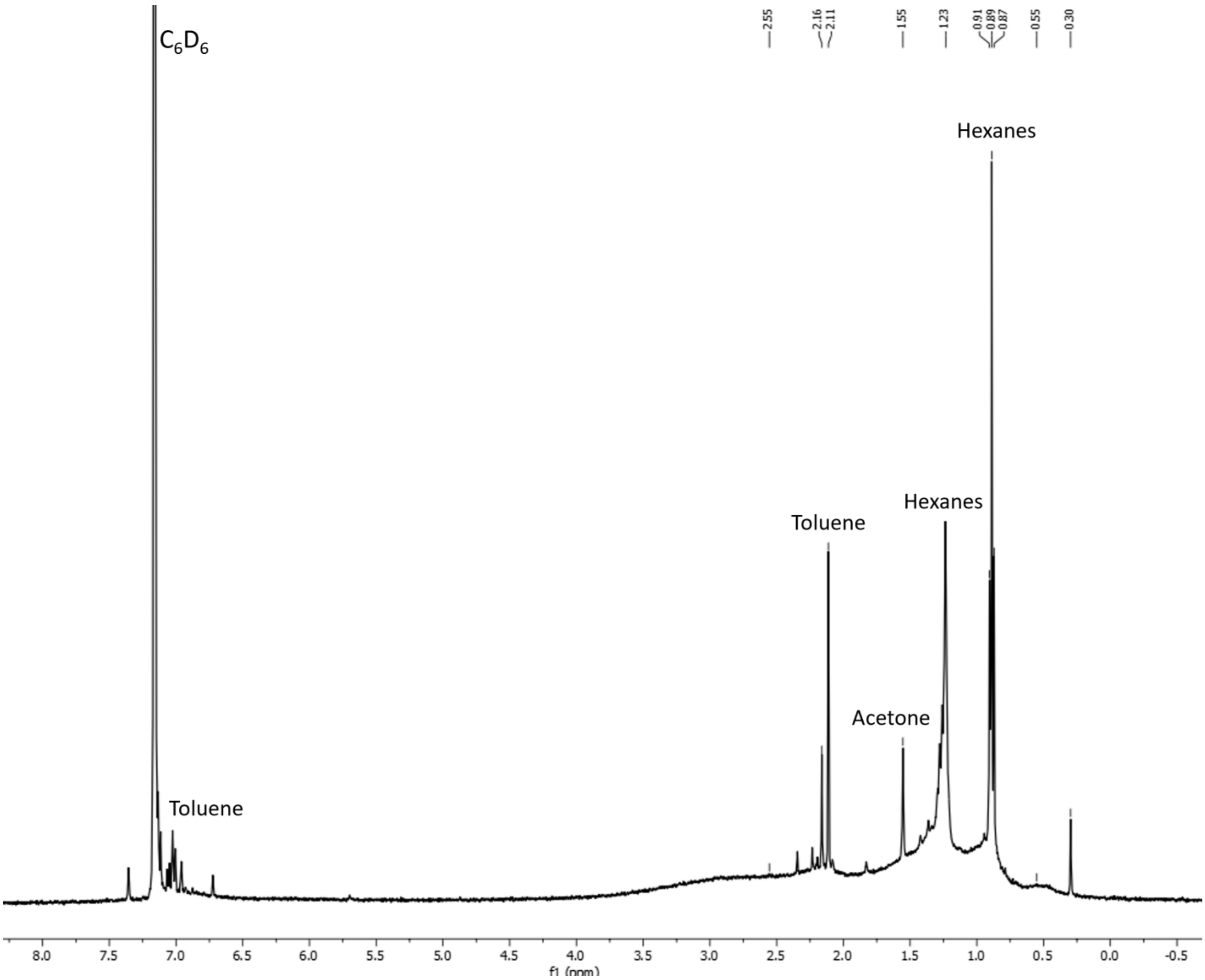


**Figure S57.** $^{1}H$ NMR spectrum of $Cu_2S@(S_2CR^1)_{0.1}$ ($C_6D_6$, 400 MHz, 20 °C).

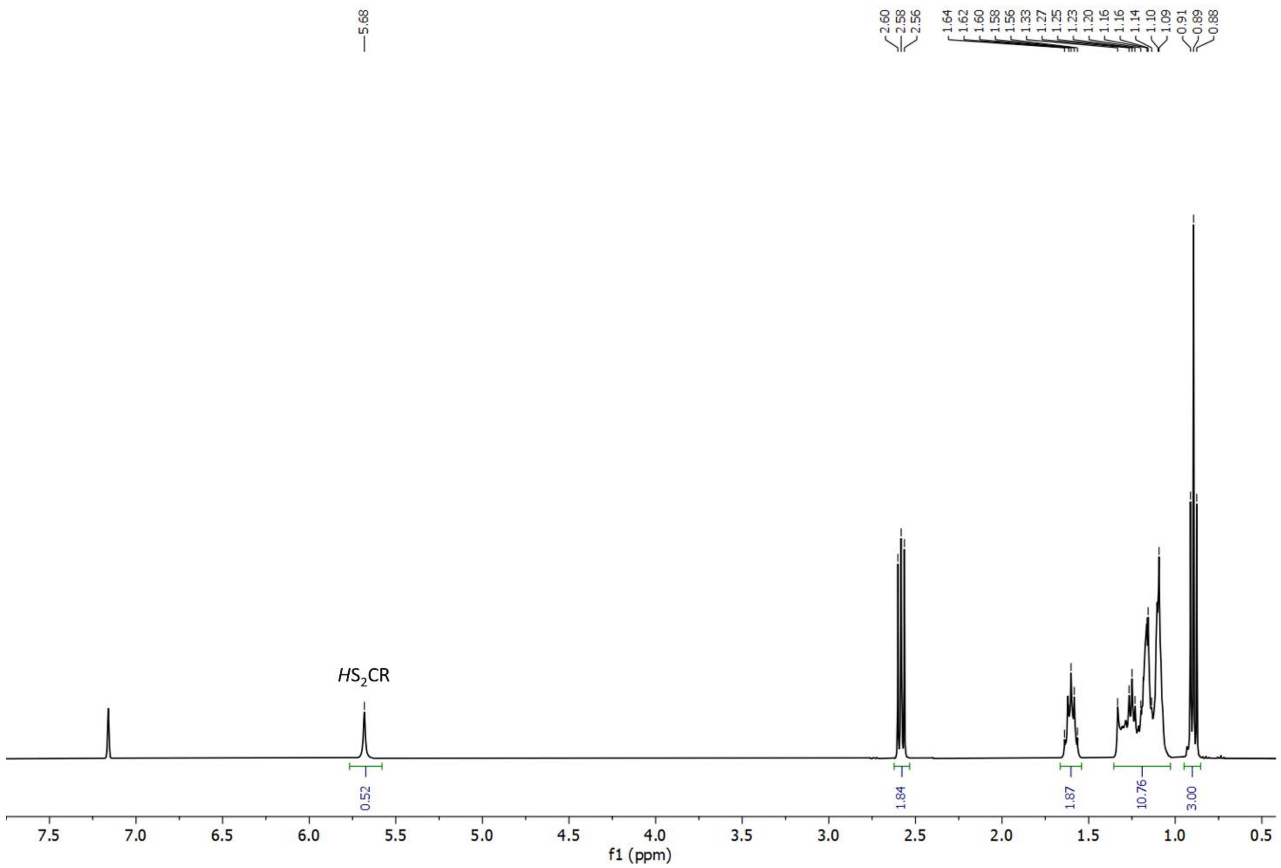


**Figure S58.** $^{1}H$ NMR spectrum of $HS_2CR^1$ ($C_6D_6$, 400 MHz, 20 °C).

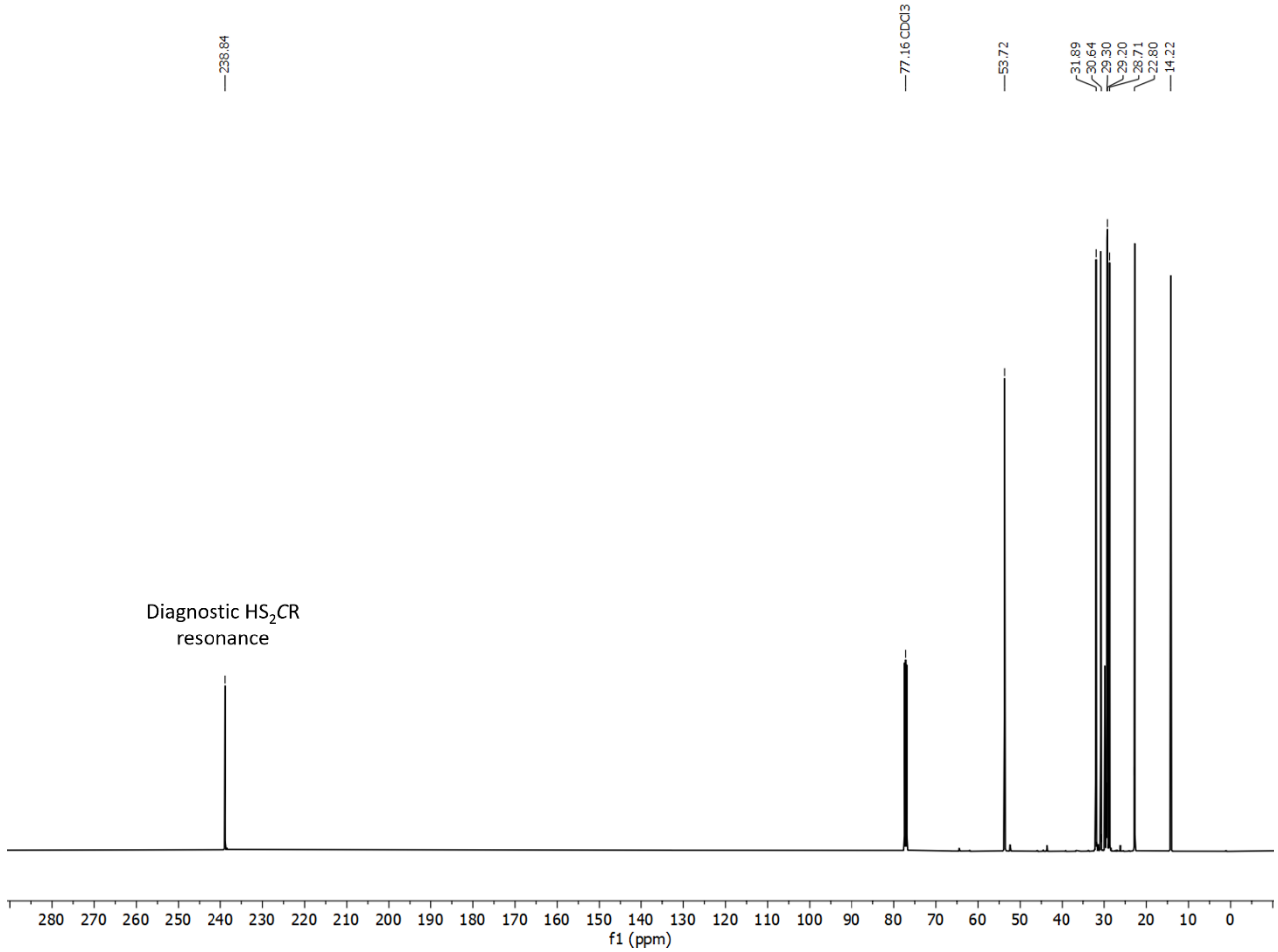


**Figure S59.** $^{13}C\{^{1}H\}$ NMR spectrum of $HS_2CR^1$ ($C_6D_6$, 101 MHz, 20 °C).

**Table S3.** Comparison of key stretching bands observed by FT-IR spectroscopy.

| **Ligand** | **ν(O-H) [cm⁻¹]** | **ν(C=O) [cm⁻¹]** | **ν(S-H) [cm⁻¹]** | **ν(C=S) [cm⁻¹]** |
|---|---|---|---|---|
| $HO_2CR^1$ | 2930 [a] | 1706 | – | – |
| $HO_2CR^2$ | 3063 | 1758, 1736 | – | – |
| $HS_2CR^1$ | – | – | 2490 | 1211 |
| | | | | |
| **Nanoparticle** | **$\nu(CO_2)^-_{asym}$ [cm⁻¹]** | **$\nu(CO_2)^-_{sym}$ [cm⁻¹]** | **$\nu(CS_2)^-_{asym}$ [cm⁻¹]** | **$\nu(CS_2)^-_{sym}$ [cm⁻¹]** |
| $Cu@(O_2CR^1)_{0.1}$ | 1421, 1410 | – | – | – |
| $Cu@(O_2CR^2)_{0.1}$ | 1427, 1408 | 1325 | – | – |
| $Cu@(S_2CR^1)_{0.1}$ | – | – | 1044, 1011 | 848 |
| $Cu_2O@(O_2CR^1)_{0.1}$ | 1547 | 1430 | – | – |
| $Cu_2O@(O_2CR^2)_{0.1}$ | 1588 | 1438, 1415 | – | – |
| $Cu_2O@(O_2CR^2)_{0.2}$ | 1584 | 1438, 1415 | – | – |
| $Cu_2S@(S_2CR^1)_{0.1}$ | – | – | 1044, 1027, 1011 | 848 |
| $Cu_2S@(S_2CR^1)_{0.2}$ | – | – | 1044, 1030, 1012 | 846 |

[a] Overlapping ν(O-H) with ν(C-H).

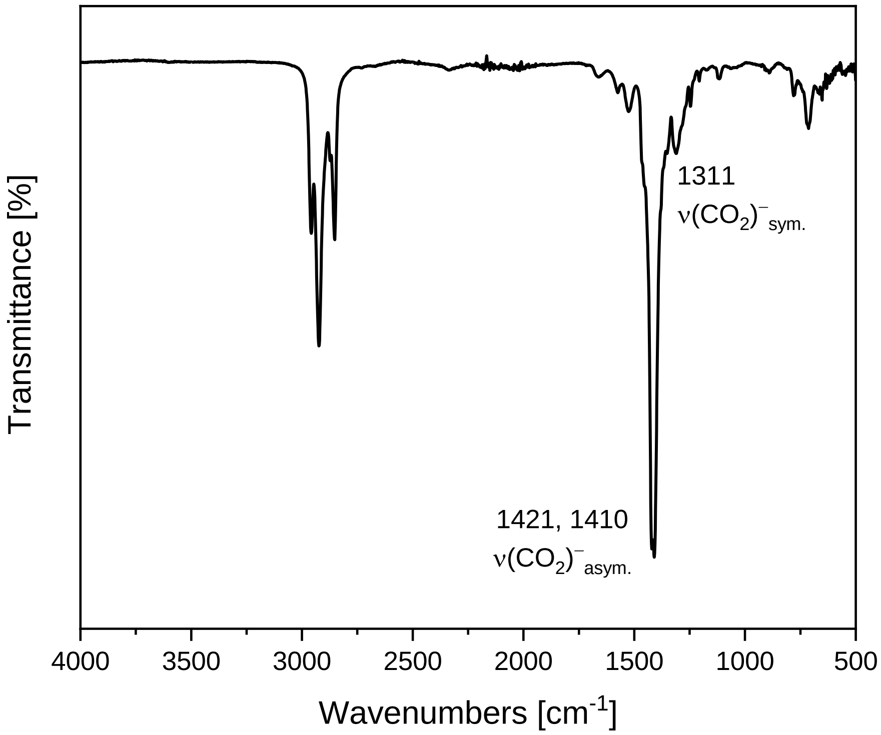


**Figure S60.** FT-IR spectrum of $Cu@(O_2CR^1)_{0.1}$.

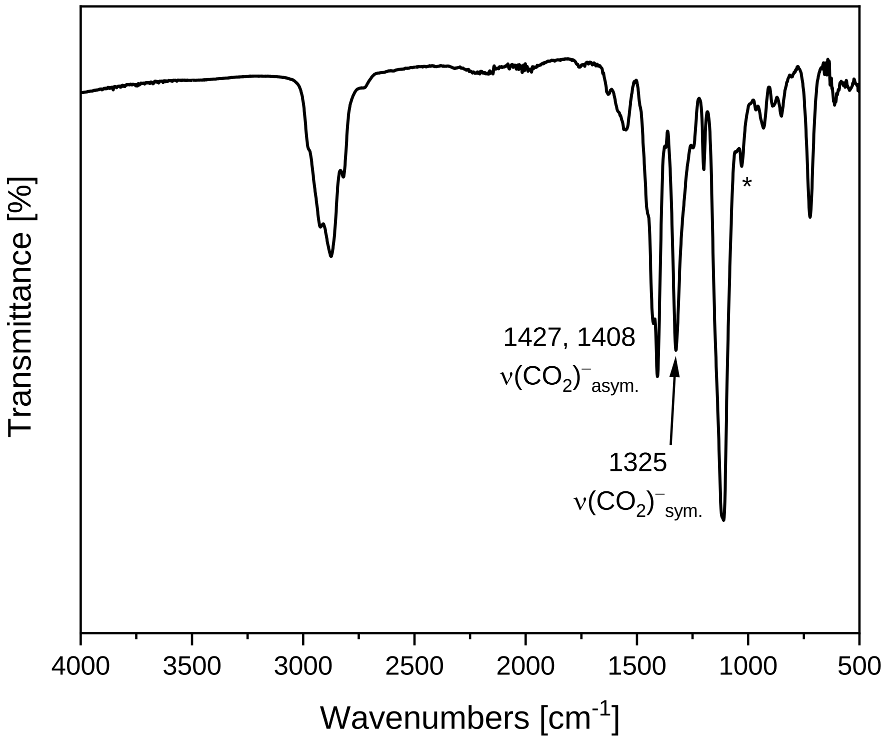


**Figure S61.** FT-IR spectrum of Cu@$(O_2CR^2)_{0.1}$; * = mesitylene.

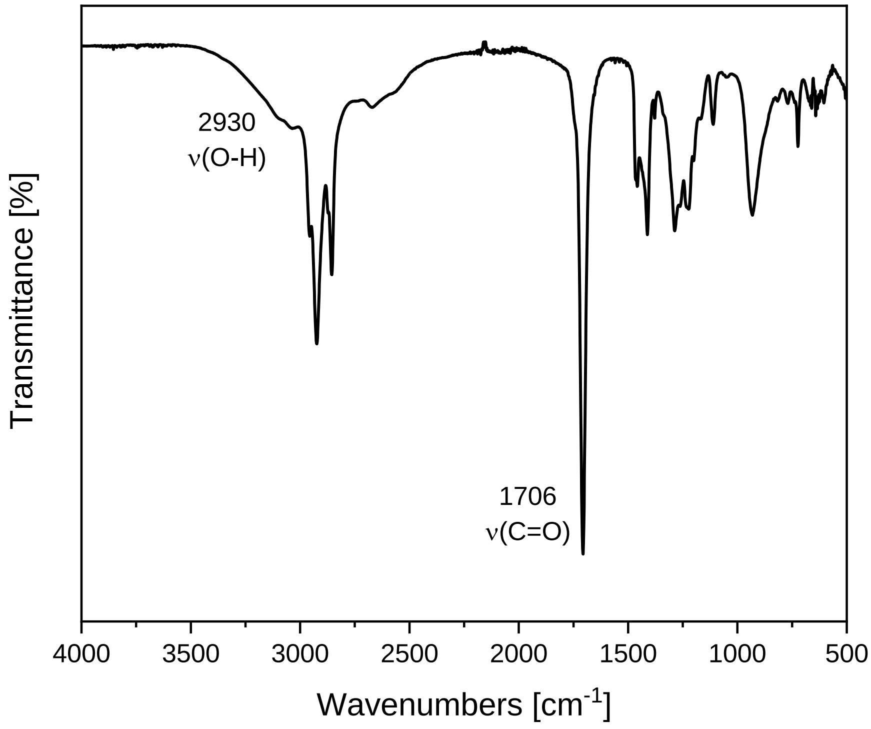


**Figure S62.** FT-IR spectrum of $HO_2CR^1$.

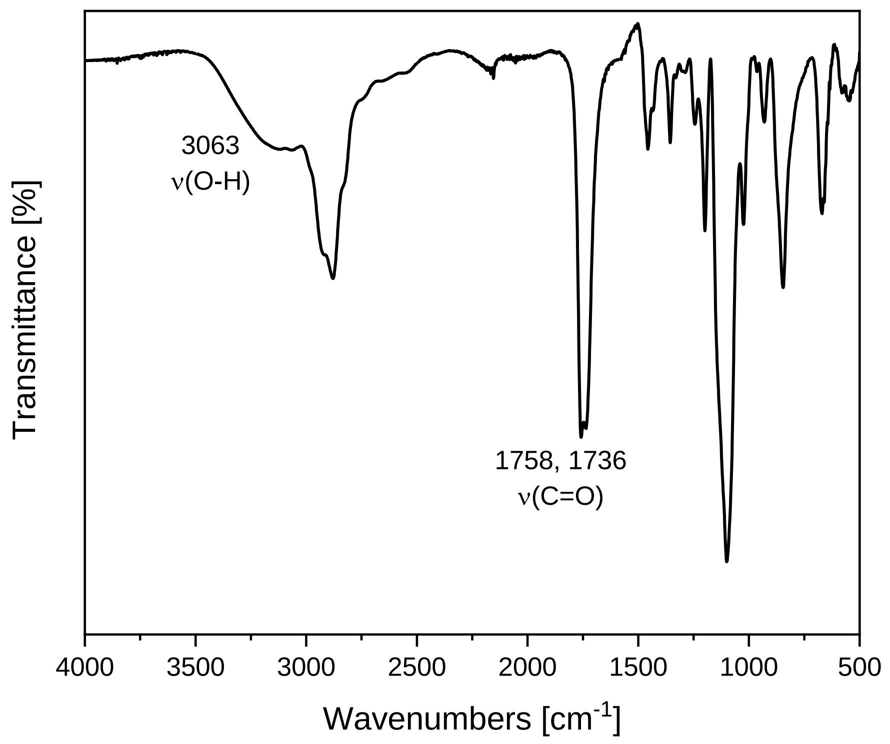


**Figure S63.** FT-IR spectrum of $HO_2CR^2$.

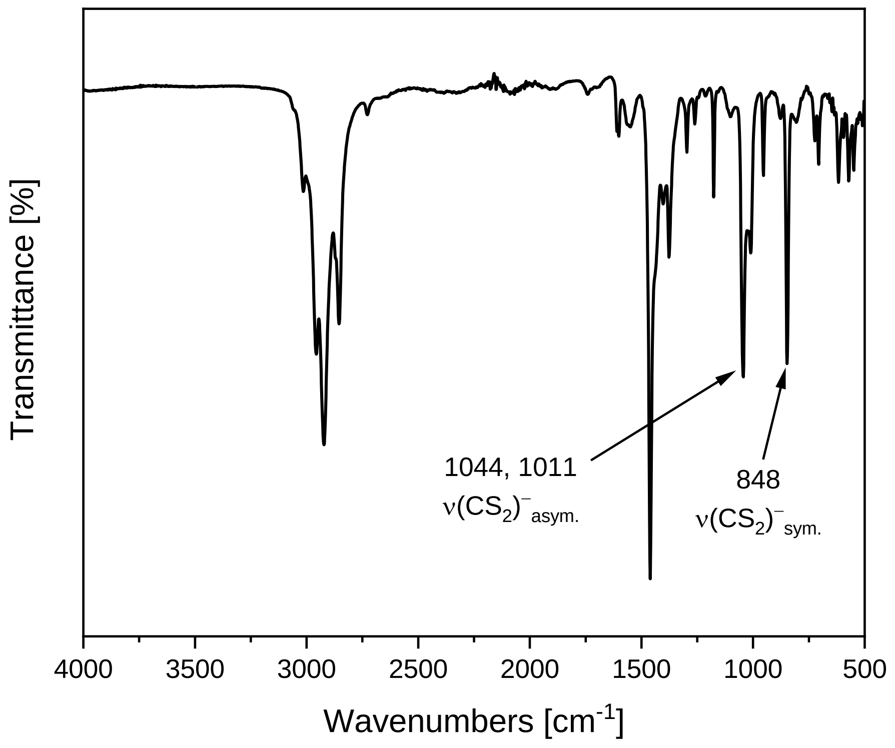


**Figure S64.** FT-IR spectrum of $Cu@(S_2CR^1)_{0.1}$.

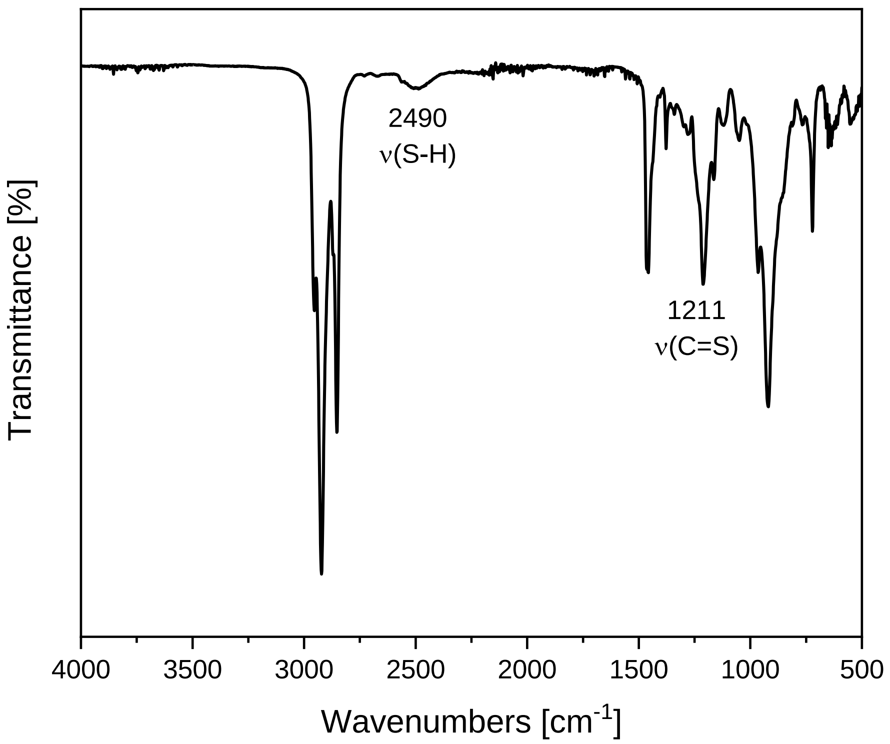


**Figure S65.** FT-IR spectrum of $HS_2CR^1$.

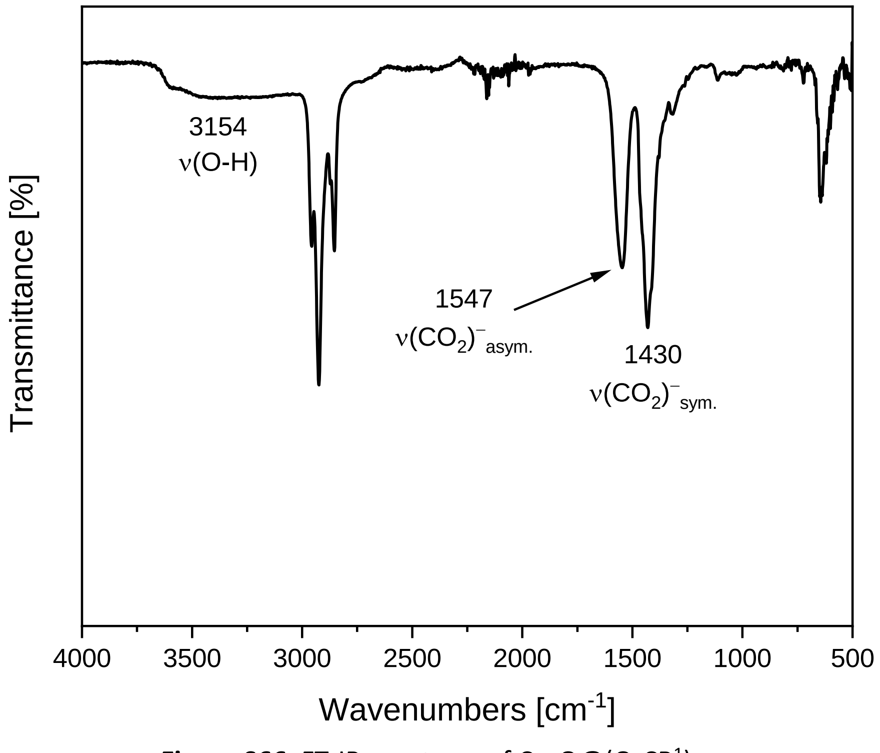


**Figure S66.** FT-IR spectrum of $Cu_2O@(O_2CR^1)_{0.1}$.

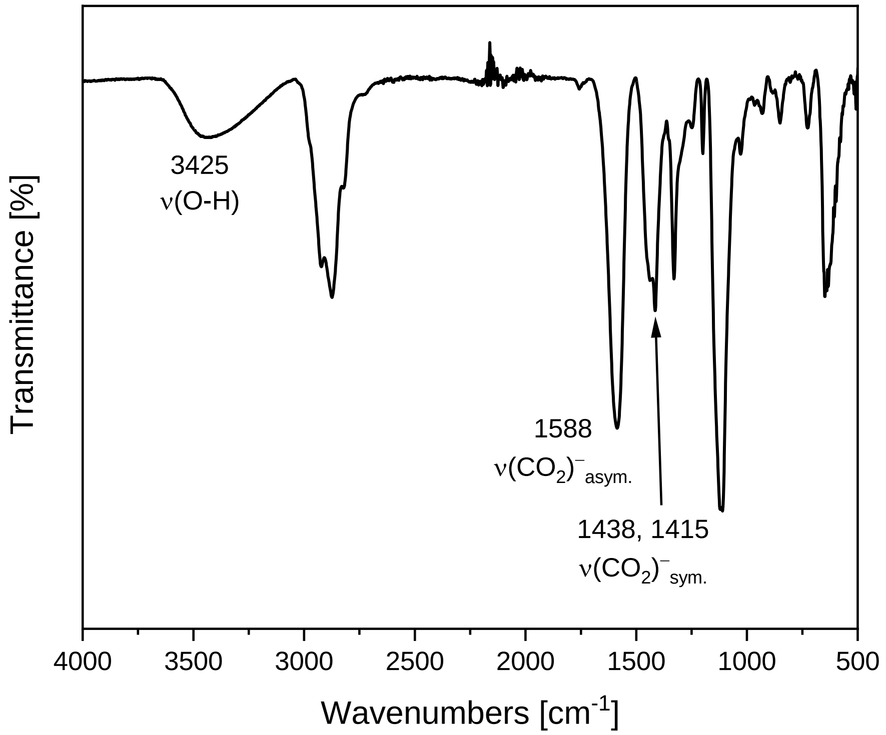


**Figure S67.** FT-IR spectrum of $Cu_2O@(O_2CR^2)_{0.1}$.

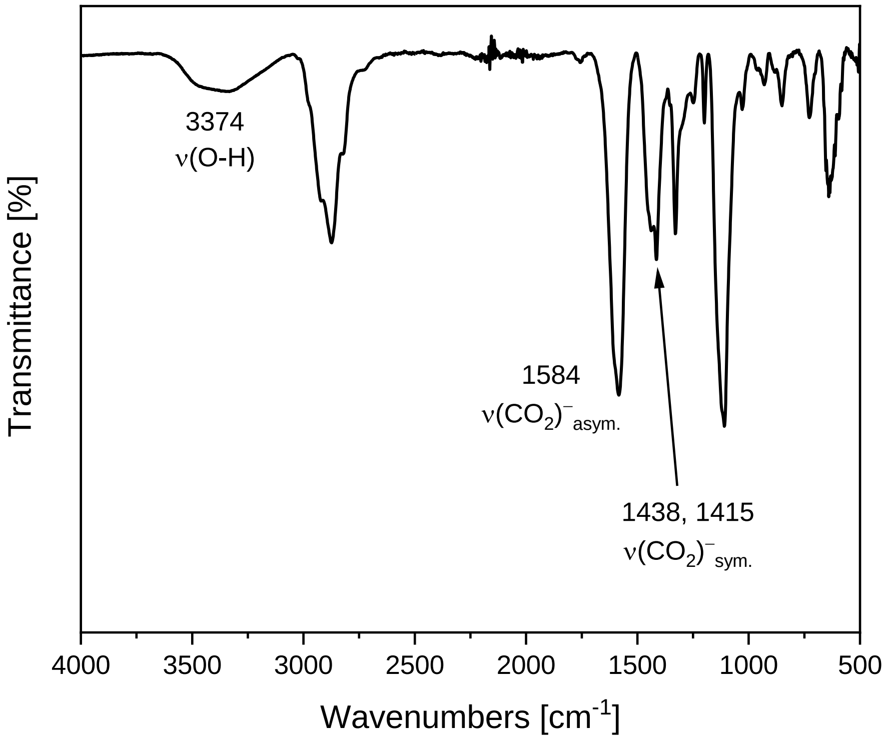


**Figure S68.** FT-IR spectrum of $Cu_2O@(O_2CR^2)_{0.2}$.

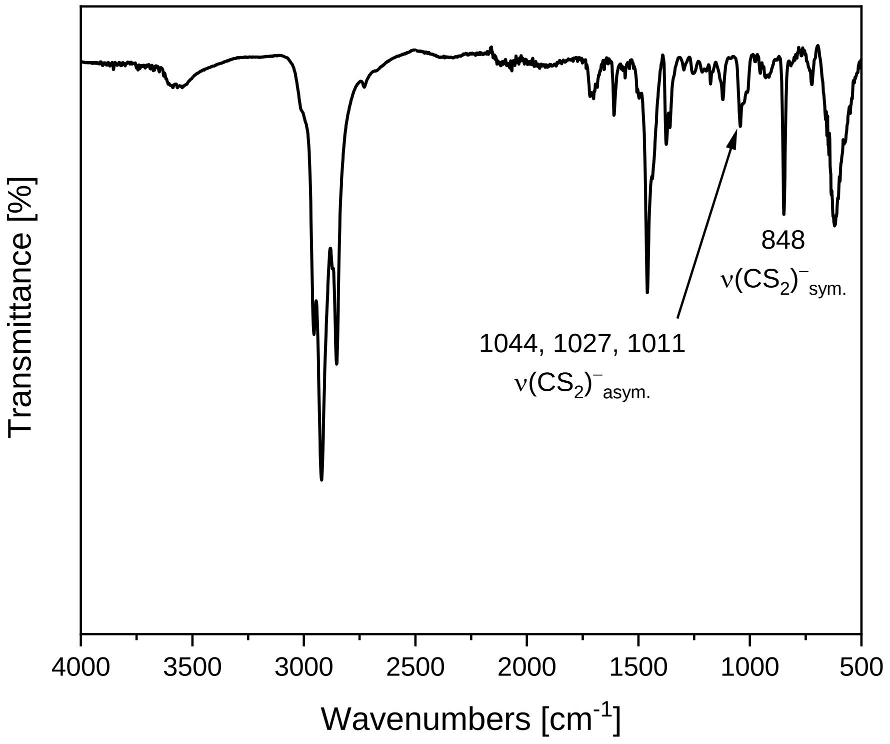


**Figure S69.** FT-IR spectrum of $Cu_2S@(S_2CR^1)_{0.1}$ when synthesized using $H_2S$.

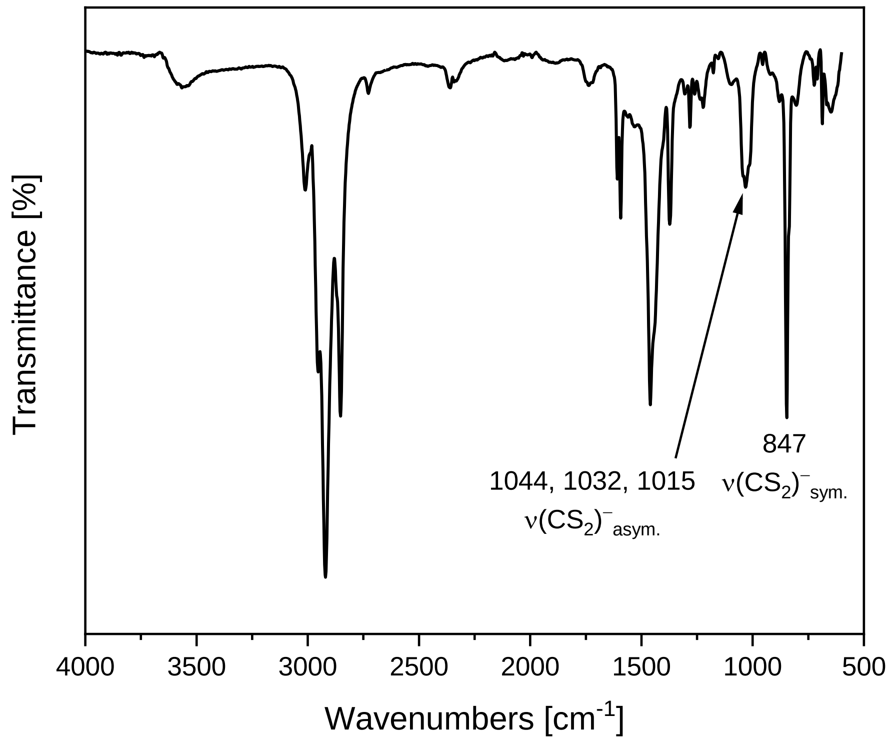


**Figure S70.** FT-IR spectrum of $Cu_2S@(S_2CR^1)_{0.1}$ when synthesized using $S(SiMe_3)_2$.

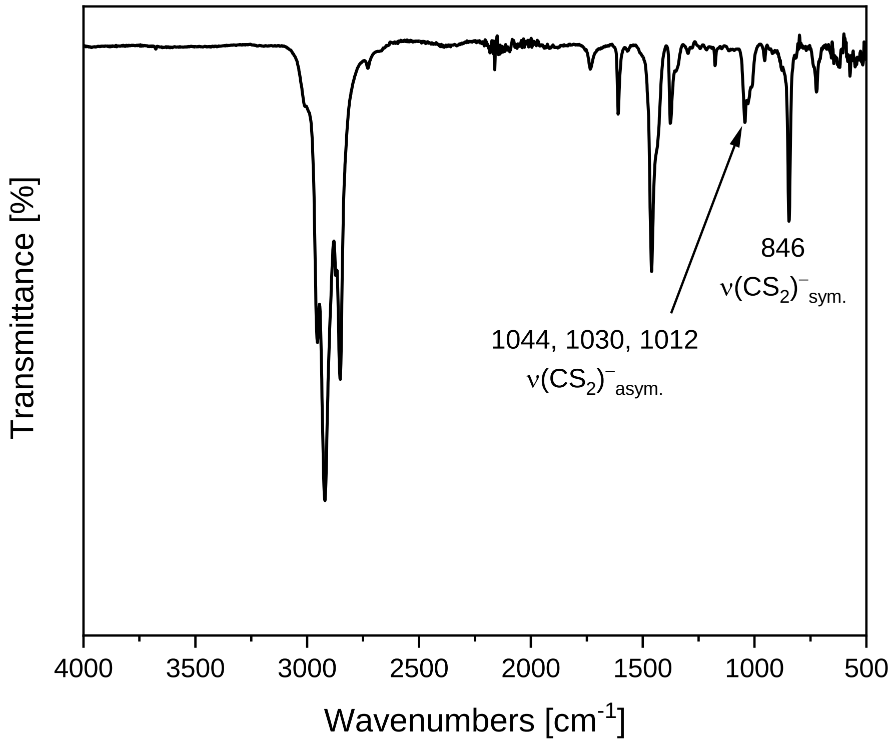


**Figure S71.** FT-IR spectrum of $Cu_2S@(S_2CR^1)_{0.2}$.

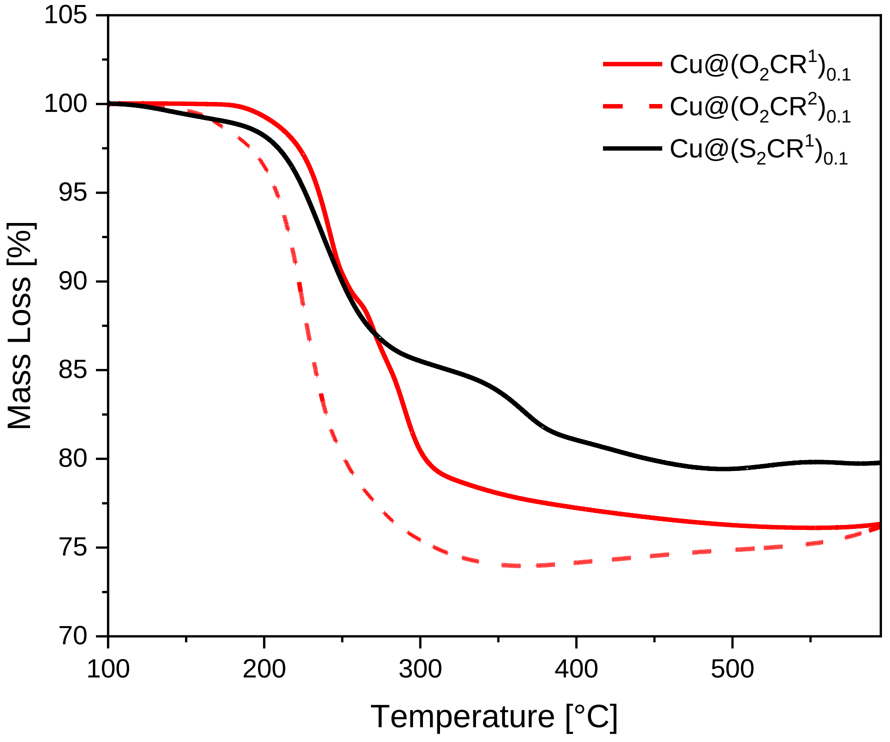


**Figure S72.** TGA thermograms of $Cu(O_2CR^n)_{0.1}$ (n = 1, 2) and $Cu@(S_2CR^1)_{0.1}$, run in sealed aluminum pans under nitrogen. The anticipated mass loss for complete loss of ligand for $Cu(O_2CR^n)_{0.1}$ (n = 1, 2) and $Cu@(S_2CR^1)_{0.1}$ is 25, 28 and 30 %, respectively. Incomplete mass loss in the case of $Cu@(S_2CR^1)_{0.1}$ has been attributed to incomplete combustion of the di(thio)carboxylate group under $N_2$, leading to the formation of surface copper-sulfides.

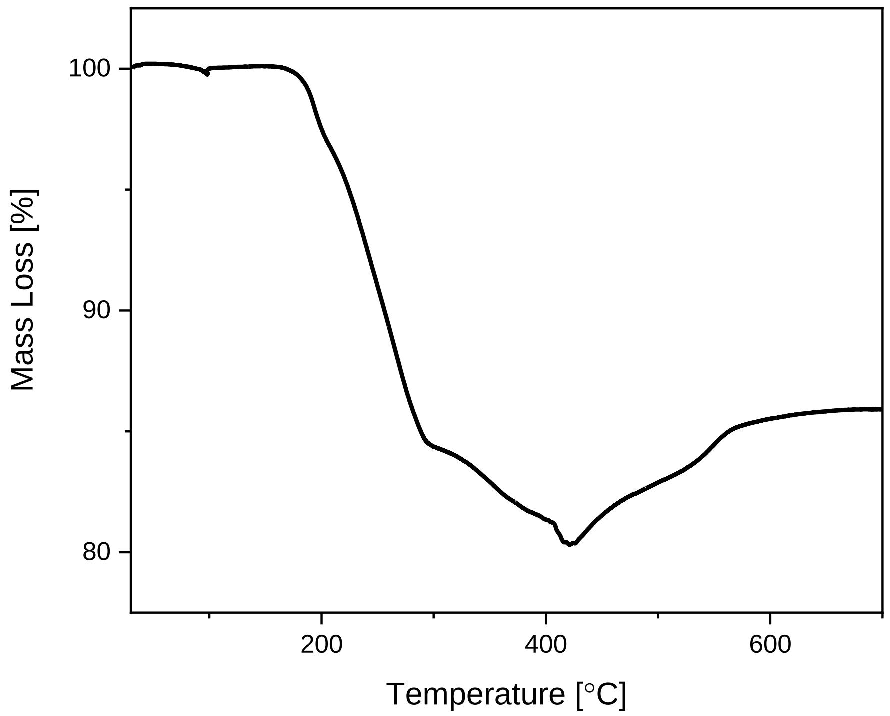


**Figure S73.** TGA thermogram of $Cu_2O@(O_2CR^1)_{0.1}$ in air.

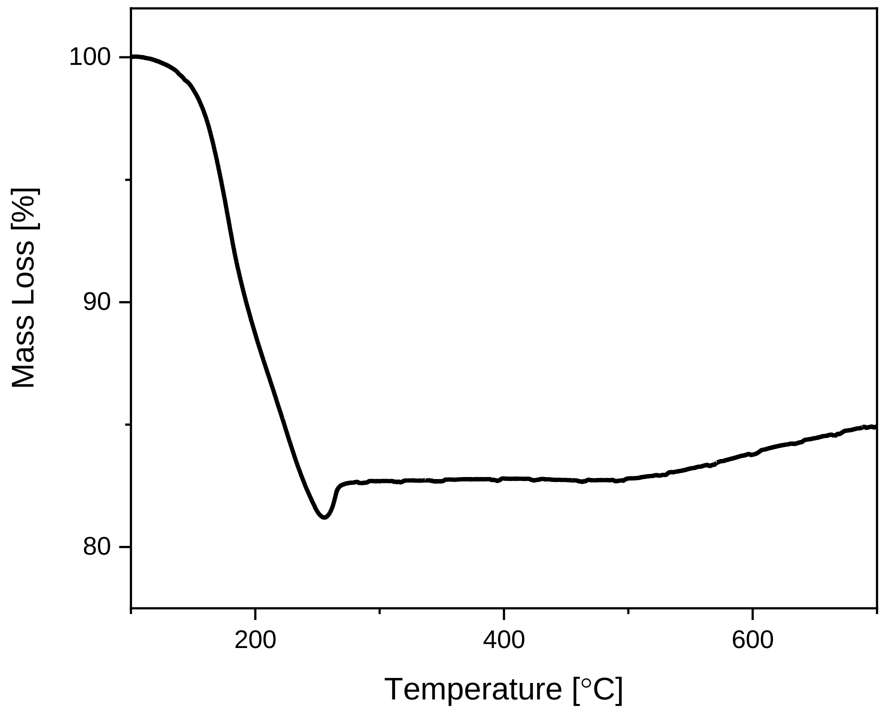


**Figure S74.** TGA thermogram of $Cu_2O@(O_2CR^2)_{0.1}$ in air.

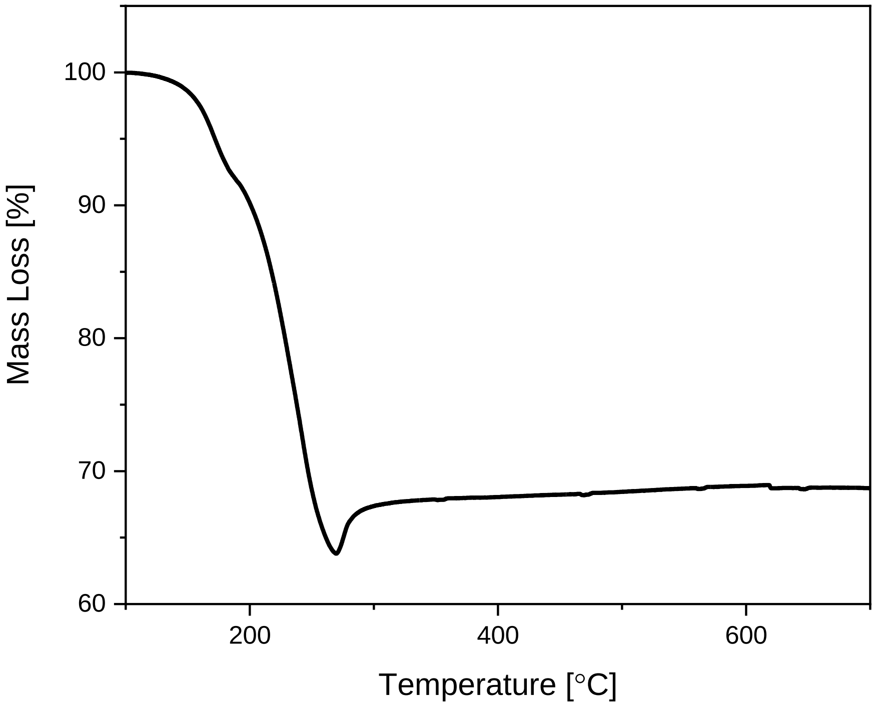


**Figure S75.** TGA thermogram of $Cu_2O@(O_2CR^2)_{0.2}$ in air.

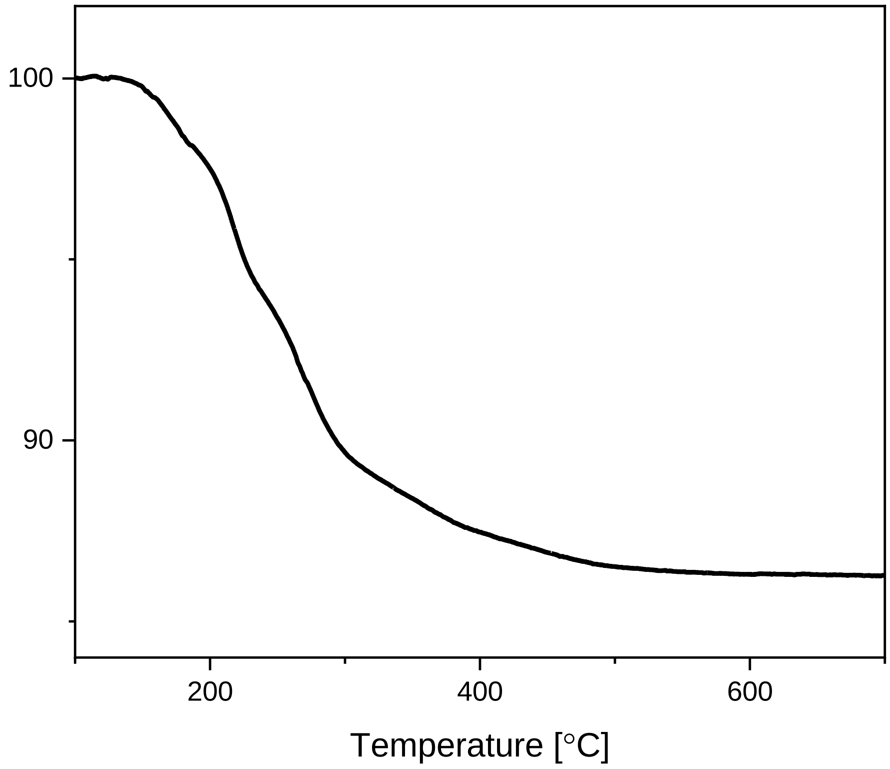


**Figure S76.** TGA thermogram of $Cu_2S@(S_2CR^1)_{0.1}$, synthesized using $S(SiMe_3)_2$, under $N_2$.

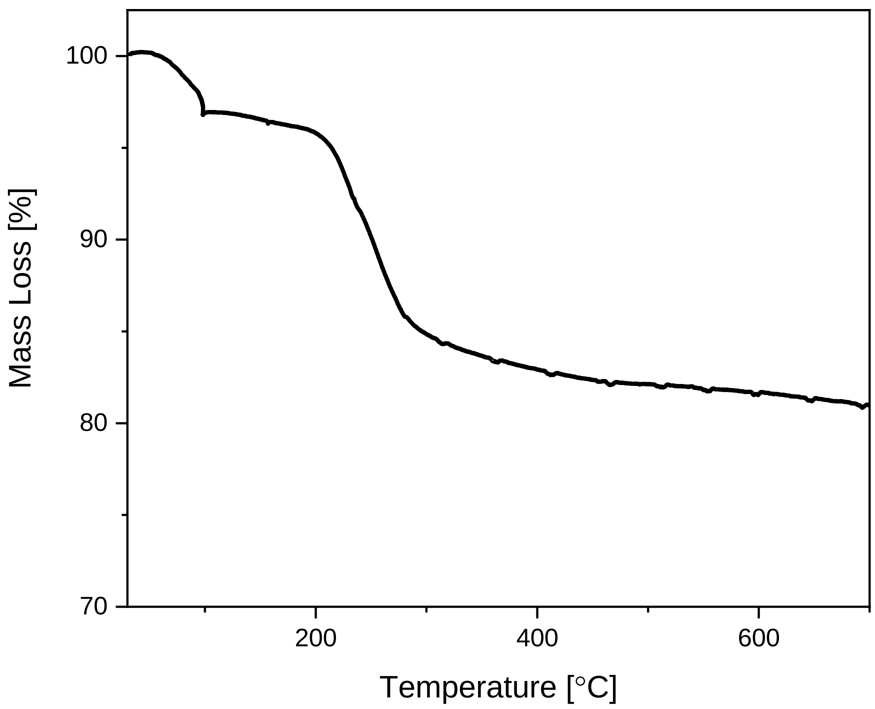


**Figure S77.** TGA thermogram of $Cu_2S@(S_2CR^1)_{0.1}$, synthesized using $H_2S$, under $N_2$.

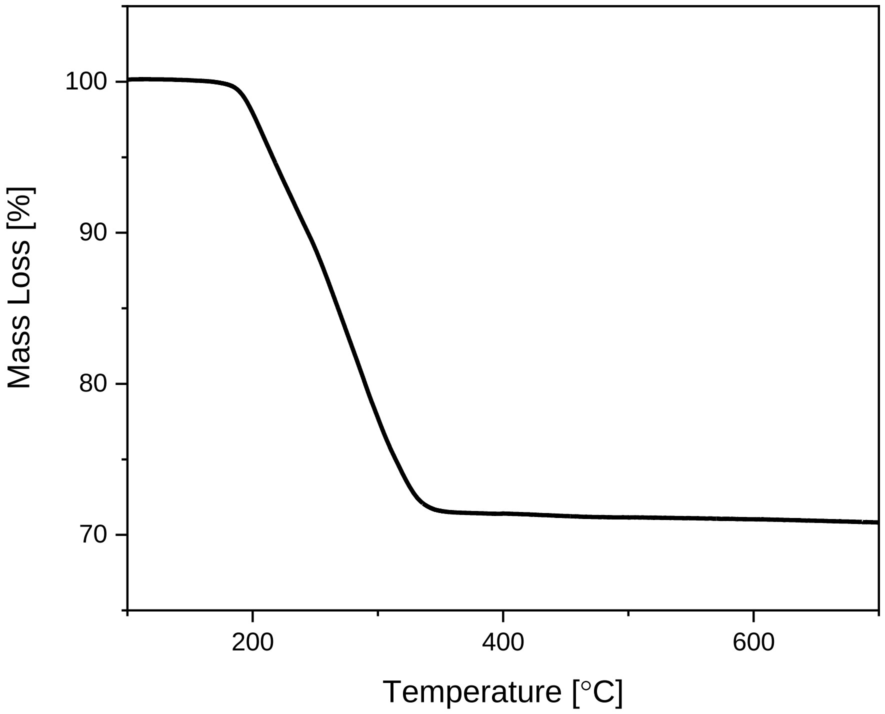


**Figure S78.** TGA thermogram of $Cu_2S@(S_2CR^1)_{0.2}$ under $N_2$.

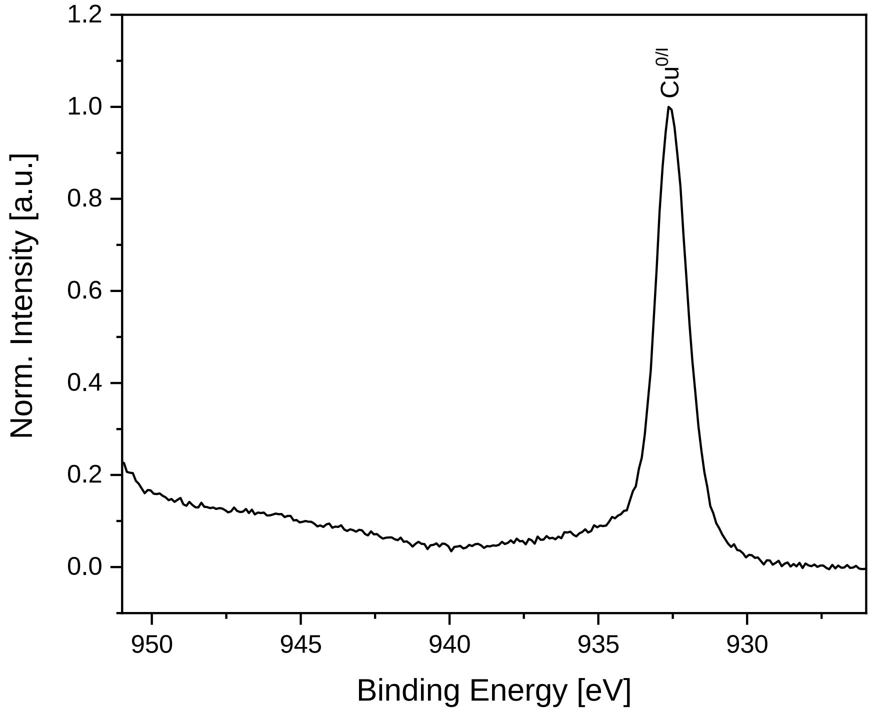


**Figure S79.** Cu $2p_{3/2}$ XP spectrum of Cu@$(O_2CR^1)_{0.1}$.

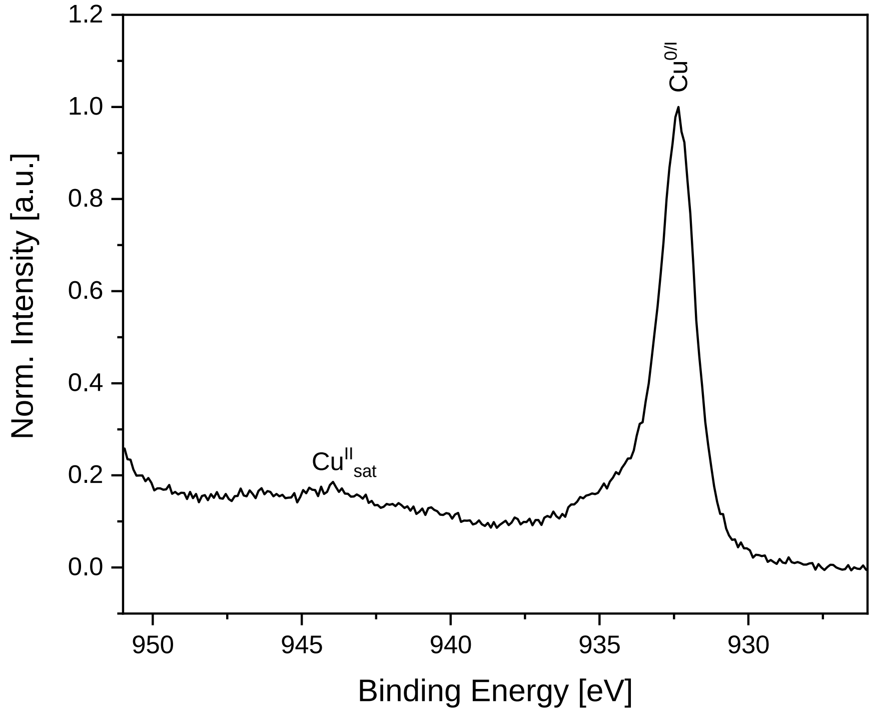


**Figure S80.** Cu $2p_{3/2}$ XP spectrum of Cu@$(O_2CR^2)_{0.1}$.

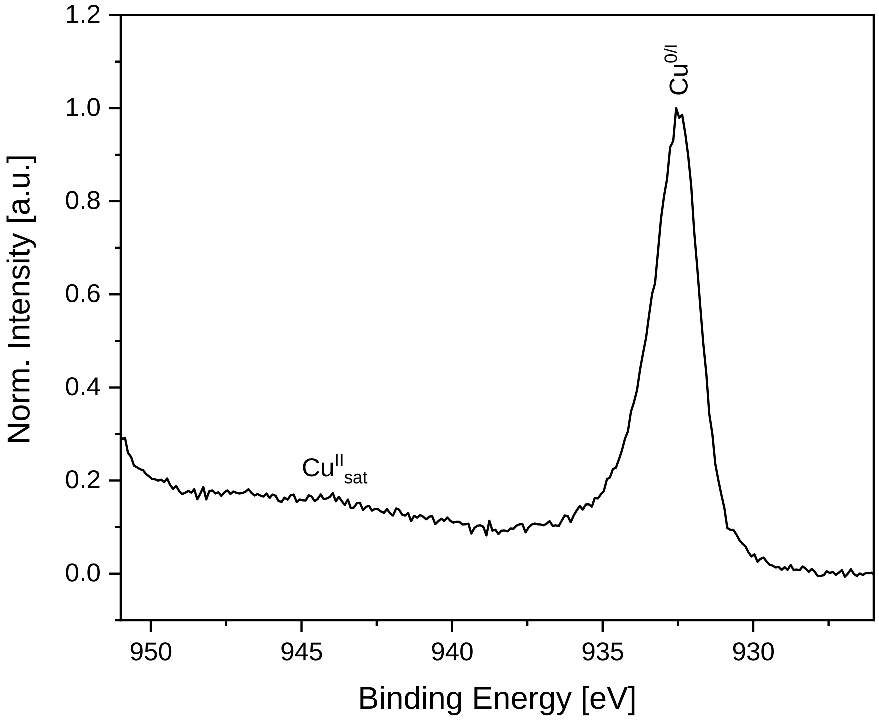


**Figure S81.** Cu 2$p_{3/2}$ XP spectrum of Cu@($S_2CR^1$)$_{0.1}$.

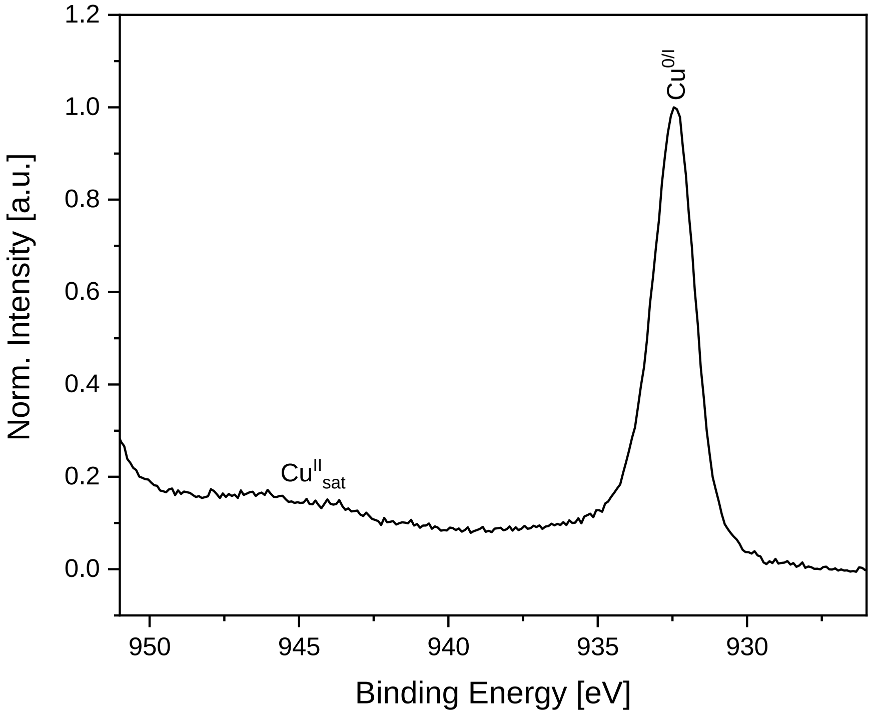


**Figure S82.** Cu 2$p_{3/2}$ XP spectrum of $Cu_2O$@($O_2CR^1$)$_{0.1}$.

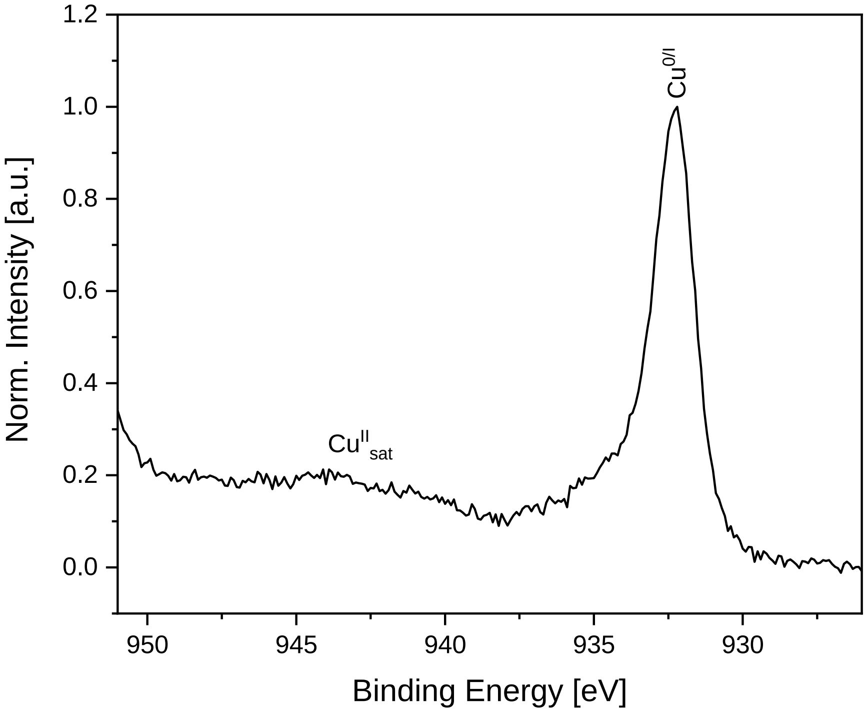


**Figure S83.** Cu 2$p_{3/2}$ XP spectrum of $Cu_2O@(O_2CR^2)_{0.1}$.

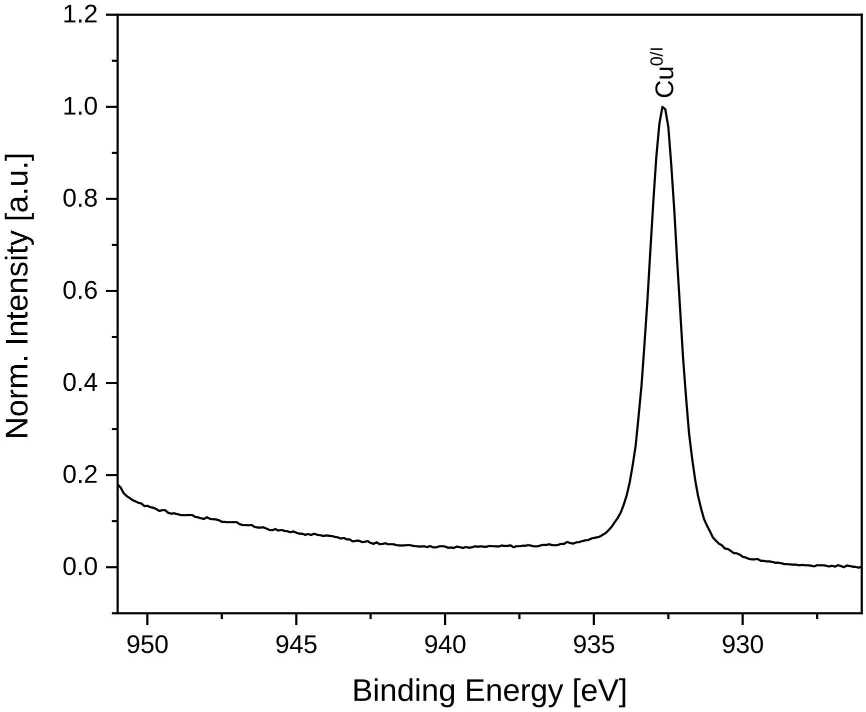


**Figure S84.** Cu 2$p_{3/2}$ XP spectrum of $Cu_2S@(S_2CR^1)_{0.1}$.

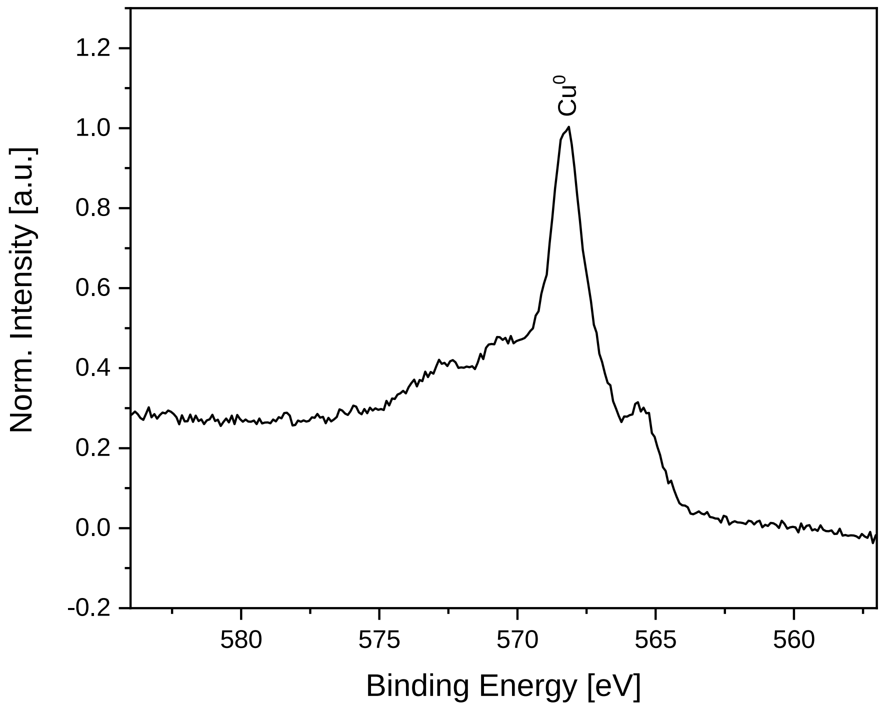


**Figure S85.** Cu $L_3M_{4,5}M_{4,5}$ XP spectrum of Cu@$(O_2CR^1)_{0.1}$.

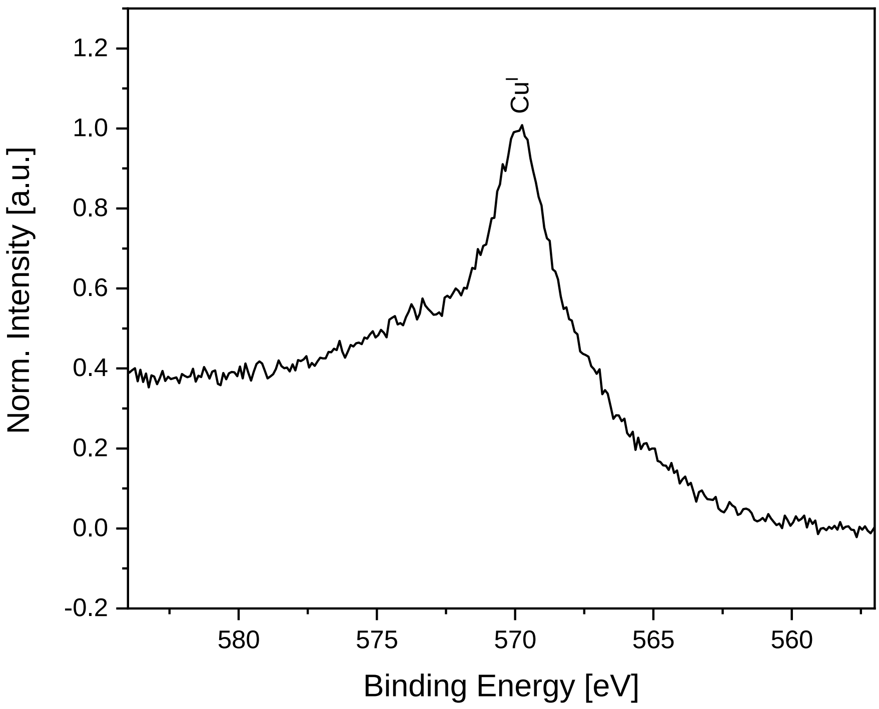


**Figure S86.** Cu $L_3M_{4,5}M_{4,5}$ XP spectrum of Cu@$(O_2CR^2)_{0.1}$. Samples show a shift to higher binding energy (BE) and a loss of the metallic line features in the Cu $L_3M_{4,5}M_{4,5}$ spectrum. These data are consistent with the presence of Cu(I) surface species, perhaps in cuprous oxide environments.[36]

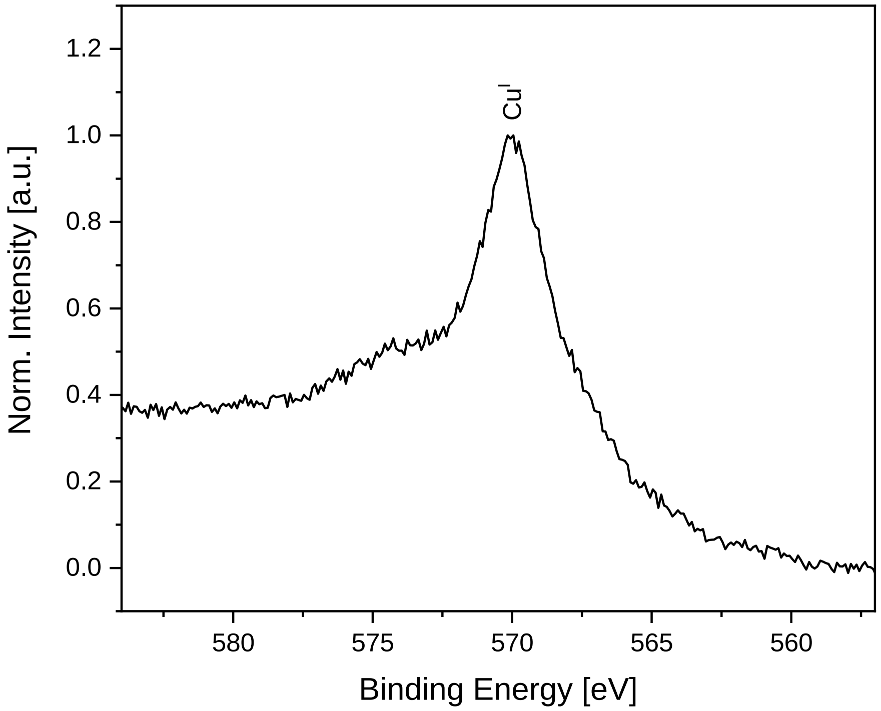


**Figure S87.** Cu $L_3M_{4,5}M_{4,5}$ XP spectrum of Cu@($S_2CR^1$)$_{0.1}$. Samples show a shift to higher binding energy (BE) and a loss of the metallic line features in the Cu $L_3M_{4,5}M_{4,5}$ spectrum. These data are consistent with the presence of Cu(I) surface species.[36]

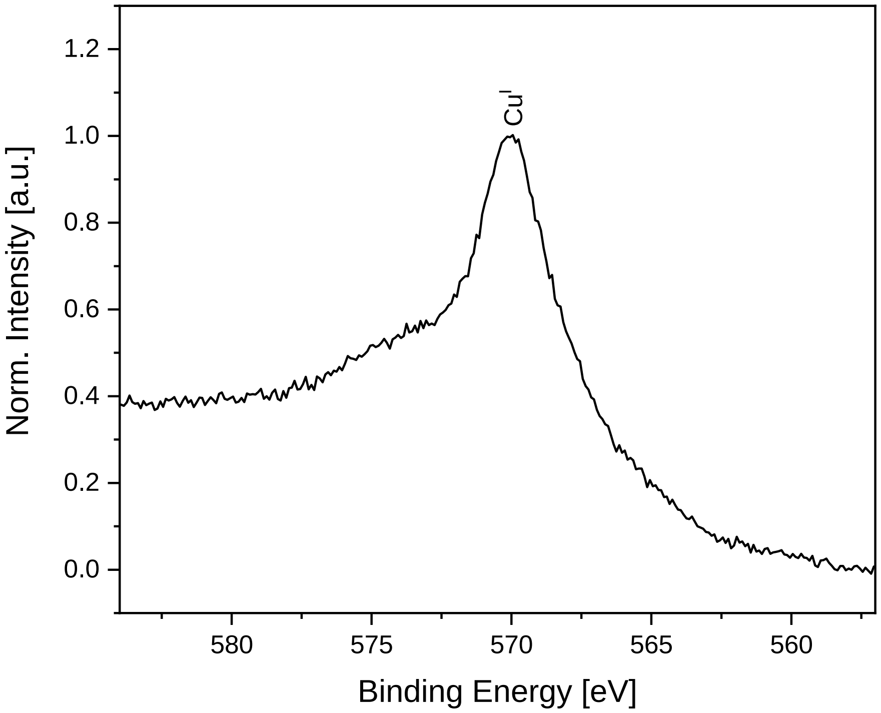


**Figure S88.** Cu $L_3M_{4,5}M_{4,5}$ XP spectrum of $Cu_2O$@($O_2CR^1$)$_{0.1}$.

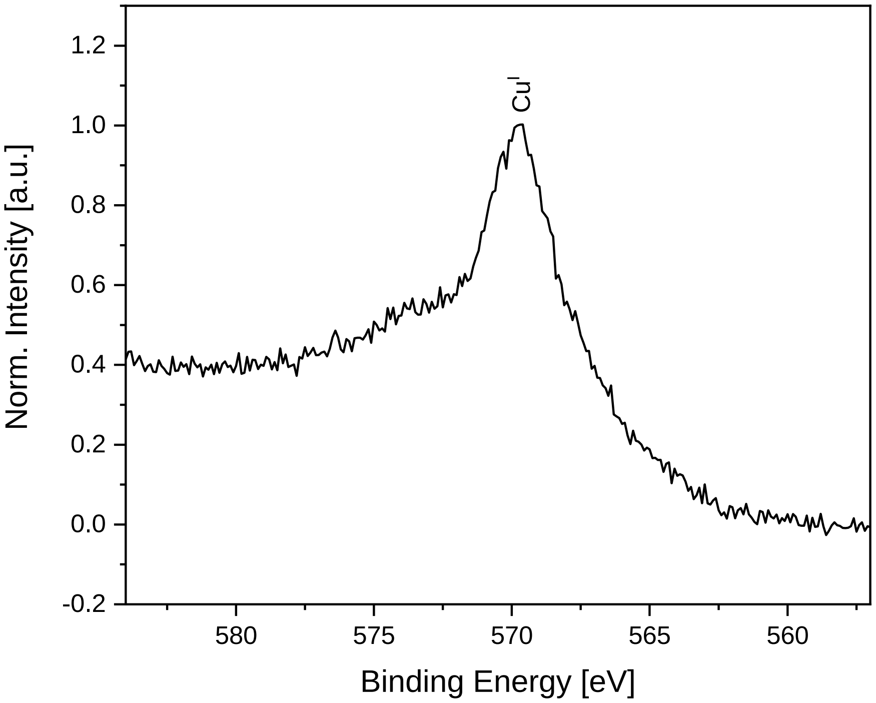


**Figure S89.** Cu $L_3M_{4,5}M_{4,5}$ XP spectrum of $Cu_2O@(O_2CR^2)_{0.1}$.

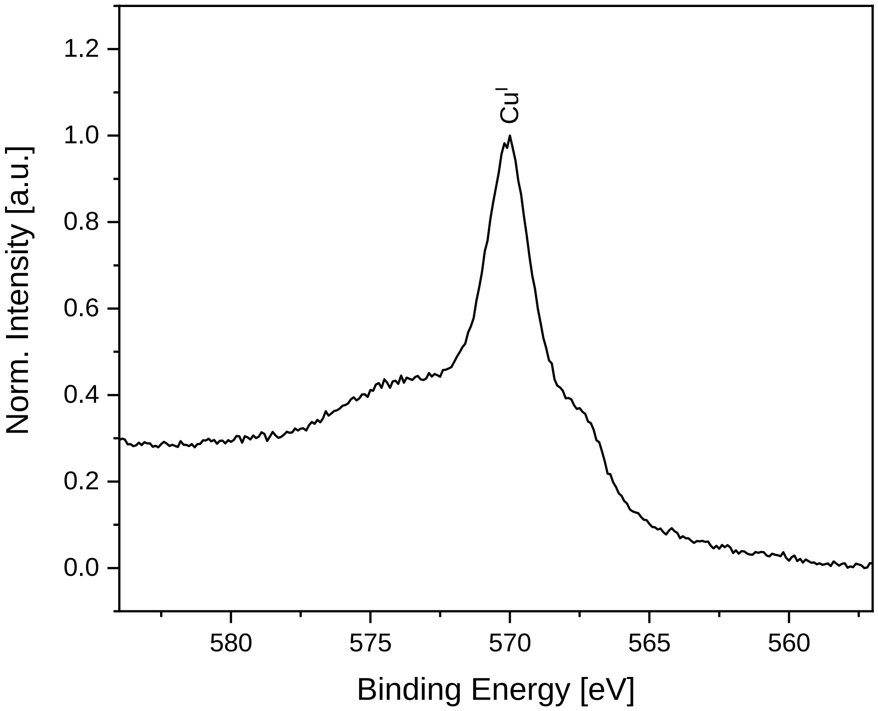


**Figure S90.** Cu $L_3M_{4,5}M_{4,5}$ XP spectrum of $Cu_2S@(S_2CR^1)_{0.1}$.

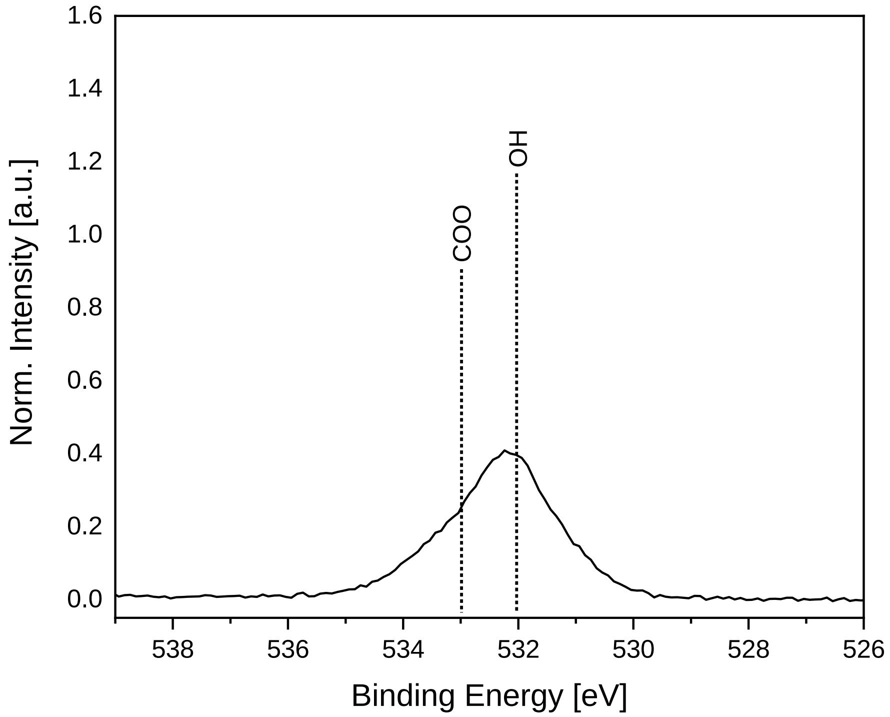


**Figure S91.** O 1*s* XP spectrum of Cu@$(O_2CR^1)_{0.1}$.

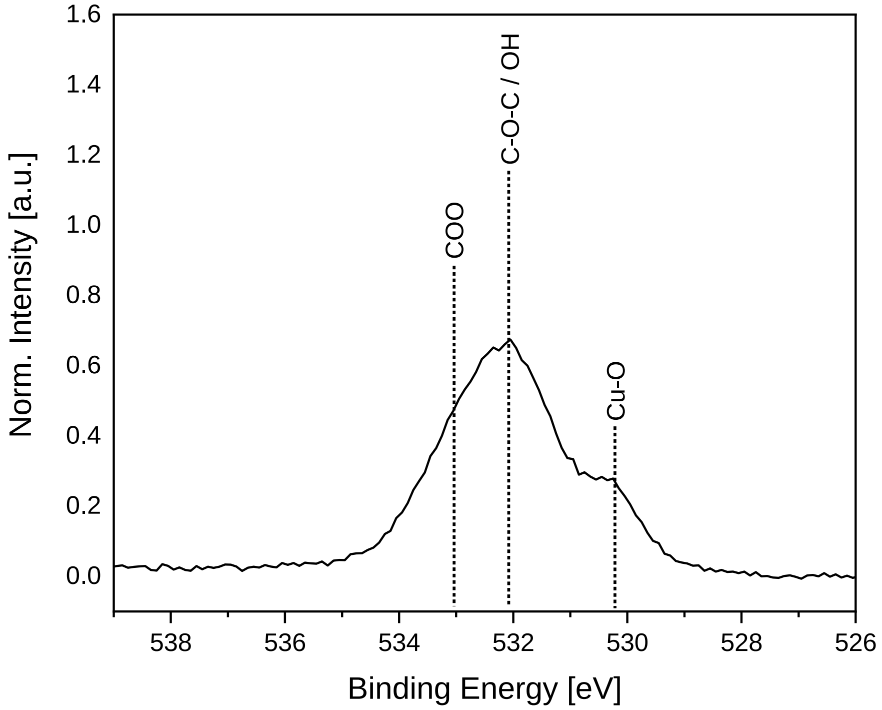


**Figure S92.** O 1*s* XP spectrum of Cu@$(O_2CR^2)_{0.1}$. The sample shows a low BE feature at ~530 eV. This feature, which is absent in the metallic Cu@$(O_2CR^1)_{0.1}$ sample, likely indicates adventitious surface oxidation during XPS sample preparation. The FT-IR spectrum shows that the surface -OH groups are absent from all the Cu samples and only appear in the $Cu_2O$ samples (see Figures S60-S68 above), indicating surface oxidation does not occur during nanoparticle synthesis.

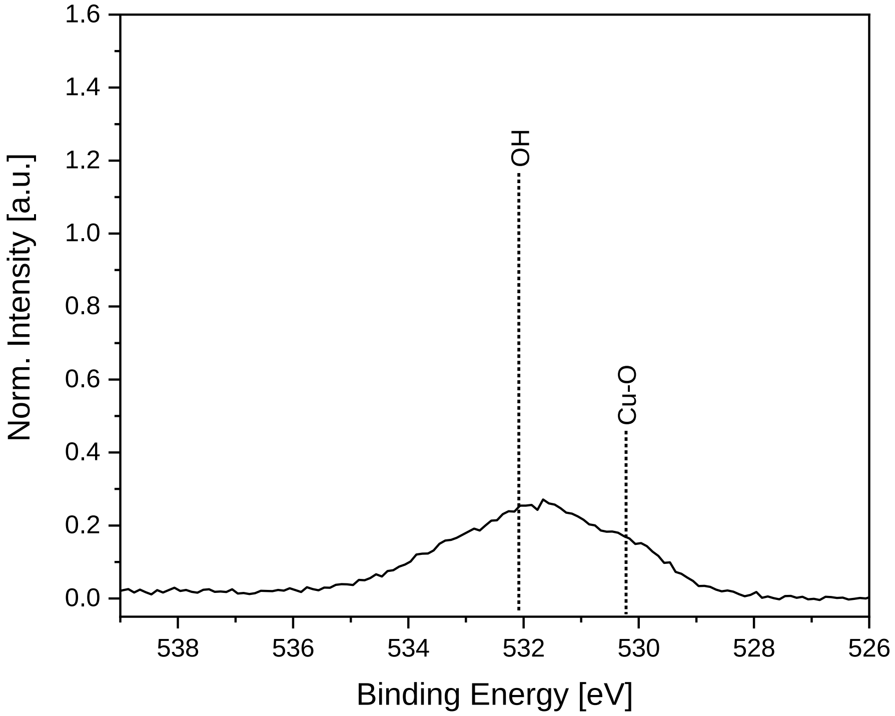


**Figure S93.** O 1*s* XP spectrum of Cu@($S_2CR^1$)$_{0.1}$. The sample shows a low BE feature at ~530 eV. This feature, which is absent in the metallic Cu@($O_2CR^1$)$_{0.1}$ sample, likely indicates adventitious surface oxidation during XPS sample preparation. The FT-IR spectrum shows that the surface -OH groups are absent from all the Cu samples (see Figures S60-S68 above), indicating surface oxidation does not occur during nanoparticle synthesis.

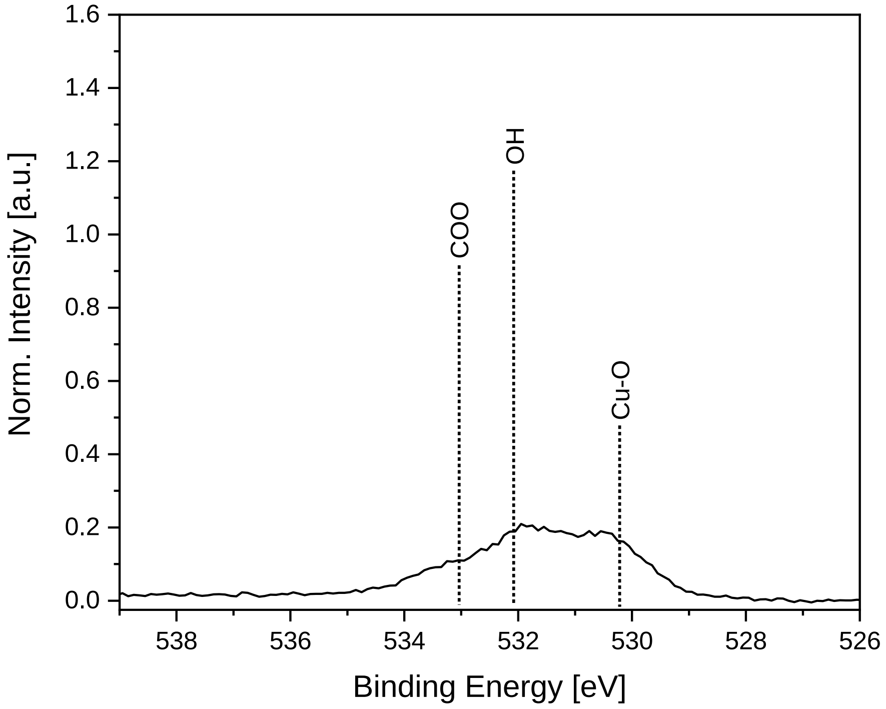


**Figure S94.** O 1*s* XP spectrum of $Cu_2O$@($O_2CR^1$)$_{0.1}$.

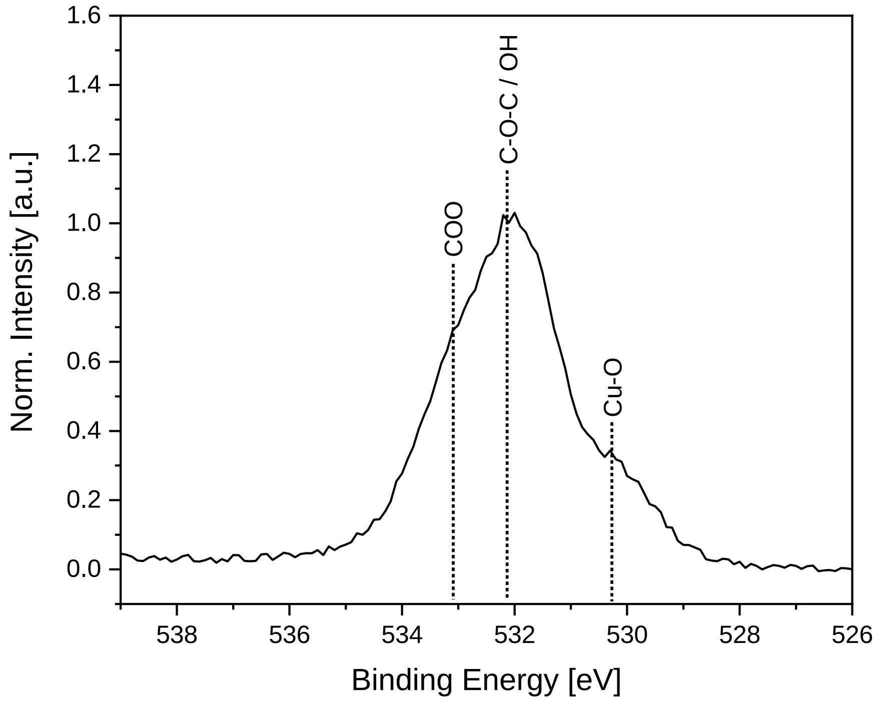


**Figure S95.** O 1*s* XP spectrum of $Cu_2O@(O_2CR^2)_{0.1}$.

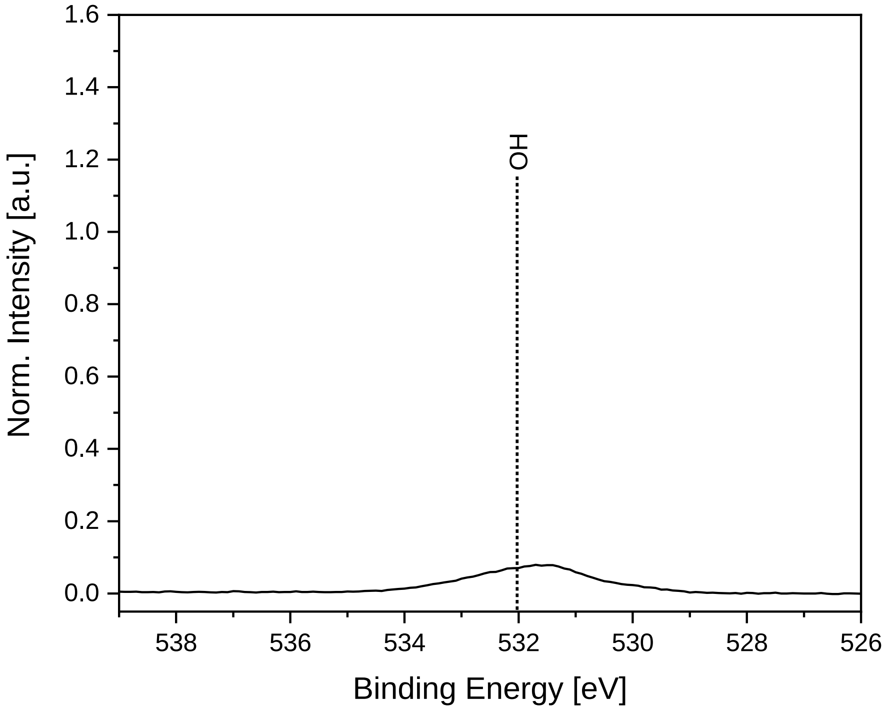


**Figure S96.** O 1*s* XP spectrum of $Cu_2S@(S_2CR^1)_{0.1}$.

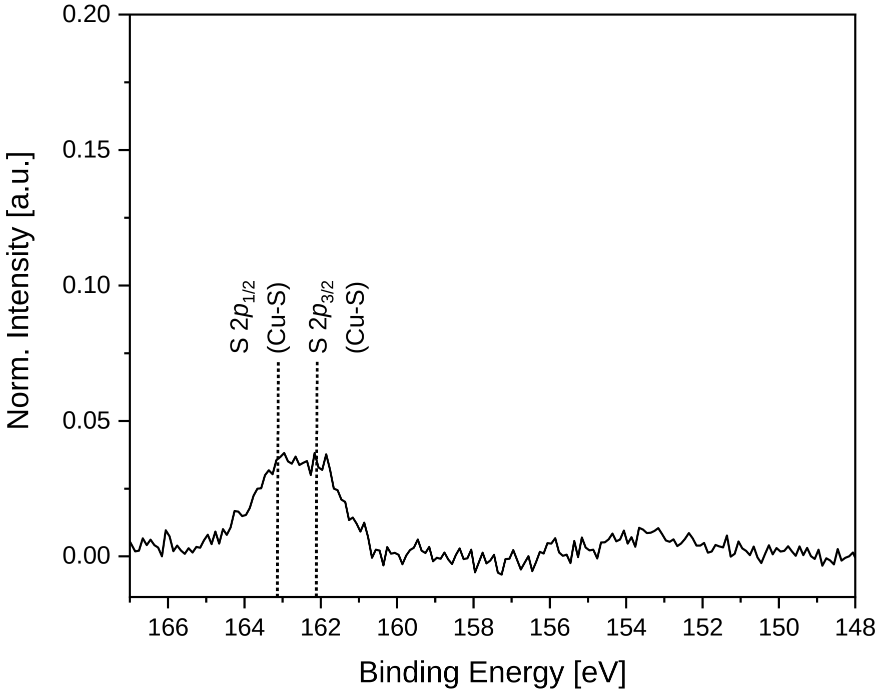


**Figure S97.** S 2*p* / Si 2*s* XP spectrum of Cu@$(S_2CR^1)_{0.1}$. The spectrum shows a clear signal from S groups of the ligand which is absent for the carboxylate samples. From peak fits to the Cu 2$p_{3/2}$ and S 2*p* spectra a ratio of Cu:S≈4:1 was found, close to the intended stoichiometry of 5:1 and potentially skewed by XPS surface sensitivity.[38]

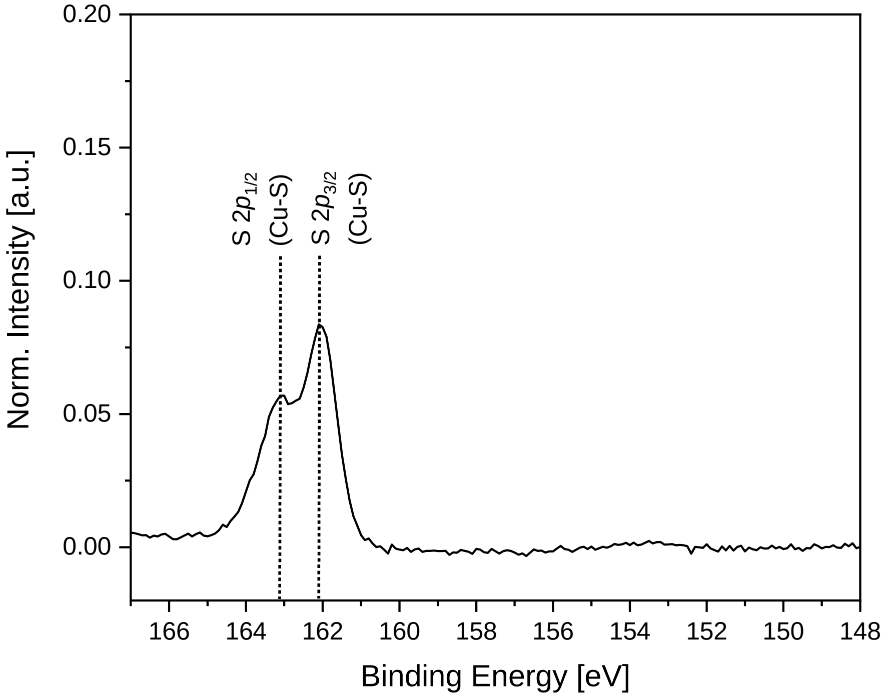


**Figure S98.** S 2*p* / Si 2*s* XP spectrum of $Cu_2S$@$(O_2CR^1)_{0.1}$.

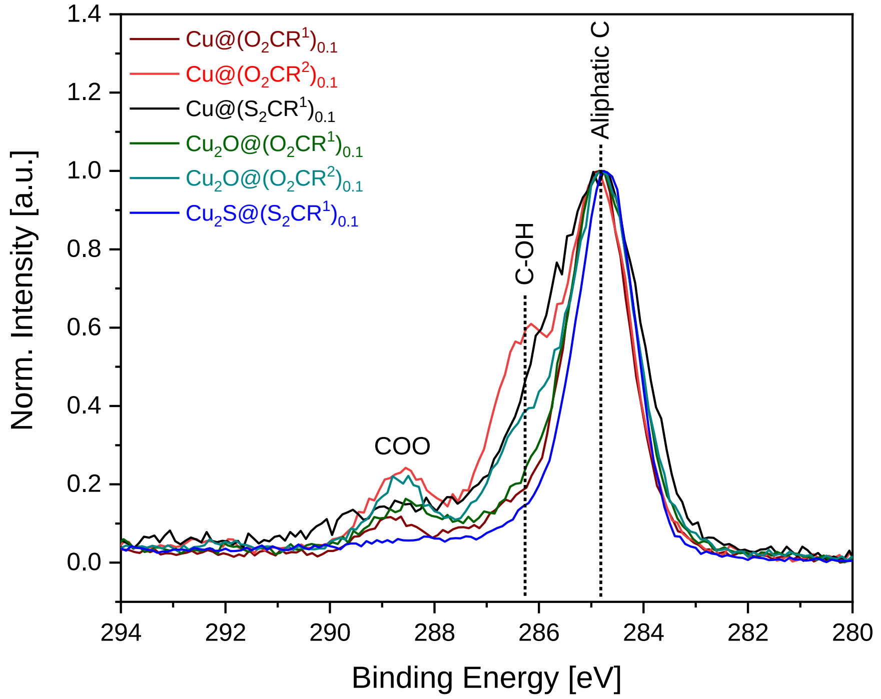


**Figure S99.** C 1*s* XP spectra of $Cu@(X_2CR^n)_{0.1}$ (X = O, S; n = 1, 2), $Cu_2O@(O_2CR^n)_{0.1}$ (n = 1, 2) and $Cu_2S@(S_2CR^1)_{0.1}$.

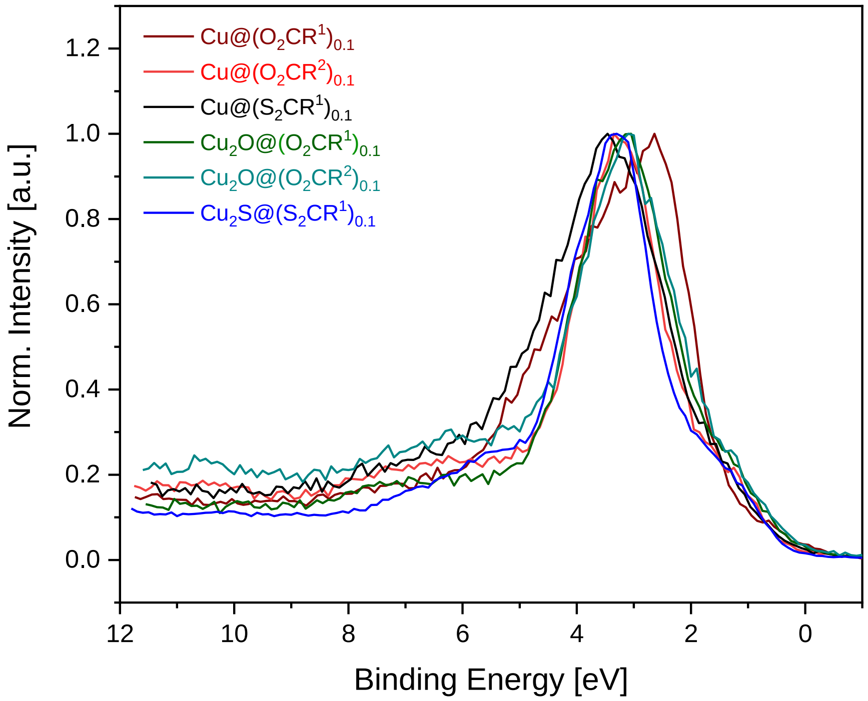


**Figure S100.** Valence band XP spectra of $Cu@(X_2CR^n)_{0.1}$ (X = O, S; n = 1, 2), $Cu_2O@(O_2CR^n)_{0.1}$ (n = 1, 2) and $Cu_2S@(S_2CR^1)_{0.1}$. Surface oxidation is observed for $Cu@(O_2CR^2)_{0.1}$ and $Cu@(S_2CR^1)_{0.1}$, in which a small shift to higher binding energy is observed.

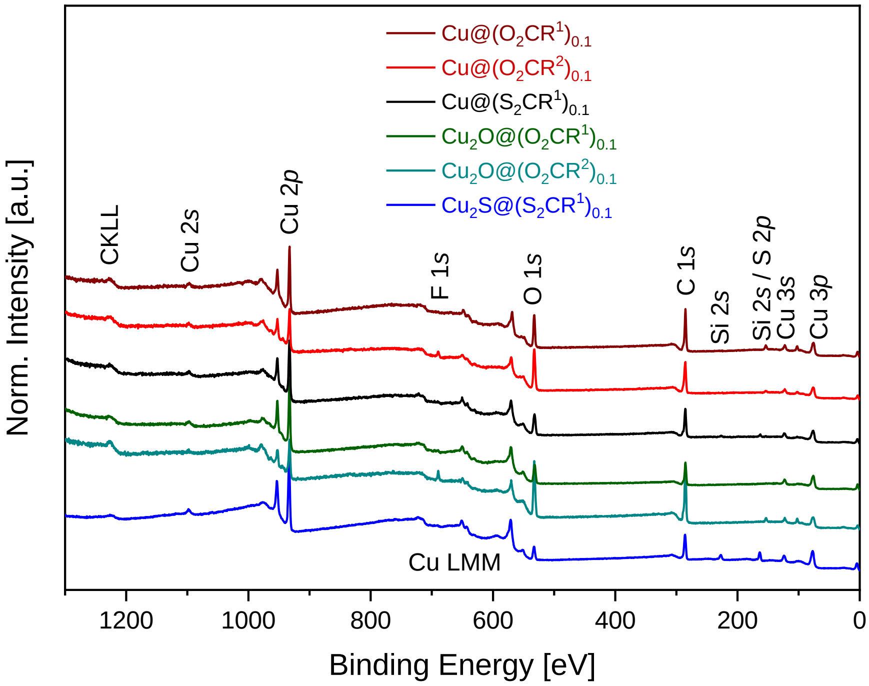


**Figure S101.** Survey XP spectra of Cu@($X_2CR^n$)$_{0.1}$ (X = O, S; n = 1, 2), $Cu_2O$@($O_2CR^n$)$_{0.1}$ (n = 1, 2) and $Cu_2S$@($S_2CR^1$)$_{0.1}$.

## Solubility Studies:

**Example Procedure:** Toluene (1 mL) was added to $Cu_2S$@($S_2CR^1$)$_{0.1}$ (20 mg) in a vial and stirred for 30 minutes at room temperature, after which the solution was filtered. An aliquot of the filtrate (0.5 mL) was then evaporated to dryness under reduced pressure inside a separate, pre-weighed vial. Mass of vial = 6.6875 g; mass after = 6.6993 g; solubility = 24 mg/mL.

## Experimental Spectra and Data Associated with Ligand Exchange Reactivity for $Cu_2S$:

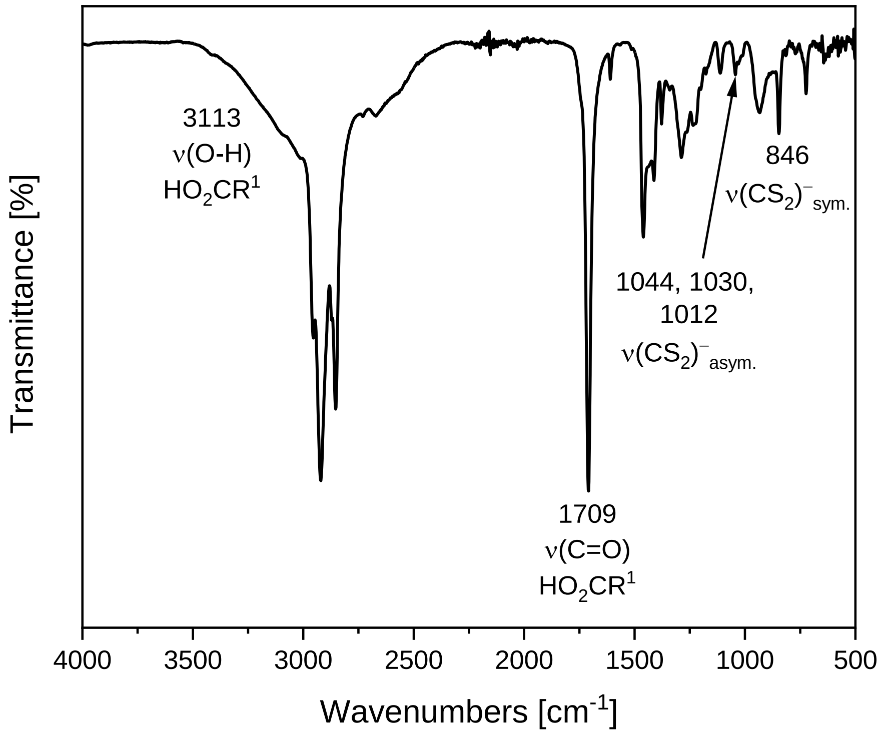


**Figure S102.** FT-IR spectrum of the crude reaction mixture following treatment of $Cu_2S@(S_2CR^1)_{0.2}$ with 0.2 equiv. of $HO_2CR^1$.

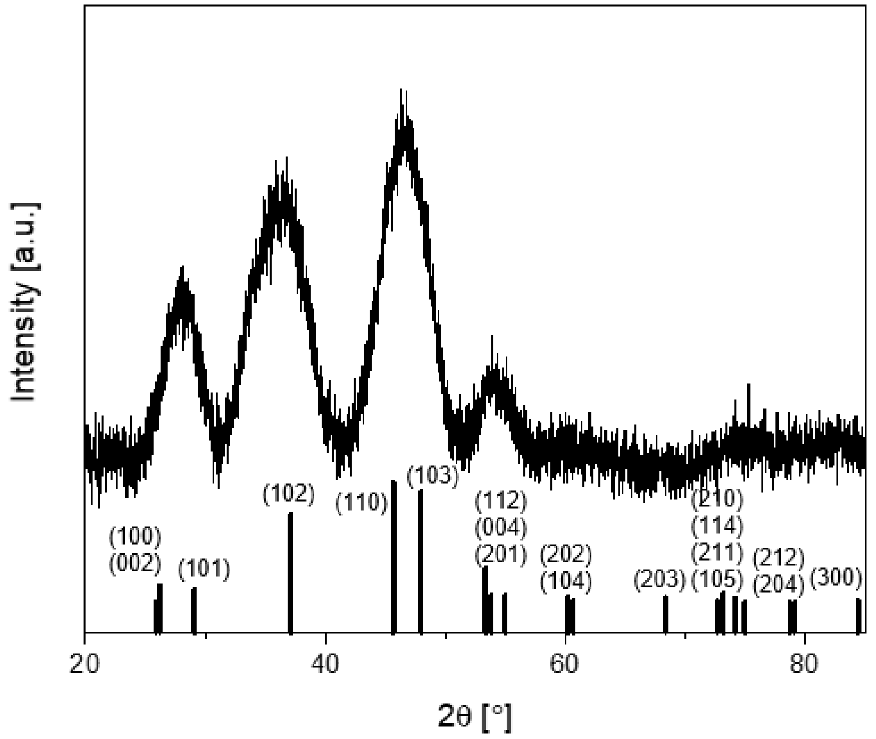


**Figure S103.** Powder X-ray diffraction pattern following treatment of $Cu_2S@(S_2CR^1)_{0.2}$ with 0.2 equiv. of $HO_2CR^1$; pattern indexed against hexagonal $Cu_2S$ as vertical bars (JCPDS 01-084-0208).

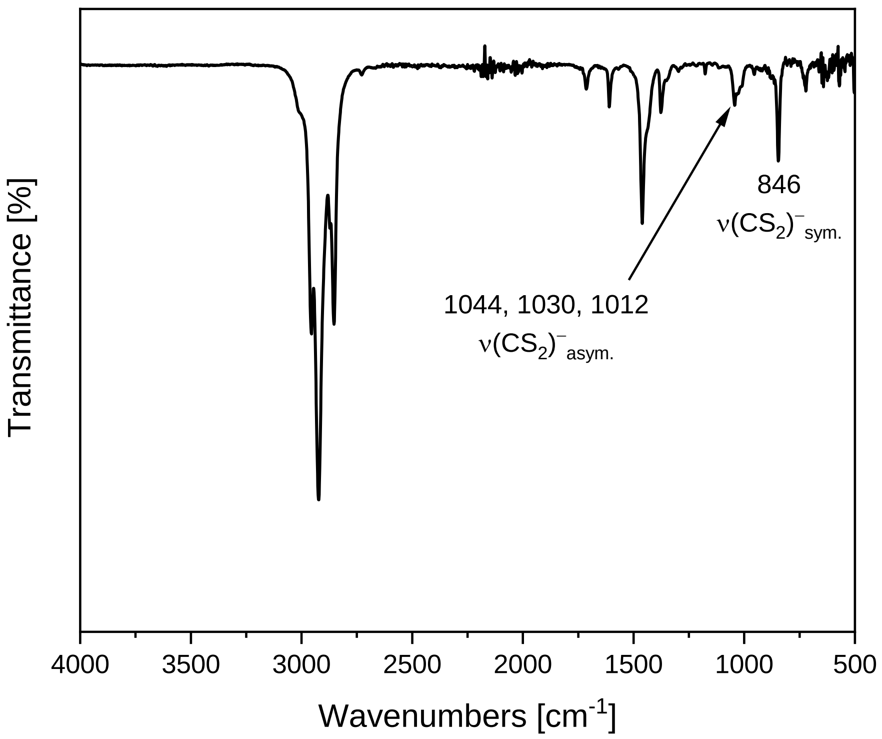


**Figure S104.** FT-IR spectrum of the isolated product following treatment of $Cu_2S@(S_2CR^1)_{0.2}$ with 0.2 equiv. of $HO_2CR^1$.

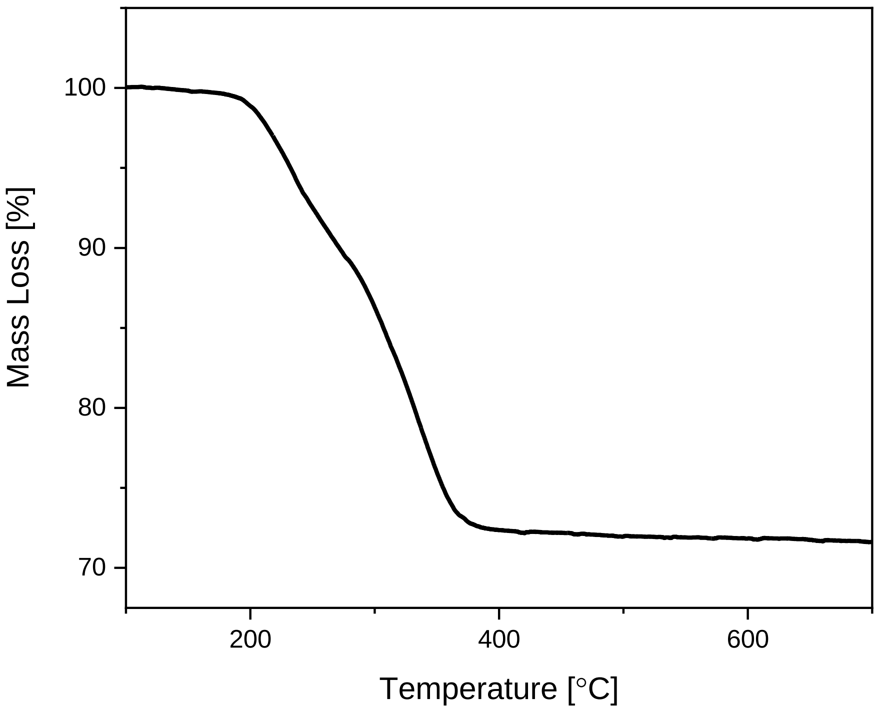


**Figure S105.** TGA Thermogram (under $N_2$) of the isolated product following treatment of $Cu_2S@(S_2CR^1)_{0.2}$ with 0.2 equiv. of $HO_2CR^1$.

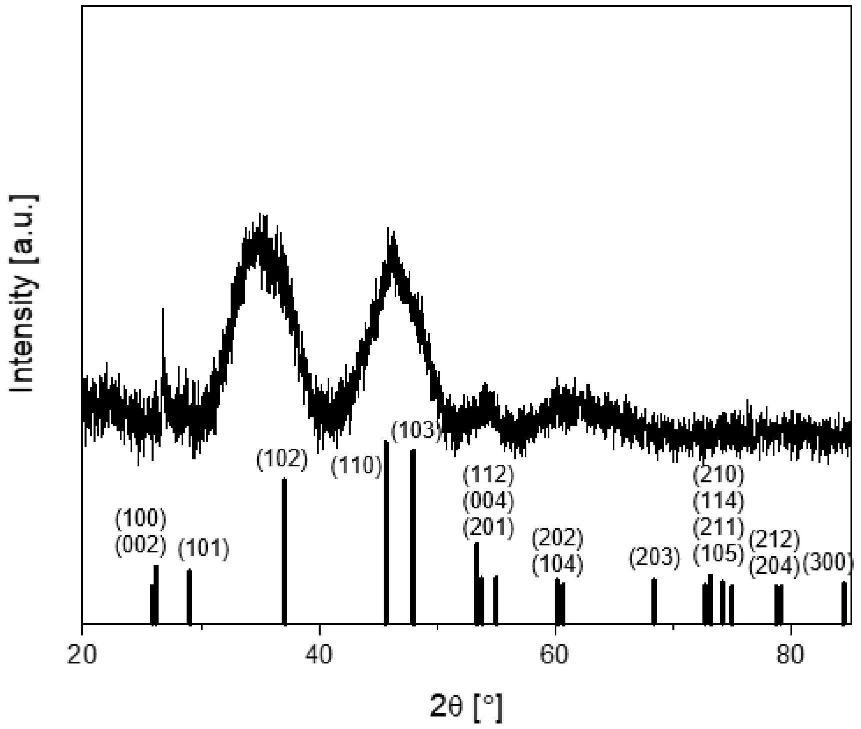


**Figure S106.** Powder X-ray diffraction pattern following treatment of $Cu_2S@(S_2CR^1)_{0.2}$ with 0.4 equiv. of $HO_2CR^1$ at room temperature; pattern indexed against hexagonal $Cu_2S$ as vertical bars (JCPDS 01-084-0208).

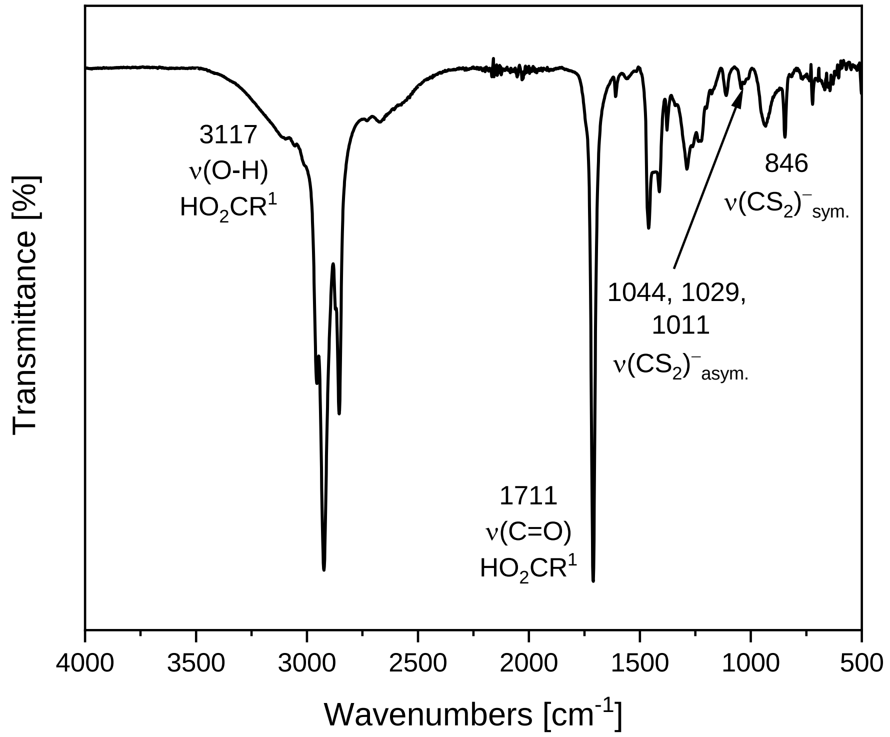


**Figure S107.** FT-IR spectrum of the crude reaction mixture following treatment of $Cu_2S@(S_2CR^1)_{0.2}$ with 0.4 equiv. of $HO_2CR^1$ at room temperature.

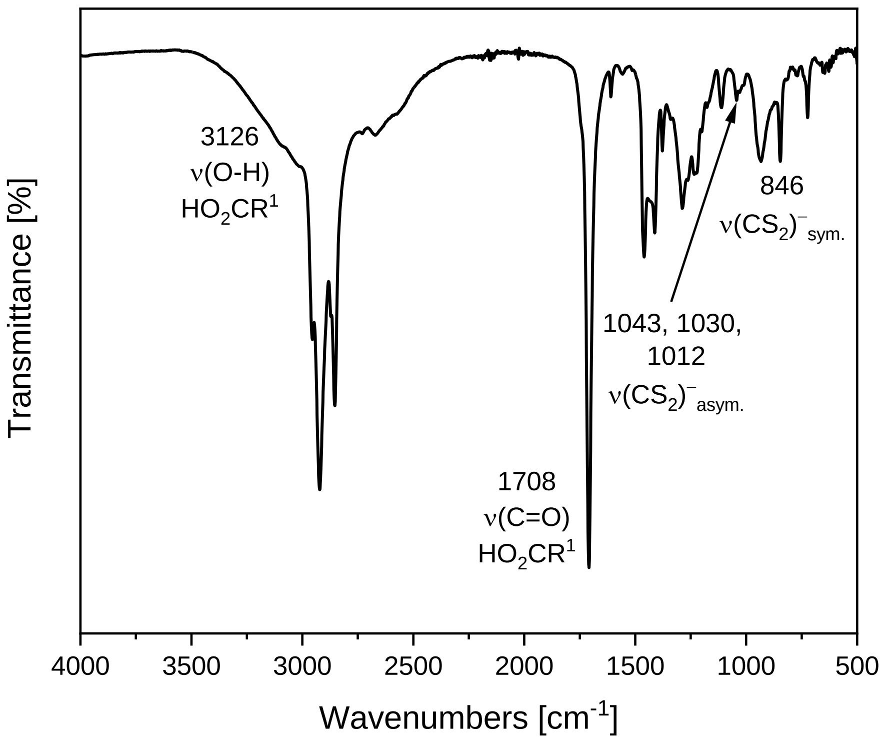


**Figure S108.** FT-IR spectrum of the crude reaction mixture following treatment of $Cu_2S@(S_2CR^1)_{0.2}$ with 0.4 equiv. of $HO_2CR^1$ at 100 °C.

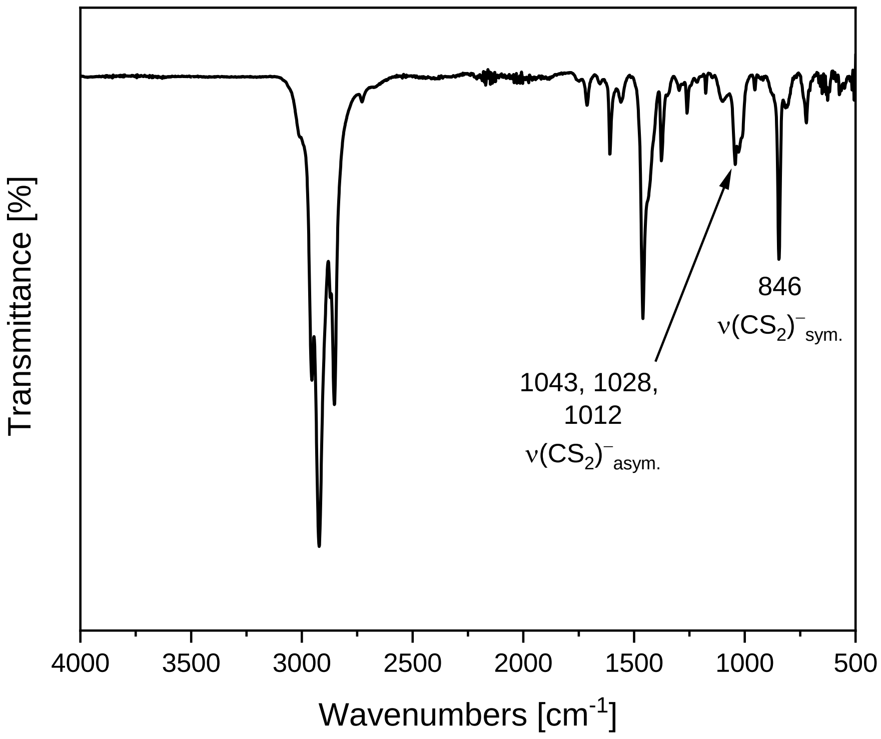


**Figure S109.** FT-IR spectrum of the isolated product following treatment of $Cu_2S@(S_2CR^1)_{0.2}$ with 0.4 equiv. of $HO_2CR^1$ at room temperature.

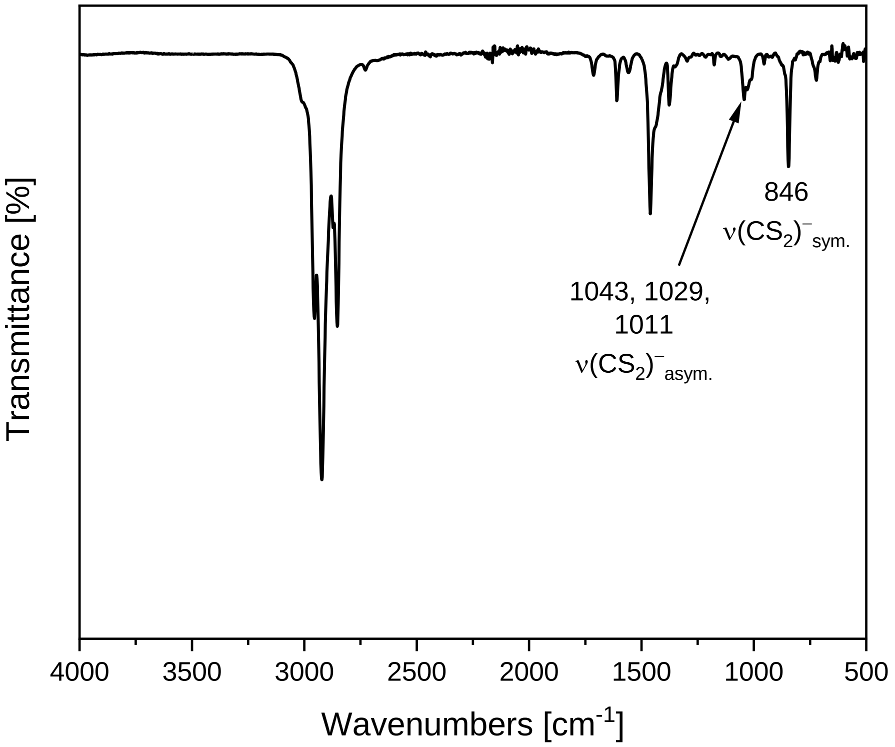


**Figure S110.** FT-IR spectrum of the isolated product following treatment of $Cu_2S@(S_2CR^1)_{0.2}$ with 0.4 equiv. of $HO_2CR^1$ at 100 °C.

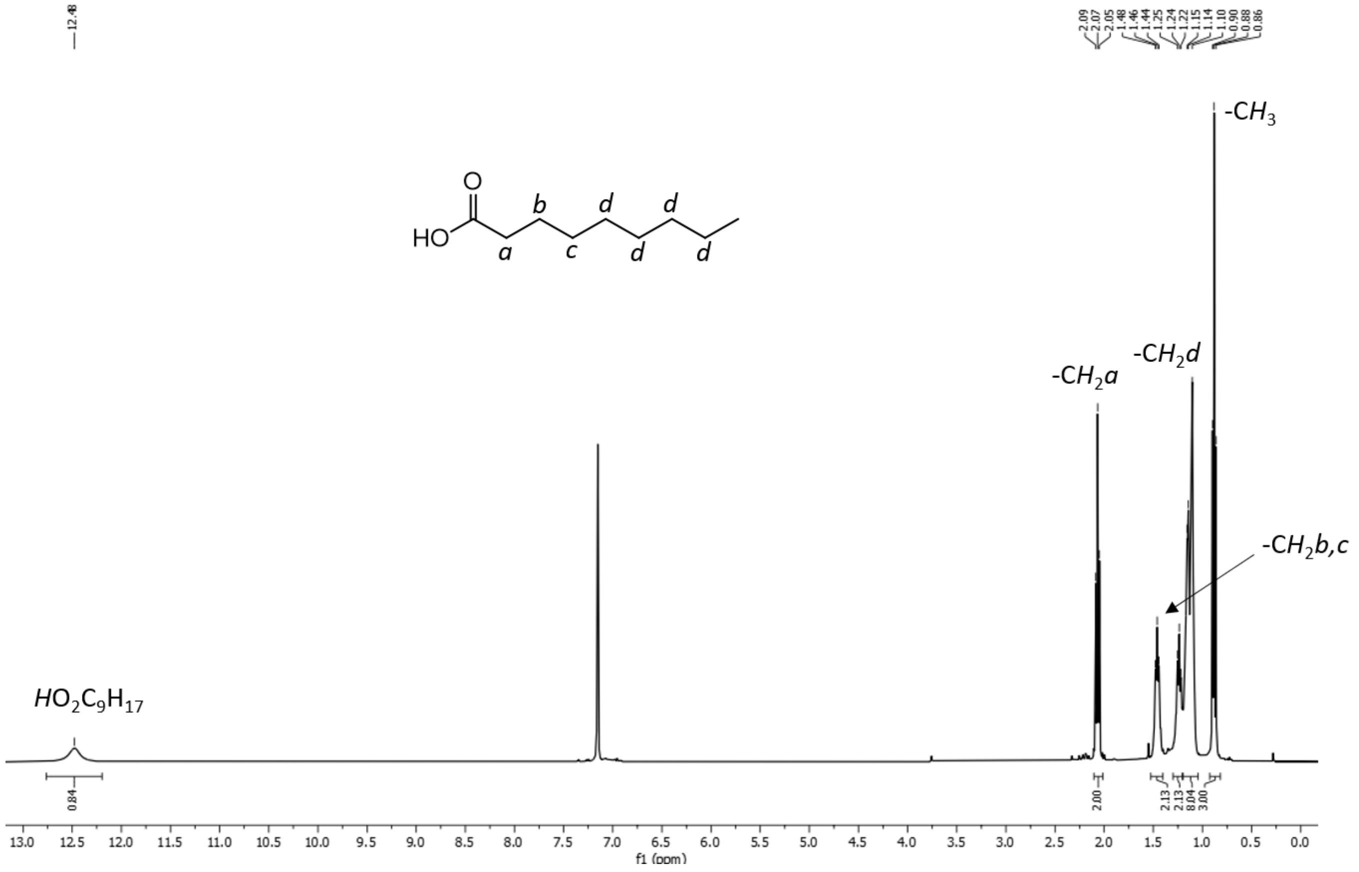


**Figure S111.** $^1H$ NMR spectrum following isolating the product from treating $Cu_2S@(S_2CR^1)_{0.2}$ with 0.4 equiv. of $HO_2CR^1$ at room temperature, and evaporating the mother liquors to dryness ($C_6D_6$, 400 MHz, 300 K); only unreacted $HO_2CR^1$ is observed.

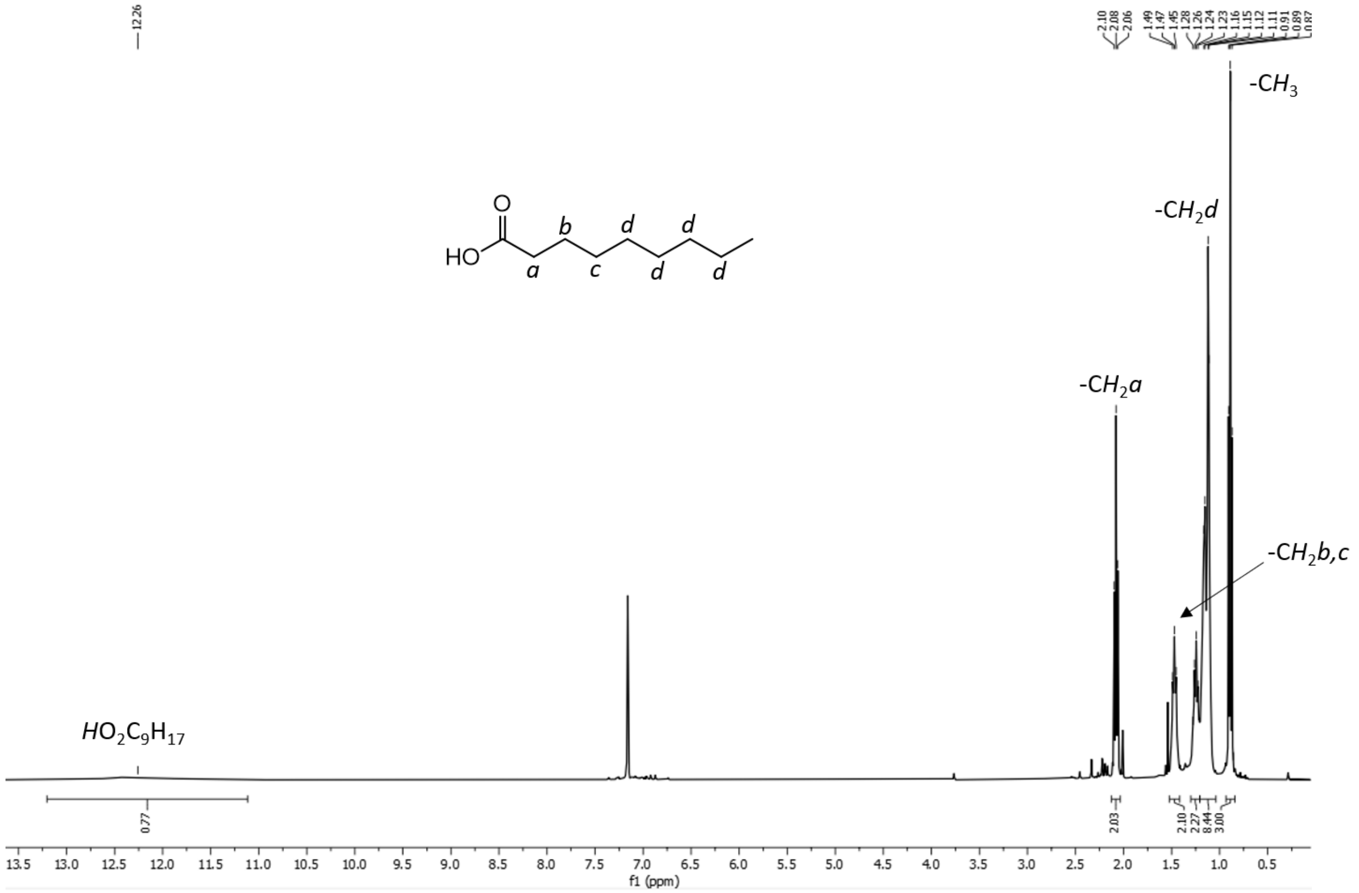


**Figure S112.** $^1$H NMR spectrum following isolating the product from treating $Cu_2S@(S_2CR^1)_{0.2}$ with 0.4 equiv. of $HO_2CR^1$ at 100 °C, and evaporating the mother liquors to dryness ($C_6D_6$, 400 MHz, 300 K); only unreacted $HO_2CR^1$ is observed.

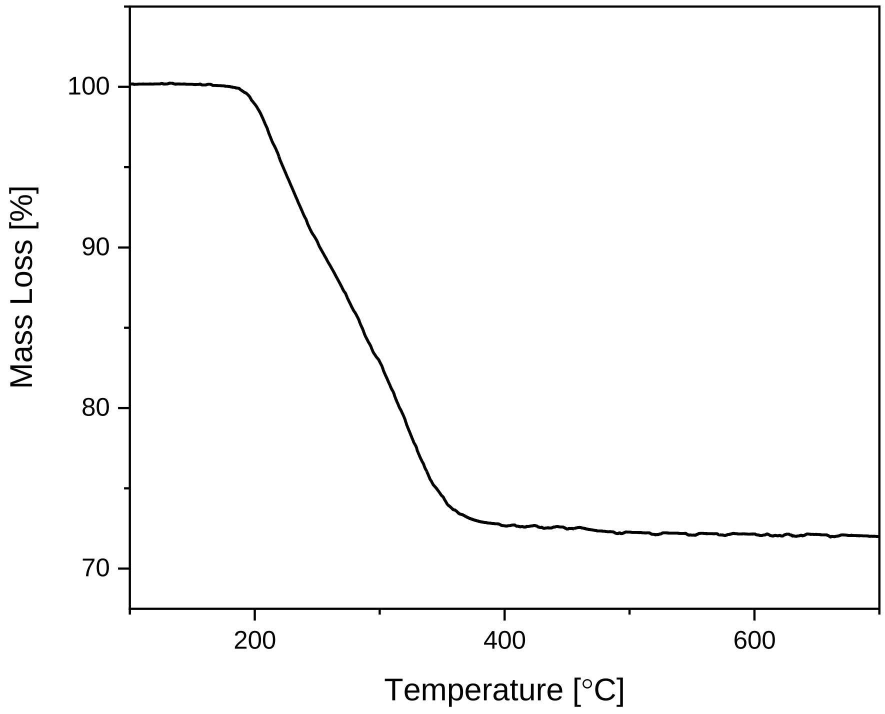


**Figure S113.** TGA Thermogram (under $N_2$) of the isolated product following treatment of $Cu_2S@(S_2CR^1)_{0.2}$ with 0.4 equiv. of $HO_2CR^1$ at 100 °C.

## Experimental Spectra and Data Associated with Ligand Exchange Reactivity for $Cu_2O$:

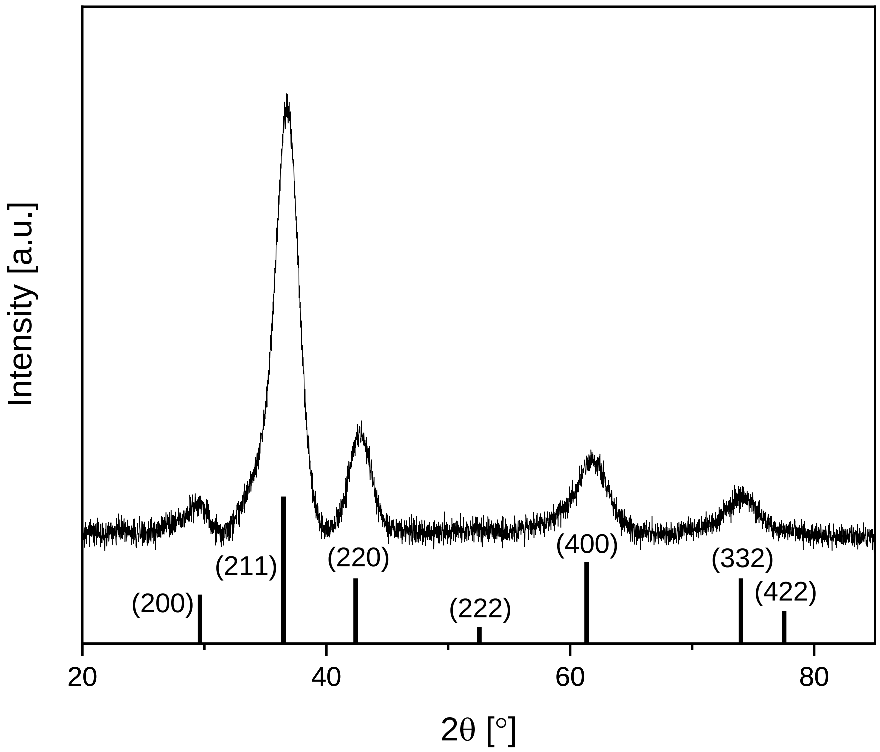


**Figure S114.** Powder X-ray diffraction pattern following treatment of $Cu_2O@(O_2CR^2)_{0.2}$ with 0.2 equiv. of $HS_2CR^1$ (3.5 nm); pattern indexed against cubic $Cu_2O$ as vertical bars (JCPDS 00-002-1067).

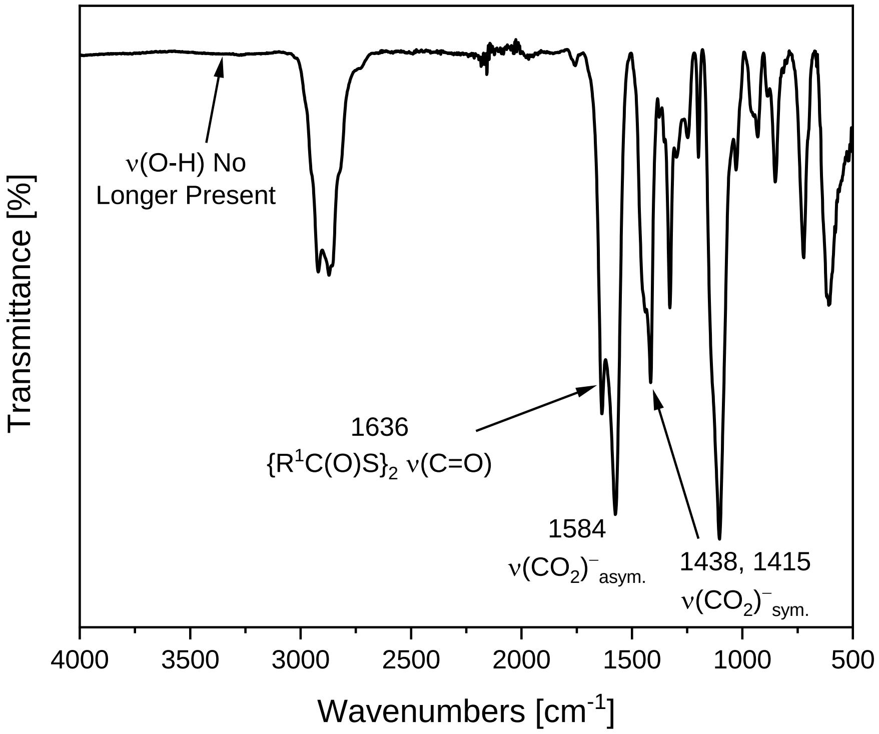


**Figure S115.** FT-IR spectrum following treatment of $Cu_2O@(O_2CR^2)_{0.2}$ with 0.2 equiv. of $HS_2CR^1$.

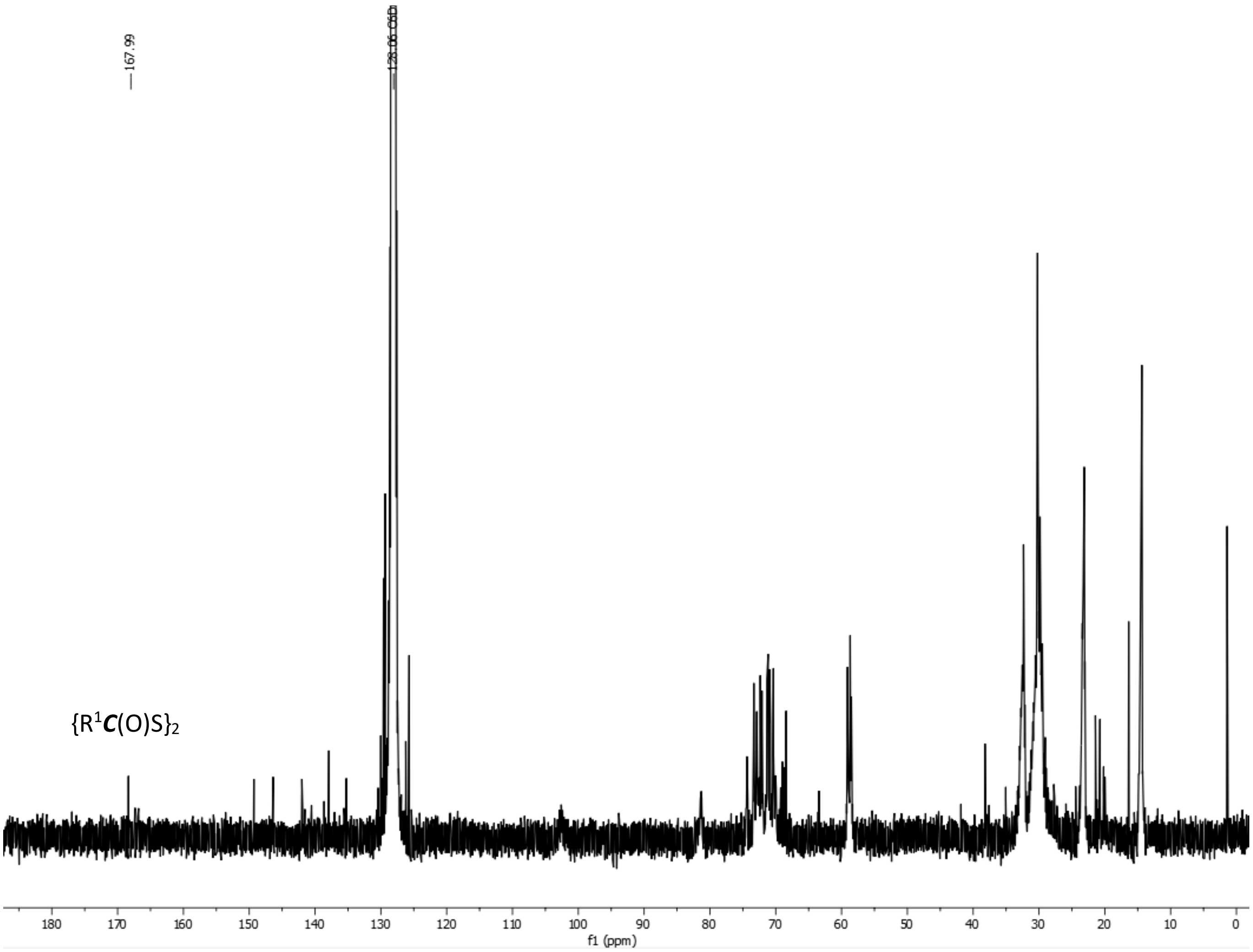


**Figure S116.** $^{13}C\{^1H\}$ NMR spectrum following treatment of $Cu_2O@(O_2CR^2)_{0.2}$ with 0.2 equiv. of $HS_2CR^1$ ($C_6D_6$, 151 MHz, 300 K).

**Table S4.** Important $^{13}C$ NMR and FT-IR data for literature diacyl disulfides.

| Compound | $^{13}C$ NMR (*C*=O) [ppm] | FT-IR v(C=O) [cm$^{-1}$] | Reference |
|---|---|---|---|
| di(benzoyl)disulfide | 186 | 1679 | [57] |
| di(heptanoyl)disulfide | 193 | – | [58] |
| di(dodecanoyl)disulfide | 194 | – | [58] |
| di(phenylacetyl)disulfide | – | 1734/1706 | [59] |
| di(ethanoyl)disulfide | – | 1736/1710 | [59] |

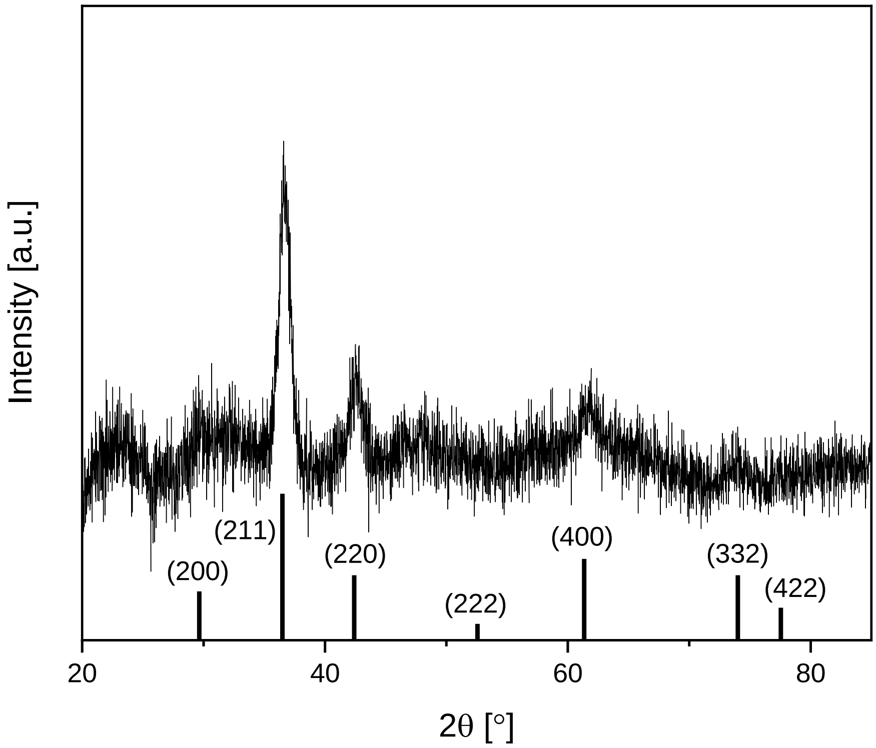


**Figure S117.** Powder X-ray diffraction pattern following treatment of $Cu_2O@(O_2CR^2)_{0.2}$ with 0.4 equiv. of $HS_2CR^1$ at room temperature; pattern indexed against cubic $Cu_2O$ as vertical bars (JCPDS 00-002-1067).

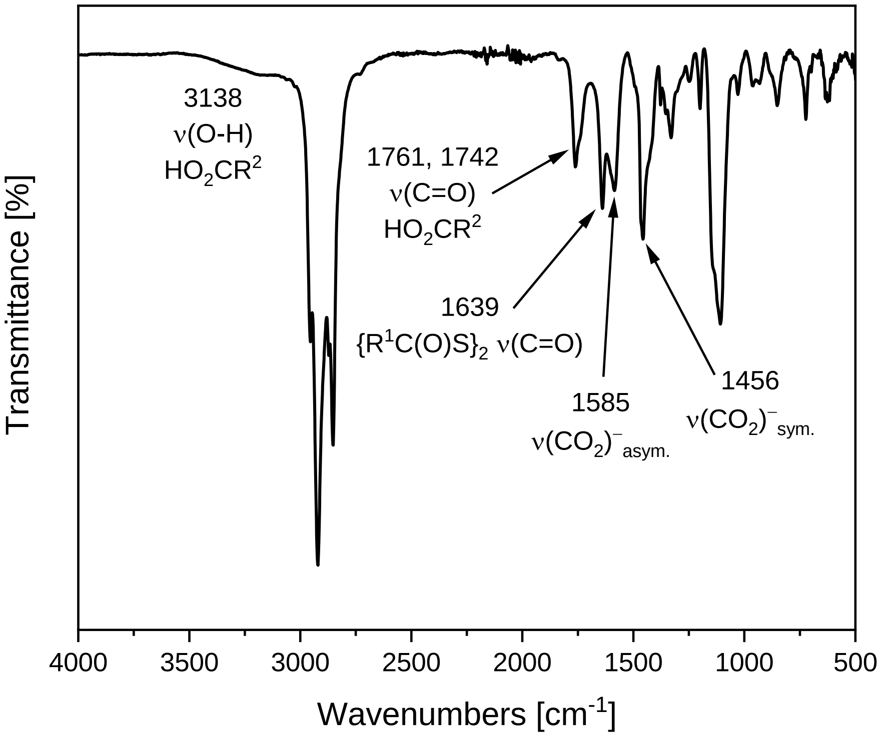


**Figure S118.** FT-IR spectrum following treatment of $Cu_2O@(O_2CR^2)_{0.2}$ with 0.4 equiv. of $HS_2CR^1$ at room temperature.

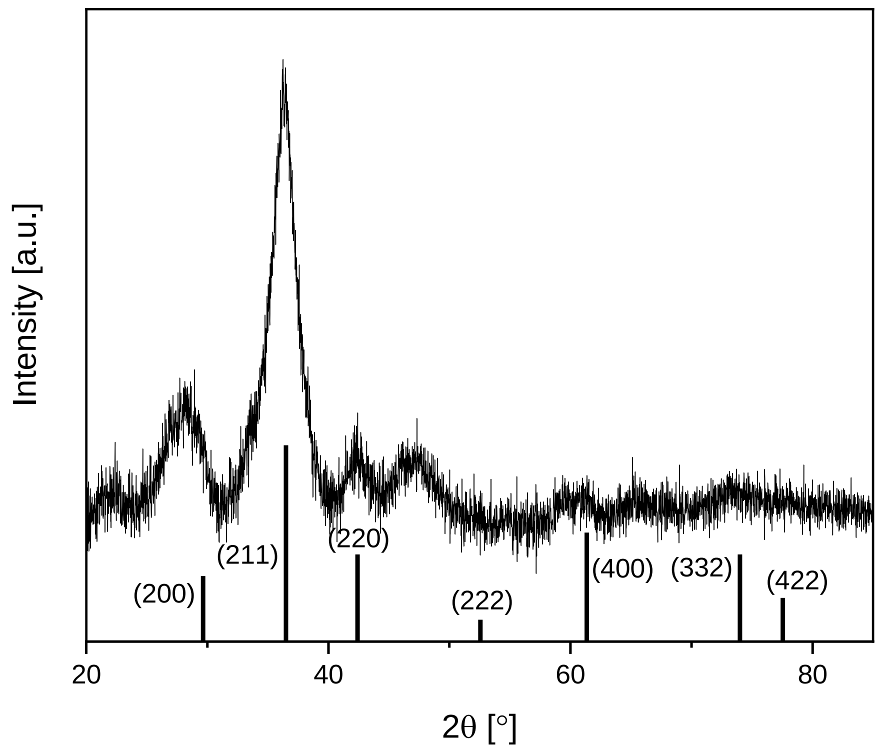


**Figure S119.** Powder X-ray diffraction pattern following treatment of $Cu_2O@(O_2CR^2)_{0.2}$ with 0.4 equiv. of $HS_2CR^1$ at 100 °C; pattern indexed against cubic $Cu_2O$ as vertical bars (JCPDS 00-002-1067).

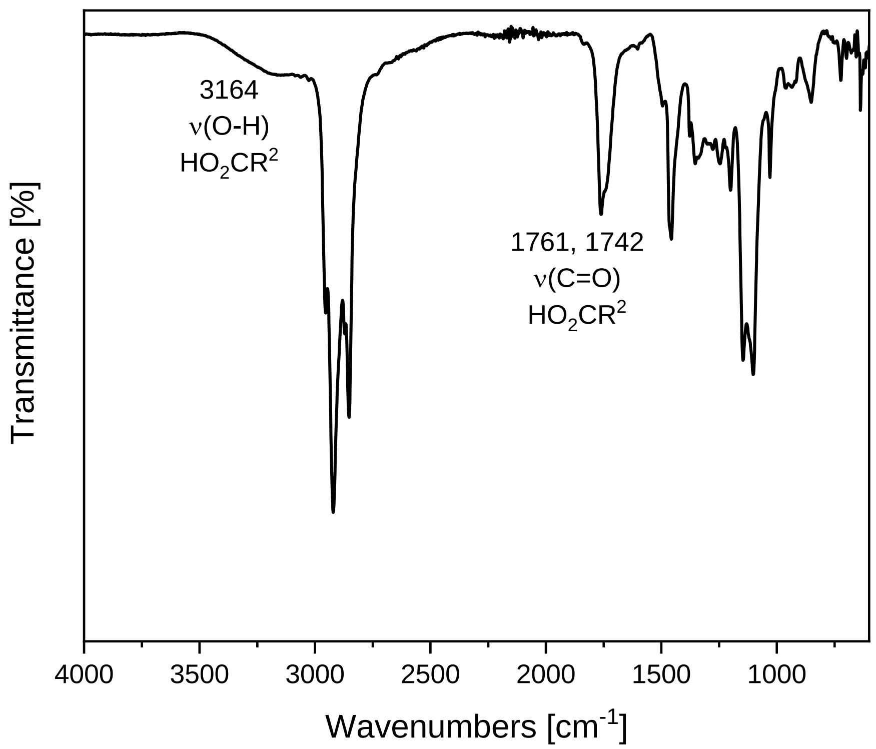


**Figure S120.** FT-IR spectrum following treatment of $Cu_2O@(O_2CR^2)_{0.2}$ with 0.4 equiv. of $HS_2CR^1$ at 100 °C.
